# A Novel Extracellular Metallopeptidase Domain Shared by Animal Host-Associated Mutualistic and Pathogenic Microbes

**DOI:** 10.1371/journal.pone.0030287

**Published:** 2012-01-27

**Authors:** Sirintra Nakjang, Didier A. Ndeh, Anil Wipat, David N. Bolam, Robert P. Hirt

**Affiliations:** 1 Institute for Cell and Molecular Biosciences, Newcastle University, Newcastle upon Tyne, United Kingdom; 2 School of Computing Science, Newcastle University, Newcastle upon Tyne, United Kingdom; Russian Academy of Sciences - Institute for Biological Instrumentation, Russian Federation

## Abstract

The mucosal microbiota is recognised as an important factor for our health, with many disease states linked to imbalances in the normal community structure. Hence, there is considerable interest in identifying the molecular basis of human-microbe interactions. In this work we investigated the capacity of microbes to thrive on mucosal surfaces, either as mutualists, commensals or pathogens, using comparative genomics to identify co-occurring molecular traits. We identified a novel domain we named M60-like/PF13402 (new Pfam entry PF13402), which was detected mainly among proteins from animal host mucosa-associated prokaryotic and eukaryotic microbes ranging from mutualists to pathogens. Lateral gene transfers between distantly related microbes explained their shared M60-like/PF13402 domain. The novel domain is characterised by a zinc-metallopeptidase-like motif and is distantly related to known viral enhancin zinc-metallopeptidases. Signal peptides and/or cell surface anchoring features were detected in most microbial M60-like/PF13402 domain-containing proteins, indicating that these proteins target an extracellular substrate. A significant subset of these putative peptidases was further characterised by the presence of associated domains belonging to carbohydrate-binding module family 5/12, 32 and 51 and other glycan-binding domains, suggesting that these novel proteases are targeted to complex glycoproteins such as mucins. An *in vitro* mucinase assay demonstrated degradation of mammalian mucins by a recombinant form of an M60-like/PF13402-containing protein from the gut mutualist *Bacteroides thetaiotaomicron.* This study reveals that M60-like domains are peptidases targeting host glycoproteins. These peptidases likely play an important role in successful colonisation of both vertebrate mucosal surfaces and the invertebrate digestive tract by both mutualistic and pathogenic microbes. Moreover, 141 entries across various peptidase families described in the MEROPS database were also identified with carbohydrate-binding modules defining a new functional context for these glycan-binding domains and providing opportunities to engineer proteases targeting specific glycoproteins for both biomedical and industrial applications.

## Introduction

The cells of our resident microbiota are estimated to outnumber human cells by factor of 10 and to encode *in toto* a significantly more extensive proteomes than the human genome [1]. This vast microbial proteome can be considered as an extension of our own as microorganisms are known to mediate numerous metabolic capabilities not carried out by mammalian cells and influence important aspects of human development, immunity and nutrition [1], [2]. The symbiotic relationship between humans and their microbiota ranges from mutualism through commensalism to parasitism, which can be considered to form a continuum rather than discretely defined phenotypes [3]. Despite the importance of the human microbiota to health there are currently significant gaps in our understanding of the molecular basis of host-microbe interactions, in particular for mutualistic outcomes. Hence there is currently tremendous interest in investigating the proteome complement of the human mucosal microbiota, as the mucosal surfaces are the dominant interface for host-microbe interactions, with microbial cell surface and secreted proteins likely representing key players mediating interactions for both mutualistic and pathogenic outcomes [4], [5], [6]. The mucus gel, the defining feature of mucosal surfaces, acts as an important defensive layer protecting the underlying epithelial cells from chemical, physical and microbial attacks [7], [8], [9]. Indeed, many pathogens produce adhesins that binds to, and enzymes that degrade, mucins, the major component of mucus to enable access to the underlying cells and tissue [7], [10]. In addition a small fraction of gut mutualists are also known to degrade mucins, which represent an important source of nutrients for these microbes and contributes to the overall mucosa homeostasis [8], [9]. Mucins are a family of high molecular weight glycoproteins composed of a linear peptide backbone heavily decorated with long oligosaccharide side chains [7], [9]. These sugar chains are usually *O*-linked and can make up 50–80% of the mucin by weight. Degradation of mucins thus requires the concerted action of both glycosidases and peptidases [9], [10] but nothing is currently known about such peptidases among mutualists [9].

Unique or enriched proteins/protein domains encoded by microorganisms sharing a given phenotype/trait, including the capacity to thrive in a given habitat, can be revealed through comparative genomics [11], [12], [13], [14], with such genotypic features being thought to correspond to specific adaptations of the autochthonous microbes for their habitat(s) of predilection. The availability of vast, and rapidly expanding, genome sequence databases enables comparative genomics to be performed over a wide range of organisms across the three domains of cellular life, encompassing a broad diversity of habitats [15]. Current sequencing technologies are also enabling metagenomic studies of microbial communities from various habitats providing additional opportunities to generate more comprehensive understanding of the molecular basis of host-microbes associations [1], [2], [16], [17].

The elucidation of the genomes of two important human mucosal pathogens, the microbial eukaryotes *Entamoeba histolytica*, a pathogen of the gastrointestinal tract (GIT) [18], [19], and *Trichomonas vaginalis* a pathogen of the urogenital tract (UGT) [20], [21], identified several genes and gene families encoding putative enzymes and surface proteins shared with other mucosal microbes through lateral gene transfers (LGT), including pathogenic and mutualistic Bacteria [22], [23], [24]. One family of *T. vaginalis* candidate surface proteins, with two members recently shown to be expressed on the cell surface of analysed clinical isolates [25], showed significant sequence similarities [23] to an *E. histolytica* immuno-dominant surface protein [26]. However, little is currently known about the function of these surface proteins from *E. histolytica* or *T. vaginalis*. The *E. histolytica* protein contains a domain with similarity to carbohydrate-binding module (CBM) from family 32 and was recently shown to accumulate at the surface of the parasite uropods [27] and might be involved in phagocytosis [28], [29]. CBMs are discrete folded domains that bind complex glycans and are normally found as ancillary modules in carbohydrate-active enzymes [30], [31]. They are organised into sequence-based families and display specificity for a wide range of mainly polymeric saccharide ligands [32]. While ligand specificity within a family is often not conserved it can be indicative of the likely activity of an uncharacterised CBM sequence belonging to that family.

Here we present the *in silico* characterisation of a novel protein domain shared between the *E. histolytica* and *T. vaginalis* surface proteins in relation to their taxonomic distribution, structural organisation and potential functions. Our analyses demonstrated that the novel domain (Pfam entry PF13402, named M60-like/PF13402) defines a new sub-family of extracellular zinc (Zn)-metallopeptidases that are conserved amongst a range of host-associated bacterial and eukaryotic microbes including mutualists and pathogens of invertebrates and vertebrates. The great majority of microbial M60-like/PF13402 containing proteins possess surface anchoring motifs and the putative peptidase domain was often associated with sequences that have been implicated in complex glycan recognition such as CBMs. Biochemical analyses demonstrated that an M60-like/PF13402 protein from *Bacteroides thetaiotaomicron*, a prominent member of our indigenous gut microbiota, displayed metal and a catalytic glutamate residue dependent proteolytic activity against mammalian mucins, identifying the first peptidase with mucinase activity from a human mutualist. These data strongly support the hypothesis that M60-like/PF13402 containing proteins play important roles in colonisation of the invertebrate digestive tract and vertebrate mucosal surfaces by a broad diversity of mutualistic and pathogenic microbes.

## Results

### Identification of a new protein domain and profile construction

**Figure 1:**
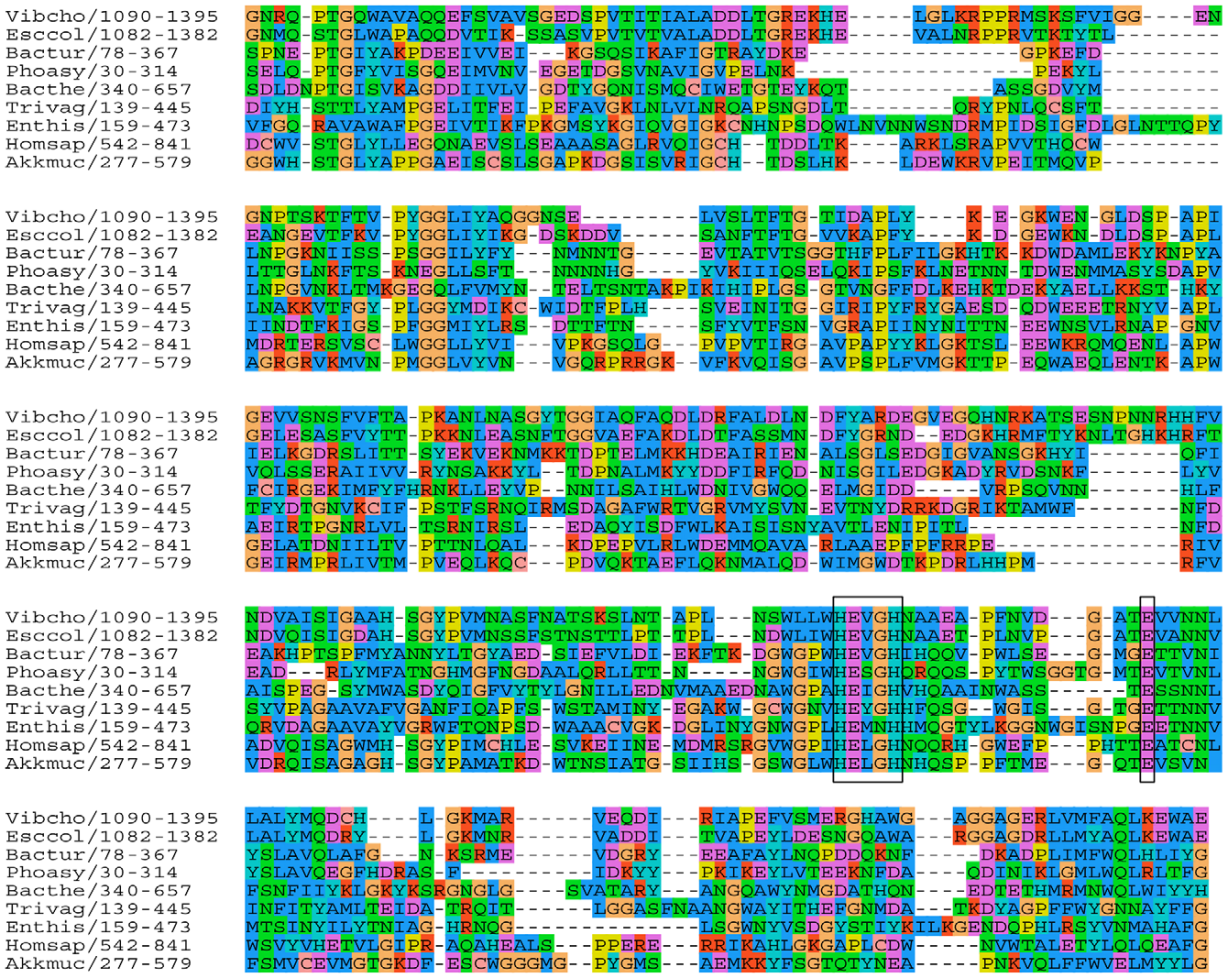
Multiple sequence alignment of proteins with the M60-like/PF13402 domain. Six selected proteins from the PF13402 seed alignment are indicated with their abbreviated species names (first three letters of genus and species name) followed by the position of the PF13402 domain. Three sequences (Vibcho, Bacthe, Enthis) were added and aligned to the PF13402 seed alignment using the MUSCLE profile alignment option in SEAVIEW. Full taxa names and GI and RefSeq accession numbers are: *Vibrio cholerae*: GI:297579165, ZP_06941093.1; *Escherichia coli*: GI:91212381, YP_542367.1; *Bacillus thuringiensis*: GI:228930091, ZP_04093101.1; *Photorhabdus asymbiotica*: GI:253989814, YP_003041170.1; *Bacteroides thetaiotaomicron*: GI:29349652, NP_813155.1 (BT4244); *Trichomonas vaginalis*: GI:123975108, XP_001330197.1; *Entamoeba histolytica*: GI:67478183, XP_654508.1; *Homo sapiens*: GI:293651621. NP_001123498.2 and *Akkermansia muciniphila*: GI:187736004, YP_001878116.1). The colour coding of amino acids indicate residues with similar physicochemical properties as defined in the alignment editor SEAVIEW. The minimal zincin metallopeptidase HEXXH motif and the additional conserved glutamate (E), defining the gluzincins-like motif HEXXHX(8,24)E, are boxed.

Based on BlastP top hits we annotated a protein family of *T. vaginalis* as candidate surface immuno-dominant proteins [20], [23] sharing sequence features with a immuno-dominant surface protein from *E. histolytica* [26]. The next most significant hits included proteins from bacteria known to be able to thrive on mammalian mucosal surfaces including, *Mycoplasma penetrans* (a Tenericutes) a human mucosal pathogen that can infect the UGT and respiratory tract (RT) [33] and *Clostridium perfringens* (a Firmicute) that can infect the GIT of various mammals [34]. Related proteins encoded by mammalian genomes were also identified (data not shown). The residues 100–500 of the N-terminus of one member of *T. vaginalis* candidate surface protein family (NCBI GI:123449825, XP_001313628, 1247 residues) were co-aligned with sub-regions of related sequences in PSI-Blast searches (Figure S1). However, no known functional features were detected in the corresponding segment when scanning the *T. vaginalis* query sequence against an integrated database of protein domains and functional sites InterPro [35]. The absence of recognised features on the broadly conserved regions suggested the discovery of a new protein domain. Hence a more specific PSI-Blast search, restricted to the first 500 residues of the same query sequence, was performed against the RefSeq protein database and identified 552 proteins (e-value ≤1.00E-4) from 333 different species/strains (Table S1). The hit list was characterised by a highly patchy taxonomic distribution including eukaryotes, bacteria and baculoviruses (Table S1). For the 333 resulting taxa, 67% of them had one protein sequence matching the query sequence. Another 30% had two to four proteins, each with one hit from the query sequence. Two microbial species living on mammalian mucosa, *T. vaginalis* and *Bacteroides caccae* were endowed with the largest hit list with 26 and 16 distinct annotated proteins, respectively. A multiple sequence alignment was generated with the sequences from the PSI-Blast hit list in order to investigate the features of the conserved domain. Maximising conservation levels of aligned sites (see Methods) and removing redundant and partial sequences over the conserved segment resulted in an alignment of 206 columns across 27 sequences (Figure S2). This alignment was submitted to the Pfam database and was confirmed to represent a novel domain. Following curation at the Pfam database the original alignment was extended to a broader range of sites (387 columns) and sequences (68 entries) (Figure 1, Figure S3) and used to generate a HMM profile defining the Pfam entry PF13402.

### The PF13402 domain is related to M60-enhancin Zn-metallopeptidases

The newly generated profile was used to search the RefSeq protein database (retrieved date: 20^th^ January 2010) with HMMER identifying 523 significant hits (e-value ≤1.00E-5) derived from 322 taxa, including a subset of those hit by the PSI-Blast search and six additional entries (Table S2). These included members of seven major bacterial and eukaryotic taxa and baculoviruses (Table S2), in line with the initial PSI-Blast searches (Table S1). The great majority of these proteins, 489 entries (93%), possess the HEXXH motif (Table S2) that was aligned to each other in a global alignment (Figure 1). This motif is characteristic of a broad range of functionally characterised Zn-metallopeptidases (where it is called the zincin motif) where the two histidine residues are ligands of a catalytic Zn^++^ and the glutamate residue represents the single catalytic amino acid residue [36]. An additional conserved glutamate was also aligned across the related sequences defining the pattern HEXXHX(8,28)E (Figure 1, Table S2). This motif is suggestive of a gluzincin-like family of Zn-metallopeptidases, where the second conserved glutamate potentially acts as a third proteous Zn^++^ ligand [36].

**Figure 2:**
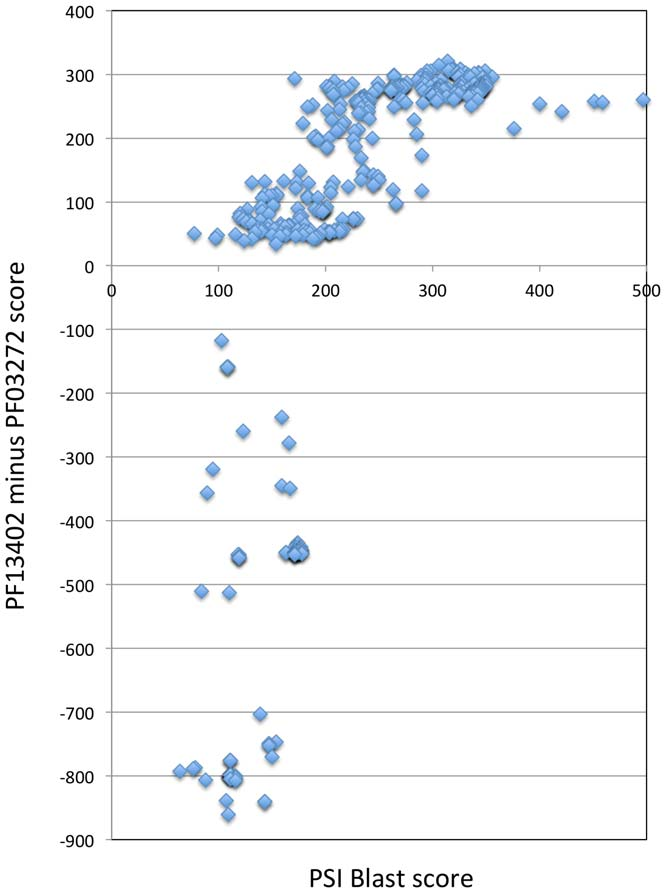
The M60-like/PF13402 and M60-enhancin/PF03272 profiles are derived from distantly related proteins. The bit score for the RefSeq proteins hit by a PSI-Blast search (X-axis) (query: *T. vaginalis* protein GI:123449825, XP_001313628; e-value ≤1.00E-04) were plotted against the difference of the HMMER bit scores for the PF13402 and PF03272 profiles (PF13402 minus PF03272)(Y-axis). A total of 415 entries were more significant for the PF13402 profile (positive values) and the remaining entries were more significant for the PF03272 profile (negative values). These results indicate that the M60-like/PF13402 and M60-enhancin/PF03272 profiles hit related proteins forming at least two distinct subfamilies defined by these two profiles that are not well discriminated by the PSI-Blast search. As expected the PSI-Blast derived profile is clearly more similar to the PF13402 profile than to the PF03272 profile as the query sequence used for the PSI-Blast search is hit by the PF13402 profile (bit-score 260, e-value 1.40E-74) but is not hit by the PF03272 profile. All values are listed in Table S2.

**Figure 3:**
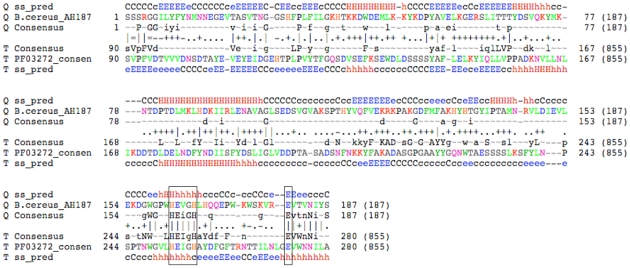
Profile-profile alignment of M60-like/PF13402 and M60-enhancin/PF03272. The alignment was derived from an HHpred search against all databases using the M60-like/PF13402 seed alignment as query (Table S4). The alignment corresponds to the second most significant hit to the enhancin/PF03272 profile (e-value 2.2E-37, score 307.8 and Probability 100%). The shown lines consists of ‘SS_pred’ lines representing sequence secondary structures predicted by PSIPRED, ‘consensus’ lines showing the consensus sequences of the PF13402 domain (shown with the top sequence) and the corresponding PF03272 domain. Amino acid residues are marked in capital letters when occurring with a frequency ≥60% and lower cases when ≥40% in the respective seed alignments. A tilda indicates an un-conserved column. The line in between the two consensus sequences shows the match quality and is defined as follows: ‘ = ’ very bad match, ‘−’ bad, ‘.’ neutral, ‘+’ good match and ‘|’ very good match. The well-conserved zincin HEXXH motif and an additional glutamate (E) residue part of a potential gluzincin are boxed. The upper and lower case letters in ‘SS_pred’ lines for secondary structure predictions show high and low probability respectively where H = helix, E = strand, and C = coil.

Consistent with this hypothesis entries positive for the PF13402 profile were also positive for a pattern characteristic of some Zn-metallopeptidases (PROSITE entry PS00142, 94 entries) or hit by the M60-enhancin domain (HMMER search with Pfam entry PF03272, 111 entries) (Table S2) with enhancin being a Zn-metallopeptidase that is a well established baculovirus virulence factor [37], [38]. However the majority (72%) of the 523 entries positive for the PF13402 profile were not positive for either of these two sequence features despite possessing the pattern HEXXHX(8,28)E. These observations prompted us to search the MEROPS peptidase database ([39] - retrieved date: 2^nd^ May 2010) with the PF13402 profile. Significant hits (with a conservative cut-off e-value ≤1.00E-5) included 38 entries and these are all members of the family M60-enhancin peptidase, with the three most significant hits being from *Bacillus cereus* (two entries) and *Akkermansia muciniphila,* a mucin degrading bacterium from the human gut [40] (Table S3). The Zn-metallopeptidase domain of the M60-enhancin annotated peptidases in MEROPS overlapped with the new domain and included the shared HEXXHX(8,28)E pattern. Of these 38 MEROPS entries, 35 were identified as having the M60-enhancin/PF03272 domain by InterProScan (Table S3). The three most significant hits (lowest e-values) for the PF13402 profile corresponded to the three entries negative for the M60-enhancin/PF03272 profile (Tables S2, S3). Plotting the difference of the HMMER search bit scores between profiles PF13402 and PF03272 against the PSI-Blast scores indicated that the PF13402 profile correlated better with the PSI-Blast profiles than the PF03272 profile does (Figure 2). The most significant hits for the M60-enhancin/PF03272 profile corresponded to the least significant hits for the PF13402 and PSI-Blast profiles, consistent with the characterisation of a new protein domain (Figure 2, Table S2). The difference of the HMMER search bit scores between profiles PF13402 and PF03272 defined a total of 415 entries positive for the PF13402 profile (Figure 2 and Table S2). The remaining entries positive for the PF03272 profile (enhancin-like entries) were from two Fungi, bacteria and baculoviruses (Table S2) of which 48 taxa also encoded one or more entries positive for the PF13402 profile (two Fungi and 46 Firmicutes)(Table S2). Hence we named the new domain and corresponding profile M60-like/PF13402 to clearly differentiate it from the related M60-enhancin/PF03272 profile. To further investigate the possibility that the M60-like/PF13402 domain corresponds to Zn-metallopeptidases we performed HMM-HMM profile comparisons (Table S4). The first hit for the M60-like/PF13402 profile corresponds to a PANTHER family (PTHR15730). However the PTHR15730 profile has no known assigned function (Table S4) and it is about three times longer than the M60-like/PF13402 profile (928 and 307 positions, respectively). The second hit corresponds to the M60-enhancin/PF03272 profile where the aligned positions with the M60-like/PF13402 included the motif HEXXHX(8,28)E (Figure 3, Table S4). Similarly to the PTHR15730 profile, the M60-enhancin/PF03272 profile is longer than the M60-like/PF13402 profile (775 and 307 positions, respectively). Moreover, searching the RefSeq database with the PTHR15730 or M60-enhancin/PF03272 profile recovered fewer entries (e-value cut off ≤1.00E-4; 127 and 212 entries, respectively) (Table S5). Among the 415 identified M60-like/PF13402 entries, the PTHR15730 and M60-enhancin/PF03272 profiles recovered only 127 and 35 entries, respectively, further illustrating the importance of the new profile PF13402 (Table S5). The following HMM-HMM profile hits were drastically less significant and corresponded to other Zn-metallopeptidase profiles with the shared zincin HEXXH motif being co-aligned between the profiles (Table S4) in all cases.

Taken together these different considerations strongly support the hypothesis that the newly defined M60-like/PF13402 domain corresponds to a novel Zn-metallopeptidase sub-family related to the M60-enhancin/PF03272 member of the MA clan as defined in the MEROPS database [39].

### The M60-like/PF13402 domain is associated with host-adapted microorganisms

The 415 proteins with most significant hits for the M60-like/PF13402 profile were derived from 256 taxa across bacteria and eukaryotes (Table S5). The majority of these taxa are microorganisms, including both bacteria (Table 1) and eukaryotes (Table 2), known to be mutualists, commensals or pathogens of animal hosts. Indeed, a highly significant positive association between the M60-like/PF13402 domain distribution and animal host-adapted microorganisms was observed; with the strongest association observed for microbes able to thrive on vertebrate mucosa (Table 3). Only 17 microbial species were without any published evidence for being associated with animal hosts including one fungus and one marine choanoflagellate (Table 2) and two species of bacteria, *Pseudomonas syringae* and *Bacillus mycoides*, known as plant and fungal pathogens, respectively (Table S6). In addition to the microbial eukaryotes, proteins with M60-like/PF13402 domains were also encoded by 14 animal genomes (Table 2). Notably, the zincin motif is deteriorated in 36% (13 proteins among 36) of the M60-like/PF13402 containing proteins from vertebrates suggesting the loss of protease activity.

**Table 1:** Short list of bacterial taxa associated with animals encoding proteins with the PF13402 domain.

Taxa	Habitat - isolation source	Disease via/in	PF13402 domain per taxa	HEXXH motif	TMD	SP or LP
**Firmicutes**						
Bacillus anthracis*	Soil/IG/mammals	GIT, RT	1–2(12)	Yes	−	Yes
Bacillus cereus*	Soil/IG/GIT	GIT, RT	1–5(69)	+(68)	+(3)	+(60)
Bacillus thuringiensis*	Soil/IG	IG	1–4(35)	Yes	+(2)	+(28)
*Clostridium bartlettii* DSM 16795	GIT	-	2	Yes	−	Yes
*Clostridium difficile* QCD-32g58	GIT	GIT	1	Yes	−	−
Clostridium perfringens*	Soil/IG/GIT	GIT	1–4(16)	+(16)	−	+(14)
*Listeria grayi* DSM 20601	GIT	-	1	Yes	−	Yes
*Mollicutes bacterium* D7	GIT		1	Yes	−	Yes
*Paenibacillus larvae* BRL-230010	IG	IG	1	Yes	−	Yes
**Tenericutes**						
*Mycoplasma penetrans* HF-2	UGT, RT	UGT, RT	3	+(2)	Yes	−
**Bacteroidetes**						
*Bacteroides caccae* ATCC 43185	GIT	Opportunist	16	Yes	+(1)	+(14)
Bacteroides fragilis*	GIT	Opportunist	1(3)	Yes	−	Yes
*Bacteroides thetaiotaomicron* VPI-5482	GIT	Opportunist	4	+(3)	+(1)	+(3)
*Prevotella melaninogenica* ATCC 25845	Oral cavity	Oral cavity	1	Yes	−	Yes
*Sphingobacterium spiritivorum* ATCC 33861	Aquatic, soil, plants, human blood and urine	RT	6(12)	Yes	−	Yes
**Actinobacteria**						
*Brachybacterium faecium* DSM 4810	Poultry GIT	-	1	Yes	−	Yes
*Eggerthella lenta* DSM 2243	GIT	Bacteremia (rare)	1	Yes	−	Yes
**Proteobacteria**						
Escherichia coli*	GIT	GIT, UGT	1(27)	Yes	−	+(22)
*Escherichia fergusonii* ATCC 35469	GIT	UGT, wound	1	Yes	−	Yes
*Grimontia hollisae* CIP 101886	GIT	GIT	2	Yes	−	−
*Photorhabdus asymbiotica* ATCC 43949	IG	Human wound	1	Yes	−	−
Pseudomonas aeruginosa*	Soil/humans	Opportunist	7	Yes	−	Yes
*Salmonella enterica* subsp. Arizona serovar 62:z4,z23:– str. RSK2980	GIT	GIT	1	Yes	−	Yes
*Shewanella pealeana* ATCC 700345	Aquatic, animal hosts, squid	-	2	Yes	−	Yes
*Shigella* sp. D9	GIT	GIT	1	Yes	−	−
Vibrio cholerae*	Aquatic, animal hosts	GIT	1–2(25)	Yes	−	+(22)
*Vibrio parahaemolyticus* 16	Aquatic, animal hosts	GIT	1–2(12)	Yes	−	+(9)
Vibrio vulnificus*	Aquatic, animal hosts	GIT	1–2(3)	Yes	−	+(2)
*Yersinia enterocolitica* 8081	Animal hosts	GIT	1	Yes	−	Yes
**Verrucomicrobia**						
*Akkermansia muciniphila* ATCC BAA-835	GIT	-	4	Yes	+(1)	+(2)
*Verrucomicrobium spinosum* DSM 4136	Soil and GIT	-	1	Yes	−	Yes

Only taxa for which there is evidence for long term or transient interactions with animal hosts are listed here. The presence of the HEXXH zincin motif, transmembrane domain (TMD) and N-terminal signal peptide (SP) or lipoprotein (LP) surface anchoring features are indicated. For each species and protein features, numbers in brackets are the total number of protein sequences that have a given feature. ‘Yes’ means all the M60-like protein sequences have a given feature. ‘*’ denotes species with more than one strains having the PF13402-containing proteins. ‘−’ is used if no sequence contains a given feature. Mucosal surfaces defined in the text are indicated: GIT, UGT and RT. IG refers to invertebrate gut (including insects, nematodes or annelids). Table S5 lists all species and strains, sequence accessions and features.

**Table 2:** Eukaryotic taxa encoding proteins with the PF13402 domain.

Taxa	Habitat - isolation source	Disease via/in	PF13402 domain per taxa	HEXXH motif	TMD	SP
**Apicomplexan**						
*Cryptosporidium muris* RN66	GIT	GIT	1	Yes	−	−
*Cryptosporidium parvum* Iowa II	GIT	GIT	1	Yes	Yes	−
**Pararasalidea**						
*Trichomonas vaginalis* G3	UGT	UGT	25	+(11)	+(6)	+(4)
**Amoebozoa**						
*Entamoeba dispar* SAW760	GIT	-	1	Yes	−	+
*Entamoeba histolytica* HM-1:IMSS	GIT	GIT	1	Yes	−	+
**Choanoflagellida**						
*Monosiga brevicollis* MX1	Aquatic	-	1	Yes	−	−
**Fungi**						
*Aspergillus flavus* NRRL3357	Soil, decaying plant and animals	Plant, RT	1	Yes	−	+
*Aspergillus oryzae* RIB40	Used in fermented food production	-	1	Yes	−	+
**Metazoa**						
**Cephalochordates**						
*Branchiostoma floridae*	-	-	2	+(1)	−	−
**Bony fish**						
*Danio rerio*	-	-	4	+(3)	−	−
**Amphibians**						
*Xenopus laevis* (African clawed fog)	-	-	1	Yes	−	−
**Birds**						
*Taeniopygia guttata* (Zebra finch)	-	-	2	+(1)	−	−
**Mammals**						
*Homo sapiens*	-	-	5	+(3)	−	−
*Pan troglodytes* (chimpanzee)	-	-	2	+(1)	−	−
*Pongo abelii* (orangutan)	-	-	1	−	−	−
*Macaca mulatta* (Rhesus monkey)	-	-	1	Yes	−	+
*Bos taurus*	-	-	2	+(1)	−	−
*Equus caballus*	-	-	2	+(1)	−	−
*Canis familiaris*	-	-	3	+(2)	−	−
*Rattus norvegicus*	-	-	5	+(3)	−	−
*Mus musculus*	-	-	3	+(2)	−	−
*Ornithorhynchus anatinus*	-	-	3	Yes	−	−

The higher taxa are indicated and for Metazoans the major sub-taxa are also listed. The presence of the HEXXH zincin motif, transmembrane domain (TMD) and N-terminal signal peptide (SP) are indicated. For each species and feature, numbers in brackets are the total number of protein sequences that have a given feature. ‘Yes’ means all the M60-like protein sequences have a given feature. ‘−’ is used if no sequence contains a given feature. Mucosal surfaces defined in the text are indicated: GIT, UGT and RT. Table S5 lists all species and strains, sequence accessions and features.

**Table 3:** The PF13402 domain is positively associated with microorganisms interacting with animal host or vertebrate mucosa.

Isolation sources or habitat for microorganisms	Number of microorganisms PF13402 positive	Number of microorganisms PF13402 negative	p-values*
Animal host*	55	327	5.9E^−06^
Non-animal host	17	333	
Mucosa*	43	154	1.7E^−08^
Non-mucosa	17	303	

*Significance of the positive association for the M60-like/PF13402 domain distribution with microorganisms living in animal host or vertebrate mucosa was calculated with a hypergeometric test – see Methods. The taxa and their habitat annotation used for the test are listed in Table S16.

### Domain architectures of M60-like/PF13402-containing proteins – a predominance of glycan binding domains

**Figure 4:**
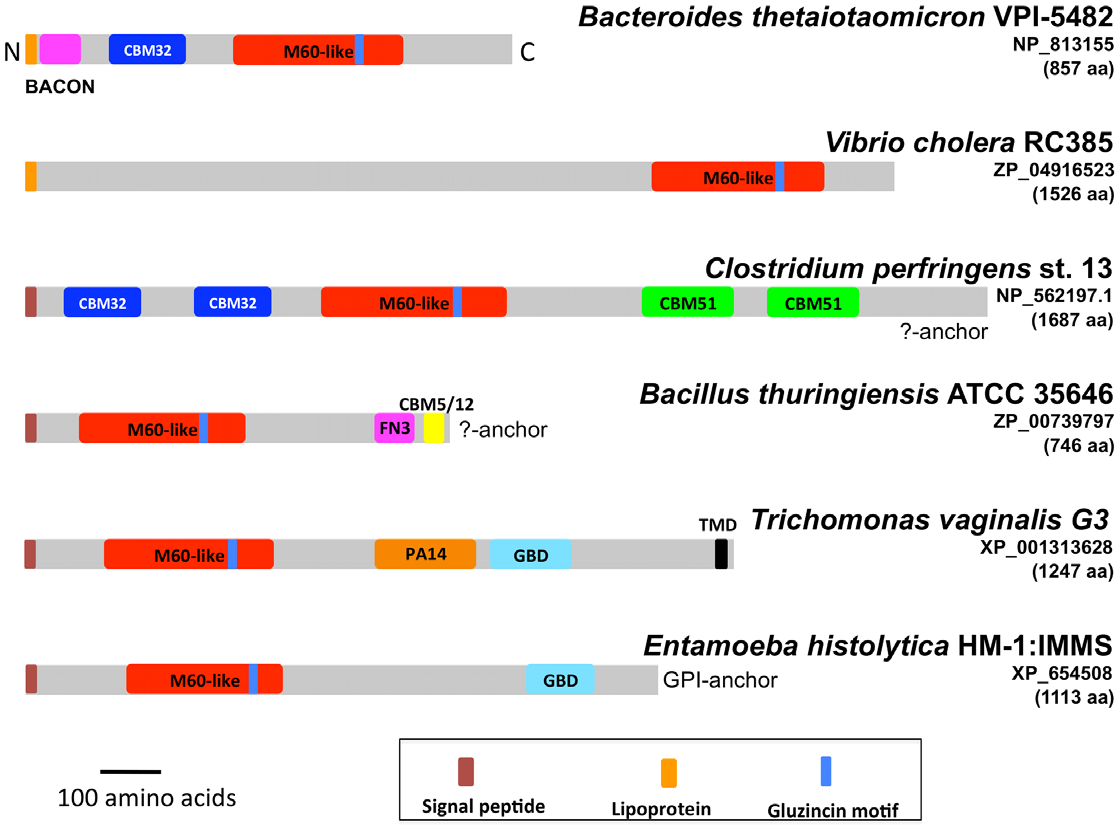
Structural organisation of selected M60-like/PF13402 containing proteins. Selected proteins possessing the PF13402 domain from a diverse taxonomic spread and cellular organisation (Gram-positive and Gram-negative bacteria and eukaryotes) with and without CBM32, CBM5_12 and/or CBM51 are depicted to illustrate their structural diversity. The putative glycan-binding BACON domain found among some *Bacteroides* spp. proteins and divergent PA14-like and GBD-like domains found among *T. vaginalis* proteins are also illustrated (see Figure S4). Specie names with accession numbers, protein length and key structural features are shown. The cartoons are drawn to the same scale and all sequences are aligned to their N-terminus. See also Figure 6 for additional structural configurations. For the *Clostridium* and *Bacillus* sequences there is no evidence for a cell surface anchoring sequence features indicated by “?-anchor”.

The majority of the 415 M60-like/PF13402-containing proteins identified in our analyses contain additional domains based on InterProScan and Pfam analyses (Table 4, Table S7). The majority of the associated Pfam domains (92%) are predicted to be involved in cell adhesion and/or glycan binding (Table 4), including CBMs that have functionally and structurally characterised entries. These are members of the CBM32 (Table S8), CBM5 and CBM12 (CBM5_12; Table S9) and CBM51 (Table S10) families respectively, as defined in the CAZy database [32] (Table 4). Members of CBM32 commonly target galactose configured animal and plant glycans and are found in a broad diversity of structural architectures [32], [41]; CBM5_12, typically found in chitinases [32], are thought to bind exclusively to chitin, a crystalline polysaccharide found in arthropods and other invertebrates, Fungi and some protists; whereas CBM51 family members are known to target galactose and blood group A/B-antigens [32], [42]. Closer inspection of the microbial distribution of the M60-like/PF13402 containing proteins linked to either CBM32 and CBM5_12 sequences revealed a distinct correlation between the CBM family and the cognate organism's predicted niche. Proteins with the CBM32 were predominantly associated with microbes known to colonise vertebrate mucosal surfaces (Table S8), whereas entries with the CBM5_12 were correlated with a capacity to thrive in the digestive tract of invertebrates, with several species being able to thrive in both insects and mammals (Table S9). One protein with CBM5_12 was derived from the fungal pathogen *Bacillus mycoides* (Table S9). The genomes from three *Bacillus cereus* strains and two *B. thuringiensis* strains encoded two to three M60-like/PF13402 containing proteins with one possessing a CBM32 and the other a CBM5_12 (Table S11). Interestingly *B. cereus* strains are known to be able to infect mammals and/or insects [43]. The 19 entries possessing CBM51 were all from the genus *Clostridium* (nine strains of *C. perfringens*, *C. bartlettii* DSM 16795 and *Clostridium* sp. 7_2_43FAA) (Table S10). A total of 16 CBM51 containing proteins also possessed a CBM32 (Table S10). In addition to known CBM families, the recently identified BACON domain [44] was identified among 22 *Bacteroides* proteins (Table S12). We also identified PA14-like [45] and CBM32-like domains in proteins from *T. vaginalis* using HMM profile-profile searches (Figure S4). Both the BACON and PA14 domains are thought to be involved in glycan binding [44], [45]. The structural organisation of selected M60-like/PF13402 containing proteins is illustrated in Figure 4.

**Table 4:** Pfam domain composition for the 415 M60-like/PF13402-containing proteins.

Pfam accession	Domain description	Number of domains	Number of proteins
PF00754^a^^,^ b	Coagulation factor 5/8 type, C-terminal – CBM32	109	78
PF00041^a^	Fibronectin, type III	40	30
PF02839^a^^,^ b	Carbohydrate-binding family V/XII – CBM5_12	28	24
PF13004^a^^,^ b	BACON	39	22
PF08305^a^^,^ b	Glycosyl hydrolase family 98, putative carbohydrate-binding module – CBM51	46	18
PF01011	Pyrrolo-quinoline quinone repeat	16	16
PF07523^a^	Bacterial Ig-like	23	15
PF07554^a^^,^ b	Uncharacterised sugar-binding	6	6
PF00746	Surface protein from Gram-positive cocci, anchoring region	5	5
PF00652^a^^,^ b	Ricin B lectin	4	4
PF00404^a^	Dockerin type 1	2	2
PF05738^a^	Collagen-binding surface protein Can-like, B region	2	2
PF00395	S-layer homology region	3	1
PF01416	tRNA pseudoouridine synthase	2	1
PF02368^a^	Bacteria Ig-like, group 2	2	1

The CBMs, numbering according to the CAZy database discussed in the text, are indicated for the description of the domains PF00754, PF02839 and PF08305. The entries are ranked according to the number of different proteins possessing a given domain.

a Domains potentially involved in adhesion activities.

b Domains potentially involved in glycan binding.

The unexpected M60-like/PF13402-CBM combinations we observed led us to ask how commonly CBMs are linked to peptidases by searching the MEROPS database for annotated peptidases possessing CBM5_12, CBM32 or CBM51. Using HMMER searches with a conservative cut off value (e-value ≤1.00E-5) we identified 141 MEROPS entries positive for CBM32 and/or CBM5_12. None were positive for CBM51. A total of 110 proteins from 16 peptidase families were positive for the CBM32 domain (Table 5), whereas 31 proteins from nine peptidase families were positive for the CBM5_12 domain (Table 6), indicating that these CBMs are widely distributed across annotated peptidases. One MEROPS entry from *Vibrio campbellii* (MER166461, ZP_02194874) was positive for both CBM32 and CBM5_12 and is a member of the Zn-metallopeptidase family M64.

**Table 5:** Annotated MEROPS peptidases families with CBM32.

Peptidase family	Bacteria	Eukaryotes	Total
M04	1	-	1
M06	4	-	4
M12A	-	1	1
M12B	-	6	6
M14B	-	65	65
M14X	-	2	2
M20A	2	-	2
M23B	3	1	4
M36	3	-	3
M60	1	-	1
M64	1*	-	1
S01A	-	4	4
S08A	7	1	8
S45	4	-	4
S63	-	3	3
T06	-	1	1
**Total**	**26**	**84**	**110**

The number of peptidases for a given family possessing at least one CBM32 is indicated.

*The unique entry with both CBM32 and CBM5_12 (Table 6).

**Table 6:** Annotated MEROPS peptidases families with CBM5_12.

Peptidase family	Archaea	Actinobacteria	Firmicutes	Proteobacteria	Total
M04	-	2	-	-	2
M06	-	-	2	-	2
M28A	-	1	-	-	1
M60	-	-	1	-	1
M64	-	-	-	1*	1
M66	-	-	-	1	1
S01A	-	9	-	5	14
S08A	2	-	1	5	8
S53	-	1	-	-	1
**Total**	**2**	**13**	**4**	**12**	**31**

The Number of peptidases for a given family possessing at least one CBM5_12 is indicated.

*The unique entry with both CBM5_12 and CBM32 (Table 5).

In contrast to the M60-like/PF13402 containing proteins the domain composition of M60-enhancin/PF03272 containing proteins was much less diverse (five additional domains) and shared with the former CBM5_12 and fibronectin type III domains (compare Table 4 and Table S13).

### Microbial proteins with the M60-like/PF13402 domain possess features of extracellular proteins

Most of the 415 M60-like/PF13402-containing proteins (76%) were predicted to possess a signal peptide (SP), one or more transmembrane domains (TMDs) or a bacterial lipoprotein motif (Table S5). These features suggest M60-like/PF13402-containing proteins are extracytoplasmic, either secreted or anchored at the surface of microbial cells and could therefore act on extracellular targets. In contrast, no extracellular-associated sequence features were detected in the 14 M60-like/PF13402-containing proteins from animals or the M60-like/PF13402-containing proteins from plant pathogens (six *Pseudomonas syringae* strains) (Table S5).

Similarly, the majority of the 141 non-M60-like/PF13402 MEROPS entries (72%) positive for CBM32 and/or CBM5_12 were predicted to possess a SP and/or one or more (range 1–12) TMD suggesting these peptidases also target extracellular glycoproteins (Table S14).

### Evidence for metal-dependent mucinase activity for one M60-like/PF13402-containing protein from a human gut mutualist

The predicted peptidase and glycan binding activities, cellular location and taxonomic distribution of a number of M60-like/PF13402 containing proteins suggest their target substrates are host glycoproteins such as mucins. In addition, a previous study has shown that genes encoding two of the three M60-like/PF13402 domain containing proteins with the gluzincin motif from the human gut bacterium *Bacteroides thetaiotaomicron* (BT3015/NP_811927.1 and BT4244/NP_813155.1 in Table S5) are up-regulated in response to host *O*-glycan mucins, both *in vitro* and *in vivo* [46].

**Figure 5:**
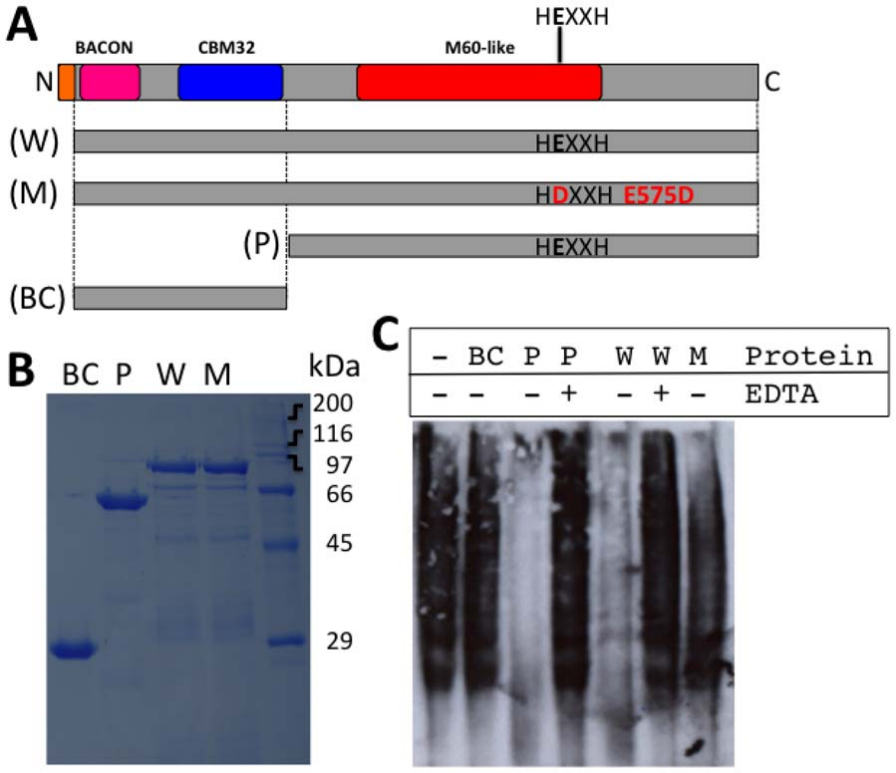
*In vitro* mucin degradation assay. (A) The structural organisation of the *B. thetaiotaomicron* BT4244 protein (GI: 29349652; RefSeq: NP_813155.1). The entire wild type (W) protein (minus signal peptide) encoded by the *BT4244* gene is contrasted to a mutant (M) (E575D) and two truncated constructs covering either the PF13402 domain (P) or the BACON-CBM32 domains (BC) only. (B) 5 µg of purified recombinant proteins were separated by SDS-PAGE and stained with Coomasie blue for detection. The predicted protein sizes in kDa are: M and W: 95.1, P: 67.3 and BC: 27.8. (C) Bovine submaxillary gland mucins were incubated with 5 µg of purified recombinant proteins W, M, P or BC in the absence (−) or presence (+) of 50 mM EDTA. Following incubation the mucin samples were separated on an agarose gel prior to detection on blots with wheat germ agglutinin lectin.

To experimentally test the hypothesis that some M60-like/PF13402 containing proteins degrade mucins we expressed and purified full-length BT4244 and constructs lacking either its N-terminal putative carbohydrate binding domains BACON and CBM32 or C-terminal M60-like/PF13402 peptidase domain and assessed their ability to degrade mucins using a gel based assay (Figure 5). The data show that the full-length recombinant protein comprising the two putative N-terminal carbohydrate binding and the M60-like/PF13402 domains, or a C-terminal fragment composed of the predicted M60-like/PF13402 peptidase domain only, both generated significant clearing of the bovine submaxillary gland mucins from the gel, indicative of degradation of the mucin peptide backbone (Figure 5). In contrast, no mucin degradation was observed in the sample containing an N-terminal segment encompassing the BACON-CBM32 domains only (Figure 5). Addition of EDTA to the full-length enzyme and peptidase domain reactions inhibited the observed shift in banding pattern, as expected if a metal was required for a proteolytic activity (Figure 5). Furthermore a conservative mutation of the predicted catalytic glutamic acid residue (E575D of the zincin motif HEXXH) dramatically reduced the mucinase activity (Figure 5). These functional data clearly support the hypothesis derived from our bioinformatics analyses that the novel M60-like/PF13402 containing proteins represent host glycoprotein degrading Zn-metallopeptidases.

## Discussion

A comprehensive understanding of the molecular basis of mammalian host-microbe associations requires the knowledge of the specific families of microbial proteins involved in interactions with host mucosal surfaces. While many proteins from microbial pathogens involved in adhesion to host tissues or degradation of host proteins (virulence factors) have been identified there is a paucity of data on the molecular basis of non-pathogenic mutualistic interactions between host and microbes, despite the importance of our microbiota in maintaining human health.

Comparative genomics can provide useful insight into structural and taxonomic or habitat contextualisations generating valuable hypotheses for the functions of uncharacterised proteins. In this study we employed *in silico* investigations and an *in vitro* mucinase assay to generate data, which together strongly support the hypothesis that we identified a novel type of Zn-metallopeptidases important for animal host-microbes interactions ranging from mutualistic to pathogenic outcomes.

### The M60-like/PF13402 domain define novel Zn-metallopeptidases

The presence of the extended consensus HEXXHX(8,28)E, suggested that the M60-like/PF13402 domain containing proteins could be considered as gluzincin metallopeptidases. Known bacterial and mammalian gluzincins have an insertion between the second H and second E, ranging from 24 to 64 amino acids [36]. However, although none of the consensus sequences for known gluzincins [36] correspond to the consensus region found among the M60-like/PF13402-posessing proteins, a minority of the M60-like/PF13402 containing proteins (23%–94 among 415) were positive for an extended pattern characteristic of known Zn-metallopeptidases (PROSITE entry: PS00142). In addition, the profiles PF13402 and PF03272 were clearly related with proteins positive for both profiles and the two profiles significantly hitting each other in profile-profile comparisons. Enhancins (defining the M60-enhancin/PF03272 domain) are insect mucin degrading Zn-metallopeptidases (Clan MA, subclan MA(E), family M60) first described in baculoviruses where they act as virulence factors [37], [38]. More recently a protein with an M60-enhancin/PF03272 domain from the insect pathogen *B. thuringiensis* (RefSeq accession: ZP_04115705.1 in Table S1) was also shown to degrade insect mucins defining a new bacterial virulence factor [47]. In insects the peritrophic membrane can be considered as analogous to the mammalian intestinal mucus, but unlike vertebrate mucus, peritrophic membranes are chitin rich matrices [48]. In vertebrates, mucus layers form an important physical surface barrier facing the external environment in the GIT, RT and UGT [8], [49]. Both vertebrate and invertebrate protective barriers play important roles in defending the digestive tract from microbial infections as well as promoting digestion processes [48], [49]. Therefore, in order for a microbe to colonise or break through these protective barriers, physical interactions and enzymes capable of processing these protective matrices, or cellular processes such as flagella mediated directed movements, are required [7], [8], [10]. For some mammalian mutualists the mucus represent an important source of food, especially when there are little exogenous nutrients available, as recently demonstrated for the prominent distal gut bacterium *B. thetaiotaomicron* [46]. Consistent with the M60-like/PF13402 domain being related to the enhancin Zn-metallopeptidases, we show here that recombinant versions of the lipoprotein BT4244 from the mutualist *B. thetaiotaomicron* displayed mucin degrading activity *in vitro* and this process was inhibited by the addition of EDTA, a metal chelator known to deactivate Zn-metallopeptidases [50] or by mutating the candidate catalytic glutamate residue of the zincins motif [51]. In addition, a previous study showed that the expression level of the *BT4244* gene increased significantly when *B. thetaiotaomicron* cells were exposed to mammalian *O*-glycan mucins *in vitro* and *in vivo* and that the gene belonged to a co-regulated polysaccharide utilisation locus (PUL#78 – spanning BT4240-50) [46]. PUL#78 also contains two glycoside hydrolase (GH) genes, a GH2 (BT4241) and GH109 (BT4243), two families that display activities consistent with mucin degradation [32], [46], [52]. Based on the results of our mucinase assay and gene content and expression data of the PUL#78 we speculate that BT4244 cleaves the peptide backbone of colonic mucins *in vivo* and contributes to host glycan foraging and niche adaptation by *B. thetaiotaomicron*. This represent the first peptidase identified for a bacterial mutualist that can target mucins [9], with proteolytic degradation of mucins thought to be important for the regulating the homeostasis and physicochemical properties of the colonic mucus and contributing to its degradation along with bacterial glycosidases [8], [9]. This process benefits the energy balance of both the bacteria and the mammalian host as short fatty acids generated by bacterial mucin fermentation are metabolised by the colonic epithelial cells [9].

### Bacterial M60-like/PF13402 domain containing proteins are encoded by the disposable pan-genome

Although proteins with an M60-like/PF13402 domain were encoded by the genomes of many different bacterial species, not all sequenced strains of a particular species (or species of a given genus) contained a copy of this gene suggesting it is part of the disposable pan-genome that contributes to specific niche adaptation, including pathogenesis [6], [16]. For example, several animal bacterial pathogens such as *Vibrio* spp. (range: 0–2 proteins per genome), including the important human pathogen *V. cholera* (Table S15), contain an M60-like/PF13402 domain gene, annotated as lipoprotein AcfD, whereas non-virulent strains of *V. cholera* do not [53]. The *V. cholera* AcfD gene is part of four genes defined as **a**ccessory **c**olonisation **f**actors (AcfA-D) required for efficient human intestinal colonisation [53], [54]. Interestingly there is evidence that the *V. cholera* AcfB-C proteins mediate host-specific chemotaxis towards mammalian mucus [53]. In line with the mucinase data for the BT4244 protein, we hypothesise that the AcfD lipoprotein is degrading human mucins, possibly in concert with an additional secreted Zn-metallopeptidase TagA [51], contributing to the pathogen's capacity to penetrate the mucus layer, a trait of virulent bacterial strains [53]. In fresh water and other aquatic environments the AcfA-D proteins could also contribute to the colonisation of fish mucosal surfaces, as *Vibrio cholera* and other *Vibrio* species are often associated with these vertebrates [55], and some species/strains can be pathogenic to both human and fish [56].

Similar to *Vibrio* spp., not all sequenced genomes from *Bacillus* spp. (range: 0–5), *Bacteroides* spp. (range: 0–16 proteins), *Clostridium* spp. (range: 0–4 proteins), *Escherichia* spp. (range: 0–1 proteins) and *Yersinia* spp. (range: 0–1 proteins) appear to encode proteins with the M60-like/PF13402 domain (Table S15). Among 32 *Escherichia spp*. possessing proteins with the M60-like/PF13402 domain 19 were defined as pathogenic strains that cause infection in various mucosal niches including GIT, UGT and RT of both mammals and birds [57] (Table S15). The other 13 *E. coli* strains encoding M60-like/PF13402 domains are defined as non-pathogenic members of the intestinal microbiota or one lab strain (Table S15). For the remaining 32 *Escherichia* species or strains there was no evidence for genes encoding any M60-like/PF13402 containing proteins (Table S15). These taxa included in particular all of the *E. coli* O157 strains, which are well known zoonotic pathogens that can lead to severe human illnesses [58]. Similarly, different *Bacteroides* spp. are thought to be adapted to different niches or food sources within the mammalian GIT with only a few species known to be able to graze host derived mucin glycans [59], [60]. The patchy distribution of M60-like/PF13402 containing proteins among *Bacteroides* species (Table S15), suggests the presence of this gene could contribute to niche specialisation by providing mucin degrading capability, a view supported by the mucinase activity displayed by BT4244 from *B. thetaiotaomicron,* a known mucin degrader.

### Pathogenic microbial eukaryotes also encode M60-like/PF13402 containing proteins

In addition to bacterial pathogens M60-like/PF13402 domains were also identified among proteins from pathogenic microbial eukaryotes including the extracellular parasites *T. vaginalis* and *E. histolytica* and the intracellular parasites *Cryptosporidium parvum* and *C. muris*. *Entamoeba* and *Cryptosporidium* species target the digestive tract of humans and other vertebrates [61] while *T. vaginalis* is a human sexually transmitted pathogen affecting both the male and female UGT [62]. The immuno-dominant surface antigen from *E. histolytica* is known as a GPI-anchored protein against which most patients with liver abscess are known to generate an immunoglobulin response [26]. Proteomics data indicated that this *E. histolytica* surface protein can be found in the parasite phagosomes and uropodes [27], [29] and it was suggested that it might be involved in phagocytosing apoptotic human cells [28]. The presence of a galactose-binding domain like sequence (GBD, related to CBM32) suggests that this domain could mediate binding to glycan chains in secreted and cell surface human glycoproteins such as mucins. The M60-like/PF13402 domain could be driving proteolysis of human glycoproteins representing a possible source of nutrients for the parasite and/or contribute to processing human proteins involved in innate and adaptive immune defences for the benefit of the microorganism. A related protein is also encoded by the genome of the commensal *E. dispar* [63]. As homologues exist in both a pathogen and a commensal, the M60-like/PF13402 containing protein might therefore not represent a virulence factor in *E. histolytica* as such but could contribute to the amoeba fitness on mucosal surfaces. As for the *E. histolytica* surface protein there is evidence for surface expression of two *T. vaginalis* M60-like/PF13402 containing proteins (GI:123975108 XP_001330197.1 and GI:123449825 XP_001313628.1 in Table S1) [25]. In contrast to the *E. histolytica* GPI-anchored proteins these *T. vaginalis* proteins possess a TMD. As *T. vaginalis* is also phagocytic [64], and mediates endocytosis [24], the M60-like/PF13402 containing proteins could be involved in nutrient binding, uptake and processing through these internalisation routes.

### The specificity of the M60-like proteins is driven by their associated carbohydrate-binding modules

A recurrent structural feature among many of the 415 identified M60-like/PF13402 containing proteins was the co-occurrence of CBMs and other glycan binding domains. Additional potential glycan-binding domains included the BACON (identified among *Bacteroides* spp.) [44] and PA14-like domains [45], [65] (identified among *Trichomonas* and *Entamoeba*). A total of 103 M60-like/PF13402 containing proteins possessed CBM32, CBM5_12 and/or CBM51 among 66 microbial species or strains that are known in their majority to colonise animal hosts. Some of these species are well known members of the human GIT microbiota including *Bacteroides thetaiotaomicron*, *B. fragilis* and *B. caccae* [2] or human pathogens including *Clostridium difficile* [66]. Others are thought to be both “free-living” (they can be isolated from the environment) and can be pathogenic when in contact with mammalian mucosal surfaces and/or the digestive tracts of insects. These species include *Paenibacillus larvae* [67], *Yersinia enterocolitica* [68], *Clostridium perfringens* [66], *C. botulinum* [69] and *Bacillus cereus* and *B. thuringiensis* [43]. The M60-like/PF13402-CBM32 proteins are from microbial species able to colonise mammalian mucosal surfaces including the GIT and the UGT ranging from mutualists to pathogens. CBM32s are known as components of enzymes involved in the processing of complex galactose configured glycans from predominantly animal sources [41]. The *B. thetaiotaomicron* M60-like/PF13402 containing protein BT4244 possesses a CBM32 and a BACON domain. These putative glycan-binding domains could contribute to mucin recognition at the surface of the bacterium by targeting the Gal and GalNAc containing *O*-glycan side chains and presenting the polypeptide to the M60-like/PF13402 Zn-metallopeptidase domain. The presence in many M60-like proteins of multiple CBMs from different families and/or in combination with other candidate binding domains (e.g. BACON-CBM32, CBM32-CBM51, PA14-like-GBD and CBM32-Fibronectin type III domain) suggests multivalent recognition of a complex ligand driving high specificity and avidity, consistent with targeting of host glycoproteins. CBMs from family 5 and 12 target chitin and were detected as components of M60-like/PF13402-containing proteins from insect pathogens such as *Paenibacillus larvae* and *Bacillus thuringiensis* as well as the fungal pathogen *Bacillus mycoides*. [70], [71]. The presence of a C-terminal CBM5_12 in the M60-like/PF13402 containing proteins from insect pathogens suggests that the target of these glycan binding domains is the chitin-rich peritrophic membrane in the insect gut. Attachment via the CBM5_12 could facilitate degradation of the protein component through the activity of the M60-like/PF13402 peptidase domain. Intriguingly, *P. larvae* is a causative agent for American foulbrood disease of honeybee larvae with the spores germinating in the gut prior to causing disease [67] and metallopeptidases were reported to be involved in *P. larvae* pathogenicity [72]. Thus the M60-like/PF13402-CBM5_12 proteins represent an attractive candidate virulence factor similar to the related baculovirus [37], [38] and bacterial enhancin Zn-metallopeptidases [47]. Most taxa encoded proteins combining the M60-like/PF13402 domain with either CBM32 or CBM5_12 but five *Bacillus* spp. (three strains of *B. cereus* and two of *B. thuringiensis*) encoded two to three proteins, each with one of these two domains combination (Table S11). Strains of *B. cereus* are known to cause disease in both mammals and insects and it would be interesting to test if these M60-like/PF13402 protein variants are differentially expressed in insect (proteins with CBM5_12) versus mammals (proteins with CBM32) hosts, possibly contributing to this *B. cereus* host promiscuity [73].

The association of CBMs with the M60-like/PF13402 peptidase domains and analysis of the MEROPS database clearly indicate a novel functional context for CBMs, which are classically associated with carbohydrate active enzymes [30], [31], [32]. In the context of host-microbe interactions the presence of CBMs linked to extracellular peptidases likely contributes to the ability of microbes to attach to, degrade and metabolise host glycoproteins including the abundant mucins. Interestingly, while the CBM domains are found at either the C-terminal or N-terminal side of the M60-like/PF13402 domain, the relative position of the protease domain when attached to the surface is often conserved, suggesting this configuration is functionally important (Figure 4). However some M60-like/PF13402 domain containing proteins, such as several entries from *Clostridium* spp. (Figures 4 and 5), possess CBMs on both sides of the protease domain indicating a variety of configurations is possible. The combination of protease-CBM5_12 domain architecture is also observed in 13 enhancins from some *Clostridium* and *Bacillus* taxa (Table S13).

### A complex evolutionary history for the M60-like/PF13402 domain

**Figure 6:**
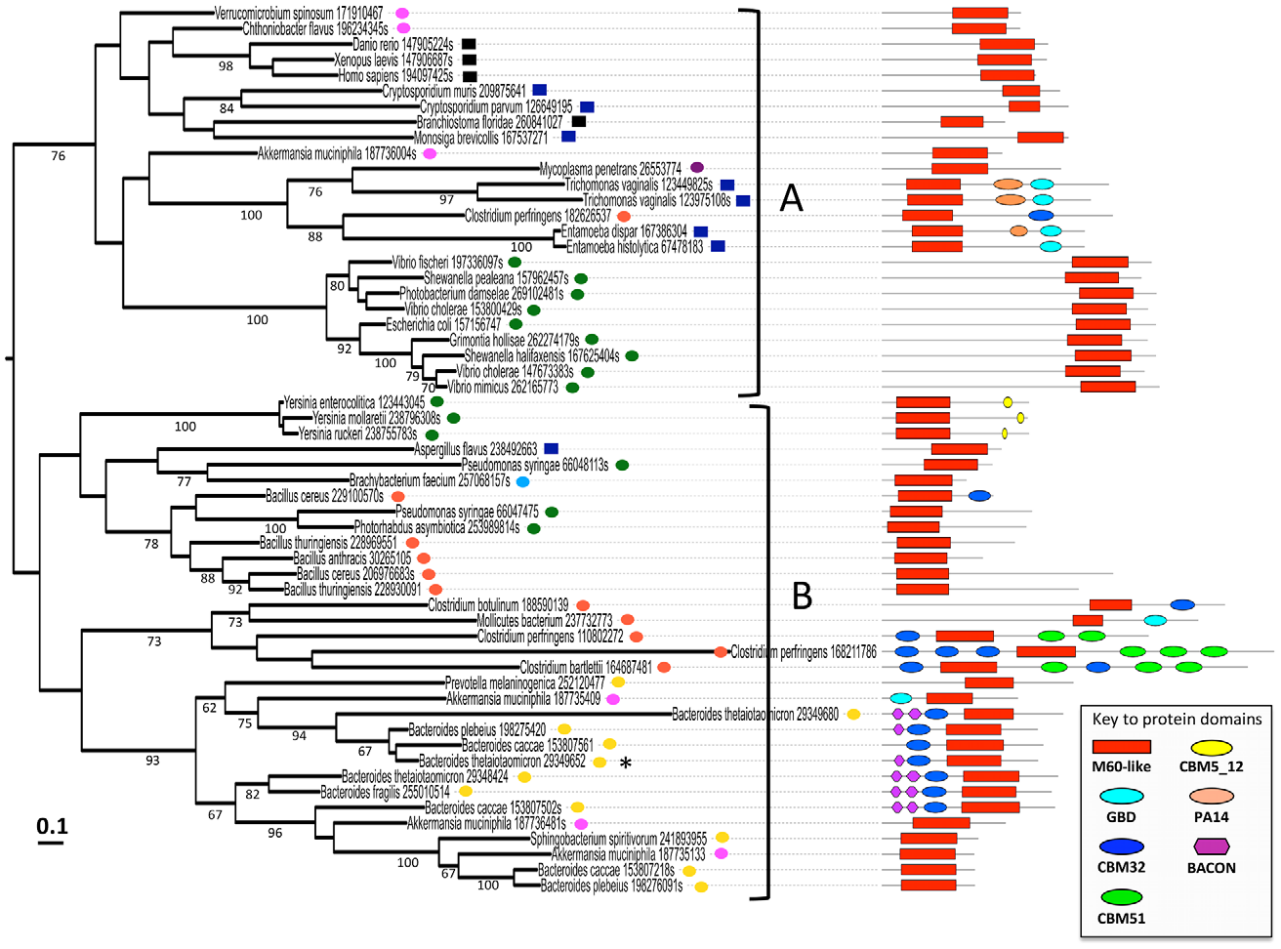
Protein maximum likelihood bootstrap consensus tree for selected M60-like/PF13402 domains. The shown maximum likelihood tree (Log likelihood: −18778.5) was generated as described in the Methods section using an alignment of 57 sequences and 175 residues drawn from an M60-like/PF13402 domain alignment, providing an evolutionary framework for the gene segments encoding these domains. For each sequence the corresponding species name is indicated along with the NCBI GI number and high-ranking taxa - squares are for eukaryotes: black-Metazoa, blue-microbial eukaryotes; circles are for Bacteria: yellow-Bacteroidetes, orange-Firmicutes, green-Proteobacteria (all gamma-proteobacteria), pink-Verrucomicrobia, violet-Tenericutes, cyan-Actinobacteria. Bootstrap support values (≥60%) are indicated below the branches. The scale bar represents the estimated number of changes per site. The domain organisation of the corresponding complete proteins is shown on the right hand side (see also Figure 4 for additional domain configurations). The sequence corresponding to the BT4244 protein used for the mucinase assay (Figure 5) is indicated by a *. The two major clans supported by a bootstrap value of 76% are indicated as clan A and B.

The broad and patchy taxonomic distributions of genes encoding proteins with the M60-like/PF13402 domain also suggest that gene sharing through LGT took place between distantly related taxa, including between bacteria and eukaryotes. Phylogenetic analyses of a representative selection of M60-like/PF13402 domain sequences strongly supports this hypothesis with in particular robust cases of gene sharing between the microbial eukaryotes *Trichomonas* and *Entamoeba* and the bacteria *Mycoplasma* and *Clostridium* respectively (Figure 6). Notably the majority of *Clostridium* species form a distinct clan (as defined in [74]), including *C. perfringens*, clearly indicating alternative origins for the M60-like/PF13402 domain among this genus. The strong bias for M60-like/PF13402 domains among microbial taxa able to colonise animal hosts, suggests that an important fraction, if not most, of these LGTs took place in the context of animal hosts where microorganisms density can be extremely high, as in the human distal colon, and where LGT is known to play important roles in shaping the gene complement of the microbial community [60]. A striking case involves four independent LGT events in the mucin degrading Verrucomicrobia, *Akkermansia muciniphila*, three from the distantly related Bacteroidetes donors (clan B in Figure 6) that share the same niche as *A. muciniphila* and a fourth LGT from an undefined source (within clan A in Figure 6). The potential initial source gene(s) for the microbial species is difficult to establish with the current taxonomic sampling and phylogenetic resolution. It could be one or more eukaryotes as a broad range of these encode M60-like/PF13402 containing proteins, indeed several bacterial sequences are part of clan A where the majority of eukaryotes, including animals sequences, are clustering (Figure 6). An animal to bacteria gene transfer was recently suggested for genes encoding two other distinct types of Zn-metallopeptidases identified in the human associated Bacteroidetes species *Bacteroides fragilis* [75] and *Tannerella forsythia* [76]. In the case of the two fungal M60-like/PF13402 containing proteins the phylogeny supports an LGT event from a bacterial donor to the *Aspergillus* lineage (only one fungal species is shown in Figure 6), possibly reflecting the adaptations of these Fungi to thrive on decaying plant and animal material. The identified LGT of genes encoding M60-like/PF13402 Zn-metallopeptidases involving mutualists, commensals and pathogens further highlights the overlap between the gene complements of microorganisms generating contrasting symbiotic outcomes with their animal hosts [4], [6], [24]. Interestingly a restricted set of taxa (two Fungi and 46 Firmicutes, Table S2), which represent a subset of the taxa encoding M60-like/PF13402 containing proteins, encoded one (or more) protein possessing the M60-like/PF13402 domain and another protein possessing the M60-enhancin/PF03272 domain with the bacteria taxa all capable to infect insects or other invertebrates (Table S2). Analysing the relationship of protein sequence members of these two protein families with CLANS (see Methods section) showed that M60-enhancin/PF03272 and M60-like/PF13402 proteins clustered in different groups further supporting our finding that the M60-like/PF13402 domain form a novel protein family (Figure S6). Interestingly, the CLANS result also suggests that the M60-enhancin/PF03272 and M60-like/PF13402 containing proteins from *Bacillus* species are more similar to each others than to other related proteins from other organisms (Figure S6). This suggests that the PF03272 domain was derived from a gene duplication of a “primordial” PF13402 domain. One possible scenario underlying the functional relevance of such a gene duplication event, followed by important differentiation of the paralogues, could be a response to the selection pressure induced by insect host peptidase inhibitors on bacterial peptidases representing virulence factors [77]. A phylogenetic analysis of the same dataset used to investigate M60-like/PF13402 domain relationships (Figure 6) complemented with selected M60-enhancin/PF03272 sequences could neither reject or provide support for this hypothesis due to lack of resolution as indicated by poor bootstrap support values (Figure S7). These evolutionary considerations along with the identified taxonomic distribution of M60-enhancin/PF03272 domains (Table S13) also suggest that the baculoviruses obtained their enhancin genes from a bacterium sharing the same insect host and subsequently diverged dramatically from their bacterial enhancin homologues.

In summary the novel type of Zn-metallopeptidase we identified across evolutionarily distantly related bacteria and microbial eukaryotes, that are found on a broad range of animal hosts, further illustrates the importance of peptidases in host-microbe interactions. This discovery will be of benefit in guiding investigations of the molecular basis of host-microbe interactions in the context of both mutualistic and pathogenic outcomes involving bacteria and microbial eukaryotes in vertebrates and invertebrates. It will be of particular interest to identify the range, and properties, of the host proteins that the novel microbial peptidases can target. The possibility of peptidases representing functional partners of other hydrolysing enzymes, such as GH (indicated by the *Bacteroides* PULs encoding both activities), to process mammalian mucins is of particular interest for the study of the role of mutualists in the homeostasis of our mucosal surfaces. The novel yet common domain combinations we identified involving peptidases and CBMs offer interesting new insights into substrate recognition by peptidases, which in turn could provide exciting opportunities to engineer peptidases targeted to specific glycoproteins for both biomedical and industrial applications.

## Methods

### Sequence similarity search and HMM profiles generation

PSI-Blast was used at the NCBI Blast server [78] (search date: 20^th^ January 2010) to identify related proteins to the *T. vaginalis* entries annotated as immuno-dominant antigen-like protein using as query the protein GI:123449825 XP_001313628 (positions 1–500). An initial PSI-Blast search with the entire GI:123449825 XP_001313628 sequence identifying the first ∼500 residues as being shared across a broad range of taxa (Figure S1), hence we used positions 1–500 to perform a most specific PSI-Blast profile search. It was performed with a standard initial BlastP search followed by one iteration step for the profile-based search. A multi-sequence alignment with the sequences derived from the PSI-Blast hit list was downloaded using the alignment retrieval option. This alignment was used to generate a profile using HMMER [79] defining the newly identified domain M60-like/PF13402. The following five steps were performed to generate our initial alignment: (1) The segment of the alignment corresponding to positions 193–378 (inclusive) of the *T. vaginalis* sequence XP_001313628 (the query sequence used for the aforementioned PSI-Blast) was identified as the most conserved across the aligned sequences by visual inspection and was retrieved using the masking option of SEAVIEW4.0 [80]. (2) Sequences with high level of identity (≥80%), considered as redundant, were removed leaving 92 sequences (the shortest entries across the aligned segment were removed). (3) These 92 sequences were re-aligned with MUSCLE using default settings within SEAVIEW4.0. (4) To minimise alignment length and optimise hypothesis of site homology indels larger than 2 residues (that complicate alignments) and present in less than 50% of the sequences were deleted. (5) Steps 3–4 were repeated and reduced the alignment to 208 aligned positions and 27 sequences (Figure S2). HMMER [79] was then used to generate and calibrate an HMM profile for the M60-like domain with the ‘hmmbulid’ commands with default settings. Following submission of the M60-like domain alignment to Pfam and Pfam curation, M60-like/PF13402 seed alignment and profile were generated and made available to us (Figure S3).

The HMM profile of the CMB32 domain (PF00754), CBM5_12 (PF02839), CBM51 (PF08305) and BACON (PF13004) were retrieved from the Pfam database.

### Detection of known protein structural features

SignalP 3.0 [81], TMHMM 2.0 [82] and PHOBIUS (that combines SP and TMD detections) [83], were employed to detection extracellular targeting N-terminal SP and TMD. Other characterised protein domains/motifs were searched using InterProScan version 4.3 [35]. The default parameters were used for every tool and where relevant the appropriate taxonomic option selected.

### Protein profile HMM searches

HMMER 3.0 was used to search M60-like/PF13402 HMM profile against proteins in RefSeq database (data obtained on 21^st^ January 2010, containing 9,662,677 protein sequences). The HMM profiles for specific domains (M60-like/PF13402, CBM32, CBM5_12, CBM51 and BACON) were used to search (i) the annotated peptidase library retrieved from the MEROPS database [39](release 9.1, data obtained 2^nd^ May 2010) and (ii) the 415 entries positive for the M60-like/PF13402 domain. The ‘hmmsearch’ command was used to search a given profile against the different protein sets.

### Protein profile-profile searches

To perform HMM-HMM profile comparison between the M60-like/PF13402 profile against other known profiles, the HHPred server running with HHSearch version 1.6.0.0 [84] was used to search all the available databases. HHPred was also used to identify divergent versions of PA14 and CBM32 domains from *T. vaginalis* M60-like/PF13402 containing proteins (Figure S4).

### Association of the M60-like/PF13402 domain to microbial habitat

To investigate the significance of the association between the presence of M60-like/PF13402 domain (genotype) and host associated or mucosal-related lifestyles (phenotype/trait) of microorganisms, we calculated the probability of the co-occurrence between the genotypic and phenotypic features according to hypergeometric distribution function [12]:
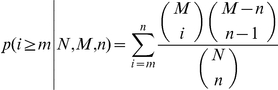
Of the total number of microorganisms with completed genome sequences in the RefSeq database at the time, 455 (N) have habitat information that can be used to determine whether an organism is able to thrive on or penetrate through vertebrate mucosa surfaces (Table S16). The number of these microorganism with an M60-like/PF13402 domain annotated was 55 (n). The number of microorganisms known to thrive on or infect host through mucosal surfaces was 197 (M). Of these 197 taxa, 43 (m) taxa possess at least one M60-like/PF13402 domain. As a result, the probability (p-value) of observing the association of the M60-like/PF13402 domain and the ability of microbe to thrive on mucosal surface can be calculated.

To determine the type of this association (either positive or negative), the mean value (μ) of hypergeometric distribution was used [12]:
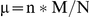
Where n, M, N and m can be referred from the previous equation, for m>μ corresponding to positive associations and for m<μ corresponding to negative associations.

### Protein family visualisation with CLANS

We used CLANS [85], a graph-based protein sequence similarity visualisation software, to investigate relationships between M60-like/PF13402 and M60-enhancins/PF03272 containing proteins. The software clusters set of protein sequences based on their BlastP p-values of the high-scoring segment pair alignments. All 693 sequences identified with HMMER searches using the M60-like/PF13402 and M60-enhancins/PF03272 profiles (all entries are listed in Table S5) with the default settings, were included into the CLANS analysis. Nodes or entries that have no similarity to other entries based on BlastP cutoff e-value 1.00E-5 were removed from the graph.

### Recombinant protein expression of BT4244 and in vitro mucinase assay

The gene encoding full length BT4244 protein lacking its N-terminal lipoprotein signal sequence was amplified from *B. thetaiotaomicron* VPI-5482 genomic DNA and cloned into pRSETA (Invitrogen) on BamHI/EcoRI generating the construct pRSETA-BT4244. Truncated constructs were generated that encoded either the C-terminal M60-like/PF13402 peptidase domain only or the N-terminal BACON and CBM32 domains only (Figure 4). Site-directed mutagenesis of the BT4244 catalytic glutamic residue (conservative mutation E575D, e.g. [51]) was carried out using the QuikChange protocol (Stratagene) according to the manufacturer's instructions with the construct pRSETA-BT4244 as template DNA. All constructs and the E575D mutant were confirmed by sequencing. The primers used for PCR amplifications and the mutagenesis are listed in Table S17. Recombinant proteins with an N-terminal His-tag were expressed in BL21 (Novagen) and purified in a single step by metal affinity chromatography using Talon resin (Clontech) as described previously [86]. Purified proteins were dialysed overnight against phosphate buffered saline pH 7.3 (OXOID, Dulbeco ‘A’ PBS) prior to the mucinase assay.

Mucins from bovine submaxillary glands Type I-S (Sigma, UK) were used as substrate for the mucinase assays in the absence or presence of 50 mM EDTA (e.g. [51]). Following incubations at 37°C for 48 hours the mucins were run on a 1% (w/v) agarose gel (SeaKem agarose, Melford ltd., UK)+0.1% (w/v) SDS in a Biorad minigel system and then transferred onto PVDF membranes by blotting. Biotin conjugated lectin from *Triticum vulgaris* (wheat germ agglutinin lyophilized powder, Sigma, UK) in combination with ExtrAvidin®–Peroxidase buffered aqueous solution (Sigma, UK) was used to detect mucin on blots.

### Phylogenetic analyses

A broad protein alignment of the M60-like/PF13402 domain was generated using the PF13402 seed alignment as reference using the “Profile alignment” function from CLUSTAL within SEAVIEW [80]. A subset of 57 sequences was eventually selected to reduce the complexity of the dataset and optimising sequence and taxonomic diversity. Maximum likelihood trees were computed with PhyML from within SEAVIEW with the LG model and a gamma shape parameter to correct for site rate heterogeneity (4 discrete rates) and using both NNI and SPR for the tree search operations. 100 bootstrap pseudo replicates were generated to calculate branch support values. The tool iTOL [87] was used to map on the inferred phylogenetic tree the structural organisation of the analysed proteins. An additional phylogenetic analysis was performed including selected enhancin proteins sequences (M60-enhancin/PF03272 domain) (Figure S5, Figure S7)

## Supporting Information

Figure S1 **PSI-Blast output figure obtained from the NCBI Blast server.** The query sequence was from *Trichomonas vaginalis* (GI:123449825, XP_001313628, the complete sequence) and two iterations (e-value ≤1.00E-05). File format: tif.
(TIF)

Figure S2 **Initial protein alignment corresponding to the new M60-like domain, 27 sequences with 206 aligned sites.** The specie names are abbreviated with the first three letters of the genus and species names along the corresponding protein sequence GenBank GI number. The query sequence used for the PSI-Blast is shown on the top with the RefSeq accession number. Format: text file with sequences in fasta format to be open in a sequence alignment editor such as SEAVIEW.
(FAS)

Figure S3 **Pfam seed alignment for the new M60-like domain, accession PF13402, consisting of 68 sequences with 387 aligned sites.** The UniProt accession number for each sequence is provided along with the position of the start and end of the aligned segments. Format: text file with sequences in fasta format to be open in a sequence alignment editor such as SEAVIEW.
(FAS)

Figure S4 **Figure illustrating the PA14-like and Galactose binding domain-like (GBD) domain identified in** ***Trichomonas vaginalis*** **M60-like/PF13402 containing proteins.**
(TIF)

Figure S5 **M60-like/PF13402 alignment of 57 PF13402 and 4 PF03272 sequences and 175 sites used to infer the phylogeny shown in** **Figure 6** **(57 PF13402 sequences only) or Figure S7 (all 61 sequences).** Format: text file with sequences in fasta format to be open in a sequence alignment editor such as SEAVIEW.
(FAS)

Figure S6 **Two-dimensional graph layout from the CLANS clustering results obtained from the full-length sequences for the M60-enhancin/PF03272 or M60-like/PF13402 containing proteins.** Each protein sequence is shown by a black dot. Lines connecting dots indicate sequence similarity generated from BlastP: red lines edges represent sequence similarity with Blast e-value <1E-100, whereas grey lines represent Blast e-value from 1E-5 to 1E-100. Entries that have more significant hits with the M60-enhancin/PF03272 profile are encircled in green. All entries outside the green circle have more significant hit on the M60-like/PF13402 profile. Selected clusters are labeled with their taxonomic composition. Notably the two families of Bacillus entries are clustering at the vicinity of each other. Format: tif.
(TIF)

Figure S7 **Protein maximum likelihood bootstrap consensus tree for selected M60-like/PF13402 and M60-enhancin/PF03272 domains.** The shown maximum likelihood tree (Log likelihood: −19083.4) was generated as described in the Methods section using an alignment of 57 sequences and 175 residues drawn from an M60-like/PF13402 (see Figure 6) domain alignment complemented with four M60-enhancin/PF03272 sequences (boxed), providing an the evolutionary framework for the gene segments encoding these domains. For each sequence the corresponding abbreviated species name is indicated along with the NCBI GI number. Format: tif.
(TIF)

Table S1 **PSI-Blast Taxonomic report derived from the NCBI Blast server.** The query sequence was from *Trichomonas vaginalis* (GI:123449825, XP_001313628, residues 1–500), 2 iterations (e-value ≤1.00E-04). After seven iterations no new sequences were recovered and these additional entries were all recovered by the HMMER searches using PF13402, PF03272 and PTHR15730 profiles, see Table S5 for the complete list of entries. Format: html file to be opened with a web browser providing links to original RefSeq entries at the NCBI.
(HTML)

Table S2 **Table listing all 523 RefSeq protein entries positive for the PF13402 profile (HMMER search, e-value ≤1.00E-05) contrasting the PSI-Blast values with HMMER scores for the PF13402 and PF03272 profiles.** The sequences for the gluzincin and zincin motif are also shown. Format: Excel file with five worksheets.
(XLSX)

Table S3 **Table listing all 38 MEROPS entries positive for the PF13402 profile (e-value ≤1.00E-05).**
(XLSX)

Table S4 **HHPred search hit list performed with the PF13402 seed alignment (Figure S3) using the global alignment mode and against all available databases.** Format: html file to be opened with a web browser.
(HTML)

Table S5 **Table listing: (i) all sequence entries identified using HMMER searches with the M60-enhancin/PF03272, M60-like/PF13402 or PTHR15730 profiles using default settings.** (ii) The 415 proteins most significant for the PF13402 profile (HMMER search) with taxa names encoding them, sequence and position of the gluzincin and zincin motifs (when present) and position of the M60-like/PF13402 domain. Output for analyses by PHOBIUS, LIPOP and TMHMM are also listed (iii) taxa counts for the 415 entries most significant for the PF13402 profile. Excel file with three worksheets.
(XLSX)

Table S6 **Table listing all the bacterial taxa encoding proteins with the M60-like/PF13402 domain without evidence for being associated with animal hosts.**
(XLSX)

Table S7 **Table listing all InterProScan hits for the 415 M60-like/PF13402 positive proteins (non-redundant list as InterProScan considers only one protein among identical entries).**
(XLSX)

Table S8 **Table listing M60-like/PF13402-CBM32 entries.** Separate worksheets list sequence counts or taxa counts along with habitat information for each species/strains. Format: Excel file with two worksheets.
(XLSX)

Table S9 **Table listing M60-like/PF13402-CBM5_12 entries.** Separate worksheets list sequence counts or taxa counts along with habitat information for each species/strains. Format: Excel file with two worksheets.
(XLSX)

Table S10 **Table listing M60-like/PF13402-CBM51 entries.** Separate worksheets list sequence counts, taxa counts along with habitat information for each species/strains. Entries dually positive for CBM51 and CBM32 are also listed. Format: Excel file with two worksheets.
(XLSX)

Table S11 **Table listing the five** ***Bacillus*** **spp./strains encoding proteins with M60-like/PF13402-CBM32 and M60-like/PF13402-CBM5_12 domain combinations.**
(XLSX)

Table S12 **Table listing the 22 M60-like/PF13402-BACON entries.**
(XLSX)

Table S13 **Table listing the 176 proteins that have most significant hits with the M60-enhancin/PF03272 profile.** The Pfam domain composition and the taxonomic origin of each sequence are also indicated. Format: Excel file with three worksheets.
(XLSX)

Table S14 **PHOBIUS analysis for 141 MEROPS entries possessing CBM32 or CBM5_12.**
(XLSX)

Table S15 **Table listing species and strains of selected bacterial genus that were present in the RefSeq database at the time of our analyses and the corresponding number of M60-like/PF13402 positive proteins:** ***Bacillus*** **,** ***Bacteroides*** **,** ***Clostridium*** **,** ***Escherichia*** **,** ***Vibrio*** **and** ***Yersinia*** **.** Each genus is listed in a separate worksheet. Format: Excel file with six worksheets.
(XLSX)

Table S16 **Table listing all taxa and their habitat (when known) considered for the PF13402 domain-habitat association analysis using a hypergeometric test.**
(XLSX)

Table S17 **Table listing all primers used to PCR clone the three different segments of the** ***BT4244*** **gene for their expression as recombinant proteins in** ***E. coli*** **.** Primers used to generate the E575D mutant are also listed. Format: Excel file.
(XLSX)
