# Supplementary figures and images for: A Novel Extracellular Metallopeptidase Domain Shared by Animal Host-Associated Mutualistic and Pathogenic Microbes
Source: PLoS One. 2012 Jan 27;7(1):e30287. doi: 10.1371/journal.pone.0030287 (PMC3267712; doi:10.1371/journal.pone.0030287)

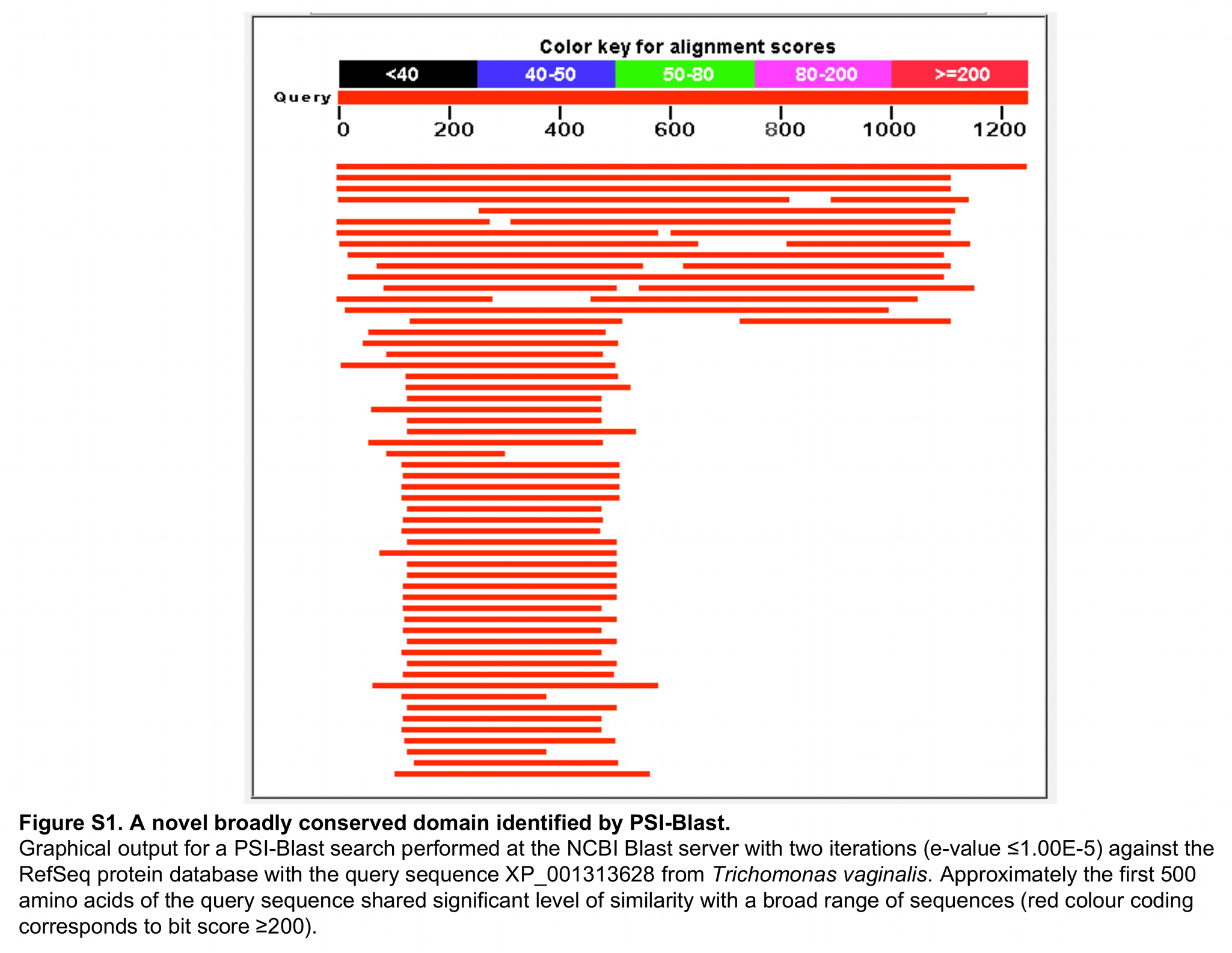

Supplement: Figure S1 — PSI-Blast output figure obtained from the NCBI Blast server. The query sequence was from Trichomonas vaginalis (GI:123449825, XP_001313628, the complete sequence) and two iterations (e-value ≤1.00E-05). File format: tif. (TIF) [file pone.0030287.s001.tif]

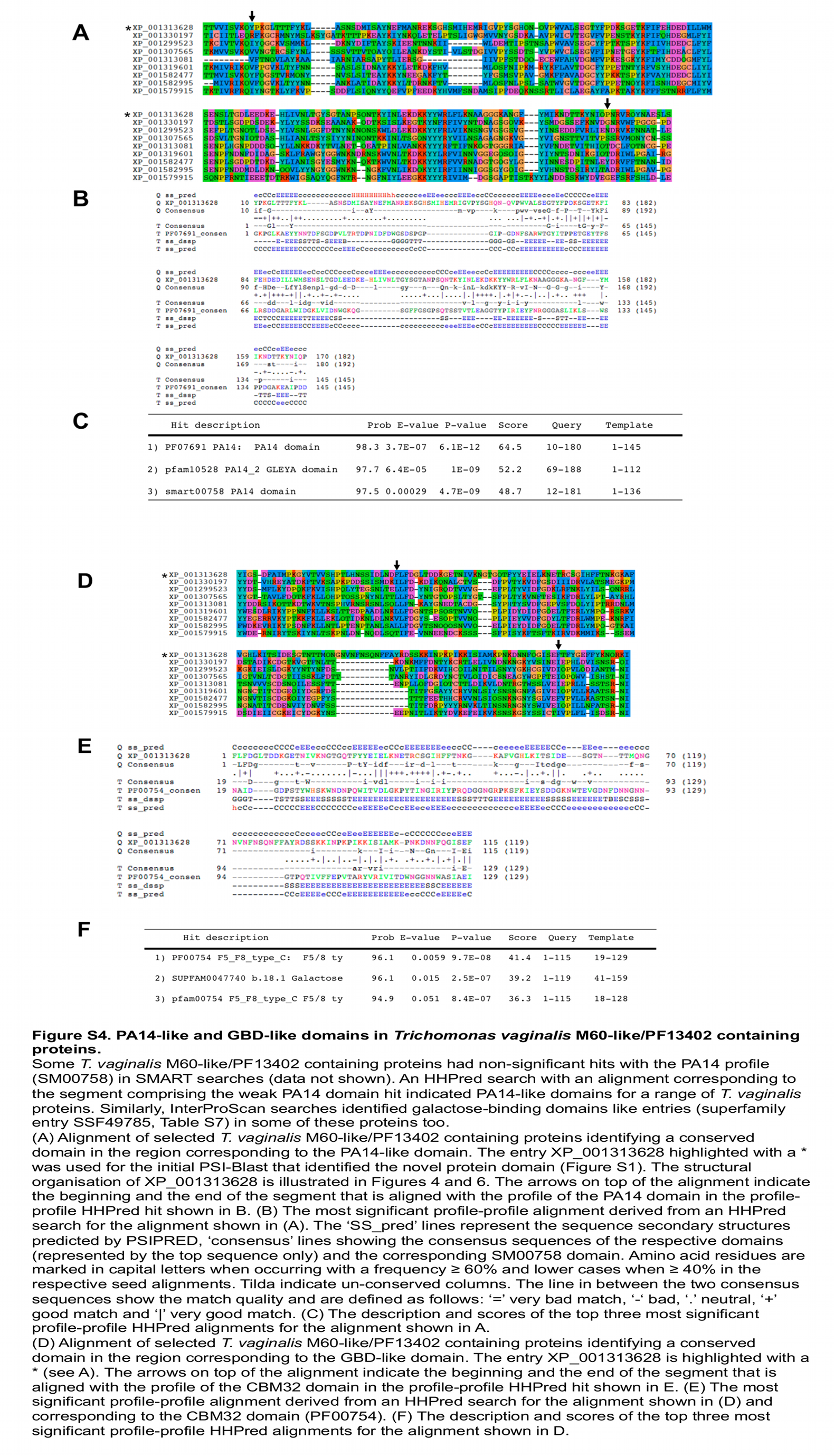

Supplement: Figure S4 — Figure illustrating the PA14-like and Galactose binding domain-like (GBD) domain identified in Trichomonas vaginalis M60-like/PF13402 containing proteins. (TIF) [file pone.0030287.s004.tif]

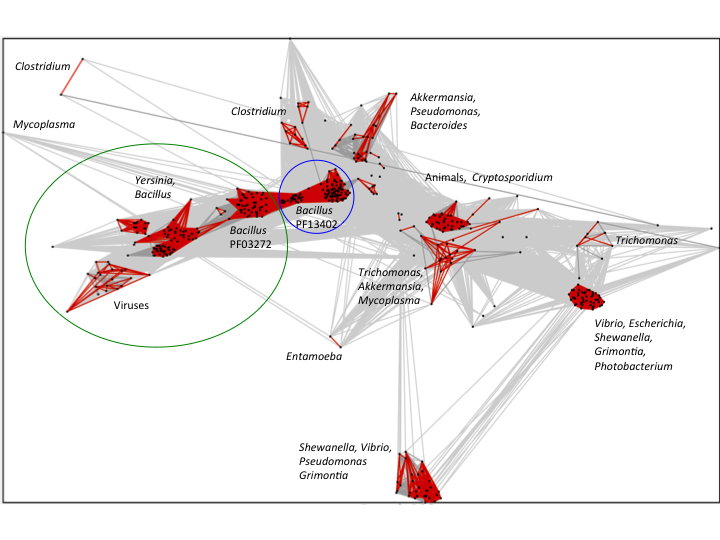

Supplement: Figure S6 — Two-dimensional graph layout from the CLANS clustering results obtained from the full-length sequences for the M60-enhancin/PF03272 or M60-like/PF13402 containing proteins. Each protein sequence is shown by a black dot. Lines connecting dots indicate sequence similarity generated from BlastP: red lines edges represent sequence similarity with Blast e-value <1E-100, whereas grey lines represent Blast e-value from 1E-5 to 1E-100. Entries that have more significant hits with the M60-enhancin/PF03272 profile are encircled in green. All entries outside the green circle have more significant hit on the M60-like/PF13402 profile. Selected clusters are labeled with their taxonomic composition. Notably the two families of Bacillus entries are clustering at the vicinity of each other. Format: tif. (TIF) [file pone.0030287.s006.tif]

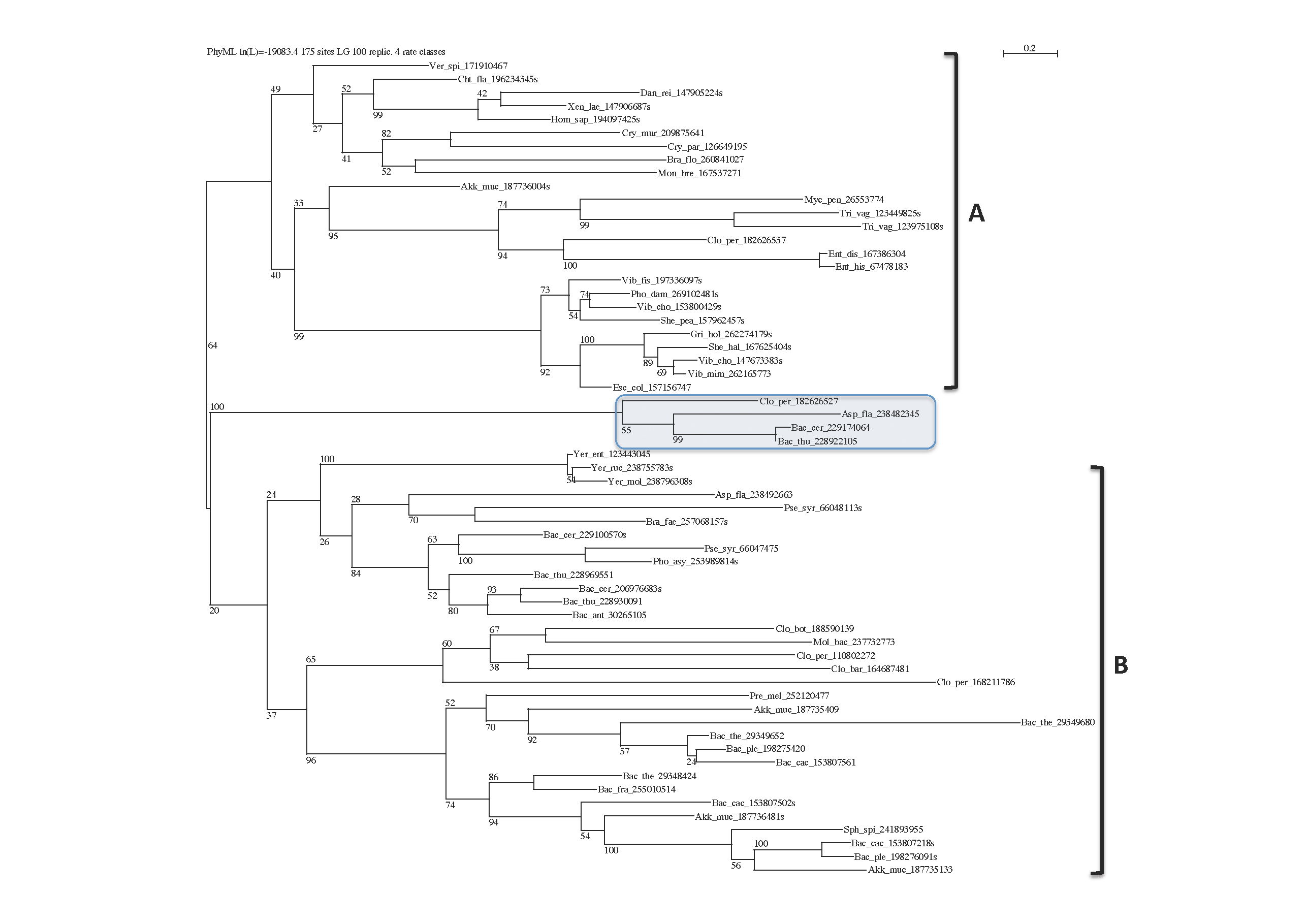

Supplement: Figure S7 — Protein maximum likelihood bootstrap consensus tree for selected M60-like/PF13402 and M60-enhancin/PF03272 domains. The shown maximum likelihood tree (Log likelihood: −19083.4) was generated as described in the Methods section using an alignment of 57 sequences and 175 residues drawn from an M60-like/PF13402 (see Figure 6) domain alignment complemented with four M60-enhancin/PF03272 sequences (boxed), providing an the evolutionary framework for the gene segments encoding these domains. For each sequence the corresponding abbreviated species name is indicated along with the NCBI GI number. Format: tif. (TIF) [file pone.0030287.s007.tif]
