# Supplementary material for: A Novel Extracellular Metallopeptidase Domain Shared by Animal Host-Associated Mutualistic and Pathogenic Microbes
Source: PLoS One. 2012 Jan 27;7(1):e30287. doi: 10.1371/journal.pone.0030287 (PMC3267712; doi:10.1371/journal.pone.0030287)
Supplement: Table S1 — PSI-Blast Taxonomic report derived from the NCBI Blast server. The query sequence was from Trichomonas vaginalis (GI:123449825, XP_001313628, residues 1–500), 2 iterations (e-value ≤1.00E-04). After seven iterations no new sequences were recovered and these additional entries were all recovered by the HMMER searches using PF13402, PF03272 and PTHR15730 profiles, see Table S5 for the complete list of entries. Format: html file to be opened with a web browser providing links to original RefSeq entries at the NCBI. (HTML) [file pone.0030287.s008.html]

### Table S1. Taxomic report for a PSI-BLAST search on the RefSeq database identifying a new domain

---


### Profile (PSSM): Query T. vaginalis protein XP\_001313628, positions 1-500

---

**Index**

- Lineage Report
- Organism Report
- Taxonomy Report
- Help

**Lineage Report**  

```
root
. cellular organisms
. . Eukaryota          [eukaryotes]
. . . Trichomonas vaginalis G3 -----------------------------------------------  497 26 hits [trichomonads]         Immuno-dominant variable surface antigen-like [Trichomonas 
. . . Danio rerio (zebra fish) ...............................................  349  4 hits [bony fishes]          PREDICTED: hypothetical protein [Danio rerio]
. . . Bos taurus (cow) .......................................................  349  2 hits [even-toed ungulates]  hypothetical protein LOC533126 [Bos taurus]
. . . Equus caballus (equine) ................................................  347  2 hits [odd-toed ungulates]   PREDICTED: hypothetical protein [Equus caballus]
. . . Canis lupus familiaris (dogs) ..........................................  347  3 hits [carnivores]           PREDICTED: hypothetical protein XP_539846 isoform 1 [Canis 
. . . Pan troglodytes ........................................................  346  2 hits [primates]             PREDICTED: similar to KIAA0738 protein [Pan troglodytes]
. . . Homo sapiens (man) .....................................................  346  5 hits [primates]             hypothetical protein LOC9747 [Homo sapiens]
. . . Macaca mulatta (rhesus macaque) ........................................  343  2 hits [primates]             PREDICTED: hypothetical protein [Macaca mulatta]
. . . Mus musculus (mouse) ...................................................  340  3 hits [rodents]              hypothetical protein LOC77574 [Mus musculus]
. . . Rattus norvegicus (brown rat) ..........................................  339  5 hits [rodents]              PREDICTED: hypothetical protein [Rattus norvegicus]
. . . Taeniopygia guttata (zebra finch) ......................................  333  2 hits [birds]                PREDICTED: similar to Protein FAM115C [Taeniopygia guttata]
. . . Ornithorhynchus anatinus (duck-billed platypus) ........................  327  3 hits [monotremes]           PREDICTED: similar to seven transmembrane helix receptor [O
. . . Xenopus laevis (common platanna) .......................................  323  1 hit  [frogs & toads]        hypothetical protein LOC494989 [Xenopus laevis]
. . . Branchiostoma floridae .................................................  290  2 hits [lancelets]            hypothetical protein BRAFLDRAFT_85351 [Branchiostoma florid
. . . Pongo abelii (Orang-utan) ..............................................  276  1 hit  [primates]             hypothetical protein LOC100173022 [Pongo abelii]
. . . Entamoeba dispar SAW760 ................................................  266  1 hit  [eukaryotes]           antigenic protein NP1 [Entamoeba dispar SAW760]
. . . Entamoeba histolytica HM-1:IMSS ........................................  266  1 hit  [eukaryotes]           immuno-dominant variable surface antigen [Entamoeba histoly
. . . Aspergillus flavus NRRL3357 ............................................  231  2 hits [ascomycetes]          cell wall associated protein, putative [Aspergillus flavus 
. . . Aspergillus oryzae RIB40 ...............................................  227  2 hits [ascomycetes]          hypothetical protein [Aspergillus oryzae RIB40]
. . . Cryptosporidium muris RN66 .............................................  207  1 hit  [apicomplexans]        hypothetical protein [Cryptosporidium muris RN66]
. . . Monosiga brevicollis MX1 ...............................................  161  1 hit  [choanoflagellates]    hypothetical protein [Monosiga brevicollis MX1]
. . . Cryptosporidium parvum Iowa II .........................................  143  1 hit  [apicomplexans]        hypothetical protein [Cryptosporidium parvum Iowa II]
. . . Uncinocarpus reesii 1704 ...............................................  139  1 hit  [ascomycetes]          hypothetical protein UREG_04003 [Uncinocarpus reesii 1704]
. . . Cryptosporidium hominis TU502 ..........................................   58  1 hit  [apicomplexans]        hypothetical protein [Cryptosporidium hominis TU502]
. . Chthoniobacter flavus Ellin428 -------------------------------------------  356  1 hit  [verrucomicrobia]      conserved hypothetical protein [Chthoniobacter flavus Ellin
. . Bacillus cereus ATCC 10987 ...............................................  350  1 hit  [firmicutes]           hypothetical protein BCE_5203 [Bacillus cereus ATCC 10987]
. . Bacillus thuringiensis serovar pakistani str. T13001 .....................  349  2 hits [firmicutes]           hypothetical protein bthur0005_47520 [Bacillus thuringiensi
. . Geobacillus sp. Y412MC10 .................................................  349  2 hits [firmicutes]           hypothetical protein GYMC10_4678 [Geobacillus sp. Y412MC10]
. . Bacillus cereus m1293 ....................................................  347  1 hit  [firmicutes]           hypothetical protein bcere0001_47200 [Bacillus cereus m1293]
. . Bacillus thuringiensis serovar sotto str. T04001 .........................  347  2 hits [firmicutes]           hypothetical protein bthur0004_49610 [Bacillus thuringiensi
. . Bacillus thuringiensis serovar konkukian str. 97-27 ......................  347  2 hits [firmicutes]           wall-associated protein [Bacillus thuringiensis serovar kon
. . Bacillus weihenstephanensis KBAB4 ........................................  346  3 hits [firmicutes]           wall-associated protein precursor [Bacillus weihenstephanen
. . Bacillus cereus AH820 ....................................................  346  3 hits [firmicutes]           wall-associated protein [Bacillus cereus AH820]
. . Bacillus cereus Rock3-42 .................................................  345  2 hits [firmicutes]           hypothetical protein bcere0021_48880 [Bacillus cereus Rock3
. . Bacillus thuringiensis serovar andalousiensis BGSC 4AW1 ..................  345  2 hits [firmicutes]           hypothetical protein bthur0009_47800 [Bacillus thuringiensi
. . Bacillus cereus 95/8201 ..................................................  345  3 hits [firmicutes]           hypothetical protein bcere0016_48840 [Bacillus cereus 95/82
. . Bacillus thuringiensis serovar monterrey BGSC 4AJ1 .......................  345  4 hits [firmicutes]           hypothetical protein bthur0007_49140 [Bacillus thuringiensi
. . Bacillus thuringiensis serovar pondicheriensis BGSC 4BA1 .................  345  3 hits [firmicutes]           hypothetical protein bthur0010_47720 [Bacillus thuringiensi
. . Bacillus thuringiensis IBL 200 ...........................................  345  2 hits [firmicutes]           hypothetical protein bthur0013_50430 [Bacillus thuringiensi
. . Bacillus thuringiensis serovar israelensis ATCC 35646 ....................  345  3 hits [firmicutes]           Wall-associated protein precursor [Bacillus thuringiensis s
. . Bacillus thuringiensis IBL 4222 ..........................................  344  2 hits [firmicutes]           hypothetical protein bthur0014_47230 [Bacillus thuringiensi
. . Bacillus cereus G9842 ....................................................  344  2 hits [firmicutes]           wall-associated protein [Bacillus cereus G9842]
. . Bacillus cereus W ........................................................  343  3 hits [firmicutes]           wall-associated protein [Bacillus cereus W]
. . Bacillus cereus AH1271 ...................................................  342  1 hit  [firmicutes]           hypothetical protein bcere0028_47380 [Bacillus cereus AH127
. . Bacillus cereus AH603 ....................................................  342  3 hits [firmicutes]           hypothetical protein bcere0026_47920 [Bacillus cereus AH603]
. . Bacillus cereus AH621 ....................................................  342  3 hits [firmicutes]           hypothetical protein bcere0007_47240 [Bacillus cereus AH621]
. . Bacillus cereus H3081.97 .................................................  342  1 hit  [firmicutes]           wall-associated protein [Bacillus cereus H3081.97]
. . Bacillus thuringiensis serovar berliner ATCC 10792 .......................  341  5 hits [firmicutes]           hypothetical protein bthur0008_48610 [Bacillus thuringiensi
. . Bacillus thuringiensis serovar thuringiensis str. T01001 .................  341  5 hits [firmicutes]           hypothetical protein bthur0008_48610 [Bacillus thuringiensi
. . Bacillus thuringiensis Bt407 .............................................  341  4 hits [firmicutes]           hypothetical protein bthur0008_48610 [Bacillus thuringiensi
. . Bacillus cereus AH187 ....................................................  341  1 hit  [firmicutes]           wall-associated protein [Bacillus cereus AH187] >gi|2220985
. . Bacillus cereus Q1 .......................................................  341  1 hit  [firmicutes]           wall-associated protein [Bacillus cereus AH187] >gi|2220985
. . Bacillus cereus Rock4-2 ..................................................  341  3 hits [firmicutes]           hypothetical protein bcere0023_49240 [Bacillus cereus Rock4
. . Bacillus cereus BDRD-ST26 ................................................  340  1 hit  [firmicutes]           hypothetical protein bcere0013_48840 [Bacillus cereus BDRD-
. . Bacillus thuringiensis str. Al Hakam .....................................  340  1 hit  [firmicutes]           wall-associated protein [Bacillus thuringiensis str. Al Hak
. . Bacillus cereus 03BB108 ..................................................  340  1 hit  [firmicutes]           wall-associated protein [Bacillus cereus 03BB108] >gi|22586
. . Bacillus cereus 03BB102 ..................................................  340  1 hit  [firmicutes]           wall-associated protein [Bacillus cereus 03BB108] >gi|22586
. . Bacillus cereus B4264 ....................................................  340  3 hits [firmicutes]           wall-associated protein [Bacillus cereus B4264]
. . Bacillus thuringiensis serovar huazhongensis BGSC 4BD1 ...................  340  3 hits [firmicutes]           hypothetical protein bthur0011_47670 [Bacillus thuringiensi
. . Bacillus cereus BGSC 6E1 .................................................  340  1 hit  [firmicutes]           hypothetical protein bcere0004_48420 [Bacillus cereus BGSC 
. . Bacillus cereus 172560W ..................................................  339  3 hits [firmicutes]           hypothetical protein bcere0005_46970 [Bacillus cereus 17256
. . Bacillus thuringiensis serovar kurstaki str. T03a001 .....................  338  3 hits [firmicutes]           hypothetical protein bthur0006_46840 [Bacillus thuringiensi
. . Bacillus cereus F65185 ...................................................  337  3 hits [firmicutes]           hypothetical protein bcere0025_47080 [Bacillus cereus F6518
. . Bacillus cereus BDRD-ST24 ................................................  337  2 hits [firmicutes]           hypothetical protein bcere0012_47390 [Bacillus cereus BDRD-
. . Bacillus cereus Rock1-15 .................................................  337  3 hits [firmicutes]           hypothetical protein bcere0018_47450 [Bacillus cereus Rock1
. . Bacillus cereus BDRD-Cer4 ................................................  337  2 hits [firmicutes]           hypothetical protein bcere0015_47530 [Bacillus cereus BDRD-
. . Bacillus cereus AH1134 ...................................................  336  4 hits [firmicutes]           wall-associated protein [Bacillus cereus AH1134]
. . Bacillus cereus AH676 ....................................................  335  3 hits [firmicutes]           hypothetical protein bcere0027_47080 [Bacillus cereus AH676]
. . Bacillus cereus MM3 ......................................................  334  3 hits [firmicutes]           hypothetical protein bcere0006_48340 [Bacillus cereus MM3]
. . Bacillus cereus NVH0597-99 ...............................................  334  2 hits [firmicutes]           wall-associated protein [Bacillus cereus NVH0597-99]
. . Bacillus cereus ATCC 10876 ...............................................  332  4 hits [firmicutes]           hypothetical protein bcere0002_49870 [Bacillus cereus ATCC 
. . Bacillus thuringiensis serovar tochigiensis BGSC 4Y1 .....................  329  2 hits [firmicutes]           hypothetical protein bthur0001_49700 [Bacillus thuringiensi
. . Planctomyces limnophilus DSM 3776 ........................................  327  1 hit  [planctomycetes]       hypothetical protein PlimDRAFT_30570 [Planctomyces limnophi
. . Bacillus cereus BDRD-ST196 ...............................................  326  3 hits [firmicutes]           hypothetical protein bcere0014_47770 [Bacillus cereus BDRD-
. . Bacillus cereus G9241 ....................................................  325  1 hit  [firmicutes]           reticulocyte binding protein [Bacillus cereus G9241]
. . Bacillus thuringiensis serovar pulsiensis BGSC 4CC1 ......................  325  2 hits [firmicutes]           hypothetical protein bthur0012_49000 [Bacillus thuringiensi
. . Verrucomicrobium spinosum DSM 4136 .......................................  324  1 hit  [verrucomicrobia]      hypothetical protein VspiD_04825 [Verrucomicrobium spinosum
. . Bacillus cereus ATCC 14579 ...............................................  321  2 hits [firmicutes]           wall-associated protein precursor [Bacillus cereus ATCC 145
. . Bacillus anthracis str. A1055 ............................................  321  2 hits [firmicutes]           wall-associated protein [Bacillus anthracis str. A1055]
. . Bacillus cereus E33L .....................................................  320  3 hits [firmicutes]           wall-associated protein [Bacillus cereus E33L]
. . Bacillus cereus ATCC 4342 ................................................  320  1 hit  [firmicutes]           hypothetical protein bcere0010_48380 [Bacillus cereus ATCC 
. . Bacillus anthracis str. Ames .............................................  319  2 hits [firmicutes]           hypothetical protein BA_5305 [Bacillus anthracis str. Ames]
. . Bacillus anthracis str. 'Ames Ancestor' ..................................  319  2 hits [firmicutes]           hypothetical protein BA_5305 [Bacillus anthracis str. Ames]
. . Bacillus anthracis str. Sterne ...........................................  319  2 hits [firmicutes]           hypothetical protein BA_5305 [Bacillus anthracis str. Ames]
. . Bacillus anthracis str. CDC 684 ..........................................  319  2 hits [firmicutes]           hypothetical protein BA_5305 [Bacillus anthracis str. Ames]
. . Bacillus anthracis str. CNEVA-9066 .......................................  319  2 hits [firmicutes]           hypothetical protein BA_5305 [Bacillus anthracis str. Ames]
. . Bacillus anthracis str. Western North America USA6153 ....................  319  2 hits [firmicutes]           hypothetical protein BA_5305 [Bacillus anthracis str. Ames]
. . Bacillus anthracis str. Kruger B .........................................  319  2 hits [firmicutes]           hypothetical protein BA_5305 [Bacillus anthracis str. Ames]
. . Bacillus anthracis str. Vollum ...........................................  319  2 hits [firmicutes]           hypothetical protein BA_5305 [Bacillus anthracis str. Ames]
. . Bacillus anthracis str. Australia 94 .....................................  319  2 hits [firmicutes]           hypothetical protein BA_5305 [Bacillus anthracis str. Ames]
. . Bacillus anthracis str. A2012 ............................................  318  2 hits [firmicutes]           hypothetical protein Bant_01000177 [Bacillus anthracis str.
. . Bacillus cereus Rock1-3 ..................................................  315  5 hits [firmicutes]           S-layer domain protein [Bacillus cereus Rock1-3]
. . Bacillus cytotoxicus NVH 391-98 ..........................................  314  1 hit  [firmicutes]           S-layer domain-containing protein [Bacillus cereus subsp. c
. . Bacillus cereus R309803 ..................................................  313  1 hit  [firmicutes]           hypothetical protein bcere0009_47940 [Bacillus cereus R3098
. . Bacillus cereus Rock3-29 .................................................  313  4 hits [firmicutes]           S-layer domain protein [Bacillus cereus Rock3-29]
. . Bacillus mycoides DSM 2048 ...............................................  312  2 hits [firmicutes]           S-layer domain protein [Bacillus mycoides DSM 2048]
. . Bacillus pseudomycoides DSM 12442 ........................................  306  2 hits [firmicutes]           S-layer domain protein [Bacillus pseudomycoides DSM 12442]
. . Bacillus cereus Rock3-28 .................................................  305  4 hits [firmicutes]           S-layer domain protein [Bacillus cereus Rock3-28]
. . Akkermansia muciniphila ATCC BAA-835 .....................................  304  4 hits [verrucomicrobia]      hypothetical protein Amuc_1514 [Akkermansia muciniphila ATC
. . Bacillus cereus m1550 ....................................................  293  2 hits [firmicutes]           Fibronectin type III domain protein [Bacillus cereus m1550]
. . Bacillus mycoides Rock3-17 ...............................................  290  1 hit  [firmicutes]           Fibronectin type III domain protein [Bacillus mycoides Rock
. . Chitinophaga pinensis DSM 2588 ...........................................  285  1 hit  [CFB group bacteria]   hypothetical protein Cpin_2532 [Chitinophaga pinensis DSM 2
. . Paenibacillus larvae subsp. larvae BRL-230010 ............................  283  3 hits [firmicutes]           S-layer domain protein [Paenibacillus larvae subsp. larvae 
. . Yersinia ruckeri ATCC 29473 ..............................................  275  1 hit  [enterobacteria]       hypothetical protein yruck0001_340 [Yersinia ruckeri ATCC 2
. . Escherichia coli B7A .....................................................  274  1 hit  [enterobacteria]       conserved hypothetical protein [Escherichia coli B7A]
. . Escherichia coli O111:H- str. 11128 ......................................  273  1 hit  [enterobacteria]       putative lipoprotein AcfD homolog precursor [Escherichia co
. . Escherichia sp. 1_1_43 ...................................................  273  1 hit  [enterobacteria]       conserved hypothetical protein [Escherichia sp. 1_1_43]
. . Yersinia enterocolitica subsp. enterocolitica 8081 .......................  272  1 hit  [enterobacteria]       hypothetical protein YE2830 [Yersinia enterocolitica subsp.
. . Escherichia coli str. K-12 substr. MG1655 ................................  272  1 hit  [enterobacteria]       predicted inner membrane lipoprotein [Escherichia coli str.
. . Escherichia coli str. K-12 substr. W3110 .................................  272  1 hit  [enterobacteria]       predicted inner membrane lipoprotein [Escherichia coli str.
. . Escherichia coli BW2952 ..................................................  272  1 hit  [enterobacteria]       predicted inner membrane lipoprotein [Escherichia coli str.
. . Escherichia coli BL21(DE3) ...............................................  272  1 hit  [enterobacteria]       predicted inner membrane lipoprotein [Escherichia coli str.
. . Escherichia coli 53638 ...................................................  271  1 hit  [enterobacteria]       conserved hypothetical protein [Escherichia coli 53638]
. . Escherichia coli 101-1 ...................................................  271  1 hit  [enterobacteria]       conserved hypothetical protein [Escherichia coli 101-1]
. . Escherichia coli IAI39 ...................................................  271  1 hit  [enterobacteria]       inner membrane lipoprotein [Escherichia coli IAI39]
. . Escherichia coli SE11 ....................................................  270  1 hit  [enterobacteria]       hypothetical protein ECSE_3249 [Escherichia coli SE11]
. . Escherichia coli 83972 ...................................................  270  1 hit  [enterobacteria]       lipoprotein AcfD precursor [Escherichia coli 83972]
. . Yersinia aldovae ATCC 35236 ..............................................  270  1 hit  [enterobacteria]       hypothetical protein yaldo0001_11520 [Yersinia aldovae ATCC
. . Escherichia coli UMN026 ..................................................  270  1 hit  [enterobacteria]       inner membrane lipoprotein [Escherichia coli UMN026]
. . Escherichia coli E22 .....................................................  270  1 hit  [enterobacteria]       conserved hypothetical protein [Escherichia coli E22]
. . Shigella sp. D9 ..........................................................  269  1 hit  [enterobacteria]       hypothetical protein ShiD9_09354 [Shigella sp. D9]
. . Escherichia coli B str. REL606 ...........................................  269  1 hit  [enterobacteria]       predicted inner membrane lipoprotein [Escherichia coli B st
. . Escherichia coli E110019 .................................................  269  1 hit  [enterobacteria]       hypothetical protein EcE110019_1447 [Escherichia coli E1100
. . Escherichia coli F11 .....................................................  268  1 hit  [enterobacteria]       conserved hypothetical protein [Escherichia coli F11]
. . Escherichia sp. 4_1_40B ..................................................  267  1 hit  [enterobacteria]       inner membrane lipoprotein [Escherichia sp. 4_1_40B]
. . Escherichia coli SMS-3-5 .................................................  267  1 hit  [enterobacteria]       hypothetical protein EcSMS35_3251 [Escherichia coli SMS-3-5]
. . Escherichia coli HS ......................................................  267  1 hit  [enterobacteria]       hypothetical protein EcHS_A3142 [Escherichia coli HS]
. . Escherichia coli 536 .....................................................  266  1 hit  [enterobacteria]       putative lipoprotein AcfD precursor [Escherichia coli 536]
. . Escherichia coli E24377A .................................................  265  1 hit  [enterobacteria]       hypothetical protein EcE24377A_3432 [Escherichia coli E2437
. . Escherichia coli O127:H6 str. E2348/69 ...................................  264  1 hit  [enterobacteria]       predicted inner membrane lipoprotein [Escherichia coli O127
. . Yersinia mollaretii ATCC 43969 ...........................................  264  1 hit  [enterobacteria]       hypothetical protein ymoll0001_21340 [Yersinia mollaretii A
. . Escherichia coli 55989 ...................................................  263  1 hit  [enterobacteria]       inner membrane lipoprotein [Escherichia coli 55989]
. . Escherichia coli S88 .....................................................  263  1 hit  [enterobacteria]       inner membrane lipoprotein [Escherichia coli S88]
. . Escherichia coli UTI89 ...................................................  262  1 hit  [enterobacteria]       putative lipoprotein AcfD-like precursor [Escherichia coli 
. . Escherichia coli APEC O1 .................................................  262  1 hit  [enterobacteria]       putative lipoprotein AcfD-like precursor [Escherichia coli 
. . Escherichia sp. 3_2_53FAA ................................................  262  1 hit  [enterobacteria]       putative lipoprotein AcfD-like precursor [Escherichia coli 
. . Escherichia coli ED1a ....................................................  261  1 hit  [enterobacteria]       inner membrane lipoprotein [Escherichia coli ED1a]
. . Escherichia albertii TW07627 .............................................  261  1 hit  [enterobacteria]       AcfD [Escherichia albertii TW07627]
. . Escherichia coli IAI1 ....................................................  261  1 hit  [enterobacteria]       inner membrane lipoprotein [Escherichia coli IAI1]
. . Escherichia fergusonii ATCC 35469 ........................................  261  1 hit  [enterobacteria]       inner membrane lipoprotein [Escherichia fergusonii ATCC 354
. . Bacteroides thetaiotaomicron VPI-5482 ....................................  250  4 hits [CFB group bacteria]   hypothetical protein BT_4244 [Bacteroides thetaiotaomicron 
. . Bacteroides sp. 1_1_6 ....................................................  250  4 hits [CFB group bacteria]   hypothetical protein BT_4244 [Bacteroides thetaiotaomicron 
. . Bacteroides sp. 3_2_5 ....................................................  249  1 hit  [CFB group bacteria]   conserved hypothetical protein [Bacteroides sp. 3_2_5]
. . Bacteroides caccae ATCC 43185 ............................................  249 16 hits [CFB group bacteria]   hypothetical protein BACCAC_01368 [Bacteroides caccae ATCC 
. . Bacteroides fragilis YCH46 ...............................................  248  1 hit  [CFB group bacteria]   hypothetical protein BF3101 [Bacteroides fragilis YCH46] >g
. . Bacteroides fragilis NCTC 9343 ...........................................  248  1 hit  [CFB group bacteria]   hypothetical protein BF3101 [Bacteroides fragilis YCH46] >g
. . Bacteroides sp. 2_1_16 ...................................................  248  1 hit  [CFB group bacteria]   hypothetical protein BF3101 [Bacteroides fragilis YCH46] >g
. . Bacteroides plebeius DSM 17135 ...........................................  245  2 hits [CFB group bacteria]   hypothetical protein BACPLE_01583 [Bacteroides plebeius DSM
. . Bacteroides fragilis 3_1_12 ..............................................  244  1 hit  [CFB group bacteria]   putative lipoprotein [Bacteroides fragilis 3_1_12]
. . Pseudomonas syringae pv. oryzae str. 1_6 .................................  244  1 hit  [g-proteobacteria]     hypothetical protein Psyrpo1_21516 [Pseudomonas syringae pv
. . Vibrio mimicus VM223 .....................................................  242  1 hit  [g-proteobacteria]     accessory colonization factor AcfD precursor [Vibrio mimicu
. . Vibrio mimicus MB-451 ....................................................  240  1 hit  [g-proteobacteria]     accessory colonization factor AcfD precursor [Vibrio mimicu
. . Vibrio mimicus VM573 .....................................................  240  2 hits [g-proteobacteria]     accessory colonization factor acfD [Vibrio mimicus VM573]
. . Photorhabdus asymbiotica .................................................  238  1 hit  [enterobacteria]       hypothetical protein PAU_02334 [Photorhabdus asymbiotica]
. . Vibrio sp. RC586 .........................................................  238  1 hit  [g-proteobacteria]     accessory colonization factor AcfD precursor [Vibrio sp. RC
. . Vibrio parahaemolyticus RIMD 2210633 .....................................  238  2 hits [g-proteobacteria]     hypothetical protein VPA1376 [Vibrio parahaemolyticus RIMD 
. . Vibrio parahaemolyticus K5030 ............................................  238  2 hits [g-proteobacteria]     hypothetical protein VPA1376 [Vibrio parahaemolyticus RIMD 
. . Vibrio parahaemolyticus AN-5034 ..........................................  238  1 hit  [g-proteobacteria]     hypothetical protein VPA1376 [Vibrio parahaemolyticus RIMD 
. . Vibrio parahaemolyticus Peru-466 .........................................  238  2 hits [g-proteobacteria]     hypothetical protein VPA1376 [Vibrio parahaemolyticus RIMD 
. . Vibrio cholerae bv. albensis VL426 .......................................  238  1 hit  [g-proteobacteria]     accessory colonization factor AcfD precursor [Vibrio choler
. . Vibrio cholerae RC385 ....................................................  237  1 hit  [g-proteobacteria]     Large exoproteins involved in heme utilization or adhesion 
. . Vibrio vulnificus CMCP6 ..................................................  236  2 hits [g-proteobacteria]     hypothetical protein VV1_0767 [Vibrio vulnificus CMCP6]
. . Grimontia hollisae CIP 101886 ............................................  235  2 hits [g-proteobacteria]     accessory colonization factor AcfD precursor [Grimontia hol
. . Vibrio cholerae O1 biovar El Tor str. N16961 .............................  235  1 hit  [g-proteobacteria]     hypothetical protein VC0845 [Vibrio cholerae O1 biovar El T
. . Vibrio cholerae 2740-80 ..................................................  235  1 hit  [g-proteobacteria]     hypothetical protein VC0845 [Vibrio cholerae O1 biovar El T
. . Vibrio cholerae V52 ......................................................  235  1 hit  [g-proteobacteria]     hypothetical protein VC0845 [Vibrio cholerae O1 biovar El T
. . Vibrio cholerae NCTC 8457 ................................................  235  1 hit  [g-proteobacteria]     hypothetical protein VC0845 [Vibrio cholerae O1 biovar El T
. . Vibrio cholerae B33 ......................................................  235  2 hits [g-proteobacteria]     hypothetical protein VC0845 [Vibrio cholerae O1 biovar El T
. . Vibrio cholerae M66-2 ....................................................  235  1 hit  [g-proteobacteria]     hypothetical protein VC0845 [Vibrio cholerae O1 biovar El T
. . Vibrio cholerae BX 330286 ................................................  235  1 hit  [g-proteobacteria]     hypothetical protein VC0845 [Vibrio cholerae O1 biovar El T
. . Vibrio cholerae RC9 ......................................................  235  1 hit  [g-proteobacteria]     hypothetical protein VC0845 [Vibrio cholerae O1 biovar El T
. . Vibrio cholerae MJ-1236 ..................................................  235  1 hit  [g-proteobacteria]     hypothetical protein VC0845 [Vibrio cholerae O1 biovar El T
. . Vibrio cholerae MO10 .....................................................  235  1 hit  [g-proteobacteria]     hypothetical protein VC0845 [Vibrio cholerae O1 biovar El T
. . Vibrio cholera CIRS 101 ..................................................  235  1 hit  [g-proteobacteria]     hypothetical protein VC0845 [Vibrio cholerae O1 biovar El T
. . Vibrio cholerae INDRE 91/1 ...............................................  235  1 hit  [g-proteobacteria]     hypothetical protein VC0845 [Vibrio cholerae O1 biovar El T
. . Vibrio cholerae V51 ......................................................  235  2 hits [g-proteobacteria]     lipoprotein, putative [Vibrio cholerae V51]
. . Vibrio cholerae TMA 21 ...................................................  234  1 hit  [g-proteobacteria]     accessory colonization factor AcfD precursor [Vibrio choler
. . Vibrio parahaemolyticus AQ3810 ...........................................  234  2 hits [g-proteobacteria]     AcfD [Vibrio parahaemolyticus AQ3810]
. . Vibrio cholerae AM-19226 .................................................  234  1 hit  [g-proteobacteria]     Large exoproteins involved in heme utilization or adhesion 
. . Vibrio cholerae O395 .....................................................  233  1 hit  [g-proteobacteria]     putative lipoprotein [Vibrio cholerae O395]
. . Vibrio cholerae 1587 .....................................................  233  1 hit  [g-proteobacteria]     accessory colonization factor AcfD [Vibrio cholerae 1587] >
. . Vibrio cholerae 623-39 ...................................................  233  1 hit  [g-proteobacteria]     accessory colonization factor AcfD [Vibrio cholerae 1587] >
. . Pseudomonas syringae pv. syringae B728a ..................................  233  2 hits [g-proteobacteria]     hypothetical protein Psyr_4248 [Pseudomonas syringae pv. sy
. . Bacteroides vulgatus ATCC 8482 ...........................................  233  1 hit  [CFB group bacteria]   hypothetical protein BVU_0949 [Bacteroides vulgatus ATCC 84
. . Vibrio cholerae MAK 757 ..................................................  231  1 hit  [g-proteobacteria]     accessory colonization factor AcfD [Vibrio cholerae MAK 757]
. . Clostridium perfringens E str. JGS1987 ...................................  230  1 hit  [firmicutes]           discoidin domain protein [Clostridium perfringens E str. JG
. . Sphingobacterium spiritivorum ATCC 33861 .................................  230  6 hits [CFB group bacteria]   conserved hypothetical protein [Sphingobacterium spiritivor
. . Shewanella halifaxensis HAW-EB4 ..........................................  230  2 hits [g-proteobacteria]     hypothetical protein Shal_3494 [Shewanella halifaxensis HAW
. . Sphingobacterium spiritivorum ATCC 33300 .................................  228  6 hits [CFB group bacteria]   conserved hypothetical protein [Sphingobacterium spiritivor
. . Clostridium perfringens D str. JGS1721 ...................................  226  6 hits [firmicutes]           discoidin domain protein [Clostridium perfringens D str. JG
. . Clostridium perfringens B str. ATCC 3626 .................................  226  2 hits [firmicutes]           discoidin domain protein [Clostridium perfringens B str. AT
. . Clostridium perfringens CPE str. F4969 ...................................  226  2 hits [firmicutes]           discoidin domain protein [Clostridium perfringens CPE str. 
. . Pseudomonas syringae pv. tomato str. DC3000 ..............................  226  2 hits [g-proteobacteria]     hypothetical protein PSPTO_4574 [Pseudomonas syringae pv. t
. . Vibrio cholerae MZO-3 ....................................................  225  2 hits [g-proteobacteria]     accessory colonization factor AcfD [Vibrio cholerae MZO-3]
. . Vibrio parahaemolyticus AQ4037 ...........................................  221  2 hits [g-proteobacteria]     inner membrane lipoprotein [Vibrio parahaemolyticus AQ4037]
. . Listeria grayi DSM 20601 .................................................  218  2 hits [firmicutes]           possible wall-associated protein precursor [Listeria grayi 
. . Vibrio harveyi 1DA3 ......................................................  217  1 hit  [g-proteobacteria]     hypothetical protein VME_34490 [Vibrio harveyi 1DA3]
. . Vibrio sp. Ex25 ..........................................................  217  2 hits [g-proteobacteria]     hypothetical protein VEA_003968 [Vibrio sp. Ex25]
. . Clostridium perfringens C str. JGS1495 ...................................  216  1 hit  [firmicutes]           ABC transporter, permease protein [Clostridium perfringens 
. . Brachybacterium faecium DSM 4810 .........................................  216  1 hit  [high GC Gram+]        hypothetical protein Bfae_09730 [Brachybacterium faecium DS
. . Vibrio harveyi HY01 ......................................................  216  1 hit  [g-proteobacteria]     AcfD [Vibrio harveyi HY01]
. . Shewanella pealeana ATCC 700345 ..........................................  214  2 hits [g-proteobacteria]     inner membrane lipoprotein [Shewanella pealeana ATCC 700345]
. . Vibrio alginolyticus 12G01 ...............................................  214  1 hit  [g-proteobacteria]     hypothetical protein V12G01_20798 [Vibrio alginolyticus 12G
. . Vibrio alginolyticus 40B .................................................  213  1 hit  [g-proteobacteria]     hypothetical protein VMC_31230 [Vibrio alginolyticus 40B]
. . Vibrio fischeri MJ11 .....................................................  208  1 hit  [g-proteobacteria]     inner membrane lipoprotein [Vibrio fischeri MJ11]
. . Clostridium botulinum E3 str. Alaska E43 .................................  208  1 hit  [firmicutes]           fibronectin type III domain protein [Clostridium botulinum 
. . Photobacterium damselae subsp. damselae CIP 102761 .......................  207  1 hit  [g-proteobacteria]     accessory colonization factor AcfD precursor [Photobacteriu
. . Clostridium botulinum E1 str. 'BoNT E Beluga' ............................  204  1 hit  [firmicutes]           fibronectin type III domain protein [Clostridium botulinum 
. . Clostridium perfringens SM101 ............................................  201  1 hit  [firmicutes]           leucine rich repeat domain-containing protein [Clostridium 
. . Clostridium bartlettii DSM 16795 .........................................  200  2 hits [firmicutes]           hypothetical protein CLOBAR_01122 [Clostridium bartlettii D
. . Vibrio orientalis CIP 102891 .............................................  199  1 hit  [g-proteobacteria]     hypothetical protein VIA_003317 [Vibrio orientalis CIP 1028
. . Clostridium perfringens ATCC 13124 .......................................  198  2 hits [firmicutes]           F5/8 type C domain-containing protein [Clostridium perfring
. . Clostridium perfringens str. 13 ..........................................  198  1 hit  [firmicutes]           hypothetical protein CPE1281 [Clostridium perfringens str. 
. . Clostridium perfringens NCTC 8239 ........................................  197  2 hits [firmicutes]           F5/8 type C domain protein [Clostridium perfringens NCTC 82
. . Vibrio parahaemolyticus 16 ...............................................  195  1 hit  [g-proteobacteria]     hypothetical protein VPMS16_1840 [Vibrio parahaemolyticus 1
. . Vibrio sp. MED222 ........................................................  193  1 hit  [g-proteobacteria]     hypothetical protein MED222_12503 [Vibrio sp. MED222]
. . Vibrio mimicus VM603 .....................................................  193  1 hit  [g-proteobacteria]     conserved hypothetical protein [Vibrio mimicus VM603]
. . Mycoplasma penetrans HF-2 ................................................  193  3 hits [mycoplasmas]          integral membrane protein [Mycoplasma penetrans HF-2]
. . Pseudomonas syringae pv. tomato T1 .......................................  191  1 hit  [g-proteobacteria]     hypothetical protein PSPTOT1_2511 [Pseudomonas syringae pv.
. . Vibrio splendidus LGP32 ..................................................  191  1 hit  [g-proteobacteria]     hypothetical protein VS_1058 [Vibrio splendidus LGP32]
. . Bacteroides coprophilus DSM 18228 ........................................  189  1 hit  [CFB group bacteria]   hypothetical protein BACCOPRO_00998 [Bacteroides coprophilu
. . Subdoligranulum variabile DSM 15176 ......................................  188  1 hit  [firmicutes]           putative fibronectin type III domain protein [Subdoligranul
. . Vibrio vulnificus YJ016 ..................................................  186  1 hit  [g-proteobacteria]     hypothetical protein VV2335 [Vibrio vulnificus YJ016]
. . Vibrionales bacterium SWAT-3 .............................................  182  1 hit  [g-proteobacteria]     hypothetical protein VSWAT3_23194 [Vibrionales bacterium SW
. . Shewanella woodyi ATCC 51908 .............................................  181  1 hit  [g-proteobacteria]     hypothetical protein Swoo_1054 [Shewanella woodyi ATCC 5190
. . Eubacterium dolichum DSM 3991 ............................................  180  1 hit  [firmicutes]           hypothetical protein EUBDOL_00526 [Eubacterium dolichum DSM
. . Vibrio fischeri ES114 ....................................................  179  1 hit  [g-proteobacteria]     accessory colonization factor AcfD-like protein, inner memb
. . Bacillus anthracis str. A0488 ............................................  176  1 hit  [firmicutes]           enhancin family protein [Bacillus anthracis str. Ames] >gi|
. . Bacillus anthracis str. A0193 ............................................  176  1 hit  [firmicutes]           enhancin family protein [Bacillus anthracis str. Ames] >gi|
. . Bacillus anthracis str. A0389 ............................................  176  1 hit  [firmicutes]           enhancin family protein [Bacillus anthracis str. Ames] >gi|
. . Bacillus anthracis str. A0174 ............................................  176  1 hit  [firmicutes]           enhancin family protein [Bacillus anthracis str. Ames] >gi|
. . Bacillus anthracis Tsiankovskii-I ........................................  176  1 hit  [firmicutes]           enhancin family protein [Bacillus anthracis str. Ames] >gi|
. . Bacillus anthracis str. A0248 ............................................  176  1 hit  [firmicutes]           enhancin family protein [Bacillus anthracis str. Ames] >gi|
. . Bacillus anthracis str. A0442 ............................................  176  1 hit  [firmicutes]           metallprotease, enhancin family [Bacillus anthracis str. A0
. . Bacillus anthracis str. A0465 ............................................  176  1 hit  [firmicutes]           metallprotease, enhancin family [Bacillus anthracis str. A0
. . Clostridium sp. 7_2_43FAA ................................................  176  1 hit  [firmicutes]           leucine rich repeat domain-containing protein [Clostridium 
. . Shewanella loihica PV-4 ..................................................  175  1 hit  [g-proteobacteria]     hypothetical protein Shew_0921 [Shewanella loihica PV-4]
. . Prevotella melaninogenica ATCC 25845 .....................................  173  1 hit  [CFB group bacteria]   conserved hypothetical protein [Prevotella melaninogenica A
. . Clostridium hathewayi DSM 13479 ..........................................  172  1 hit  [firmicutes]           coagulation factor 5/8 type domain protein [Clostridium hat
. . Pseudomonas aeruginosa PA7 ...............................................  170  1 hit  [g-proteobacteria]     hypothetical protein PSPA7_0675 [Pseudomonas aeruginosa PA7]
. . Clostridium difficile QCD-66c26 ..........................................  169  1 hit  [firmicutes]           hypothetical protein CdifQC_13801 [Clostridium difficile QC
. . Clostridium difficile CIP 107932 .........................................  169  1 hit  [firmicutes]           hypothetical protein CdifQC_13801 [Clostridium difficile QC
. . Clostridium difficile QCD-76w55 ..........................................  169  1 hit  [firmicutes]           hypothetical protein CdifQC_13801 [Clostridium difficile QC
. . Clostridium difficile QCD-97b34 ..........................................  169  1 hit  [firmicutes]           hypothetical protein CdifQC_13801 [Clostridium difficile QC
. . Clostridium difficile QCD-37x79 ..........................................  169  1 hit  [firmicutes]           hypothetical protein CdifQC_13801 [Clostridium difficile QC
. . Clostridium difficile CD196 ..............................................  169  1 hit  [firmicutes]           hypothetical protein CdifQC_13801 [Clostridium difficile QC
. . Clostridium difficile R20291 .............................................  169  1 hit  [firmicutes]           hypothetical protein CdifQC_13801 [Clostridium difficile QC
. . Pseudomonas aeruginosa PACS2 .............................................  167  1 hit  [g-proteobacteria]     hypothetical protein PaerPA_01000570 [Pseudomonas aeruginos
. . Pseudomonas aeruginosa 2192 ..............................................  166  1 hit  [g-proteobacteria]     hypothetical protein PA2G_04490 [Pseudomonas aeruginosa 219
. . Pseudomonas aeruginosa C3719 .............................................  166  1 hit  [g-proteobacteria]     hypothetical protein PACG_03268 [Pseudomonas aeruginosa C37
. . Pseudomonas aeruginosa PAO1 ..............................................  166  1 hit  [g-proteobacteria]     hypothetical protein PA0572 [Pseudomonas aeruginosa PAO1] >
. . Pseudomonas aeruginosa LESB58 ............................................  166  1 hit  [g-proteobacteria]     hypothetical protein PA0572 [Pseudomonas aeruginosa PAO1] >
. . Pseudomonas aeruginosa UCBPP-PA14 ........................................  166  1 hit  [g-proteobacteria]     hypothetical protein PA14_07430 [Pseudomonas aeruginosa UCB
. . Clostridium difficile QCD-63q42 ..........................................  164  1 hit  [firmicutes]           hypothetical protein CdifQCD-6_14221 [Clostridium difficile
. . Clostridium difficile ATCC 43255 .........................................  164  1 hit  [firmicutes]           hypothetical protein CdifA_14696 [Clostridium difficile ATC
. . Clostridium ramosum DSM 1402 .............................................  164  1 hit  [firmicutes]           hypothetical protein CLORAM_00251 [Clostridium ramosum DSM 
. . Clostridium difficile 630 ................................................  163  1 hit  [firmicutes]           hypothetical protein CD2797 [Clostridium difficile 630]
. . Clostridium hiranonis DSM 13275 ..........................................  163  1 hit  [firmicutes]           hypothetical protein CLOHIR_00533 [Clostridium hiranonis DS
. . Mollicutes bacterium D7 ..................................................  163  1 hit  [mycoplasmas]          conserved hypothetical protein [Mollicutes bacterium D7]
. . Shewanella oneidensis MR-1 ...............................................  158  1 hit  [g-proteobacteria]     hypothetical protein SO_0440 [Shewanella oneidensis MR-1]
. . Bacteroides capillosus ATCC 29799 ........................................  158  1 hit  [CFB group bacteria]   hypothetical protein BACCAP_01474 [Bacteroides capillosus A
. . Hahella chejuensis KCTC 2396 .............................................  157  1 hit  [g-proteobacteria]     hypothetical protein HCH_03041 [Hahella chejuensis KCTC 239
. . Eggerthella lenta DSM 2243 ...............................................  154  1 hit  [high GC Gram+]        coagulation factor 5/8 type domain protein [Eggerthella len
. . Shewanella sp. MR-7 ......................................................  154  1 hit  [g-proteobacteria]     hypothetical protein Shewmr7_3586 [Shewanella sp. MR-7]
. . Clostridium botulinum A2 str. Kyoto ......................................  154  1 hit  [firmicutes]           viral enhancin protein [Clostridium botulinum A2 str. Kyoto]
. . Shewanella sp. ANA-3 .....................................................  152  1 hit  [g-proteobacteria]     hypothetical protein Shewana3_0439 [Shewanella sp. ANA-3]
. . Clostridium botulinum F str. Langeland ...................................  150  1 hit  [firmicutes]           enhancing factor [Clostridium botulinum F str. Langeland]
. . Clostridium botulinum Bf .................................................  148  1 hit  [firmicutes]           enhancing factor [Clostridium botulinum Bf]
. . Clostridium botulinum Ba4 str. 657 .......................................  147  1 hit  [firmicutes]           enhancing factor [Clostridium botulinum Ba4 str. 657]
. . Salmonella enterica subsp. arizonae serovar 62:z4,z23:-- .................  147  1 hit  [enterobacteria]       hypothetical protein SARI_04252 [Salmonella enterica subsp.
. . Clostridium botulinum B1 str. Okra .......................................  147  1 hit  [firmicutes]           enhancing factor [Clostridium botulinum B1 str. Okra]
. . Bacteroides finegoldii DSM 17565 .........................................  147  1 hit  [CFB group bacteria]   conserved hypothetical protein [Bacteroides finegoldii DSM 
. . Shewanella sp. MR-4 ......................................................  146  1 hit  [g-proteobacteria]     hypothetical protein Shewmr4_0443 [Shewanella sp. MR-4]
. . Bacteroides sp. D2 .......................................................  141  1 hit  [CFB group bacteria]   hypothetical protein BacD2_03774 [Bacteroides sp. D2]
. . Shewanella baltica OS185 .................................................  141  1 hit  [g-proteobacteria]     hypothetical protein Shew185_3916 [Shewanella baltica OS185]
. . Shewanella amazonensis SB2B ..............................................  140  1 hit  [g-proteobacteria]     hypothetical protein Sama_0393 [Shewanella amazonensis SB2B]
. . Shewanella baltica OS195 .................................................  140  1 hit  [g-proteobacteria]     hypothetical protein Sbal195_4038 [Shewanella baltica OS195]
. . Shewanella baltica OS155 .................................................  137  1 hit  [g-proteobacteria]     hypothetical protein Sbal_0418 [Shewanella baltica OS155]
. . Shewanella baltica OS223 .................................................  137  1 hit  [g-proteobacteria]     hypothetical protein Sbal223_3842 [Shewanella baltica OS223]
. . Pantoea sp. At-9b ........................................................  134  1 hit  [enterobacteria]       hypothetical protein Pat9bDRAFT_3619 [Pantoea sp. At-9b]
. . Clostridium difficile QCD-32g58 ..........................................  124  2 hits [firmicutes]           hypothetical protein CdifQ_04003262 [Clostridium difficile 
. . Salmonella enterica subsp. enterica serovar Dublin str. CT_02021853 ......  119  1 hit  [enterobacteria]       viral enhancin protein [Salmonella enterica subsp. enterica
. . Salmonella enterica subsp. enterica serovar Enteritidis str. P125109 .....  119  1 hit  [enterobacteria]       putative viral enhancing factor [Salmonella enterica subsp.
. . Salmonella enterica subsp. enterica serovar Schwarzengrund str. SL480 ....  119  1 hit  [enterobacteria]       viral enhancin protein [Salmonella enterica subsp. enterica
. . Salmonella enterica subsp. enterica serovar Schwarzengrund str. CVM19633 .  119  1 hit  [enterobacteria]       viral enhancin protein [Salmonella enterica subsp. enterica
. . Salmonella enterica subsp. enterica serovar Javiana str. GA_MM04042433 ...  118  1 hit  [enterobacteria]       viral enhancin protein [Salmonella enterica subsp. enterica
. . Yersinia pseudotuberculosis IP 31758 .....................................  116  1 hit  [enterobacteria]       viral enhancin protein [Yersinia pseudotuberculosis IP 3175
. . Yersinia pseudotuberculosis IP 32953 .....................................  116  1 hit  [enterobacteria]       enhancing factor [Yersinia pseudotuberculosis IP 32953]
. . Yersinia pseudotuberculosis YPIII ........................................  116  1 hit  [enterobacteria]       peptidase M60 viral enhancin protein [Yersinia pseudotuberc
. . Yersinia pseudotuberculosis PB1/+ ........................................  116  1 hit  [enterobacteria]       peptidase M60 viral enhancin protein [Yersinia pseudotuberc
. . Clostridium butyricum 5521 ...............................................  114  1 hit  [firmicutes]           putative exported protein [Clostridium butyricum 5521] >gi|
. . Clostridium butyricum E4 str. BoNT E BL5262 ..............................  114  1 hit  [firmicutes]           putative exported protein [Clostridium butyricum 5521] >gi|
. . Yersinia pestis KIM 10 ...................................................  111  1 hit  [enterobacteria]       enhancing factor [Yersinia pestis KIM] >gi|45440347|ref|NP_
. . Yersinia pestis biovar Microtus str. 91001 ...............................  111  1 hit  [enterobacteria]       enhancing factor [Yersinia pestis KIM] >gi|45440347|ref|NP_
. . Yersinia pestis CO92 .....................................................  111  1 hit  [enterobacteria]       enhancing factor [Yersinia pestis KIM] >gi|45440347|ref|NP_
. . Yersinia pestis Antiqua ..................................................  111  1 hit  [enterobacteria]       enhancing factor [Yersinia pestis Antiqua] >gi|108813490|re
. . Yersinia pestis Nepal516 .................................................  111  2 hits [enterobacteria]       enhancing factor [Yersinia pestis Antiqua] >gi|108813490|re
. . Yersinia pestis CA88-4125 ................................................  111  1 hit  [enterobacteria]       enhancing factor [Yersinia pestis Antiqua] >gi|108813490|re
. . Yersinia pestis Angola ...................................................  111  1 hit  [enterobacteria]       enhancing factor [Yersinia pestis Antiqua] >gi|108813490|re
. . Yersinia pestis biovar Orientalis str. IP275 .............................  111  1 hit  [enterobacteria]       enhancing factor [Yersinia pestis Antiqua] >gi|108813490|re
. . Yersinia pestis biovar Antiqua str. E1979001 .............................  111  1 hit  [enterobacteria]       enhancing factor [Yersinia pestis Antiqua] >gi|108813490|re
. . Yersinia pestis biovar Antiqua str. UG05-0454 ............................  111  1 hit  [enterobacteria]       enhancing factor [Yersinia pestis Antiqua] >gi|108813490|re
. . Yersinia pestis biovar Mediaevalis str. K1973002 .........................  111  1 hit  [enterobacteria]       enhancing factor [Yersinia pestis Antiqua] >gi|108813490|re
. . Yersinia pestis FV-1 .....................................................  111  1 hit  [enterobacteria]       enhancing factor [Yersinia pestis Antiqua] >gi|108813490|re
. . Yersinia pestis Pestoides A ..............................................  111  1 hit  [enterobacteria]       enhancing factor [Yersinia pestis Antiqua] >gi|108813490|re
. . Yersinia pestis biovar Orientalis str. PEXU2 .............................  111  1 hit  [enterobacteria]       enhancing factor [Yersinia pestis Antiqua] >gi|108813490|re
. . Yersinia pestis biovar Orientalis str. India 195 .........................  111  1 hit  [enterobacteria]       enhancing factor [Yersinia pestis Antiqua] >gi|108813490|re
. . Yersinia pestis biovar Orientalis str. F1991016 ..........................  111  1 hit  [enterobacteria]       viral enhancin protein [Yersinia pestis biovar Orientalis s
. . Yersinia pestis biovar Antiqua str. B42003004 ............................  111  1 hit  [enterobacteria]       viral enhancin protein [Yersinia pestis biovar Orientalis s
. . Yersinia pestis biovar Orientalis str. MG05-1020 .........................  111  1 hit  [enterobacteria]       viral enhancin protein [Yersinia pestis biovar Orientalis s
. . Yersinia pestis Pestoides F ..............................................  111  1 hit  [enterobacteria]       enhancing factor [Yersinia pestis Pestoides F]
. . Yersinia pestis KIM D27 ..................................................  110  1 hit  [enterobacteria]       viral enhancin protein [Yersinia pestis KIM D27]
. . Lactobacillus jensenii 27-2-CHN ..........................................  109  1 hit  [firmicutes]           enhancin family protein [Lactobacillus jensenii 27-2-CHN]
. . Lactobacillus jensenii 208-1 .............................................  108  1 hit  [firmicutes]           putative enhancing factor [Lactobacillus jensenii 208-1]
. . Lactobacillus jensenii 115-3-CHN .........................................  108  1 hit  [firmicutes]           enhancing factor [Lactobacillus jensenii 115-3-CHN]
. . Yersinia kristensenii ATCC 33638 .........................................   94  1 hit  [enterobacteria]       Enhancing factor (Viral) [Yersinia kristensenii ATCC 33638]
. . Staphylococcus carnosus subsp. carnosus TM300 ............................   84  1 hit  [firmicutes]           putative surface associated protein with similarity to prot
. . Comamonas testosteroni KF-1 ..............................................   75  1 hit  [b-proteobacteria]     conserved hypothetical protein [Comamonas testosteroni KF-1]
. . Clostridium thermocellum DSM 2360 ........................................   54  1 hit  [firmicutes]           Carbohydrate-binding CenC domain protein [Clostridium therm
. . Clostridium thermocellum ATCC 27405 ......................................   53  1 hit  [firmicutes]           carbohydrate-binding, CenC-like protein [Clostridium thermo
. . Clostridium thermocellum JW20 ............................................   52  1 hit  [firmicutes]           Carbohydrate-binding CenC domain protein [Clostridium therm
. . Clostridium cellulolyticum H10 ...........................................   52  1 hit  [firmicutes]           carbohydrate-binding, CenC-like protein [Clostridium cellul
. Lymantria dispar MNPV ------------------------------------------------------  111  2 hits [viruses]              viral enhancing factor 2 [Lymantria dispar MNPV]
. Xestia c-nigrum granulovirus ...............................................  109  2 hits [viruses]              ORF152 [Xestia c-nigrum granulovirus]
. Helicoverpa armigera granulovirus ..........................................  107  2 hits [viruses]              enhancin-2 [Helicoverpa armigera granulovirus]
. Choristoneura fumiferana MNPV (spruce budworm nuclear...) ..................   89  1 hit  [viruses]              enhancin-like [Choristoneura fumiferana MNPV]
. Euproctis pseudoconspersa nucleopolyhedrovirus .............................   84  1 hit  [viruses]              vef [Euproctis pseudoconspersa nucleopolyhedrovirus]
. Agrotis ipsilon multiple nucleopolyhedrovirus ..............................   68  1 hit  [viruses]              agip82 [Agrotis ipsilon multiple nucleopolyhedrovirus]
. Helicoverpa armigera multiple nucleopolyhedrovirus .........................   68  1 hit  [viruses]              putative viral enhancing factor [Helicoverpa armigera multi
. Mamestra configurata NPV-B .................................................   68  1 hit  [viruses]              putative viral enhancing factor [Mamestra configurata NPV-B]
. Agrotis segetum nucleopolyhedrovirus .......................................   64  2 hits [viruses]              VEF-2 [Agrotis segetum nucleopolyhedrovirus]
. Mamestra configurata NPV-A .................................................   64  1 hit  [viruses]              viral enhancing factor VEF [Mamestra configurata NPV-A]
. Agrotis segetum granulovirus ...............................................   61  1 hit  [viruses]              ORF55 [Agrotis segetum granulovirus]
```

---

**Organism Report**

```
  Trichomonas vaginalis G3 [trichomonads] taxid 412133
 ref|XP_001313628.1| Immuno-dominant variable surface antig...     497  9e-139
 ref|XP_001583909.1| Immuno-dominant variable surface antig...     459  2e-127
 ref|XP_001582477.1| Immuno-dominant variable surface antig...     451  6e-125
 ref|XP_001310029.1| Immuno-dominant variable surface antig...     421  8e-116
 ref|XP_001330197.1| Immuno-dominant variable surface antig...     400  2e-109
 ref|XP_001304497.1| Immuno-dominant variable surface antig...     376  4e-102
 ref|XP_001313082.1| Immuno-dominant variable surface antig...     342  4e-92
 ref|XP_001319600.1| Immuno-dominant variable surface antig...     290  2e-76
 ref|XP_001579914.1| Immuno-dominant variable surface antig...     263  3e-68
 ref|XP_001299523.1| Immuno-dominant variable surface antig...     205  8e-51
 ref|XP_001322037.1| hypothetical protein [Trichomonas vagi...     174  2e-41
 ref|XP_001330484.1| hypothetical protein [Trichomonas vagi...     172  8e-41
 ref|XP_001319601.1| Immuno-dominant variable surface antig...     162  9e-38
 ref|XP_001326787.1| hypothetical protein [Trichomonas vagi...     141  1e-31
 ref|XP_001583210.1| F5/8 type C domain containing protein ...     131  2e-28
 ref|XP_001317727.1| hypothetical protein [Trichomonas vagi...     131  2e-28
 ref|XP_001323206.1| hypothetical protein [Trichomonas vagi...     129  4e-28
 ref|XP_001326227.1| hypothetical protein [Trichomonas vagi...     129  8e-28
 ref|XP_001325313.1| hypothetical protein [Trichomonas vagi...     127  3e-27
 ref|XP_001319139.1| hypothetical protein [Trichomonas vagi...     123  4e-26
 ref|XP_001310435.1| hypothetical protein [Trichomonas vagi...     122  1e-25
 ref|XP_001297964.1| hypothetical protein [Trichomonas vagi...     119  9e-25
 ref|XP_001327547.1| hypothetical protein [Trichomonas vagi...     116  8e-24
 ref|XP_001580677.1| hypothetical protein [Trichomonas vagi...      97  3e-18
 ref|XP_001319226.1| hypothetical protein [Trichomonas vagi...      77  3e-12
 ref|XP_001308807.1| Clan MA, family M1, aminopeptidase N-l...      52  8e-05

  Chthoniobacter flavus Ellin428 [verrucomicrobia] taxid 497964
 ref|ZP_03133174.1| conserved hypothetical protein [Chthoni...     356  2e-96

  Bacillus cereus ATCC 10987 [firmicutes] taxid 222523
 ref|NP_981496.1| hypothetical protein BCE_5203 [Bacillus c...     350  2e-94

  Danio rerio (zebra fish, ...) [bony fishes] taxid 7955
 ref|XP_683371.3| PREDICTED: hypothetical protein [Danio re...     349  3e-94
 ref|XP_683284.3| PREDICTED: hypothetical protein [Danio re...     348  7e-94
 ref|XP_001923464.1| PREDICTED: hypothetical protein [Danio...     347  2e-93
 ref|NP_001082796.1| hypothetical protein LOC323315 [Danio ...     339  4e-91

  Bacillus thuringiensis serovar pakistani str. T13001 [firmicutes] taxid 527027
 ref|ZP_04122922.1| hypothetical protein bthur0005_47520 [B...     349  4e-94
 ref|ZP_04121263.1| Metallprotease, enhancin [Bacillus thur...     174  2e-41

  Geobacillus sp. Y412MC10 [firmicutes] taxid 481743
 ref|YP_003244705.1| hypothetical protein GYMC10_4678 [Geob...     349  4e-94
 ref|YP_003244709.1| hypothetical protein GYMC10_4682 [Geob...     336  3e-90

  Bos taurus (cow, ...) [even-toed ungulates] taxid 9913
 ref|NP_001092524.1| hypothetical protein LOC533126 [Bos ta...     349  5e-94
 ref|NP_001095394.1| hypothetical protein LOC510320 [Bos ta...     327  2e-87

  Bacillus cereus m1293 [firmicutes] taxid 526973
 ref|ZP_04325889.1| hypothetical protein bcere0001_47200 [B...     347  2e-93

  Equus caballus (equine, ...) [odd-toed ungulates] taxid 9796
 ref|XP_001502235.2| PREDICTED: hypothetical protein [Equus...     347  2e-93
 ref|XP_001490568.1| PREDICTED: similar to Protein FAM139A ...     331  8e-89

  Bacillus thuringiensis serovar sotto str. T04001 [firmicutes] taxid 527026
 ref|ZP_04129181.1| hypothetical protein bthur0004_49610 [B...     347  2e-93
 ref|ZP_04130357.1| hypothetical protein bthur0004_62240 [B...     306  4e-81

  Bacillus thuringiensis serovar konkukian str. 97-27 [firmicutes] taxid 281309
 ref|YP_039079.1| wall-associated protein [Bacillus thuring...     347  2e-93
 ref|YP_037494.1| enhancin family protein [Bacillus thuring...     174  2e-41

  Canis lupus familiaris (dogs) [carnivores] taxid 9615
 ref|XP_539846.2| PREDICTED: hypothetical protein XP_539846...     347  2e-93
 ref|XP_848591.1| PREDICTED: hypothetical protein XP_843498...     347  2e-93
 ref|XP_539847.2| PREDICTED: hypothetical protein XP_539847...     334  1e-89

  Bacillus weihenstephanensis KBAB4 [firmicutes] taxid 315730
 ref|YP_001647664.1| wall-associated protein precursor [Bac...     346  2e-93
 ref|YP_001645178.1| S-layer domain-containing protein [Bac...     315  5e-84
 ref|YP_001642566.1| fibronectin type III domain-containing...     298  9e-79

  Pan troglodytes [primates] taxid 9598
 ref|XP_519454.2| PREDICTED: similar to KIAA0738 protein [P...     346  3e-93
 ref|XP_519453.2| PREDICTED: hypothetical protein [Pan trog...     344  1e-92

  Homo sapiens (man) [primates] taxid 9606
 ref|NP_055534.1| hypothetical protein LOC9747 [Homo sapiens]      346  3e-93
 ref|NP_001123498.1| hypothetical protein LOC285966 isoform...     264  1e-68
 ref|NP_775949.2| hypothetical protein LOC285966 isoform A ...     264  1e-68
 ref|NP_001123497.1| hypothetical protein LOC285966 isoform...     264  1e-68
 ref|XP_002346094.1| PREDICTED: similar to FAM115A protein ...     150  3e-34

  Bacillus cereus AH820 [firmicutes] taxid 405535
 ref|YP_002454093.1| wall-associated protein [Bacillus cere...     346  4e-93
 ref|YP_002454094.1| wall-associated protein [Bacillus cere...     324  1e-86
 ref|YP_002452359.1| metallprotease, enhancin family [Bacil...     175  7e-42

  Bacillus cereus Rock3-42 [firmicutes] taxid 526985
 ref|ZP_04225257.1| hypothetical protein bcere0021_48880 [B...     345  4e-93
 ref|ZP_04223521.1| Metallprotease, enhancin [Bacillus cere...     169  7e-40

  Bacillus thuringiensis serovar andalousiensis BGSC 4AW1 [firmicutes] taxid 527032
 ref|ZP_04099141.1| hypothetical protein bthur0009_47800 [B...     345  5e-93
 ref|ZP_04097503.1| Metallprotease, enhancin [Bacillus thur...     174  2e-41

  Bacillus cereus 95/8201 [firmicutes] taxid 526979
 ref|ZP_04253785.1| hypothetical protein bcere0016_48840 [B...     345  5e-93
 ref|ZP_04253786.1| hypothetical protein bcere0016_48850 [B...     326  3e-87
 ref|ZP_04252126.1| Metallprotease, enhancin [Bacillus cere...     174  2e-41

  Bacillus thuringiensis serovar monterrey BGSC 4AJ1 [firmicutes] taxid 527022
 ref|ZP_04111067.1| hypothetical protein bthur0007_49140 [B...     345  5e-93
 ref|ZP_04112386.1| S-layer domain protein [Bacillus thurin...     304  1e-80
 ref|ZP_04111485.1| S-layer domain protein [Bacillus thurin...     300  2e-79
 ref|ZP_04109312.1| Metallprotease, enhancin [Bacillus thur...     174  2e-41

  Bacillus thuringiensis serovar pondicheriensis BGSC 4BA1 [firmicutes] taxid 527029
 ref|ZP_04093100.1| hypothetical protein bthur0010_47720 [B...     345  6e-93
 ref|ZP_04093101.1| hypothetical protein bthur0010_47730 [B...     325  5e-87
 ref|ZP_04091477.1| Metallprotease, enhancin [Bacillus thur...     174  2e-41

  Bacillus thuringiensis IBL 200 [firmicutes] taxid 527019
 ref|ZP_04074710.1| hypothetical protein bthur0013_50430 [B...     345  6e-93
 ref|ZP_04071470.1| Fibronectin type III domain protein [Ba...     291  1e-76

  Bacillus thuringiensis serovar israelensis ATCC 35646 [firmicutes] taxid 339854
 ref|ZP_00741467.1| Wall-associated protein precursor [Baci...     345  7e-93
 ref|ZP_00739797.1| Wall-associated protein precursor [Baci...     305  9e-81
 ref|ZP_00738692.1| Wall-associated protein precursor [Baci...     300  2e-79

  Bacillus thuringiensis IBL 4222 [firmicutes] taxid 527020
 ref|ZP_04067686.1| hypothetical protein bthur0014_47230 [B...     344  1e-92
 ref|ZP_04068100.1| S-layer domain protein [Bacillus thurin...     307  1e-81

  Bacillus cereus G9842 [firmicutes] taxid 405531
 ref|YP_002448623.1| wall-associated protein [Bacillus cere...     344  1e-92
 ref|YP_002447016.1| S-layer domain protein [Bacillus cereu...     308  6e-82

  Bacillus cereus W [firmicutes] taxid 405917
 ref|ZP_03100686.1| wall-associated protein [Bacillus cereu...     343  2e-92
 ref|ZP_03100442.1| wall-associated protein [Bacillus cereu...     325  7e-87
 ref|ZP_03102903.1| metallprotease, enhancin family [Bacill...     174  2e-41

  Macaca mulatta (rhesus macaque, ...) [primates] taxid 9544
 ref|XP_001092570.1| PREDICTED: hypothetical protein [Macac...     343  2e-92
 ref|XP_001093192.1| PREDICTED: similar to experimental aut...      71  2e-10

  Bacillus cereus AH1271 [firmicutes] taxid 526992
 ref|ZP_04188665.1| hypothetical protein bcere0028_47380 [B...     342  3e-92

  Bacillus cereus AH603 [firmicutes] taxid 526990
 ref|ZP_04200035.1| hypothetical protein bcere0026_47920 [B...     342  4e-92
 ref|ZP_04200658.1| S-layer domain protein [Bacillus cereus...     312  5e-83
 ref|ZP_04200907.1| S-layer domain protein [Bacillus cereus...     305  7e-81

  Bacillus cereus AH621 [firmicutes] taxid 526972
 ref|ZP_04297480.1| hypothetical protein bcere0007_47240 [B...     342  6e-92
 ref|ZP_04298287.1| S-layer domain protein [Bacillus cereus...     312  5e-83
 ref|ZP_04298280.1| S-layer domain protein [Bacillus cereus...     300  2e-79

  Bacillus cereus H3081.97 [firmicutes] taxid 451708
 ref|ZP_03237588.1| wall-associated protein [Bacillus cereu...     342  6e-92

  Bacillus thuringiensis serovar berliner ATCC 10792 [firmicutes] taxid 527031
 ref|ZP_04104770.1| hypothetical protein bthur0008_48610 [B...     341  8e-92
 ref|ZP_04103158.1| S-layer domain protein [Bacillus thurin...     304  2e-80
 ref|ZP_04103016.1| Metallprotease, enhancin [Bacillus thur...     171  2e-40
 ref|ZP_04101632.1| Fibronectin type III domain protein [Ba...     155  8e-36
 ref|ZP_04105302.1| Fibronectin type III domain protein [Ba...     154  2e-35

  Bacillus thuringiensis serovar thuringiensis str. T01001 [firmicutes] taxid 527025
 ref|ZP_04135719.1| hypothetical protein bthur0003_49080 [B...     341  8e-92
 ref|ZP_04134096.1| S-layer domain protein [Bacillus thurin...     304  2e-80
 ref|ZP_04132535.1| Fibronectin type III domain protein [Ba...     293  2e-77
 ref|ZP_04133950.1| Metallprotease, enhancin [Bacillus thur...     171  2e-40
 ref|ZP_04136244.1| Fibronectin type III domain protein [Ba...     140  3e-31

  Bacillus thuringiensis Bt407 [firmicutes] taxid 527021
 ref|ZP_04142094.1| hypothetical protein bthur0002_49580 [B...     341  8e-92
 ref|ZP_04140386.1| S-layer domain protein [Bacillus thurin...     304  2e-80
 ref|ZP_04138897.1| Fibronectin type III domain protein [Ba...     293  2e-77
 ref|ZP_04140245.1| Metallprotease, enhancin [Bacillus thur...     171  2e-40

  Bacillus cereus AH187 [firmicutes] taxid 405534
 ref|YP_002341115.1| wall-associated protein [Bacillus cere...     341  9e-92

  Bacillus cereus Q1 [firmicutes] taxid 361100
 ref|YP_002532570.1| Wall-associated protein precursor [Bac...     341  9e-92

  Bacillus cereus Rock4-2 [firmicutes] taxid 526987
 ref|ZP_04214770.1| hypothetical protein bcere0023_49240 [B...     341  9e-92
 ref|ZP_04215346.1| S-layer domain protein [Bacillus cereus...     304  1e-80
 ref|ZP_04213119.1| Metallprotease, enhancin [Bacillus cere...     171  1e-40

  Bacillus cereus BDRD-ST26 [firmicutes] taxid 526975
 ref|ZP_04270324.1| hypothetical protein bcere0013_48840 [B...     340  1e-91

  Bacillus thuringiensis str. Al Hakam [firmicutes] taxid 412694
 ref|YP_897294.1| wall-associated protein [Bacillus thuring...     340  1e-91

  Bacillus cereus 03BB108 [firmicutes] taxid 451709
 ref|ZP_03114768.1| wall-associated protein [Bacillus cereu...     340  1e-91

  Bacillus cereus 03BB102 [firmicutes] taxid 572264
 ref|YP_002752424.1| wall-associated protein [Bacillus cere...     340  1e-91

  Mus musculus (mouse) [rodents] taxid 10090
 ref|NP_084206.1| hypothetical protein LOC77574 [Mus musculus]     340  1e-91
 ref|NP_666286.1| hypothetical protein LOC232748 [Mus muscu...     335  4e-90
 ref|NP_981933.1| experimental autoimmune prostatitis antig...     325  5e-87

  Bacillus cereus B4264 [firmicutes] taxid 405532
 ref|YP_002369867.1| wall-associated protein [Bacillus cere...     340  1e-91
 ref|YP_002366602.1| S-layer domain protein [Bacillus cereu...     295  6e-78
 ref|YP_002368097.1| metallprotease, enhancin family [Bacil...     171  2e-40

  Bacillus thuringiensis serovar huazhongensis BGSC 4BD1 [firmicutes] taxid 527030
 ref|ZP_04087070.1| hypothetical protein bthur0011_47670 [B...     340  2e-91
 ref|ZP_04087543.1| S-layer domain protein [Bacillus thurin...     305  5e-81
 ref|ZP_04085415.1| Metallprotease, enhancin [Bacillus thur...     173  5e-41

  Bacillus cereus BGSC 6E1 [firmicutes] taxid 526970
 ref|ZP_04314451.1| hypothetical protein bcere0004_48420 [B...     340  2e-91

  Rattus norvegicus (brown rat, ...) [rodents] taxid 10116
 ref|XP_001072497.1| PREDICTED: hypothetical protein [Rattu...     339  5e-91
 ref|XP_342676.3| PREDICTED: hypothetical protein [Rattus n...     339  5e-91
 ref|NP_001009534.1| experimental autoimmune prostatitis an...     327  1e-87
 ref|XP_001072414.1| PREDICTED: hypothetical protein [Rattu...     326  4e-87
 ref|XP_001057103.1| PREDICTED: hypothetical protein [Rattu...     326  4e-87

  Bacillus cereus 172560W [firmicutes] taxid 526967
 ref|ZP_04308684.1| hypothetical protein bcere0005_46970 [B...     339  5e-91
 ref|ZP_04309267.1| S-layer domain protein [Bacillus cereus...     303  3e-80
 ref|ZP_04307018.1| Metallprotease, enhancin [Bacillus cere...     171  2e-40

  Bacillus thuringiensis serovar kurstaki str. T03a001 [firmicutes] taxid 527023
 ref|ZP_04117334.1| hypothetical protein bthur0006_46840 [B...     338  6e-91
 ref|ZP_04117796.1| S-layer domain protein [Bacillus thurin...     300  1e-79
 ref|ZP_04115705.1| Metallprotease, enhancin [Bacillus thur...     171  2e-40

  Bacillus cereus F65185 [firmicutes] taxid 526989
 ref|ZP_04205749.1| hypothetical protein bcere0025_47080 [B...     337  1e-90
 ref|ZP_04206946.1| S-layer domain protein [Bacillus cereus...     303  2e-80
 ref|ZP_04204064.1| Metallprotease, enhancin [Bacillus cere...     171  2e-40

  Bacillus cereus BDRD-ST24 [firmicutes] taxid 526974
 ref|ZP_04275957.1| hypothetical protein bcere0012_47390 [B...     337  1e-90
 ref|ZP_04274345.1| Metallprotease, enhancin [Bacillus cere...     167  2e-39

  Bacillus cereus Rock1-15 [firmicutes] taxid 526982
 ref|ZP_04242042.1| hypothetical protein bcere0018_47450 [B...     337  2e-90
 ref|ZP_04238966.1| Fibronectin type III domain protein [Ba...     293  2e-77
 ref|ZP_04240369.1| Metallprotease, enhancin [Bacillus cere...     173  4e-41

  Bacillus cereus BDRD-Cer4 [firmicutes] taxid 526978
 ref|ZP_04259276.1| hypothetical protein bcere0015_47530 [B...     337  2e-90
 ref|ZP_04257640.1| Metallprotease, enhancin [Bacillus cere...     173  3e-41

  Bacillus cereus AH1134 [firmicutes] taxid 405533
 ref|ZP_03231149.1| wall-associated protein [Bacillus cereu...     336  3e-90
 ref|ZP_03233872.1| fibronectin type III domain protein [Ba...     300  1e-79
 ref|ZP_03234378.1| fibronectin type III domain protein [Ba...     289  4e-76
 ref|ZP_03230028.1| metallprotease, enhancin family [Bacill...     171  1e-40

  Bacillus cereus AH676 [firmicutes] taxid 526991
 ref|ZP_04194307.1| hypothetical protein bcere0027_47080 [B...     335  4e-90
 ref|ZP_04191382.1| Fibronectin type III domain protein [Ba...     293  2e-77
 ref|ZP_04192671.1| Metallprotease, enhancin [Bacillus cere...     175  7e-42

  Bacillus cereus MM3 [firmicutes] taxid 526971
 ref|ZP_04303264.1| hypothetical protein bcere0006_48340 [B...     334  1e-89
 ref|ZP_04300725.1| Fibronectin type III domain protein [Ba...     289  3e-76
 ref|ZP_04301600.1| Metallprotease, enhancin [Bacillus cere...     178  1e-42

  Bacillus cereus NVH0597-99 [firmicutes] taxid 451707
 ref|ZP_03106796.1| wall-associated protein [Bacillus cereu...     334  1e-89
 ref|ZP_03105337.1| metallprotease, enhancin family [Bacill...     174  1e-41

  Taeniopygia guttata (zebra finch) [birds] taxid 59729
 ref|XP_002190961.1| PREDICTED: similar to Protein FAM115C ...     333  3e-89
 ref|XP_002197366.1| PREDICTED: similar to Protein FAM115C,...     176  5e-42

  Bacillus cereus ATCC 10876 [firmicutes] taxid 526980
 ref|ZP_04320291.1| hypothetical protein bcere0002_49870 [B...     332  4e-89
 ref|ZP_04319929.1| S-layer domain protein [Bacillus cereus...     303  2e-80
 ref|ZP_04320752.1| Fibronectin type III domain protein [Ba...     285  1e-74
 ref|ZP_04318470.1| Metallprotease, enhancin [Bacillus cere...     171  1e-40

  Bacillus thuringiensis serovar tochigiensis BGSC 4Y1 [firmicutes] taxid 527024
 ref|ZP_04148407.1| hypothetical protein bthur0001_49700 [B...     329  5e-88
 ref|ZP_04146606.1| Metallprotease, enhancin [Bacillus thur...     174  1e-41

  Ornithorhynchus anatinus (duck-billed platypus, ...) [monotremes] taxid 9258
 ref|XP_001509330.1| PREDICTED: similar to seven transmembr...     327  2e-87
 ref|XP_001521332.1| PREDICTED: similar to BC011487 protein...     325  4e-87
 ref|XP_001521183.1| PREDICTED: similar to FLJ00264 protein...     291  1e-76

  Planctomyces limnophilus DSM 3776 [planctomycetes] taxid 521674
 ref|ZP_04428328.1| hypothetical protein PlimDRAFT_30570 [P...     327  2e-87

  Bacillus cereus BDRD-ST196 [firmicutes] taxid 526976
 ref|ZP_04264662.1| hypothetical protein bcere0014_47770 [B...     326  3e-87
 ref|ZP_04262242.1| S-layer domain protein [Bacillus cereus...     310  2e-82
 ref|ZP_04265361.1| Fibronectin type III domain protein [Ba...     292  8e-77

  Bacillus cereus G9241 [firmicutes] taxid 269801
 ref|ZP_00238015.1| reticulocyte binding protein [Bacillus ...     325  5e-87

  Bacillus thuringiensis serovar pulsiensis BGSC 4CC1 [firmicutes] taxid 527028
 ref|ZP_04081237.1| hypothetical protein bthur0012_49000 [B...     325  7e-87
 ref|ZP_04079571.1| Metallprotease, enhancin [Bacillus thur...     174  1e-41

  Verrucomicrobium spinosum DSM 4136 [verrucomicrobia] taxid 240016
 ref|ZP_02925937.1| hypothetical protein VspiD_04825 [Verru...     324  2e-86

  Xenopus laevis (common platanna, ...) [frogs & toads] taxid 8355
 ref|NP_001088165.1| hypothetical protein LOC494989 [Xenopu...     323  2e-86

  Bacillus cereus ATCC 14579 [firmicutes] taxid 226900
 ref|NP_834723.1| wall-associated protein precursor [Bacill...     321  1e-85
 ref|NP_833119.1| enhancin [Bacillus cereus ATCC 14579]            172  6e-41

  Bacillus anthracis str. A1055 [firmicutes] taxid 280355
 ref|ZP_05186275.1| wall-associated protein [Bacillus anthr...     321  1e-85
 ref|ZP_05185152.1| metallprotease, enhancin family protein...     177  2e-42

  Bacillus cereus E33L [firmicutes] taxid 288681
 ref|YP_086361.1| wall-associated protein [Bacillus cereus ...     320  2e-85
 ref|YP_084679.1| enhancin family protein [Bacillus cereus ...     178  1e-42
 ref|YP_245638.1| enhancin family protein [Bacillus cereus ...     159  6e-37

  Bacillus cereus ATCC 4342 [firmicutes] taxid 526977
 ref|ZP_04286723.1| hypothetical protein bcere0010_48380 [B...     320  2e-85

  Bacillus anthracis str. Ames [firmicutes] taxid 198094
 ref|NP_847482.1| hypothetical protein BA_5305 [Bacillus an...     319  4e-85
 ref|NP_845726.1| enhancin family protein [Bacillus anthrac...     176  4e-42

  Bacillus anthracis str. 'Ames Ancestor' [firmicutes] taxid 261594
 ref|YP_021963.1| hypothetical protein GBAA5305 [Bacillus a...     319  4e-85
 ref|YP_020076.1| enhancin family protein [Bacillus anthrac...     176  4e-42

  Bacillus anthracis str. Sterne [firmicutes] taxid 260799
 ref|YP_031170.1| hypothetical protein BAS4929 [Bacillus an...     319  4e-85
 ref|YP_029447.1| enhancin family protein [Bacillus anthrac...     176  4e-42

  Bacillus anthracis str. CDC 684 [firmicutes] taxid 568206
 ref|YP_002817846.1| hypothetical protein BAMEG_5358 [Bacil...     319  4e-85
 ref|YP_002813791.1| metallprotease, enhancin family [Bacil...     176  4e-42

  Bacillus anthracis str. CNEVA-9066 [firmicutes] taxid 280354
 ref|ZP_05150351.1| hypothetical protein BantC_21900 [Bacil...     319  4e-85
 ref|ZP_05149820.1| metallprotease, enhancin family protein...     176  4e-42

  Bacillus anthracis str. Western North America USA6153 [firmicutes] taxid 212045
 ref|ZP_05193406.1| hypothetical protein BantWNA_11134 [Bac...     319  4e-85
 ref|ZP_05196135.1| metallprotease, enhancin family protein...     176  4e-42

  Bacillus anthracis str. Kruger B [firmicutes] taxid 205919
 ref|ZP_05201895.1| hypothetical protein BantKB_24984 [Baci...     319  4e-85
 ref|ZP_05200087.1| metallprotease, enhancin family protein...     176  4e-42

  Bacillus anthracis str. Vollum [firmicutes] taxid 261591
 ref|ZP_05203030.1| hypothetical protein BantV_00927 [Bacil...     319  4e-85
 ref|ZP_05204784.1| metallprotease, enhancin family protein...     176  4e-42

  Bacillus anthracis str. Australia 94 [firmicutes] taxid 280477
 ref|ZP_05208995.1| hypothetical protein BantA9_01517 [Baci...     319  4e-85
 ref|ZP_05213285.1| metallprotease, enhancin family protein...     176  4e-42

  Bacillus anthracis str. A2012 [firmicutes] taxid 191218
 ref|ZP_00390010.1| hypothetical protein Bant_01000177 [Bac...     318  8e-85
 ref|ZP_00393637.1| hypothetical protein Bant_01004104 [Bac...     176  4e-42

  Bacillus cereus Rock1-3 [firmicutes] taxid 526981
 ref|ZP_04248750.1| S-layer domain protein [Bacillus cereus...     315  7e-84
 ref|ZP_04245415.1| S-layer domain protein [Bacillus cereus...     312  4e-83
 ref|ZP_04247877.1| hypothetical protein bcere0017_47900 [B...     298  1e-78
 ref|ZP_04248460.1| Fibronectin type III domain protein [Ba...     295  5e-78
 ref|ZP_04246240.1| Metallprotease, enhancin [Bacillus cere...     163  3e-38

  Bacillus cytotoxicus NVH 391-98 [firmicutes] taxid 315749
 ref|YP_001376651.1| S-layer domain-containing protein [Bac...     314  1e-83

  Bacillus cereus R309803 [firmicutes] taxid 526968
 ref|ZP_04291969.1| hypothetical protein bcere0009_47940 [B...     313  2e-83

  Bacillus cereus Rock3-29 [firmicutes] taxid 526984
 ref|ZP_04228026.1| S-layer domain protein [Bacillus cereus...     313  3e-83
 ref|ZP_04231342.1| Fibronectin type III domain protein [Ba...     303  3e-80
 ref|ZP_04231422.1| Fibronectin type III domain protein [Ba...     298  1e-78
 ref|ZP_04228801.1| Metallprotease, enhancin [Bacillus cere...     163  3e-38

  Bacillus mycoides DSM 2048 [firmicutes] taxid 526997
 ref|ZP_04168944.1| S-layer domain protein [Bacillus mycoid...     312  4e-83
 ref|ZP_04171806.1| S-layer domain protein [Bacillus mycoid...     308  7e-82

  Bacillus pseudomycoides DSM 12442 [firmicutes] taxid 527000
 ref|ZP_04154891.1| S-layer domain protein [Bacillus pseudo...     306  4e-81
 ref|ZP_04155012.1| Fibronectin type III domain protein [Ba...     294  1e-77

  Bacillus cereus Rock3-28 [firmicutes] taxid 526983
 ref|ZP_04233797.1| S-layer domain protein [Bacillus cereus...     305  8e-81
 ref|ZP_04237054.1| Fibronectin type III domain protein [Ba...     298  1e-78
 ref|ZP_04236724.1| Fibronectin type III domain protein [Ba...     293  2e-77
 ref|ZP_04234601.1| Metallprotease, enhancin [Bacillus cere...     174  1e-41

  Akkermansia muciniphila ATCC BAA-835 [verrucomicrobia] taxid 349741
 ref|YP_001878116.1| hypothetical protein Amuc_1514 [Akkerm...     304  1e-80
 ref|YP_001877245.1| hypothetical protein Amuc_0627 [Akkerm...     206  4e-51
 ref|YP_001878593.1| putative lipoprotein [Akkermansia muci...     204  2e-50
 ref|YP_001877521.1| hypothetical protein Amuc_0908 [Akkerm...     184  1e-44

  Bacillus cereus m1550 [firmicutes] taxid 526969
 ref|ZP_04278345.1| Fibronectin type III domain protein [Ba...     293  2e-77
 ref|ZP_04281429.1| hypothetical protein bcere0011_47810 [B...     120  4e-25

  Bacillus mycoides Rock3-17 [firmicutes] taxid 526999
 ref|ZP_04160383.1| Fibronectin type III domain protein [Ba...     290  2e-76

  Branchiostoma floridae [lancelets] taxid 7739
 ref|XP_002613810.1| hypothetical protein BRAFLDRAFT_85351 ...     290  2e-76
 ref|XP_002610768.1| hypothetical protein BRAFLDRAFT_91560 ...     139  7e-31

  Chitinophaga pinensis DSM 2588 [CFB group bacteria] taxid 485918
 ref|YP_003122217.1| hypothetical protein Cpin_2532 [Chitin...     285  9e-75

  Paenibacillus larvae subsp. larvae BRL-230010 [firmicutes] taxid 392917
 ref|ZP_02327682.1| S-layer domain protein [Paenibacillus l...     283  4e-74
 ref|ZP_02329259.1| enhancin family protein [Paenibacillus ...     123  4e-26
 ref|ZP_02240735.1| enhancin family protein [Paenibacillus ...     103  5e-20

  Pongo abelii (Orang-utan, ...) [primates] taxid 9601
 ref|NP_001126070.1| hypothetical protein LOC100173022 [Pon...     276  3e-72

  Yersinia ruckeri ATCC 29473 [enterobacteria] taxid 527005
 ref|ZP_04617115.1| hypothetical protein yruck0001_340 [Yer...     275  7e-72

  Escherichia coli B7A [enterobacteria] taxid 340184
 ref|ZP_03030866.1| conserved hypothetical protein [Escheri...     274  1e-71

  Escherichia coli O111:H- str. 11128 [enterobacteria] taxid 585396
 ref|YP_003236130.1| putative lipoprotein AcfD homolog prec...     273  3e-71

  Escherichia sp. 1_1_43 [enterobacteria] taxid 457400
 ref|ZP_04872202.1| conserved hypothetical protein [Escheri...     273  4e-71

  Yersinia enterocolitica subsp. enterocolitica 8081 [enterobacteria] taxid 393305
 ref|YP_001007019.1| hypothetical protein YE2830 [Yersinia ...     272  4e-71

  Escherichia coli str. K-12 substr. MG1655 [enterobacteria] taxid 511145
 ref|YP_026189.1| predicted inner membrane lipoprotein [Esc...     272  7e-71

  Escherichia coli str. K-12 substr. W3110 [enterobacteria] taxid 316407
 ref|AP_003528.1| predicted inner membrane lipoprotein [Esc...     272  7e-71

  Escherichia coli BW2952 [enterobacteria] taxid 595496
 ref|YP_002927888.1| putative inner membrane lipoprotein [E...     272  7e-71

  Escherichia coli BL21(DE3) [enterobacteria] taxid 469008
 ref|YP_003035019.1| lipoprotein AcfD-like protein [Escheri...     272  7e-71

  Escherichia coli 53638 [enterobacteria] taxid 344610
 ref|ZP_02998968.1| conserved hypothetical protein [Escheri...     271  9e-71

  Escherichia coli 101-1 [enterobacteria] taxid 358709
 ref|ZP_03068889.1| conserved hypothetical protein [Escheri...     271  1e-70

  Escherichia coli IAI39 [enterobacteria] taxid 585057
 ref|YP_002409370.1| inner membrane lipoprotein [Escherichi...     271  1e-70

  Escherichia coli SE11 [enterobacteria] taxid 409438
 ref|YP_002294524.1| hypothetical protein ECSE_3249 [Escher...     270  2e-70

  Escherichia coli 83972 [enterobacteria] taxid 525281
 ref|ZP_04005476.1| lipoprotein AcfD precursor [Escherichia...     270  2e-70

  Yersinia aldovae ATCC 35236 [enterobacteria] taxid 527002
 ref|ZP_04618439.1| hypothetical protein yaldo0001_11520 [Y...     270  2e-70

  Escherichia coli UMN026 [enterobacteria] taxid 585056
 ref|YP_002414113.1| inner membrane lipoprotein [Escherichi...     270  2e-70

  Escherichia coli E22 [enterobacteria] taxid 340185
 ref|ZP_03043208.1| conserved hypothetical protein [Escheri...     270  3e-70

  Shigella sp. D9 [enterobacteria] taxid 556266
 ref|ZP_05432974.1| hypothetical protein ShiD9_09354 [Shige...     269  4e-70

  Escherichia coli B str. REL606 [enterobacteria] taxid 413997
 ref|YP_003046028.1| predicted inner membrane lipoprotein [...     269  5e-70

  Escherichia coli E110019 [enterobacteria] taxid 340186
 ref|ZP_03048261.1| hypothetical protein EcE110019_1447 [Es...     269  5e-70

  Escherichia coli F11 [enterobacteria] taxid 340197
 ref|ZP_03033388.1| conserved hypothetical protein [Escheri...     268  7e-70

  Escherichia sp. 4_1_40B [enterobacteria] taxid 457401
 ref|ZP_05438313.1| inner membrane lipoprotein [Escherichia...     267  2e-69

  Escherichia coli SMS-3-5 [enterobacteria] taxid 439855
 ref|YP_001745231.1| hypothetical protein EcSMS35_3251 [Esc...     267  2e-69

  Escherichia coli HS [enterobacteria] taxid 331112
 ref|YP_001459760.1| hypothetical protein EcHS_A3142 [Esche...     267  2e-69

  Entamoeba dispar SAW760 [eukaryotes] taxid 370354
 ref|XP_001737701.1| antigenic protein NP1 [Entamoeba dispa...     266  3e-69

  Escherichia coli 536 [enterobacteria] taxid 362663
 ref|YP_670934.1| putative lipoprotein AcfD precursor [Esch...     266  3e-69

  Entamoeba histolytica HM-1:IMSS [eukaryotes] taxid 294381
 ref|XP_654508.1| immuno-dominant variable surface antigen ...     266  4e-69

  Escherichia coli E24377A [enterobacteria] taxid 331111
 ref|YP_001464429.1| hypothetical protein EcE24377A_3432 [E...     265  9e-69

  Escherichia coli O127:H6 str. E2348/69 [enterobacteria] taxid 574521
 ref|YP_002330723.1| predicted inner membrane lipoprotein [...     264  1e-68

  Yersinia mollaretii ATCC 43969 [enterobacteria] taxid 349967
 ref|ZP_04639817.1| hypothetical protein ymoll0001_21340 [Y...     264  1e-68

  Escherichia coli 55989 [enterobacteria] taxid 585055
 ref|YP_002404344.1| inner membrane lipoprotein [Escherichi...     263  2e-68

  Escherichia coli S88 [enterobacteria] taxid 585035
 ref|YP_002392955.1| inner membrane lipoprotein [Escherichi...     263  4e-68

  Escherichia coli UTI89 [enterobacteria] taxid 364106
 ref|YP_542367.1| putative lipoprotein AcfD-like precursor ...     262  5e-68

  Escherichia coli APEC O1 [enterobacteria] taxid 405955
 ref|YP_854419.1| putative lipoprotein AcfD-like precursor ...     262  5e-68

  Escherichia sp. 3_2_53FAA [enterobacteria] taxid 469598
 ref|ZP_04536768.1| conserved hypothetical protein [Escheri...     262  5e-68

  Escherichia coli ED1a [enterobacteria] taxid 585397
 ref|YP_002399471.1| inner membrane lipoprotein [Escherichi...     261  7e-68

  Escherichia albertii TW07627 [enterobacteria] taxid 502347
 ref|ZP_02900937.1| AcfD [Escherichia albertii TW07627]            261  1e-67

  Escherichia coli IAI1 [enterobacteria] taxid 585034
 ref|YP_002388453.1| inner membrane lipoprotein [Escherichi...     261  1e-67

  Escherichia fergusonii ATCC 35469 [enterobacteria] taxid 585054
 ref|YP_002384010.1| inner membrane lipoprotein [Escherichi...     261  1e-67

  Bacteroides thetaiotaomicron VPI-5482 [CFB group bacteria] taxid 226186
 ref|NP_813155.1| hypothetical protein BT_4244 [Bacteroides...     250  2e-64
 ref|NP_811927.1| hypothetical protein BT_3015 [Bacteroides...     231  9e-59
 ref|NP_809190.1| hypothetical protein BT_0277 [Bacteroides...     151  2e-34
 ref|NP_813183.1| hypothetical protein BT_4272 [Bacteroides...     147  2e-33

  Bacteroides sp. 1_1_6 [CFB group bacteria] taxid 469586
 ref|ZP_04847386.1| conserved hypothetical protein [Bactero...     250  2e-64
 ref|ZP_04846622.1| conserved hypothetical protein [Bactero...     233  2e-59
 ref|ZP_04845085.1| coagulation factor 5/8 type [Bacteroide...     151  2e-34
 ref|ZP_04847358.1| conserved hypothetical protein [Bactero...     147  2e-33

  Bacteroides sp. 3_2_5 [CFB group bacteria] taxid 457392
 ref|ZP_04842342.1| conserved hypothetical protein [Bactero...     249  4e-64

  Bacteroides caccae ATCC 43185 [CFB group bacteria] taxid 411901
 ref|ZP_01959759.1| hypothetical protein BACCAC_01368 [Bact...     249  4e-64
 ref|ZP_01961267.1| hypothetical protein BACCAC_02897 [Bact...     249  5e-64
 ref|ZP_01960229.1| hypothetical protein BACCAC_01841 [Bact...     245  6e-63
 ref|ZP_01960228.1| hypothetical protein BACCAC_01840 [Bact...     245  1e-62
 ref|ZP_01960238.1| hypothetical protein BACCAC_01850 [Bact...     241  1e-61
 ref|ZP_01960247.1| hypothetical protein BACCAC_01859 [Bact...     240  2e-61
 ref|ZP_01959885.1| hypothetical protein BACCAC_01495 [Bact...     236  3e-60
 ref|ZP_01960954.1| hypothetical protein BACCAC_02574 [Bact...     236  5e-60
 ref|ZP_01961832.1| hypothetical protein BACCAC_03474 [Bact...     235  7e-60
 ref|ZP_01959638.1| hypothetical protein BACCAC_01246 [Bact...     228  1e-57
 ref|ZP_01959886.1| hypothetical protein BACCAC_01496 [Bact...     213  2e-53
 ref|ZP_01961967.1| hypothetical protein BACCAC_03612 [Bact...     205  8e-51
 ref|ZP_01960170.1| hypothetical protein BACCAC_01782 [Bact...     188  2e-45
 ref|ZP_01959767.1| hypothetical protein BACCAC_01376 [Bact...     183  3e-44
 ref|ZP_01959768.1| hypothetical protein BACCAC_01377 [Bact...     143  3e-32
 ref|ZP_01958589.1| hypothetical protein BACCAC_00161 [Bact...     131  1e-28

  Bacteroides fragilis YCH46 [CFB group bacteria] taxid 295405
 ref|YP_100380.1| hypothetical protein BF3101 [Bacteroides ...     248  6e-64

  Bacteroides fragilis NCTC 9343 [CFB group bacteria] taxid 272559
 ref|YP_212553.1| putative lipoprotein [Bacteroides fragili...     248  6e-64

  Bacteroides sp. 2_1_16 [CFB group bacteria] taxid 469587
 ref|ZP_06094201.1| conserved hypothetical protein [Bactero...     248  6e-64

  Bacteroides plebeius DSM 17135 [CFB group bacteria] taxid 484018
 ref|ZP_03207951.1| hypothetical protein BACPLE_01583 [Bact...     245  6e-63
 ref|ZP_03208622.1| hypothetical protein BACPLE_02278 [Bact...     202  7e-50

  Bacteroides fragilis 3_1_12 [CFB group bacteria] taxid 457424
 ref|ZP_05282640.1| putative lipoprotein [Bacteroides fragi...     244  2e-62

  Pseudomonas syringae pv. oryzae str. 1_6 [g-proteobacteria] taxid 563797
 ref|ZP_04589884.1| hypothetical protein Psyrpo1_21516 [Pse...     244  2e-62

  Vibrio mimicus VM223 [g-proteobacteria] taxid 675820
 ref|ZP_06033510.1| accessory colonization factor AcfD prec...     242  6e-62

  Vibrio mimicus MB-451 [g-proteobacteria] taxid 675806
 ref|ZP_06039074.1| accessory colonization factor AcfD prec...     240  2e-61

  Vibrio mimicus VM573 [g-proteobacteria] taxid 671076
 ref|ZP_05718328.1| accessory colonization factor acfD [Vib...     240  4e-61
 ref|ZP_05715067.1| Accessory colonization factor AcfD [Vib...     233  3e-59

  Photorhabdus asymbiotica [enterobacteria] taxid 291112
 ref|YP_003041170.1| hypothetical protein PAU_02334 [Photor...     238  1e-60

  Vibrio sp. RC586 [g-proteobacteria] taxid 675815
 ref|ZP_06078833.1| accessory colonization factor AcfD prec...     238  1e-60

  Vibrio parahaemolyticus RIMD 2210633 [g-proteobacteria] taxid 223926
 ref|NP_800886.1| hypothetical protein VPA1376 [Vibrio para...     238  1e-60
 ref|NP_797381.1| hypothetical protein VP1002 [Vibrio parah...     203  4e-50

  Vibrio parahaemolyticus K5030 [g-proteobacteria] taxid 627611
 ref|ZP_05775657.1| hypothetical protein VparK_06627 [Vibri...     238  1e-60
 ref|ZP_05776554.1| hypothetical protein VparK_11235 [Vibri...     203  4e-50

  Vibrio parahaemolyticus AN-5034 [g-proteobacteria] taxid 563773
 ref|ZP_05892431.1| hypothetical protein VparAN_19891 [Vibr...     238  1e-60

  Vibrio parahaemolyticus Peru-466 [g-proteobacteria] taxid 563771
 ref|ZP_05903689.1| hypothetical protein VparP_03774 [Vibri...     238  1e-60
 ref|ZP_05906413.1| hypothetical protein VparP_17696 [Vibri...     203  4e-50

  Vibrio cholerae bv. albensis VL426 [g-proteobacteria] taxid 593585
 ref|ZP_04413087.1| accessory colonization factor AcfD prec...     238  1e-60

  Vibrio cholerae RC385 [g-proteobacteria] taxid 345074
 ref|ZP_04916523.1| Large exoproteins involved in heme util...     237  2e-60

  Vibrio vulnificus CMCP6 [g-proteobacteria] taxid 216895
 ref|NP_759746.1| hypothetical protein VV1_0767 [Vibrio vul...     236  5e-60
 ref|NP_760966.1| hypothetical protein VV1_2105 [Vibrio vul...     185  9e-45

  Grimontia hollisae CIP 101886 [g-proteobacteria] taxid 675812
 ref|ZP_06051991.1| accessory colonization factor AcfD prec...     235  5e-60
 ref|ZP_06051982.1| hypothetical protein VHA_001146 [Grimon...     158  1e-36

  Vibrio cholerae O1 biovar El Tor str. N16961 [g-proteobacteria] taxid 243277
 ref|NP_230493.1| hypothetical protein VC0845 [Vibrio chole...     235  6e-60

  Vibrio cholerae 2740-80 [g-proteobacteria] taxid 412614
 ref|ZP_01675490.1| lipoprotein, putative [Vibrio cholerae ...     235  6e-60

  Vibrio cholerae V52 [g-proteobacteria] taxid 345076
 ref|ZP_01679355.1| lipoprotein, putative [Vibrio cholerae ...     235  6e-60

  Vibrio cholerae NCTC 8457 [g-proteobacteria] taxid 417399
 ref|ZP_01970715.1| lipoprotein, putative [Vibrio cholerae ...     235  6e-60

  Vibrio cholerae B33 [g-proteobacteria] taxid 417400
 ref|ZP_01974705.1| lipoprotein, putative [Vibrio cholerae ...     235  6e-60
 ref|ZP_04400263.1| accessory colonization factor AcfD prec...     235  6e-60

  Vibrio cholerae M66-2 [g-proteobacteria] taxid 579112
 ref|YP_002809573.1| putative lipoprotein [Vibrio cholerae ...     235  6e-60

  Vibrio cholerae BX 330286 [g-proteobacteria] taxid 593587
 ref|ZP_04395055.1| accessory colonization factor AcfD prec...     235  6e-60

  Vibrio cholerae RC9 [g-proteobacteria] taxid 593589
 ref|ZP_04407349.1| accessory colonization factor AcfD prec...     235  6e-60

  Vibrio cholerae MJ-1236 [g-proteobacteria] taxid 593588
 ref|YP_002879212.1| accessory colonization factor AcfD pre...     235  6e-60

  Vibrio cholerae MO10 [g-proteobacteria] taxid 345072
 ref|ZP_05237331.1| accessory colonization factor AcfD [Vib...     235  6e-60

  Vibrio cholera CIRS 101 [g-proteobacteria] taxid 661513
 ref|ZP_05418596.1| accessory colonization factor AcfD prec...     235  6e-60

  Vibrio cholerae INDRE 91/1 [g-proteobacteria] taxid 675808
 ref|ZP_06030335.1| accessory colonization factor AcfD prec...     235  6e-60

  Vibrio cholerae V51 [g-proteobacteria] taxid 345075
 ref|ZP_04918705.1| lipoprotein, putative [Vibrio cholerae ...     235  9e-60
 ref|ZP_04918555.1| Large exoproteins involved in heme util...     233  2e-59

  Vibrio cholerae TMA 21 [g-proteobacteria] taxid 593590
 ref|ZP_04404639.1| accessory colonization factor AcfD prec...     234  1e-59

  Vibrio parahaemolyticus AQ3810 [g-proteobacteria] taxid 419109
 ref|ZP_01988669.1| AcfD [Vibrio parahaemolyticus AQ3810]          234  1e-59
 ref|ZP_01990000.1| conserved hypothetical protein [Vibrio ...     208  1e-51

  Vibrio cholerae AM-19226 [g-proteobacteria] taxid 404974
 ref|ZP_04960229.1| Large exoproteins involved in heme util...     234  2e-59

  Vibrio cholerae O395 [g-proteobacteria] taxid 345073
 ref|YP_001216326.1| putative lipoprotein [Vibrio cholerae ...     233  2e-59

  Vibrio cholerae 1587 [g-proteobacteria] taxid 412966
 ref|ZP_01949281.1| accessory colonization factor AcfD [Vib...     233  2e-59

  Vibrio cholerae 623-39 [g-proteobacteria] taxid 417397
 ref|ZP_01981213.1| AcfD [Vibrio cholerae 623-39]                  233  2e-59

  Pseudomonas syringae pv. syringae B728a [g-proteobacteria] taxid 205918
 ref|YP_237316.1| hypothetical protein Psyr_4248 [Pseudomon...     233  3e-59
 ref|YP_237954.1| hypothetical protein Psyr_4889 [Pseudomon...     203  3e-50

  Bacteroides vulgatus ATCC 8482 [CFB group bacteria] taxid 435590
 ref|YP_001298266.1| hypothetical protein BVU_0949 [Bactero...     233  3e-59

  Vibrio cholerae MAK 757 [g-proteobacteria] taxid 412967
 ref|ZP_01954520.1| accessory colonization factor AcfD [Vib...     231  8e-59

  Aspergillus flavus NRRL3357 [ascomycetes] taxid 332952
 ref|XP_002377568.1| cell wall associated protein, putative...     231  1e-58
 ref|XP_002372411.1| viral-enhancing factor, putative [Aspe...     143  4e-32

  Clostridium perfringens E str. JGS1987 [firmicutes] taxid 451755
 ref|ZP_02631053.1| discoidin domain protein [Clostridium p...     230  2e-58

  Sphingobacterium spiritivorum ATCC 33861 [CFB group bacteria] taxid 525373
 ref|ZP_04779511.1| conserved hypothetical protein [Sphingo...     230  3e-58
 ref|ZP_04781290.1| possible wall-associated protein [Sphin...     213  2e-53
 ref|ZP_04781251.1| conserved hypothetical protein [Sphingo...     208  9e-52
 ref|ZP_04778523.1| conserved hypothetical protein [Sphingo...     202  6e-50
 ref|ZP_04777486.1| lipoprotein [Sphingobacterium spiritivo...     201  1e-49
 ref|ZP_04780830.1| possible wall-associated protein [Sphin...     183  3e-44

  Shewanella halifaxensis HAW-EB4 [g-proteobacteria] taxid 458817
 ref|YP_001675698.1| hypothetical protein Shal_3494 [Shewan...     230  4e-58
 ref|YP_001673185.1| hypothetical protein Shal_0951 [Shewan...     179  3e-43

  Sphingobacterium spiritivorum ATCC 33300 [CFB group bacteria] taxid 525372
 ref|ZP_03970216.1| conserved hypothetical protein [Sphingo...     228  1e-57
 ref|ZP_03969571.1| possible wall-associated protein [Sphin...     210  3e-52
 ref|ZP_03969516.1| conserved hypothetical protein [Sphingo...     207  2e-51
 ref|ZP_03967680.1| lipoprotein [Sphingobacterium spiritivo...     204  2e-50
 ref|ZP_03968927.1| conserved hypothetical protein [Sphingo...     201  1e-49
 ref|ZP_03966994.1| possible wall-associated protein [Sphin...     183  3e-44

  Aspergillus oryzae RIB40 [ascomycetes] taxid 510516
 ref|XP_001825930.1| hypothetical protein [Aspergillus oryz...     227  2e-57
 ref|XP_001817345.1| hypothetical protein [Aspergillus oryz...     143  3e-32

  Clostridium perfringens D str. JGS1721 [firmicutes] taxid 488537
 ref|ZP_02953684.1| discoidin domain protein [Clostridium p...     226  3e-57
 ref|ZP_02954286.1| putative antigenic protein NP1 [Clostri...     221  1e-55
 ref|ZP_02951972.1| F5/8 type C domain protein [Clostridium...     198  1e-48
 ref|ZP_02953260.1| discoidin domain protein [Clostridium p...     174  2e-41
 ref|ZP_02954276.1| LRR adjacent family [Clostridium perfri...     166  5e-39
 ref|ZP_02954459.1| putative enhancin family protein [Clost...     159  7e-37

  Clostridium perfringens B str. ATCC 3626 [firmicutes] taxid 451754
 ref|ZP_02637411.1| discoidin domain protein [Clostridium p...     226  4e-57
 ref|ZP_02635227.1| F5/8 type C domain protein [Clostridium...     197  2e-48

  Clostridium perfringens CPE str. F4969 [firmicutes] taxid 451756
 ref|ZP_02637953.1| discoidin domain protein [Clostridium p...     226  4e-57
 ref|ZP_02638647.1| F5/8 type C domain protein [Clostridium...     194  1e-47

  Pseudomonas syringae pv. tomato str. DC3000 [g-proteobacteria] taxid 223283
 ref|NP_794325.2| hypothetical protein PSPTO_4574 [Pseudomo...     226  4e-57
 ref|NP_795062.1| hypothetical protein PSPTO_5331 [Pseudomo...     191  1e-46

  Vibrio cholerae MZO-3 [g-proteobacteria] taxid 412883
 ref|ZP_01955015.1| accessory colonization factor AcfD [Vib...     225  1e-56
 ref|ZP_01955094.1| conserved hypothetical protein [Vibrio ...     150  3e-34

  Vibrio parahaemolyticus AQ4037 [g-proteobacteria] taxid 563772
 ref|ZP_05908271.1| inner membrane lipoprotein [Vibrio para...     221  2e-55
 ref|ZP_05911847.1| hypothetical protein VparAQ_22344 [Vibr...     204  2e-50

  Listeria grayi DSM 20601 [firmicutes] taxid 525367
 ref|ZP_04443744.1| possible wall-associated protein precur...     218  8e-55
 ref|ZP_04443281.1| enhancin family protein [Listeria grayi...     111  2e-22

  Vibrio harveyi 1DA3 [g-proteobacteria] taxid 673519
 ref|ZP_06177065.1| hypothetical protein VME_34490 [Vibrio ...     217  2e-54

  Vibrio sp. Ex25 [g-proteobacteria] taxid 150340
 ref|YP_003286593.1| hypothetical protein VEA_003968 [Vibri...     217  2e-54
 ref|ZP_04922420.1| conserved hypothetical protein [Vibrio ...     212  5e-53

  Clostridium perfringens C str. JGS1495 [firmicutes] taxid 445334
 ref|ZP_02865871.1| ABC transporter, permease protein [Clos...     216  4e-54

  Brachybacterium faecium DSM 4810 [high GC Gram+] taxid 446465
 ref|YP_003154412.1| hypothetical protein Bfae_09730 [Brach...     216  5e-54

  Vibrio harveyi HY01 [g-proteobacteria] taxid 410291
 ref|ZP_01985049.1| AcfD [Vibrio harveyi HY01]                     216  5e-54

  Shewanella pealeana ATCC 700345 [g-proteobacteria] taxid 398579
 ref|YP_001502491.1| inner membrane lipoprotein [Shewanella...     214  1e-53
 ref|YP_001500761.1| hypothetical protein Spea_0899 [Shewan...     183  4e-44

  Vibrio alginolyticus 12G01 [g-proteobacteria] taxid 314288
 ref|ZP_01260413.1| hypothetical protein V12G01_20798 [Vibr...     214  1e-53

  Vibrio alginolyticus 40B [g-proteobacteria] taxid 674977
 ref|ZP_06181693.1| hypothetical protein VMC_31230 [Vibrio ...     213  2e-53

  Vibrio fischeri MJ11 [g-proteobacteria] taxid 388396
 ref|YP_002155459.1| inner membrane lipoprotein [Vibrio fis...     208  1e-51

  Clostridium botulinum E3 str. Alaska E43 [firmicutes] taxid 508767
 ref|YP_001920091.1| fibronectin type III domain protein [C...     208  1e-51

  Photobacterium damselae subsp. damselae CIP 102761 [g-proteobacteria] taxid 675817
 ref|ZP_06155178.1| accessory colonization factor AcfD prec...     207  2e-51

  Cryptosporidium muris RN66 [apicomplexans] taxid 441375
 ref|XP_002139263.1| hypothetical protein [Cryptosporidium ...     207  3e-51

  Clostridium botulinum E1 str. 'BoNT E Beluga' [firmicutes] taxid 536233
 ref|ZP_04823618.1| fibronectin type III domain protein [Cl...     204  1e-50

  Clostridium perfringens SM101 [firmicutes] taxid 289380
 ref|YP_698600.1| leucine rich repeat domain-containing pro...     201  2e-49

  Clostridium bartlettii DSM 16795 [firmicutes] taxid 445973
 ref|ZP_02211509.1| hypothetical protein CLOBAR_01122 [Clos...     200  3e-49
 ref|ZP_02211508.1| hypothetical protein CLOBAR_01121 [Clos...     190  2e-46

  Vibrio orientalis CIP 102891 [g-proteobacteria] taxid 675816
 ref|ZP_05945865.1| hypothetical protein VIA_003317 [Vibrio...     199  6e-49

  Clostridium perfringens ATCC 13124 [firmicutes] taxid 195103
 ref|YP_695932.1| F5/8 type C domain-containing protein [Cl...     198  8e-49
 ref|YP_695519.1| discoidin domain-containing protein [Clos...     176  6e-42

  Clostridium perfringens str. 13 [firmicutes] taxid 195102
 ref|NP_562197.1| hypothetical protein CPE1281 [Clostridium...     198  1e-48

  Clostridium perfringens NCTC 8239 [firmicutes] taxid 451757
 ref|ZP_02642699.1| F5/8 type C domain protein [Clostridium...     197  3e-48
 ref|ZP_02641714.1| discoidin domain protein [Clostridium p...     178  1e-42

  Vibrio parahaemolyticus 16 [g-proteobacteria] taxid 391586
 ref|ZP_05120656.1| hypothetical protein VPMS16_1840 [Vibri...     195  7e-48

  Vibrio sp. MED222 [g-proteobacteria] taxid 314290
 ref|ZP_01064757.1| hypothetical protein MED222_12503 [Vibr...     193  4e-47

  Vibrio mimicus VM603 [g-proteobacteria] taxid 671074
 ref|ZP_05722171.1| conserved hypothetical protein [Vibrio ...     193  4e-47

  Mycoplasma penetrans HF-2 [mycoplasmas] taxid 272633
 ref|NP_757708.1| integral membrane protein [Mycoplasma pen...     193  4e-47
 ref|NP_757816.1| hypothetical protein MYPE4300 [Mycoplasma...     126  4e-27
 ref|NP_757817.1| putative integral membrane protein [Mycop...      99  9e-19

  Pseudomonas syringae pv. tomato T1 [g-proteobacteria] taxid 546231
 ref|ZP_03399297.1| hypothetical protein PSPTOT1_2511 [Pseu...     191  1e-46

  Vibrio splendidus LGP32 [g-proteobacteria] taxid 575788
 ref|YP_002416673.1| hypothetical protein VS_1058 [Vibrio s...     191  2e-46

  Bacteroides coprophilus DSM 18228 [CFB group bacteria] taxid 547042
 ref|ZP_03642641.1| hypothetical protein BACCOPRO_00998 [Ba...     189  4e-46

  Subdoligranulum variabile DSM 15176 [firmicutes] taxid 411471
 ref|ZP_05981011.2| putative fibronectin type III domain pr...     188  1e-45

  Vibrio vulnificus YJ016 [g-proteobacteria] taxid 196600
 ref|NP_935128.1| hypothetical protein VV2335 [Vibrio vulni...     186  4e-45

  Vibrionales bacterium SWAT-3 [g-proteobacteria] taxid 391574
 ref|ZP_01814153.1| hypothetical protein VSWAT3_23194 [Vibr...     182  6e-44

  Shewanella woodyi ATCC 51908 [g-proteobacteria] taxid 392500
 ref|YP_001759442.1| hypothetical protein Swoo_1054 [Shewan...     181  1e-43

  Eubacterium dolichum DSM 3991 [firmicutes] taxid 428127
 ref|ZP_02076735.1| hypothetical protein EUBDOL_00526 [Euba...     180  3e-43

  Vibrio fischeri ES114 [g-proteobacteria] taxid 312309
 ref|YP_204079.1| accessory colonization factor AcfD-like p...     179  5e-43

  Bacillus anthracis str. A0488 [firmicutes] taxid 486624
 ref|ZP_02213488.1| metallprotease, enhancin family [Bacill...     176  4e-42

  Bacillus anthracis str. A0193 [firmicutes] taxid 486619
 ref|ZP_02395688.1| metallprotease, enhancin family [Bacill...     176  4e-42

  Bacillus anthracis str. A0389 [firmicutes] taxid 486623
 ref|ZP_02895627.1| metallprotease, enhancin family [Bacill...     176  4e-42

  Bacillus anthracis str. A0174 [firmicutes] taxid 486622
 ref|ZP_02933074.1| metallprotease, enhancin family [Bacill...     176  4e-42

  Bacillus anthracis Tsiankovskii-I [firmicutes] taxid 405536
 ref|ZP_03017932.1| metallprotease, enhancin family [Bacill...     176  4e-42

  Bacillus anthracis str. A0248 [firmicutes] taxid 592021
 ref|YP_002867605.1| metallprotease, enhancin family [Bacil...     176  4e-42

  Bacillus anthracis str. A0442 [firmicutes] taxid 486621
 ref|ZP_02390091.1| metallprotease, enhancin family [Bacill...     176  4e-42

  Bacillus anthracis str. A0465 [firmicutes] taxid 486620
 ref|ZP_02876223.1| metallprotease, enhancin family [Bacill...     176  4e-42

  Clostridium sp. 7_2_43FAA [firmicutes] taxid 457396
 ref|ZP_05129649.1| leucine rich repeat domain-containing p...     176  6e-42

  Shewanella loihica PV-4 [g-proteobacteria] taxid 323850
 ref|YP_001093051.1| hypothetical protein Shew_0921 [Shewan...     175  1e-41

  Prevotella melaninogenica ATCC 25845 [CFB group bacteria] taxid 553174
 ref|ZP_04834099.1| conserved hypothetical protein [Prevote...     173  3e-41

  Clostridium hathewayi DSM 13479 [firmicutes] taxid 566550
 ref|ZP_06117837.1| coagulation factor 5/8 type domain prot...     172  7e-41

  Pseudomonas aeruginosa PA7 [g-proteobacteria] taxid 381754
 ref|YP_001346070.1| hypothetical protein PSPA7_0675 [Pseud...     170  3e-40

  Clostridium difficile QCD-66c26 [firmicutes] taxid 455631
 ref|ZP_05272863.1| hypothetical protein CdifQC_13801 [Clos...     169  7e-40

  Clostridium difficile CIP 107932 [firmicutes] taxid 499176
 ref|ZP_05323255.1| hypothetical protein CdifC_14116 [Clost...     169  7e-40

  Clostridium difficile QCD-76w55 [firmicutes] taxid 479832
 ref|ZP_05357111.1| hypothetical protein CdifQCD-7_14289 [C...     169  7e-40

  Clostridium difficile QCD-97b34 [firmicutes] taxid 479833
 ref|ZP_05385867.1| hypothetical protein CdifQCD-_13828 [Cl...     169  7e-40

  Clostridium difficile QCD-37x79 [firmicutes] taxid 479834
 ref|ZP_05398209.1| hypothetical protein CdifQCD_14063 [Clo...     169  7e-40

  Clostridium difficile CD196 [firmicutes] taxid 645462
 ref|YP_003215657.1| hypothetical protein CD196_2639 [Clost...     169  7e-40

  Clostridium difficile R20291 [firmicutes] taxid 645463
 ref|YP_003219165.1| hypothetical protein CDR20291_2686 [Cl...     169  7e-40

  Pseudomonas aeruginosa PACS2 [g-proteobacteria] taxid 388272
 ref|ZP_01363476.1| hypothetical protein PaerPA_01000570 [P...     167  2e-39

  Pseudomonas aeruginosa 2192 [g-proteobacteria] taxid 350703
 ref|ZP_04936986.1| hypothetical protein PA2G_04490 [Pseudo...     166  6e-39

  Pseudomonas aeruginosa C3719 [g-proteobacteria] taxid 350704
 ref|ZP_04930535.1| hypothetical protein PACG_03268 [Pseudo...     166  6e-39

  Pseudomonas aeruginosa PAO1 [g-proteobacteria] taxid 208964
 ref|NP_249263.1| hypothetical protein PA0572 [Pseudomonas ...     166  6e-39

  Pseudomonas aeruginosa LESB58 [g-proteobacteria] taxid 557722
 ref|YP_002438177.1| hypothetical protein PLES_05691 [Pseud...     166  6e-39

  Pseudomonas aeruginosa UCBPP-PA14 [g-proteobacteria] taxid 208963
 ref|YP_788743.1| hypothetical protein PA14_07430 [Pseudomo...     166  6e-39

  Clostridium difficile QCD-63q42 [firmicutes] taxid 479831
 ref|ZP_05330944.1| hypothetical protein CdifQCD-6_14221 [C...     164  1e-38

  Clostridium difficile ATCC 43255 [firmicutes] taxid 499175
 ref|ZP_05352006.1| hypothetical protein CdifA_14696 [Clost...     164  2e-38

  Clostridium ramosum DSM 1402 [firmicutes] taxid 445974
 ref|ZP_02426874.1| hypothetical protein CLORAM_00251 [Clos...     164  2e-38

  Clostridium difficile 630 [firmicutes] taxid 272563
 ref|YP_001089310.1| hypothetical protein CD2797 [Clostridi...     163  3e-38

  Clostridium hiranonis DSM 13275 [firmicutes] taxid 500633
 ref|ZP_03292590.1| hypothetical protein CLOHIR_00533 [Clos...     163  3e-38

  Mollicutes bacterium D7 [mycoplasmas] taxid 556270
 ref|ZP_04563254.1| conserved hypothetical protein [Mollicu...     163  4e-38

  Monosiga brevicollis MX1 [choanoflagellates] taxid 431895
 ref|XP_001750305.1| hypothetical protein [Monosiga brevico...     161  2e-37

  Shewanella oneidensis MR-1 [g-proteobacteria] taxid 211586
 ref|NP_716077.1| hypothetical protein SO_0440 [Shewanella ...     158  1e-36

  Bacteroides capillosus ATCC 29799 [CFB group bacteria] taxid 411467
 ref|ZP_02035877.1| hypothetical protein BACCAP_01474 [Bact...     158  1e-36

  Hahella chejuensis KCTC 2396 [g-proteobacteria] taxid 349521
 ref|YP_434232.1| hypothetical protein HCH_03041 [Hahella c...     157  2e-36

  Eggerthella lenta DSM 2243 [high GC Gram+] taxid 479437
 ref|YP_003182171.1| coagulation factor 5/8 type domain pro...     154  2e-35

  Shewanella sp. MR-7 [g-proteobacteria] taxid 60481
 ref|YP_739623.1| hypothetical protein Shewmr7_3586 [Shewan...     154  2e-35

  Clostridium botulinum A2 str. Kyoto [firmicutes] taxid 536232
 ref|YP_002804477.1| viral enhancin protein [Clostridium bo...     154  3e-35

  Shewanella sp. ANA-3 [g-proteobacteria] taxid 94122
 ref|YP_868088.1| hypothetical protein Shewana3_0439 [Shewa...     152  7e-35

  Clostridium botulinum F str. Langeland [firmicutes] taxid 441772
 ref|YP_001391397.1| enhancing factor [Clostridium botulinu...     150  3e-34

  Clostridium botulinum Bf [firmicutes] taxid 445336
 ref|ZP_02618432.1| enhancing factor [Clostridium botulinum...     148  1e-33

  Clostridium botulinum Ba4 str. 657 [firmicutes] taxid 515621
 ref|YP_002863079.1| enhancing factor [Clostridium botulinu...     147  2e-33

  Salmonella enterica subsp. arizonae serovar 62:z4,z23:-- [enterobacteria] taxid 41514
 ref|YP_001573177.1| hypothetical protein SARI_04252 [Salmo...     147  2e-33

  Clostridium botulinum B1 str. Okra [firmicutes] taxid 498213
 ref|YP_001781652.1| enhancing factor [Clostridium botulinu...     147  2e-33

  Bacteroides finegoldii DSM 17565 [CFB group bacteria] taxid 483215
 ref|ZP_05415629.1| conserved hypothetical protein [Bactero...     147  2e-33

  Shewanella sp. MR-4 [g-proteobacteria] taxid 60480
 ref|YP_732580.1| hypothetical protein Shewmr4_0443 [Shewan...     146  4e-33

  Cryptosporidium parvum Iowa II [apicomplexans] taxid 353152
 ref|XP_001388270.1| hypothetical protein [Cryptosporidium ...     143  3e-32

  Bacteroides sp. D2 [CFB group bacteria] taxid 556259
 ref|ZP_05757374.1| hypothetical protein BacD2_03774 [Bacte...     141  2e-31

  Shewanella baltica OS185 [g-proteobacteria] taxid 402882
 ref|YP_001368103.1| hypothetical protein Shew185_3916 [She...     141  2e-31

  Shewanella amazonensis SB2B [g-proteobacteria] taxid 326297
 ref|YP_926273.1| hypothetical protein Sama_0393 [Shewanell...     140  3e-31

  Shewanella baltica OS195 [g-proteobacteria] taxid 399599
 ref|YP_001556458.1| hypothetical protein Sbal195_4038 [She...     140  3e-31

  Uncinocarpus reesii 1704 [ascomycetes] taxid 336963
 ref|XP_002544486.1| hypothetical protein UREG_04003 [Uncin...     139  6e-31

  Shewanella baltica OS155 [g-proteobacteria] taxid 325240
 ref|YP_001048819.1| hypothetical protein Sbal_0418 [Shewan...     137  2e-30

  Shewanella baltica OS223 [g-proteobacteria] taxid 407976
 ref|YP_002359742.1| hypothetical protein Sbal223_3842 [She...     137  2e-30

  Pantoea sp. At-9b [enterobacteria] taxid 592316
 ref|ZP_05730303.1| hypothetical protein Pat9bDRAFT_3619 [P...     134  2e-29

  Clostridium difficile QCD-32g58 [firmicutes] taxid 367459
 ref|ZP_01802287.1| hypothetical protein CdifQ_04003262 [Cl...     124  3e-26
 ref|ZP_01802288.1| hypothetical protein CdifQ_04003263 [Cl...      52  8e-05

  Salmonella enterica subsp. enterica serovar Dublin str. CT_02021853 [enterobacteria] taxid 439851
 ref|YP_002214171.1| viral enhancin protein [Salmonella ent...     119  5e-25

  Salmonella enterica subsp. enterica serovar Enteritidis str. P125109 [enterobacteria] taxid 550537
 ref|YP_002242378.1| putative viral enhancing factor [Salmo...     119  6e-25

  Salmonella enterica subsp. enterica serovar Schwarzengrund str. SL480 [enterobacteria] taxid 454165
 ref|ZP_02660045.1| viral enhancin protein [Salmonella ente...     119  8e-25

  Salmonella enterica subsp. enterica serovar Schwarzengrund str. CVM19633 [enterobacteria] taxid 439843
 ref|YP_002113233.1| viral enhancin protein [Salmonella ent...     119  8e-25

  Salmonella enterica subsp. enterica serovar Javiana str. GA_MM04042433 [enterobacteria] taxid 454167
 ref|ZP_03218530.1| viral enhancin protein [Salmonella ente...     118  1e-24

  Yersinia pseudotuberculosis IP 31758 [enterobacteria] taxid 349747
 ref|YP_001402703.1| viral enhancin protein [Yersinia pseud...     116  5e-24

  Yersinia pseudotuberculosis IP 32953 [enterobacteria] taxid 273123
 ref|YP_068937.1| enhancing factor [Yersinia pseudotubercul...     116  5e-24

  Yersinia pseudotuberculosis YPIII [enterobacteria] taxid 502800
 ref|YP_001722552.1| peptidase M60 viral enhancin protein [...     116  5e-24

  Yersinia pseudotuberculosis PB1/+ [enterobacteria] taxid 502801
 ref|YP_001870865.1| peptidase M60 viral enhancin protein [...     116  5e-24

  Clostridium butyricum 5521 [firmicutes] taxid 447214
 ref|ZP_02950852.1| putative exported protein [Clostridium ...     114  2e-23

  Clostridium butyricum E4 str. BoNT E BL5262 [firmicutes] taxid 632245
 ref|ZP_04526007.1| fibronectin type III domain protein [Cl...     114  2e-23

  Yersinia pestis KIM 10 [enterobacteria] taxid 187410
 ref|NP_667934.1| enhancing factor [Yersinia pestis KIM]           111  1e-22

  Yersinia pestis biovar Microtus str. 91001 [enterobacteria] taxid 229193
 ref|NP_991886.1| enhancing factor [Yersinia pestis biovar ...     111  1e-22

  Yersinia pestis CO92 [enterobacteria] taxid 214092
 ref|YP_002345418.1| enhancing factor (viral) [Yersinia pes...     111  1e-22

  Yersinia pestis Antiqua [enterobacteria] taxid 360102
 ref|YP_653848.1| enhancing factor [Yersinia pestis Antiqua]       111  1e-22

  Yersinia pestis Nepal516 [enterobacteria] taxid 377628
 ref|YP_649257.1| enhancing factor [Yersinia pestis Nepal516]      111  1e-22
 ref|ZP_04519087.1| Enhancing factor [Yersinia pestis Nepal...     111  1e-22

  Yersinia pestis CA88-4125 [enterobacteria] taxid 412420
 ref|ZP_01917344.1| enhancin [Yersinia pestis CA88-4125]           111  1e-22

  Yersinia pestis Angola [enterobacteria] taxid 349746
 ref|YP_001605310.1| enhancing factor [Yersinia pestis Angola]     111  1e-22

  Yersinia pestis biovar Orientalis str. IP275 [enterobacteria] taxid 373665
 ref|ZP_02224939.1| viral enhancin protein [Yersinia pestis...     111  1e-22

  Yersinia pestis biovar Antiqua str. E1979001 [enterobacteria] taxid 360099
 ref|ZP_02231354.1| viral enhancin protein [Yersinia pestis...     111  1e-22

  Yersinia pestis biovar Antiqua str. UG05-0454 [enterobacteria] taxid 404218
 ref|ZP_02307965.1| viral enhancin protein [Yersinia pestis...     111  1e-22

  Yersinia pestis biovar Mediaevalis str. K1973002 [enterobacteria] taxid 404216
 ref|ZP_02317890.1| viral enhancin protein [Yersinia pestis...     111  1e-22

  Yersinia pestis FV-1 [enterobacteria] taxid 375450
 ref|ZP_02335350.1| enhancing factor (viral) [Yersinia pest...     111  1e-22

  Yersinia pestis Pestoides A [enterobacteria] taxid 545431
 ref|ZP_04456764.1| Enhancing factor [Yersinia pestis Pesto...     111  1e-22

  Yersinia pestis biovar Orientalis str. PEXU2 [enterobacteria] taxid 547046
 ref|ZP_04460366.1| Enhancing factor [Yersinia pestis biova...     111  1e-22

  Yersinia pestis biovar Orientalis str. India 195 [enterobacteria] taxid 547047
 ref|ZP_04462443.1| Enhancing factor [Yersinia pestis biova...     111  1e-22

  Yersinia pestis biovar Orientalis str. F1991016 [enterobacteria] taxid 404214
 ref|ZP_02222610.1| viral enhancin protein [Yersinia pestis...     111  1e-22

  Yersinia pestis biovar Antiqua str. B42003004 [enterobacteria] taxid 404215
 ref|ZP_02239994.1| viral enhancin protein [Yersinia pestis...     111  1e-22

  Yersinia pestis biovar Orientalis str. MG05-1020 [enterobacteria] taxid 404217
 ref|ZP_02311074.1| viral enhancin protein [Yersinia pestis...     111  1e-22

  Yersinia pestis Pestoides F [enterobacteria] taxid 386656
 ref|YP_001164956.1| enhancing factor [Yersinia pestis Pest...     111  1e-22

  Lymantria dispar MNPV [viruses] taxid 10449
 ref|NP_047797.1| viral enhancing factor 2 [Lymantria dispa...     111  2e-22
 ref|NP_047702.1| viral enhancing factor 1 [Lymantria dispa...      87  2e-15

  Yersinia pestis KIM D27 [enterobacteria] taxid 687916
 ref|ZP_06206112.1| viral enhancin protein [Yersinia pestis...     110  2e-22

  Xestia c-nigrum granulovirus [viruses] taxid 51677
 ref|NP_059300.1| ORF152 [Xestia c-nigrum granulovirus]            109  6e-22
 ref|NP_059302.1| ORF154 [Xestia c-nigrum granulovirus]             76  6e-12

  Lactobacillus jensenii 27-2-CHN [firmicutes] taxid 575606
 ref|ZP_05556372.1| enhancin family protein [Lactobacillus ...     109  6e-22

  Lactobacillus jensenii 208-1 [firmicutes] taxid 596326
 ref|ZP_06337521.1| putative enhancing factor [Lactobacillu...     108  2e-21

  Lactobacillus jensenii 115-3-CHN [firmicutes] taxid 575605
 ref|ZP_05862111.1| enhancing factor [Lactobacillus jenseni...     108  2e-21

  Helicoverpa armigera granulovirus [viruses] taxid 489830
 ref|YP_001649134.1| enhancin-2 [Helicoverpa armigera granu...     107  4e-21
 ref|YP_001649135.1| enhancin-3 [Helicoverpa armigera granu...      78  2e-12

  Yersinia kristensenii ATCC 33638 [enterobacteria] taxid 527012
 ref|ZP_04624151.1| Enhancing factor (Viral) [Yersinia kris...      94  2e-17

  Choristoneura fumiferana MNPV (spruce budworm nuclear polyhedrosis virus, ...) [viruses] taxid 208973
 ref|NP_848341.1| enhancin-like [Choristoneura fumiferana M...      89  7e-16

  Staphylococcus carnosus subsp. carnosus TM300 [firmicutes] taxid 396513
 ref|YP_002633120.1| putative surface associated protein wi...      84  2e-14

  Euproctis pseudoconspersa nucleopolyhedrovirus [viruses] taxid 307467
 ref|YP_002854632.1| vef [Euproctis pseudoconspersa nucleop...      84  3e-14

  Comamonas testosteroni KF-1 [b-proteobacteria] taxid 399795
 ref|ZP_03541921.1| conserved hypothetical protein [Comamon...      75  7e-12

  Agrotis ipsilon multiple nucleopolyhedrovirus [viruses] taxid 208013
 ref|YP_002268112.1| agip82 [Agrotis ipsilon multiple nucle...      68  1e-09

  Helicoverpa armigera multiple nucleopolyhedrovirus [viruses] taxid 559170
 ref|YP_002332617.1| putative viral enhancing factor [Helic...      68  1e-09

  Mamestra configurata NPV-B [viruses] taxid 204440
 ref|NP_689263.1| putative viral enhancing factor [Mamestra...      68  2e-09

  Agrotis segetum nucleopolyhedrovirus [viruses] taxid 31508
 ref|YP_529746.1| VEF-2 [Agrotis segetum nucleopolyhedrovirus]      64  2e-08
 ref|YP_529745.1| VEF-1 [Agrotis segetum nucleopolyhedrovirus]      57  3e-06

  Mamestra configurata NPV-A [viruses] taxid 207830
 ref|NP_613172.1| viral enhancing factor VEF [Mamestra conf...      64  3e-08

  Agrotis segetum granulovirus [viruses] taxid 10464
 ref|YP_006289.1| ORF55 [Agrotis segetum granulovirus]              61  2e-07

  Cryptosporidium hominis TU502 [apicomplexans] taxid 353151
 ref|XP_668189.1| hypothetical protein [Cryptosporidium hom...      58  2e-06

  Clostridium thermocellum DSM 2360 [firmicutes] taxid 572545
 ref|ZP_05428045.1| Carbohydrate-binding CenC domain protei...      54  2e-05

  Clostridium thermocellum ATCC 27405 [firmicutes] taxid 203119
 ref|YP_001039197.1| carbohydrate-binding, CenC-like protei...      53  4e-05

  Clostridium thermocellum JW20 [firmicutes] taxid 492476
 ref|ZP_06250293.1| Carbohydrate-binding CenC domain protei...      52  6e-05

  Clostridium cellulolyticum H10 [firmicutes] taxid 394503
 ref|YP_002505233.1| carbohydrate-binding, CenC-like protei...      52  7e-05
```

---

**Taxonomy Report**

```
root .......................................................................................   552 hits  333 orgs 
. cellular organisms .......................................................................   537 hits  322 orgs 
. . Eukaryota ..............................................................................    74 hits   24 orgs 
. . . Trichomonas vaginalis G3 .............................................................    26 hits    1 orgs [Parabasalidea; Trichomonada; Trichomonadida; Trichomonadidae; Trichomonadinae; Trichomonas; Trichomonas vaginalis]
. . . Fungi/Metazoa group ..................................................................    43 hits   18 orgs 
. . . . Chordata ...........................................................................    37 hits   14 orgs [Metazoa; Eumetazoa; Bilateria; Coelomata; Deuterostomia]
. . . . . Euteleostomi .....................................................................    35 hits   13 orgs [Craniata; Vertebrata; Gnathostomata; Teleostomi]
. . . . . . Danio rerio ....................................................................     4 hits    1 orgs [Actinopterygii; Actinopteri; Neopterygii; Teleostei; Elopocephala; Clupeocephala; Otocephala; Ostariophysi; Otophysi; Cypriniphysi; Cypriniformes; Cyprinoidea; Cyprinidae; Danio]
. . . . . . Tetrapoda ......................................................................    31 hits   12 orgs [Sarcopterygii]
. . . . . . . Amniota ......................................................................    30 hits   11 orgs 
. . . . . . . . Mammalia ...................................................................    28 hits   10 orgs 
. . . . . . . . . Eutheria .................................................................    25 hits    9 orgs [Theria]
. . . . . . . . . . Laurasiatheria .........................................................     7 hits    3 orgs 
. . . . . . . . . . . Bos taurus ...........................................................     2 hits    1 orgs [Cetartiodactyla; Ruminantia; Pecora; Bovidae; Bovinae; Bos]
. . . . . . . . . . . Equus caballus .......................................................     2 hits    1 orgs [Perissodactyla; Equidae; Equus; Equus subg. Equus]
. . . . . . . . . . . Canis lupus familiaris ...............................................     3 hits    1 orgs [Carnivora; Caniformia; Canidae; Canis; Canis lupus]
. . . . . . . . . . Euarchontoglires .......................................................    18 hits    6 orgs 
. . . . . . . . . . . Catarrhini ...........................................................    10 hits    4 orgs [Primates; Haplorrhini; Simiiformes]
. . . . . . . . . . . . Hominidae ..........................................................     8 hits    3 orgs [Hominoidea]
. . . . . . . . . . . . . Homininae ........................................................     7 hits    2 orgs 
. . . . . . . . . . . . . . Pan troglodytes ................................................     2 hits    1 orgs [Pan]
. . . . . . . . . . . . . . Homo sapiens ...................................................     5 hits    1 orgs [Homo]
. . . . . . . . . . . . . Pongo abelii .....................................................     1 hits    1 orgs [Ponginae; Pongo]
. . . . . . . . . . . . Macaca mulatta .....................................................     2 hits    1 orgs [Cercopithecoidea; Cercopithecidae; Cercopithecinae; Macaca]
. . . . . . . . . . . Murinae ..............................................................     8 hits    2 orgs [Glires; Rodentia; Sciurognathi; Muroidea; Muridae]
. . . . . . . . . . . . Mus musculus .......................................................     3 hits    1 orgs [Mus]
. . . . . . . . . . . . Rattus norvegicus ..................................................     5 hits    1 orgs [Rattus]
. . . . . . . . . Ornithorhynchus anatinus .................................................     3 hits    1 orgs [Prototheria; Monotremata; Ornithorhynchidae; Ornithorhynchus]
. . . . . . . . Taeniopygia guttata ........................................................     2 hits    1 orgs [Sauropsida; Sauria; Archosauria; Dinosauria; Saurischia; Theropoda; Coelurosauria; Aves; Neognathae; Passeriformes; Passeroidea; Estrildidae; Estrildinae; Taeniopygia]
. . . . . . . Xenopus laevis ...............................................................     1 hits    1 orgs [Amphibia; Batrachia; Anura; Mesobatrachia; Pipoidea; Pipidae; Xenopodinae; Xenopus; Xenopus]
. . . . . Branchiostoma floridae ...........................................................     2 hits    1 orgs [Cephalochordata; Branchiostomidae; Branchiostoma]
. . . . Eurotiomycetidae ...................................................................     5 hits    3 orgs [Fungi; Dikarya; Ascomycota; Pezizomycotina; Eurotiomycetes]
. . . . . Aspergillus ......................................................................     4 hits    2 orgs [Eurotiales; Trichocomaceae; mitosporic Trichocomaceae]
. . . . . . Aspergillus flavus NRRL3357 ....................................................     2 hits    1 orgs [Aspergillus flavus]
. . . . . . Aspergillus oryzae RIB40 .......................................................     2 hits    1 orgs [Aspergillus oryzae]
. . . . . Uncinocarpus reesii 1704 .........................................................     1 hits    1 orgs [Onygenales; Onygenaceae; Uncinocarpus; Uncinocarpus reesii]
. . . . Monosiga brevicollis MX1 ...........................................................     1 hits    1 orgs [Choanoflagellida; Codonosigidae; Monosiga; Monosiga brevicollis]
. . . Entamoeba ............................................................................     2 hits    2 orgs [Amoebozoa; Archamoebae; Entamoebidae]
. . . . Entamoeba dispar SAW760 ............................................................     1 hits    1 orgs [Entamoeba dispar]
. . . . Entamoeba histolytica HM-1:IMSS ....................................................     1 hits    1 orgs [Entamoeba histolytica]
. . . Cryptosporidium ......................................................................     3 hits    3 orgs [Alveolata; Apicomplexa; Coccidia; Eucoccidiorida; Eimeriorina; Cryptosporidiidae]
. . . . Cryptosporidium muris RN66 .........................................................     1 hits    1 orgs [Cryptosporidium muris]
. . . . Cryptosporidium parvum Iowa II .....................................................     1 hits    1 orgs [Cryptosporidium parvum]
. . . . Cryptosporidium hominis TU502 ......................................................     1 hits    1 orgs [Cryptosporidium hominis]
. . Bacteria ...............................................................................   463 hits  298 orgs 
. . . Verrucomicrobia ......................................................................     6 hits    3 orgs [Chlamydiae/Verrucomicrobia group]
. . . . Chthoniobacter flavus Ellin428 .....................................................     1 hits    1 orgs [Spartobacteria; Chthoniobacter; Chthoniobacter flavus]
. . . . Verrucomicrobiaceae ................................................................     5 hits    2 orgs [Verrucomicrobiae; Verrucomicrobiales]
. . . . . Verrucomicrobium spinosum DSM 4136 ...............................................     1 hits    1 orgs [Verrucomicrobium; Verrucomicrobium spinosum]
. . . . . Akkermansia muciniphila ATCC BAA-835 .............................................     4 hits    1 orgs [Akkermansia; Akkermansia muciniphila]
. . . Firmicutes ...........................................................................   238 hits  127 orgs 
. . . . Bacilli ............................................................................   187 hits   87 orgs 
. . . . . Bacillales .......................................................................   184 hits   84 orgs 
. . . . . . Bacillaceae ....................................................................   178 hits   81 orgs 
. . . . . . . Bacillus .....................................................................   176 hits   80 orgs 
. . . . . . . . Bacillus cereus group ......................................................   174 hits   79 orgs 
. . . . . . . . . Bacillus cereus ..........................................................    90 hits   39 orgs 
. . . . . . . . . . Bacillus cereus ATCC 10987 .............................................     1 hits    1 orgs 
. . . . . . . . . . Bacillus cereus m1293 ..................................................     1 hits    1 orgs 
. . . . . . . . . . Bacillus cereus AH820 ..................................................     3 hits    1 orgs 
. . . . . . . . . . Bacillus cereus Rock3-42 ...............................................     2 hits    1 orgs 
. . . . . . . . . . Bacillus cereus 95/8201 ................................................     3 hits    1 orgs 
. . . . . . . . . . Bacillus cereus G9842 ..................................................     2 hits    1 orgs 
. . . . . . . . . . Bacillus cereus W ......................................................     3 hits    1 orgs 
. . . . . . . . . . Bacillus cereus AH1271 .................................................     1 hits    1 orgs 
. . . . . . . . . . Bacillus cereus AH603 ..................................................     3 hits    1 orgs 
. . . . . . . . . . Bacillus cereus AH621 ..................................................     3 hits    1 orgs 
. . . . . . . . . . Bacillus cereus H3081.97 ...............................................     1 hits    1 orgs 
. . . . . . . . . . Bacillus cereus AH187 ..................................................     1 hits    1 orgs 
. . . . . . . . . . Bacillus cereus Q1 .....................................................     1 hits    1 orgs 
. . . . . . . . . . Bacillus cereus Rock4-2 ................................................     3 hits    1 orgs 
. . . . . . . . . . Bacillus cereus BDRD-ST26 ..............................................     1 hits    1 orgs 
. . . . . . . . . . Bacillus cereus 03BB108 ................................................     1 hits    1 orgs 
. . . . . . . . . . Bacillus cereus 03BB102 ................................................     1 hits    1 orgs 
. . . . . . . . . . Bacillus cereus B4264 ..................................................     3 hits    1 orgs 
. . . . . . . . . . Bacillus cereus BGSC 6E1 ...............................................     1 hits    1 orgs 
. . . . . . . . . . Bacillus cereus 172560W ................................................     3 hits    1 orgs 
. . . . . . . . . . Bacillus cereus F65185 .................................................     3 hits    1 orgs 
. . . . . . . . . . Bacillus cereus BDRD-ST24 ..............................................     2 hits    1 orgs 
. . . . . . . . . . Bacillus cereus Rock1-15 ...............................................     3 hits    1 orgs 
. . . . . . . . . . Bacillus cereus BDRD-Cer4 ..............................................     2 hits    1 orgs 
. . . . . . . . . . Bacillus cereus AH1134 .................................................     4 hits    1 orgs 
. . . . . . . . . . Bacillus cereus AH676 ..................................................     3 hits    1 orgs 
. . . . . . . . . . Bacillus cereus MM3 ....................................................     3 hits    1 orgs 
. . . . . . . . . . Bacillus cereus NVH0597-99 .............................................     2 hits    1 orgs 
. . . . . . . . . . Bacillus cereus ATCC 10876 .............................................     4 hits    1 orgs 
. . . . . . . . . . Bacillus cereus BDRD-ST196 .............................................     3 hits    1 orgs 
. . . . . . . . . . Bacillus cereus G9241 ..................................................     1 hits    1 orgs 
. . . . . . . . . . Bacillus cereus ATCC 14579 .............................................     2 hits    1 orgs 
. . . . . . . . . . Bacillus cereus E33L ...................................................     3 hits    1 orgs 
. . . . . . . . . . Bacillus cereus ATCC 4342 ..............................................     1 hits    1 orgs 
. . . . . . . . . . Bacillus cereus Rock1-3 ................................................     5 hits    1 orgs 
. . . . . . . . . . Bacillus cereus R309803 ................................................     1 hits    1 orgs 
. . . . . . . . . . Bacillus cereus Rock3-29 ...............................................     4 hits    1 orgs 
. . . . . . . . . . Bacillus cereus Rock3-28 ...............................................     4 hits    1 orgs 
. . . . . . . . . . Bacillus cereus m1550 ..................................................     2 hits    1 orgs 
. . . . . . . . . Bacillus thuringiensis ...................................................    47 hits   17 orgs 
. . . . . . . . . . Bacillus thuringiensis serovar pakistani str. T13001 ...................     2 hits    1 orgs [Bacillus thuringiensis serovar pakistani]
. . . . . . . . . . Bacillus thuringiensis serovar sotto str. T04001 .......................     2 hits    1 orgs [Bacillus thuringiensis serovar sotto]
. . . . . . . . . . Bacillus thuringiensis serovar konkukian str. 97-27 ....................     2 hits    1 orgs [Bacillus thuringiensis serovar konkukian]
. . . . . . . . . . Bacillus thuringiensis serovar andalousiensis BGSC 4AW1 ................     2 hits    1 orgs [Bacillus thuringiensis serovar andalousiensis]
. . . . . . . . . . Bacillus thuringiensis serovar monterrey BGSC 4AJ1 .....................     4 hits    1 orgs [Bacillus thuringiensis serovar monterrey]
. . . . . . . . . . Bacillus thuringiensis serovar pondicheriensis BGSC 4BA1 ...............     3 hits    1 orgs [Bacillus thuringiensis serovar pondicheriensis]
. . . . . . . . . . Bacillus thuringiensis IBL 200 .........................................     2 hits    1 orgs 
. . . . . . . . . . Bacillus thuringiensis serovar israelensis ATCC 35646 ..................     3 hits    1 orgs [Bacillus thuringiensis serovar israelensis]
. . . . . . . . . . Bacillus thuringiensis IBL 4222 ........................................     2 hits    1 orgs 
. . . . . . . . . . Bacillus thuringiensis serovar berliner ATCC 10792 .....................     5 hits    1 orgs [Bacillus thuringiensis serovar berliner]
. . . . . . . . . . Bacillus thuringiensis serovar thuringiensis str. T01001 ...............     5 hits    1 orgs [Bacillus thuringiensis serovar thuringiensis]
. . . . . . . . . . Bacillus thuringiensis Bt407 ...........................................     4 hits    1 orgs 
. . . . . . . . . . Bacillus thuringiensis str. Al Hakam ...................................     1 hits    1 orgs 
. . . . . . . . . . Bacillus thuringiensis serovar huazhongensis BGSC 4BD1 .................     3 hits    1 orgs [Bacillus thuringiensis serovar huazhongensis]
. . . . . . . . . . Bacillus thuringiensis serovar kurstaki str. T03a001 ...................     3 hits    1 orgs [Bacillus thuringiensis serovar kurstaki]
. . . . . . . . . . Bacillus thuringiensis serovar tochigiensis BGSC 4Y1 ...................     2 hits    1 orgs [Bacillus thuringiensis serovar tochigiensis]
. . . . . . . . . . Bacillus thuringiensis serovar pulsiensis BGSC 4CC1 ....................     2 hits    1 orgs [Bacillus thuringiensis serovar pulsiensis]
. . . . . . . . . Bacillus weihenstephanensis KBAB4 ........................................     3 hits    1 orgs [Bacillus weihenstephanensis]
. . . . . . . . . Bacillus anthracis .......................................................    30 hits   19 orgs 
. . . . . . . . . . Bacillus anthracis str. A1055 ..........................................     2 hits    1 orgs 
. . . . . . . . . . Bacillus anthracis str. Ames ...........................................     2 hits    1 orgs 
. . . . . . . . . . Bacillus anthracis str. 'Ames Ancestor' ................................     2 hits    1 orgs 
. . . . . . . . . . Bacillus anthracis str. Sterne .........................................     2 hits    1 orgs 
. . . . . . . . . . Bacillus anthracis str. CDC 684 ........................................     2 hits    1 orgs 
. . . . . . . . . . Bacillus anthracis str. CNEVA-9066 .....................................     2 hits    1 orgs 
. . . . . . . . . . Bacillus anthracis str. Western North America USA6153 ..................     2 hits    1 orgs 
. . . . . . . . . . Bacillus anthracis str. Kruger B .......................................     2 hits    1 orgs 
. . . . . . . . . . Bacillus anthracis str. Vollum .........................................     2 hits    1 orgs 
. . . . . . . . . . Bacillus anthracis str. Australia 94 ...................................     2 hits    1 orgs 
. . . . . . . . . . Bacillus anthracis str. A2012 ..........................................     2 hits    1 orgs 
. . . . . . . . . . Bacillus anthracis str. A0488 ..........................................     1 hits    1 orgs 
. . . . . . . . . . Bacillus anthracis str. A0193 ..........................................     1 hits    1 orgs 
. . . . . . . . . . Bacillus anthracis str. A0389 ..........................................     1 hits    1 orgs 
. . . . . . . . . . Bacillus anthracis str. A0174 ..........................................     1 hits    1 orgs 
. . . . . . . . . . Bacillus anthracis Tsiankovskii-I ......................................     1 hits    1 orgs 
. . . . . . . . . . Bacillus anthracis str. A0248 ..........................................     1 hits    1 orgs 
. . . . . . . . . . Bacillus anthracis str. A0442 ..........................................     1 hits    1 orgs 
. . . . . . . . . . Bacillus anthracis str. A0465 ..........................................     1 hits    1 orgs 
. . . . . . . . . Bacillus cytotoxicus NVH 391-98 ..........................................     1 hits    1 orgs [Bacillus cytotoxicus]
. . . . . . . . . Bacillus mycoides ........................................................     3 hits    2 orgs 
. . . . . . . . . . Bacillus mycoides DSM 2048 .............................................     2 hits    1 orgs 
. . . . . . . . . . Bacillus mycoides Rock3-17 .............................................     1 hits    1 orgs 
. . . . . . . . Bacillus pseudomycoides DSM 12442 ..........................................     2 hits    1 orgs [Bacillus pseudomycoides]
. . . . . . . Geobacillus sp. Y412MC10 .....................................................     2 hits    1 orgs [Geobacillus]
. . . . . . Paenibacillus larvae subsp. larvae BRL-230010 ..................................     3 hits    1 orgs [Paenibacillaceae; Paenibacillus; Paenibacillus larvae; Paenibacillus larvae subsp. larvae]
. . . . . . Listeria grayi DSM 20601 .......................................................     2 hits    1 orgs [Listeriaceae; Listeria; Listeria grayi]
. . . . . . Staphylococcus carnosus subsp. carnosus TM300 ..................................     1 hits    1 orgs [Staphylococcaceae; Staphylococcus; Staphylococcus carnosus; Staphylococcus carnosus subsp. carnosus]
. . . . . Lactobacillus jensenii ...........................................................     3 hits    3 orgs [Lactobacillales; Lactobacillaceae; Lactobacillus]
. . . . . . Lactobacillus jensenii 27-2-CHN ................................................     1 hits    1 orgs 
. . . . . . Lactobacillus jensenii 208-1 ...................................................     1 hits    1 orgs 
. . . . . . Lactobacillus jensenii 115-3-CHN ...............................................     1 hits    1 orgs 
. . . . Clostridiales ......................................................................    49 hits   38 orgs [Clostridia]
. . . . . Clostridium ......................................................................    48 hits   37 orgs [Clostridiaceae]
. . . . . . Clostridium perfringens ........................................................    18 hits    9 orgs 
. . . . . . . Clostridium perfringens E str. JGS1987 .......................................     1 hits    1 orgs [Clostridium perfringens E]
. . . . . . . Clostridium perfringens D str. JGS1721 .......................................     6 hits    1 orgs [Clostridium perfringens D]
. . . . . . . Clostridium perfringens B str. ATCC 3626 .....................................     2 hits    1 orgs [Clostridium perfringens B]
. . . . . . . Clostridium perfringens CPE str. F4969 .......................................     2 hits    1 orgs [Clostridium perfringens CPE]
. . . . . . . Clostridium perfringens C str. JGS1495 .......................................     1 hits    1 orgs [Clostridium perfringens C]
. . . . . . . Clostridium perfringens SM101 ................................................     1 hits    1 orgs 
. . . . . . . Clostridium perfringens ATCC 13124 ...........................................     2 hits    1 orgs 
. . . . . . . Clostridium perfringens str. 13 ..............................................     1 hits    1 orgs 
. . . . . . . Clostridium perfringens NCTC 8239 ............................................     2 hits    1 orgs 
. . . . . . Clostridium botulinum ..........................................................     7 hits    7 orgs 
. . . . . . . Clostridium botulinum E ......................................................     2 hits    2 orgs 
. . . . . . . . Clostridium botulinum E3 str. Alaska E43 ...................................     1 hits    1 orgs 
. . . . . . . . Clostridium botulinum E1 str. 'BoNT E Beluga' ..............................     1 hits    1 orgs 
. . . . . . . Clostridium botulinum A2 str. Kyoto ..........................................     1 hits    1 orgs [Clostridium botulinum A]
. . . . . . . Clostridium botulinum F str. Langeland .......................................     1 hits    1 orgs [Clostridium botulinum F]
. . . . . . . Clostridium botulinum Bf .....................................................     1 hits    1 orgs 
. . . . . . . Clostridium botulinum B ......................................................     2 hits    2 orgs 
. . . . . . . . Clostridium botulinum Ba4 str. 657 .........................................     1 hits    1 orgs 
. . . . . . . . Clostridium botulinum B1 str. Okra .........................................     1 hits    1 orgs [Clostridium botulinum B1]
. . . . . . Clostridium bartlettii DSM 16795 ...............................................     2 hits    1 orgs [Clostridium bartlettii]
. . . . . . Clostridium sp. 7_2_43FAA ......................................................     1 hits    1 orgs 
. . . . . . Clostridium hathewayi DSM 13479 ................................................     1 hits    1 orgs [Clostridium hathewayi]
. . . . . . Clostridium difficile ..........................................................    12 hits   11 orgs 
. . . . . . . Clostridium difficile QCD-66c26 ..............................................     1 hits    1 orgs 
. . . . . . . Clostridium difficile CIP 107932 .............................................     1 hits    1 orgs 
. . . . . . . Clostridium difficile QCD-76w55 ..............................................     1 hits    1 orgs 
. . . . . . . Clostridium difficile QCD-97b34 ..............................................     1 hits    1 orgs 
. . . . . . . Clostridium difficile QCD-37x79 ..............................................     1 hits    1 orgs 
. . . . . . . Clostridium difficile CD196 ..................................................     1 hits    1 orgs 
. . . . . . . Clostridium difficile R20291 .................................................     1 hits    1 orgs 
. . . . . . . Clostridium difficile QCD-63q42 ..............................................     1 hits    1 orgs 
. . . . . . . Clostridium difficile ATCC 43255 .............................................     1 hits    1 orgs 
. . . . . . . Clostridium difficile 630 ....................................................     1 hits    1 orgs 
. . . . . . . Clostridium difficile QCD-32g58 ..............................................     2 hits    1 orgs 
. . . . . . Clostridium hiranonis DSM 13275 ................................................     1 hits    1 orgs [Clostridium hiranonis]
. . . . . . Clostridium butyricum ..........................................................     2 hits    2 orgs 
. . . . . . . Clostridium butyricum 5521 ...................................................     1 hits    1 orgs 
. . . . . . . Clostridium butyricum E4 str. BoNT E BL5262 ..................................     1 hits    1 orgs 
. . . . . . Clostridium thermocellum .......................................................     3 hits    3 orgs 
. . . . . . . Clostridium thermocellum DSM 2360 ............................................     1 hits    1 orgs 
. . . . . . . Clostridium thermocellum ATCC 27405 ..........................................     1 hits    1 orgs 
. . . . . . . Clostridium thermocellum JW20 ................................................     1 hits    1 orgs 
. . . . . . Clostridium cellulolyticum H10 .................................................     1 hits    1 orgs [Clostridium cellulolyticum]
. . . . . Subdoligranulum variabile DSM 15176 ..............................................     1 hits    1 orgs [Ruminococcaceae; Subdoligranulum; Subdoligranulum variabile]
. . . . unclassified Erysipelotrichaceae ...................................................     2 hits    2 orgs [Erysipelotrichi; Erysipelotrichales; Erysipelotrichaceae]
. . . . . Eubacterium dolichum DSM 3991 ....................................................     1 hits    1 orgs [Eubacterium dolichum]
. . . . . Clostridium ramosum DSM 1402 .....................................................     1 hits    1 orgs [Clostridium ramosum]
. . . Planctomyces limnophilus DSM 3776 ....................................................     1 hits    1 orgs [Planctomycetes; Planctomycetacia; Planctomycetales; Planctomycetaceae; Planctomyces; Planctomyces limnophilus]
. . . Bacteroidetes ........................................................................    50 hits   18 orgs [Bacteroidetes/Chlorobi group]
. . . . Sphingobacteriales .................................................................    13 hits    3 orgs [Sphingobacteria]
. . . . . Chitinophaga pinensis DSM 2588 ...................................................     1 hits    1 orgs [Chitinophagaceae; Chitinophaga; Chitinophaga pinensis]
. . . . . Sphingobacterium spiritivorum ....................................................    12 hits    2 orgs [Sphingobacteriaceae; Sphingobacterium]
. . . . . . Sphingobacterium spiritivorum ATCC 33861 .......................................     6 hits    1 orgs 
. . . . . . Sphingobacterium spiritivorum ATCC 33300 .......................................     6 hits    1 orgs 
. . . . Bacteroidales ......................................................................    37 hits   15 orgs [Bacteroidia]
. . . . . Bacteroides ......................................................................    36 hits   14 orgs [Bacteroidaceae]
. . . . . . Bacteroides thetaiotaomicron VPI-5482 ..........................................     4 hits    1 orgs [Bacteroides thetaiotaomicron]
. . . . . . Bacteroides sp. 1_1_6 ..........................................................     4 hits    1 orgs 
. . . . . . Bacteroides sp. 3_2_5 ..........................................................     1 hits    1 orgs 
. . . . . . Bacteroides caccae ATCC 43185 ..................................................    16 hits    1 orgs [Bacteroides caccae]
. . . . . . Bacteroides fragilis ...........................................................     3 hits    3 orgs 
. . . . . . . Bacteroides fragilis YCH46 ...................................................     1 hits    1 orgs 
. . . . . . . Bacteroides fragilis NCTC 9343 ...............................................     1 hits    1 orgs 
. . . . . . . Bacteroides fragilis 3_1_12 ..................................................     1 hits    1 orgs 
. . . . . . Bacteroides sp. 2_1_16 .........................................................     1 hits    1 orgs 
. . . . . . Bacteroides plebeius DSM 17135 .................................................     2 hits    1 orgs [Bacteroides plebeius]
. . . . . . Bacteroides vulgatus ATCC 8482 .................................................     1 hits    1 orgs [Bacteroides vulgatus]
. . . . . . Bacteroides coprophilus DSM 18228 ..............................................     1 hits    1 orgs [Bacteroides coprophilus]
. . . . . . Bacteroides capillosus ATCC 29799 ..............................................     1 hits    1 orgs [Bacteroides capillosus]
. . . . . . Bacteroides finegoldii DSM 17565 ...............................................     1 hits    1 orgs [Bacteroides finegoldii]
. . . . . . Bacteroides sp. D2 .............................................................     1 hits    1 orgs 
. . . . . Prevotella melaninogenica ATCC 25845 .............................................     1 hits    1 orgs [Prevotellaceae; Prevotella; Prevotella melaninogenica]
. . . Proteobacteria .......................................................................   162 hits  145 orgs 
. . . . Gammaproteobacteria ................................................................   161 hits  144 orgs 
. . . . . Enterobacteriaceae ...............................................................    71 hits   70 orgs [Enterobacteriales]
. . . . . . Yersinia .......................................................................    30 hits   29 orgs 
. . . . . . . Yersinia ruckeri ATCC 29473 ..................................................     1 hits    1 orgs [Yersinia ruckeri]
. . . . . . . Yersinia enterocolitica subsp. enterocolitica 8081 ...........................     1 hits    1 orgs [Yersinia enterocolitica; Yersinia enterocolitica subsp. enterocolitica]
. . . . . . . Yersinia aldovae ATCC 35236 ..................................................     1 hits    1 orgs [Yersinia aldovae]
. . . . . . . Yersinia mollaretii ATCC 43969 ...............................................     1 hits    1 orgs [Yersinia mollaretii]
. . . . . . . Yersinia pseudotuberculosis ..................................................     4 hits    4 orgs 
. . . . . . . . Yersinia pseudotuberculosis IP 31758 .......................................     1 hits    1 orgs 
. . . . . . . . Yersinia pseudotuberculosis IP 32953 .......................................     1 hits    1 orgs 
. . . . . . . . Yersinia pseudotuberculosis YPIII ..........................................     1 hits    1 orgs 
. . . . . . . . Yersinia pseudotuberculosis PB1/+ ..........................................     1 hits    1 orgs 
. . . . . . . Yersinia pestis ..............................................................    21 hits   20 orgs 
. . . . . . . . Yersinia pestis KIM 10 .....................................................     2 hits    2 orgs 
. . . . . . . . . Yersinia pestis KIM D27 ..................................................     1 hits    1 orgs 
. . . . . . . . Yersinia pestis biovar Microtus str. 91001 .................................     1 hits    1 orgs 
. . . . . . . . Yersinia pestis CO92 .......................................................     1 hits    1 orgs 
. . . . . . . . Yersinia pestis Antiqua ....................................................     1 hits    1 orgs 
. . . . . . . . Yersinia pestis Nepal516 ...................................................     2 hits    1 orgs 
. . . . . . . . Yersinia pestis CA88-4125 ..................................................     1 hits    1 orgs 
. . . . . . . . Yersinia pestis Angola .....................................................     1 hits    1 orgs 
. . . . . . . . Yersinia pestis biovar Orientalis str. IP275 ...............................     1 hits    1 orgs 
. . . . . . . . Yersinia pestis biovar Antiqua str. E1979001 ...............................     1 hits    1 orgs 
. . . . . . . . Yersinia pestis biovar Antiqua str. UG05-0454 ..............................     1 hits    1 orgs 
. . . . . . . . Yersinia pestis biovar Mediaevalis str. K1973002 ...........................     1 hits    1 orgs 
. . . . . . . . Yersinia pestis FV-1 .......................................................     1 hits    1 orgs 
. . . . . . . . Yersinia pestis Pestoides A ................................................     1 hits    1 orgs 
. . . . . . . . Yersinia pestis biovar Orientalis str. PEXU2 ...............................     1 hits    1 orgs 
. . . . . . . . Yersinia pestis biovar Orientalis str. India 195 ...........................     1 hits    1 orgs 
. . . . . . . . Yersinia pestis biovar Orientalis str. F1991016 ............................     1 hits    1 orgs 
. . . . . . . . Yersinia pestis biovar Antiqua str. B42003004 ..............................     1 hits    1 orgs 
. . . . . . . . Yersinia pestis biovar Orientalis str. MG05-1020 ...........................     1 hits    1 orgs 
. . . . . . . . Yersinia pestis Pestoides F ................................................     1 hits    1 orgs 
. . . . . . . Yersinia kristensenii ATCC 33638 .............................................     1 hits    1 orgs [Yersinia kristensenii]
. . . . . . Escherichia ....................................................................    32 hits   32 orgs 
. . . . . . . Escherichia coli .............................................................    27 hits   27 orgs 
. . . . . . . . Escherichia coli B7A .......................................................     1 hits    1 orgs 
. . . . . . . . Escherichia coli O111:H- str. 11128 ........................................     1 hits    1 orgs [Escherichia coli O111:H-]
. . . . . . . . Escherichia coli K-12 ......................................................     3 hits    3 orgs 
. . . . . . . . . Escherichia coli str. K-12 substr. MG1655 ................................     1 hits    1 orgs 
. . . . . . . . . Escherichia coli str. K-12 substr. W3110 .................................     1 hits    1 orgs 
. . . . . . . . . Escherichia coli BW2952 ..................................................     1 hits    1 orgs 
. . . . . . . . Escherichia coli BL21(DE3) .................................................     1 hits    1 orgs 
. . . . . . . . Escherichia coli 53638 .....................................................     1 hits    1 orgs 
. . . . . . . . Escherichia coli 101-1 .....................................................     1 hits    1 orgs 
. . . . . . . . Escherichia coli IAI39 .....................................................     1 hits    1 orgs 
. . . . . . . . Escherichia coli SE11 ......................................................     1 hits    1 orgs 
. . . . . . . . Escherichia coli 83972 .....................................................     1 hits    1 orgs 
. . . . . . . . Escherichia coli UMN026 ....................................................     1 hits    1 orgs 
. . . . . . . . Escherichia coli E22 .......................................................     1 hits    1 orgs [Escherichia coli O103:H2]
. . . . . . . . Escherichia coli B str. REL606 .............................................     1 hits    1 orgs [Escherichia coli B]
. . . . . . . . Escherichia coli E110019 ...................................................     1 hits    1 orgs 
. . . . . . . . Escherichia coli F11 .......................................................     1 hits    1 orgs 
. . . . . . . . Escherichia coli SMS-3-5 ...................................................     1 hits    1 orgs 
. . . . . . . . Escherichia coli HS ........................................................     1 hits    1 orgs 
. . . . . . . . Escherichia coli 536 .......................................................     1 hits    1 orgs 
. . . . . . . . Escherichia coli E24377A ...................................................     1 hits    1 orgs 
. . . . . . . . Escherichia coli O127:H6 str. E2348/69 .....................................     1 hits    1 orgs [Escherichia coli O127:H6]
. . . . . . . . Escherichia coli 55989 .....................................................     1 hits    1 orgs 
. . . . . . . . Escherichia coli S88 .......................................................     1 hits    1 orgs 
. . . . . . . . Escherichia coli UTI89 .....................................................     1 hits    1 orgs 
. . . . . . . . Escherichia coli APEC O1 ...................................................     1 hits    1 orgs 
. . . . . . . . Escherichia coli ED1a ......................................................     1 hits    1 orgs 
. . . . . . . . Escherichia coli IAI1 ......................................................     1 hits    1 orgs 
. . . . . . . Escherichia sp. 1_1_43 .......................................................     1 hits    1 orgs 
. . . . . . . Escherichia sp. 4_1_40B ......................................................     1 hits    1 orgs 
. . . . . . . Escherichia sp. 3_2_53FAA ....................................................     1 hits    1 orgs 
. . . . . . . Escherichia albertii TW07627 .................................................     1 hits    1 orgs [Escherichia albertii]
. . . . . . . Escherichia fergusonii ATCC 35469 ............................................     1 hits    1 orgs [Escherichia fergusonii]
. . . . . . Shigella sp. D9 ................................................................     1 hits    1 orgs [Shigella]
. . . . . . Photorhabdus asymbiotica .......................................................     1 hits    1 orgs [Photorhabdus]
. . . . . . Salmonella enterica ............................................................     6 hits    6 orgs [Salmonella]
. . . . . . . Salmonella enterica subsp. arizonae serovar 62:z4,z23:-- .....................     1 hits    1 orgs [Salmonella enterica subsp. arizonae]
. . . . . . . Salmonella enterica subsp. enterica ..........................................     5 hits    5 orgs 
. . . . . . . . Salmonella enterica subsp. enterica serovar Dublin str. CT_02021853 ........     1 hits    1 orgs [Salmonella enterica subsp. enterica serovar Dublin]
. . . . . . . . Salmonella enterica subsp. enterica serovar Enteritidis str. P125109 .......     1 hits    1 orgs [Salmonella enterica subsp. enterica serovar Enteritidis]
. . . . . . . . Salmonella enterica subsp. enterica serovar Schwarzengrund .................     2 hits    2 orgs 
. . . . . . . . . Salmonella enterica subsp. enterica serovar Schwarzengrund str. SL480 ....     1 hits    1 orgs 
. . . . . . . . . Salmonella enterica subsp. enterica serovar Schwarzengrund str. CVM19633 .     1 hits    1 orgs 
. . . . . . . . Salmonella enterica subsp. enterica serovar Javiana str. GA_MM04042433 .....     1 hits    1 orgs [Salmonella enterica subsp. enterica serovar Javiana]
. . . . . . Pantoea sp. At-9b ..............................................................     1 hits    1 orgs [Pantoea]
. . . . . Pseudomonas ......................................................................    13 hits   11 orgs [Pseudomonadales; Pseudomonadaceae]
. . . . . . Pseudomonas syringae group .....................................................     6 hits    4 orgs 
. . . . . . . Pseudomonas syringae pv. oryzae str. 1_6 .....................................     1 hits    1 orgs [Pseudomonas coronafaciens; Pseudomonas syringae pv. oryzae]
. . . . . . . Pseudomonas syringae pv. syringae B728a ......................................     2 hits    1 orgs [Pseudomonas syringae group genomosp. 1; Pseudomonas syringae; Pseudomonas syringae pv. syringae]
. . . . . . . Pseudomonas syringae pv. tomato ..............................................     3 hits    2 orgs [Pseudomonas syringae group genomosp. 3]
. . . . . . . . Pseudomonas syringae pv. tomato str. DC3000 ................................     2 hits    1 orgs 
. . . . . . . . Pseudomonas syringae pv. tomato T1 .........................................     1 hits    1 orgs 
. . . . . . Pseudomonas aeruginosa .........................................................     7 hits    7 orgs [Pseudomonas aeruginosa group]
. . . . . . . Pseudomonas aeruginosa PA7 ...................................................     1 hits    1 orgs 
. . . . . . . Pseudomonas aeruginosa PACS2 .................................................     1 hits    1 orgs 
. . . . . . . Pseudomonas aeruginosa 2192 ..................................................     1 hits    1 orgs 
. . . . . . . Pseudomonas aeruginosa C3719 .................................................     1 hits    1 orgs 
. . . . . . . Pseudomonas aeruginosa PAO1 ..................................................     1 hits    1 orgs 
. . . . . . . Pseudomonas aeruginosa LESB58 ................................................     1 hits    1 orgs 
. . . . . . . Pseudomonas aeruginosa UCBPP-PA14 ............................................     1 hits    1 orgs 
. . . . . Vibrionales ......................................................................    61 hits   49 orgs 
. . . . . . Vibrionaceae ...................................................................    60 hits   48 orgs 
. . . . . . . Vibrio .......................................................................    55 hits   44 orgs 
. . . . . . . . Vibrio mimicus .............................................................     5 hits    4 orgs 
. . . . . . . . . Vibrio mimicus VM223 .....................................................     1 hits    1 orgs 
. . . . . . . . . Vibrio mimicus MB-451 ....................................................     1 hits    1 orgs 
. . . . . . . . . Vibrio mimicus VM573 .....................................................     2 hits    1 orgs 
. . . . . . . . . Vibrio mimicus VM603 .....................................................     1 hits    1 orgs 
. . . . . . . . Vibrio sp. RC586 ...........................................................     1 hits    1 orgs 
. . . . . . . . Vibrio parahaemolyticus ....................................................    12 hits    7 orgs 
. . . . . . . . . Vibrio parahaemolyticus RIMD 2210633 .....................................     2 hits    1 orgs 
. . . . . . . . . Vibrio parahaemolyticus K5030 ............................................     2 hits    1 orgs 
. . . . . . . . . Vibrio parahaemolyticus AN-5034 ..........................................     1 hits    1 orgs 
. . . . . . . . . Vibrio parahaemolyticus Peru-466 .........................................     2 hits    1 orgs 
. . . . . . . . . Vibrio parahaemolyticus AQ3810 ...........................................     2 hits    1 orgs 
. . . . . . . . . Vibrio parahaemolyticus AQ4037 ...........................................     2 hits    1 orgs 
. . . . . . . . . Vibrio parahaemolyticus 16 ...............................................     1 hits    1 orgs 
. . . . . . . . Vibrio cholerae ............................................................    25 hits   22 orgs 
. . . . . . . . . Vibrio cholerae bv. albensis VL426 .......................................     1 hits    1 orgs [Vibrio cholerae bv. albensis]
. . . . . . . . . Vibrio cholerae RC385 ....................................................     1 hits    1 orgs 
. . . . . . . . . Vibrio cholerae O1 .......................................................     2 hits    2 orgs 
. . . . . . . . . . Vibrio cholerae O1 biovar El Tor str. N16961 ...........................     1 hits    1 orgs [Vibrio cholerae O1 biovar El Tor]
. . . . . . . . . . Vibrio cholerae MJ-1236 ................................................     1 hits    1 orgs 
. . . . . . . . . Vibrio cholerae 2740-80 ..................................................     1 hits    1 orgs 
. . . . . . . . . Vibrio cholerae V52 ......................................................     1 hits    1 orgs 
. . . . . . . . . Vibrio cholerae NCTC 8457 ................................................     1 hits    1 orgs 
. . . . . . . . . Vibrio cholerae B33 ......................................................     2 hits    1 orgs 
. . . . . . . . . Vibrio cholerae M66-2 ....................................................     1 hits    1 orgs 
. . . . . . . . . Vibrio cholerae BX 330286 ................................................     1 hits    1 orgs 
. . . . . . . . . Vibrio cholerae RC9 ......................................................     1 hits    1 orgs 
. . . . . . . . . Vibrio cholerae MO10 .....................................................     1 hits    1 orgs 
. . . . . . . . . Vibrio cholera CIRS 101 ..................................................     1 hits    1 orgs 
. . . . . . . . . Vibrio cholerae INDRE 91/1 ...............................................     1 hits    1 orgs 
. . . . . . . . . Vibrio cholerae V51 ......................................................     2 hits    1 orgs 
. . . . . . . . . Vibrio cholerae TMA 21 ...................................................     1 hits    1 orgs 
. . . . . . . . . Vibrio cholerae AM-19226 .................................................     1 hits    1 orgs 
. . . . . . . . . Vibrio cholerae O395 .....................................................     1 hits    1 orgs 
. . . . . . . . . Vibrio cholerae 1587 .....................................................     1 hits    1 orgs 
. . . . . . . . . Vibrio cholerae 623-39 ...................................................     1 hits    1 orgs 
. . . . . . . . . Vibrio cholerae MAK 757 ..................................................     1 hits    1 orgs 
. . . . . . . . . Vibrio cholerae MZO-3 ....................................................     2 hits    1 orgs 
. . . . . . . . Vibrio vulnificus ..........................................................     3 hits    2 orgs 
. . . . . . . . . Vibrio vulnificus CMCP6 ..................................................     2 hits    1 orgs 
. . . . . . . . . Vibrio vulnificus YJ016 ..................................................     1 hits    1 orgs 
. . . . . . . . Vibrio harveyi .............................................................     2 hits    2 orgs 
. . . . . . . . . Vibrio harveyi 1DA3 ......................................................     1 hits    1 orgs 
. . . . . . . . . Vibrio harveyi HY01 ......................................................     1 hits    1 orgs 
. . . . . . . . Vibrio sp. Ex25 ............................................................     2 hits    1 orgs 
. . . . . . . . Vibrio alginolyticus .......................................................     2 hits    2 orgs 
. . . . . . . . . Vibrio alginolyticus 12G01 ...............................................     1 hits    1 orgs 
. . . . . . . . . Vibrio alginolyticus 40B .................................................     1 hits    1 orgs 
. . . . . . . . Vibrio orientalis CIP 102891 ...............................................     1 hits    1 orgs [Vibrio orientalis]
. . . . . . . . Vibrio sp. MED222 ..........................................................     1 hits    1 orgs 
. . . . . . . . Vibrio splendidus LGP32 ....................................................     1 hits    1 orgs [Vibrio splendidus]
. . . . . . . Grimontia hollisae CIP 101886 ................................................     2 hits    1 orgs [Grimontia; Grimontia hollisae]
. . . . . . . Vibrio fischeri ..............................................................     2 hits    2 orgs [Aliivibrio]
. . . . . . . . Vibrio fischeri MJ11 .......................................................     1 hits    1 orgs 
. . . . . . . . Vibrio fischeri ES114 ......................................................     1 hits    1 orgs 
. . . . . . . Photobacterium damselae subsp. damselae CIP 102761 ...........................     1 hits    1 orgs [Photobacterium; Photobacterium damselae; Photobacterium damselae subsp. damselae]
. . . . . . Vibrionales bacterium SWAT-3 ...................................................     1 hits    1 orgs [unclassified Vibrionales]
. . . . . Shewanella .......................................................................    15 hits   13 orgs [Alteromonadales; Shewanellaceae]
. . . . . . Shewanella halifaxensis HAW-EB4 ................................................     2 hits    1 orgs [Shewanella halifaxensis]
. . . . . . Shewanella pealeana ATCC 700345 ................................................     2 hits    1 orgs [Shewanella pealeana]
. . . . . . Shewanella woodyi ATCC 51908 ...................................................     1 hits    1 orgs [Shewanella woodyi]
. . . . . . Shewanella loihica PV-4 ........................................................     1 hits    1 orgs [Shewanella loihica]
. . . . . . Shewanella oneidensis MR-1 .....................................................     1 hits    1 orgs [Shewanella oneidensis]
. . . . . . Shewanella sp. MR-7 ............................................................     1 hits    1 orgs 
. . . . . . Shewanella sp. ANA-3 ...........................................................     1 hits    1 orgs 
. . . . . . Shewanella sp. MR-4 ............................................................     1 hits    1 orgs 
. . . . . . Shewanella baltica .............................................................     4 hits    4 orgs 
. . . . . . . Shewanella baltica OS185 .....................................................     1 hits    1 orgs 
. . . . . . . Shewanella baltica OS195 .....................................................     1 hits    1 orgs 
. . . . . . . Shewanella baltica OS155 .....................................................     1 hits    1 orgs 
. . . . . . . Shewanella baltica OS223 .....................................................     1 hits    1 orgs 
. . . . . . Shewanella amazonensis SB2B ....................................................     1 hits    1 orgs [Shewanella amazonensis]
. . . . . Hahella chejuensis KCTC 2396 .....................................................     1 hits    1 orgs [Oceanospirillales; Hahellaceae; Hahella; Hahella chejuensis]
. . . . Comamonas testosteroni KF-1 ........................................................     1 hits    1 orgs [Betaproteobacteria; Burkholderiales; Comamonadaceae; Comamonas; Comamonas testosteroni]
. . . Actinobacteria (class) ...............................................................     2 hits    2 orgs [Actinobacteria]
. . . . Brachybacterium faecium DSM 4810 ...................................................     1 hits    1 orgs [Actinobacteridae; Actinomycetales; Micrococcineae; Dermabacteraceae; Brachybacterium; Brachybacterium faecium]
. . . . Eggerthella lenta DSM 2243 .........................................................     1 hits    1 orgs [Coriobacteridae; Coriobacteriales; Coriobacterineae; Coriobacteriaceae; Eggerthella; Eggerthella lenta]
. . . Mollicutes ...........................................................................     4 hits    2 orgs [Tenericutes]
. . . . Mycoplasma penetrans HF-2 ..........................................................     3 hits    1 orgs [Mycoplasmatales; Mycoplasmataceae; Mycoplasma; Mycoplasma penetrans]
. . . . Mollicutes bacterium D7 ............................................................     1 hits    1 orgs [unclassified Mollicutes]
. Baculoviridae ............................................................................    15 hits   11 orgs [Viruses; dsDNA viruses, no RNA stage]
. . Alphabaculovirus .......................................................................    10 hits    8 orgs 
. . . Lymantria dispar MNPV ................................................................     2 hits    1 orgs 
. . . Choristoneura fumiferana MNPV ........................................................     1 hits    1 orgs 
. . . unclassified Nucleopolyhedrovirus ....................................................     4 hits    3 orgs 
. . . . Euproctis pseudoconspersa nucleopolyhedrovirus .....................................     1 hits    1 orgs 
. . . . Helicoverpa armigera multiple nucleopolyhedrovirus .................................     1 hits    1 orgs 
. . . . Agrotis segetum nucleopolyhedrovirus ...............................................     2 hits    1 orgs 
. . . Agrotis ipsilon multiple nucleopolyhedrovirus ........................................     1 hits    1 orgs 
. . . Mamestra configurata NPV-B ...........................................................     1 hits    1 orgs 
. . . Mamestra configurata NPV-A ...........................................................     1 hits    1 orgs 
. . Betabaculovirus ........................................................................     5 hits    3 orgs 
. . . Xestia c-nigrum granulovirus .........................................................     2 hits    1 orgs 
. . . Helicoverpa armigera granulovirus ....................................................     2 hits    1 orgs 
. . . Agrotis segetum granulovirus .........................................................     1 hits    1 orgs [unclassified Betabaculovirus]
```


NCBI |
NLM |
NIH |
DHHS

Copyright |
Disclaimer |
Privacy |
Accessibility |
Contact |
Send feedback
