# Supplementary material for: A Novel Extracellular Metallopeptidase Domain Shared by Animal Host-Associated Mutualistic and Pathogenic Microbes
Source: PLoS One. 2012 Jan 27;7(1):e30287. doi: 10.1371/journal.pone.0030287 (PMC3267712; doi:10.1371/journal.pone.0030287)
Supplement: Table S4 — HHPred search hit list performed with the PF13402 seed alignment (Figure S3) using the global alignment mode and against all available databases. Format: html file to be opened with a web browser. (HTML) [file pone.0030287.s011.html]

**Table S6. Homology detection & structure prediction by HMM-HMM comparison for the M60-like/PF13402 profile.**

```
Query Q5E705_VIBF1/1109-1410/Q5E705 (seq=GSRQSAGVWI...FFGQLKLWAE Len=302 Neff=8.7  Nseqs=128)  
Parameters  score SS:yes search:local realign with MAP:no  


   No Hit                             Prob E-value P-value  Score    SS Cols Query HMM  Template HMM
  1 PTHR15730 EXPERIMENTAL AUTOIMM 100.0       0       0  389.4  14.2  282    3-302   546-843 (928)
  2 PF03272 Enhancin:  Viral enhan 100.0 2.2E-37 1.8E-42  307.8  25.8  281    1-302    27-331 (775)
  3 pfam03272 Enhancin Viral enhan 100.0 1.3E-33 1.1E-38  277.7  22.4  287    1-302    27-330 (775)
  4 pfam07828 PA-IL PA-IL-like pro  88.8     6.1   5E-05   32.0   6.6   85    4-88     14-120 (121)
  5 3N2V_Entity_1                   80.2     5.1 4.2E-05   34.4   3.3   90  141-230    21-117 (158)
  6 3N2U_Entity_1                   80.2     5.1 4.2E-05   34.4   3.3   90  141-230    21-117 (158)
  7 1l7l_A PA-I galactophilic lect  78.6     3.8 3.1E-05   33.0   1.8   15    4-18     14-28  (121)
  8 1qib_A Protein (gelatinase A);  78.3     3.9 3.2E-05   35.2   2.0   90  141-230    22-119 (161)
  9 1i76_A MMP-8;, neutrophil coll  76.9     9.9 8.1E-05   33.3   4.0   86  141-230    26-122 (163)
 10 1hv5_A Stromelysin 3; inhibiti  76.5      11 9.3E-05   32.5   4.3   90  141-230    26-123 (165)
 11 PF12388 Peptidase_M57:  Dual-a  75.6      11 9.3E-05   34.6   4.1   85  144-231    56-144 (211)
 12 1y93_A Macrophage metalloelast  74.8     5.9 4.8E-05   34.1   2.2   90  141-230    22-118 (159)
 13 d1i76a_ d.92.1.11 (A:) Neutrop  74.7     7.2 5.9E-05   33.9   2.7   90  141-230    26-122 (163)
 14 1hy7_A Stromelysin-1, MMP-3; m  73.3     9.4 7.7E-05   33.5   3.0   90  141-230    27-123 (173)
 15 d1l7la_ b.18.1.16 (A:) PA-IL,   73.1      18 0.00015   28.9   4.0   78    4-81     14-103 (121)
 16 1mmq_A Matrilysin; metalloprot  72.3     9.9 8.1E-05   33.2   3.0   90  141-230    27-124 (170)
 17 SUPFAM0037477 d.92.1 Metallopr  71.4     4.4 3.6E-05   32.1   0.6    9  224-232   117-125 (172)
 18 d1hfca_ d.92.1.11 (A:) Fibrobl  71.0      16 0.00013   31.4   3.9   90  141-230    20-116 (157)
 19 SUPFAM0042596 d.92.1 Metallopr  70.9     4.2 3.4E-05   31.5   0.4    8  224-231   111-118 (159)
 20 SUPFAM0038581 d.92.1 Metallopr  70.2     4.6 3.7E-05   31.4   0.5    8  224-231   112-119 (160)
 21 SUPFAM0036188 d.92.1 Metallopr  69.5     4.9   4E-05   31.5   0.5    9  224-232   117-125 (164)
 22 PIRSF005785 Zn-prot_arch        68.9     4.5 3.7E-05   36.8   0.2   10  223-232   149-158 (204)
 23 d1mmqa_ d.92.1.11 (A:) Matrily  68.5      13 0.00011   32.4   2.9   90  141-230    27-124 (166)
 24 SUPFAM0035975 d.92.1 Metallopr  68.5     5.3 4.3E-05   31.5   0.5    9  224-232   116-124 (168)
 25 d1kapp2 d.92.1.6 (P:1-246) Met  68.4      19 0.00016   34.0   4.1   88  143-230    90-180 (246)
 26 SUPFAM0036877 d.92.1 Metallopr  68.1     5.3 4.3E-05   31.4   0.4    9  224-232   118-126 (169)
 27 1cge_A Fibroblast collagenase;  67.8      19 0.00016   31.4   3.8   90  141-230    25-121 (168)
 28 SUPFAM0041006 d.92.1 Metallopr  67.5     5.6 4.6E-05   31.3   0.4    9  224-232   118-126 (166)
 29 pfam04228 Zn_peptidase Putativ  67.2     5.6 4.6E-05   37.9   0.4   12  222-233   172-183 (292)
 30 SUPFAM0035790 d.92.1 Metallopr  66.8     4.9   4E-05   31.3   0.0    9  224-232   117-125 (165)
 31 1bkcA00 3.40.390.10             65.6     6.6 5.4E-05   33.5   0.5    9  224-232   185-193 (255)
 32 SUPFAM0043304 d.92.1 Metallopr  65.5     6.3 5.1E-05   31.1   0.4    9  224-232   121-129 (169)
 33 SUPFAM0045657 d.92.1 Metallopr  64.4     5.4 4.4E-05   31.5  -0.2    9  224-232   117-125 (168)
 34 SUPFAM0035666 d.92.1 Metallopr  64.2     6.9 5.6E-05   31.1   0.4    9  224-232   124-132 (174)
 35 SUPFAM0039592 d.92.1 Metallopr  64.1     5.9 4.8E-05   30.6  -0.0    9  224-232   111-119 (158)
 36 3KEC_Entity_1                   63.7      18 0.00014   31.4   2.8   90  141-230    27-123 (167)
 37 d1hv5a_ d.92.1.11 (A:) Stromel  63.4      34 0.00028   30.0   4.4   88  141-232    26-125 (162)
 38 SUPFAM0040001 d.92.1 Metallopr  63.1     5.6 4.6E-05   34.0  -0.3    9  224-232   174-182 (246)
 39 830c_A MMP-13, MMP-13; matrix   62.9      20 0.00016   31.3   3.0   92  141-232    27-125 (168)
 40 SUPFAM0038130 d.92.1 Metallopr  62.9     5.9 4.8E-05   30.7  -0.2    8  224-231   115-122 (159)
 41 d1hy7a_ d.92.1.11 (A:) Stromel  62.8      21 0.00017   31.1   3.0   90  141-230    27-123 (168)
 42 SUPFAM0035598 d.92.1 Metallopr  62.5     8.6   7E-05   33.1   0.7    9  224-232   185-193 (255)
 43 SUPFAM0038739 d.92.1 Metallopr  62.2     6.4 5.2E-05   30.8  -0.1    9  224-232   117-125 (162)
 44 SUPFAM0040268 d.92.1 Metallopr  61.8     4.9   4E-05   30.0  -0.8    9  224-232    81-89  (132)
 45 pfam12388 Peptidase_M57 Dual-a  61.7      28 0.00023   32.0   3.6  102  144-247    56-163 (211)
 46 PF04228 Zn_peptidase:  Putativ  61.5     8.6   7E-05   37.3   0.5   10  224-233   174-183 (292)
 47 2ovx_A Matrix metalloproteinas  61.2      17 0.00014   31.2   2.2   90  141-230    24-121 (159)
 48 2jsd_A Matrix metalloproteinas  59.5      59 0.00048   28.2   5.1   90  141-230    22-118 (160)
 49 SUPFAM0040267 d.92.1 Metallopr  59.3      11 8.9E-05   31.1   0.7    9  224-232   140-148 (201)
 50 d1rm8a_ d.92.1.11 (A:) Matrix   58.9      11 9.1E-05   32.6   0.7    9  222-230   119-127 (169)
 51 SUPFAM0044993 d.92.1 Metallopr  57.2      13 0.00011   30.6   0.9    9  224-232   138-146 (198)
 52 cd04271 ZnMc_ADAM_fungal Zinc-  57.0     8.1 6.6E-05   35.9  -0.4    9  224-232   149-157 (228)
 53 cd04279 ZnMc_MMP_like_1 Zinc-d  56.8      43 0.00035   29.5   3.9   89  142-230    19-114 (156)
 54 SUPFAM0038010 d.92.1 Metallopr  56.5     8.4 6.8E-05   32.8  -0.4    9  224-232   165-173 (242)
 55 PIRSF020485 PA-IL               56.5      24  0.0002   29.7   2.2   15    4-18     16-30  (123)
 56 1bud_A Protein (acutolysin A);  55.7      14 0.00012   33.3   0.9    9  224-232   137-145 (197)
 57 SUPFAM0035195 d.92.1 Metallopr  55.5      15 0.00012   30.3   0.9    9  224-232   138-146 (200)
 58 d1xuca1 d.92.1.11 (A:104-272)   55.3      14 0.00011   32.3   0.7    9  222-230   115-123 (169)
 59 d1bswa_ d.92.1.9 (A:) Snake ve  55.3      15 0.00012   33.2   0.9    9  224-232   137-145 (197)
 60 SUPFAM0042988 d.92.1 Metallopr  55.2      15 0.00012   30.1   0.8    9  224-232   139-147 (197)
 61 1slm_A Stromelysin-1; hydrolas  55.1      33 0.00027   32.9   3.1   90  141-230   109-205 (255)
 62 d1y93a1 d.92.1.11 (A:106-263)   54.8      15 0.00013   31.5   0.9    9  224-232   111-119 (158)
 63 2w15_A Zinc metalloproteinase   54.7      15 0.00012   33.3   0.9    9  224-232   140-148 (202)
 64 d1bqqm_ d.92.1.11 (M:) Membran  54.3 1.6E+02  0.0013   25.6   7.0   92  141-232    23-132 (174)
 65 SUPFAM0045583 d.92.1 Metallopr  54.3      16 0.00013   30.2   0.8    9  224-232   139-147 (201)
 66 pfam06262 DUF1025 Domain of un  54.2      16 0.00013   29.2   0.9    9  224-232    76-84  (96)
 67 COG3824 Predicted Zn-dependent  53.9      14 0.00011   30.7   0.5    9  224-232   113-121 (136)
 68 1c7k_A NCNP, zinc endoprotease  53.5      16 0.00013   31.0   0.8    7  224-230    81-87  (132)
 69 d2ejqa1 d.92.1.17 (A:2-108) Un  52.7      17 0.00014   29.9   0.7    9  224-232    92-100 (107)
 70 d2ovxa1 d.92.1.11 (A:110-443)   52.2      18 0.00015   31.2   0.9    9  224-232   115-123 (159)
 71 1qua_A Acutolysin-C, hemorrhag  52.1      18 0.00015   32.7   0.9    9  224-232   139-147 (197)
 72 cd04267 ZnMc_ADAM_like Zinc-de  51.9      16 0.00013   33.1   0.6    9  224-232   137-145 (192)
 73 3b8z_A Protein adamts-5; alpha  51.7      18 0.00015   33.1   0.9    9  224-232   145-153 (217)
 74 d1r55a_ d.92.1.9 (A:) ADAM33 {  51.6      18 0.00015   32.8   0.9    9  224-232   136-144 (203)
 75 3ma2_D Matrix metalloproteinas  51.6      19 0.00015   31.6   0.9    9  224-232   126-134 (181)
 76 d1qiba_ d.92.1.11 (A:) Gelatin  51.4      18 0.00015   31.2   0.8    7  224-230   113-119 (161)
 77 SUPFAM0043540 d.92.1 Metallopr  50.5     9.9   8E-05   32.2  -0.9    7  224-230   171-177 (243)
 78 PF07828 PA-IL:  PA-IL-like pro  50.5      12 9.9E-05   30.0  -0.3   15    4-18     14-28  (121)
 79 SUPFAM0041314 d.92.1 Metallopr  50.5      19 0.00016   29.6   0.9    9  224-232   140-148 (202)
 80 2ejq_A Hypothetical protein TT  50.5      19 0.00015   30.7   0.7    9  224-232    93-101 (130)
 81 1yp1_A FII; FII hydrolase; 1.9  50.1      20 0.00016   32.7   0.9    9  224-232   139-147 (202)
 82 d1kufa_ d.92.1.9 (A:) Snake ve  50.1      20 0.00016   32.6   0.9    9  224-232   140-148 (201)
 83 d1quaa_ d.92.1.9 (A:) Snake ve  50.1      20 0.00016   32.4   0.9    9  224-232   139-147 (197)
 84 PF00413 Peptidase_M10:  Matrix  50.0      17 0.00014   31.3   0.5    9  224-232   106-114 (152)
 85 1kuf_A Atrolysin E, metallopro  49.8      20 0.00017   32.6   0.9    9  224-232   142-150 (203)
 86 d1nd1a_ d.92.1.9 (A:) Snake ve  49.8      20 0.00017   32.6   0.9    9  224-232   140-148 (202)
 87 PF01433 Peptidase_M1:  Peptida  49.7 1.9E+02  0.0015   29.6   7.5  100  141-258   227-335 (391)
 88 1rm8_A MMP-16, matrix metallop  49.3      20 0.00016   31.1   0.7    9  224-232   121-129 (169)
 89 d1sata2 d.92.1.6 (A:4-246) Met  49.2      75 0.00061   30.2   4.4   86  143-230    80-177 (243)
 90 d3b7sa3 d.92.1.13 (A:209-460)   48.8 2.1E+02  0.0017   27.2   7.3  114  121-259     6-121 (252)
 91 COG1164 Oligoendopeptidase F [  48.7      48 0.00039   36.1   3.4   82  129-233   312-393 (598)
 92 3ba0_A Macrophage metalloelast  48.5      55 0.00044   33.1   3.6   90  141-230    21-117 (365)
 93 1k7iA02 3.40.390.10             48.1      52 0.00042   27.5   3.0   88  143-230    64-157 (227)
 94 cd04272 ZnMc_salivary_gland_MP  47.9      20 0.00016   33.3   0.5    9  224-232   149-157 (220)
 95 COG3590 PepO Predicted metallo  47.7      22 0.00018   37.6   0.9   20  224-243   491-510 (654)
 96 d1atla_ d.92.1.9 (A:) Snake ve  47.4      23 0.00019   32.1   0.9    9  224-232   138-146 (200)
 97 2ddf_A ADAM 17; hydrolase; HET  47.0      24 0.00019   33.5   0.9    9  224-232   186-194 (257)
 98 cd04273 ZnMc_ADAMTS_like Zinc-  47.0      23 0.00019   32.7   0.8    9  224-232   144-152 (207)
 99 1atl_A Atrolysin C; metalloend  46.1      25 0.00021   32.0   0.9    9  224-232   140-148 (202)
100 COG1913 Predicted Zn-dependent  45.4      26 0.00021   31.3   0.9   10  223-232   127-136 (181)

  No 1  
>template EXPERIMENTAL AUTOIMMUNE PROSTATITIS ANTIGEN 2-RELATED.
  Probab=100.00  E-value=0  Score=389.42  Aligned_cols=282  Identities=21%  Similarity=0.275  Sum_probs=0.0

  Q ss_pred             cccceEEECCCCEEEEEecC--CCCcEEEEEeecCCCcccccccccCCCCceEEEEecCCeEEEecCCCcEEEEEcCCCC
  Q Q5E705_VIBF1/1    3 RQSAGVWIPAREVAYVHGLS--SDDTVMIAMADNLTGRVNHEMALNRPPRVSMSFNGVEASNGFKVPYGGSVYITLGSKE   80 (302)
  Q Consensus         3 ~~~TG~y~~~Ge~i~V~~~~--~~~~~~v~i~~~~~~~~~~~~~~~r~~~~~~~~~L~~g~n~i~~p~GG~iyi~~~~~~   80 (302)
                        |.|||+|++.++.+.|.++.  ...++++||||| .|+++....+.|.|.+....-|+.....|++-+||++||.++.+.
  T Consensus       546 W~STGLY~~~~q~~~v~~pe~Aasa~L~~qiGCh-tDdlt~A~kl~R~P~v~~~C~ld~~~~S~~ClWGGLlYiiVp~~~  624 (928)
  T PTHR15730       546 WMSTGLYLPGRQVIDVSLPESAASADLKVQIGCH-TDDLTRASKLFRGPLVINRCCLDKPTKSITCLWGGLLYIIVPQNS  624 (928)
  T ss_pred             eeecceeccCceeEEEEechhhhcCCceEEEeec-ccchhHHHHhhcCceeeeeeeeccccceeeeeecceeEEEeeccc


  Q ss_pred             c---eEEEEecceEECceeecCCCCHHHHHHHHHhCCCCEEEEEcCcEEEEEEHHHHhhhcccCHHHHHHHHHHHHHHHH
  Q Q5E705_VIBF1/1   81 S---AQVSFGGSAIAAPMFMMTSATEGSWITTPEESDAPITEIVGKRFSYTTTTAGIKGHSEVDVLEMTKQFDLFTIGVN  157 (302)
  Q Consensus        81 ~---v~v~i~~~~~~~P~f~~g~~t~~~w~~~l~~~~~p~~ei~~~~v~~t~p~~~~~~~~~~d~~~l~~~~d~ii~~~~  157 (302)
                        +   |.++|.| ++++|+|++|+|+.+||++.+.++++||.|+.+|++++|+|+..++.+  +||..+++.||+||++..
  T Consensus       625 ~lG~vp~t~~g-Av~AP~yklG~Ts~eeWk~~~~~~~gPWGELaTdNiILTvPt~nl~~L--~~PeplL~LWDemmqAva  701 (928)
  T PTHR15730       625 KLGSVPVTVKG-AVRAPFYKLGETSKEEWKRRLLEYPGPWGELATDNIILTVPTANLRTL--ENPEPLLRLWDEMMQAVA  701 (928)
  T ss_pred             cccceeEEEee-ccccchhhcCCCcHHHHHHHHHhcCCCchhhhhcceeeeccchhhhhh--cCchHHHHHHHHHHHHHH


  Q ss_pred             HHhCCCCCCccccccccccccccchhhceeeeeccccceecCCcceeeecccccc---ceeccCCCcchhHHhhhhhccc
  Q Q5E705_VIBF1/1  158 EFYGRDGVSGAHKMFTDSAPELEYQNMRLVDDIQISIGSAHSGYPVMSTSFPRQK---SSLFKATDNWMLGHEIGHNQAA  234 (302)
  Q Consensus       158 ~l~Gl~~~~~~~~~~~~~~~~~~~~~~~~~~~~~~~~~~~~~g~~~~~~~~~~~~---~~~~~~~~~WG~~HEiGH~~Q~  234 (302)
                        +|.|.+..-.+|+              |+|+|+|++.||||+||||||+..+...   ...+.+.|-||+.||+|||+|.
  T Consensus       702 ~L~~~~fpl~~p~--------------RiVaDvQiS~GWmH~GYPImChlesv~eli~e~~ir~~G~WGp~HELG~NqQ~  767 (928)
  T PTHR15730       702 KLGGEPFPLRLPE--------------RIVADVQISVGWMHAGYPIMCHLESVQELINEKLIRTKGLWGPVHELGRNQQR  767 (928)
  T ss_pred             HhcCCCcccccch--------------heeeehhhccchhhccchHHHHHHHHHHHHhHHHHhhcCCcccccccCCcccc


  Q ss_pred             cccccCC-CccchhhHHHHHHHHHHhc-------cccchhhhHHHHHHhhccCcccccCCHHHHHHHHHHHHHhcC
  Q Q5E705_VIBF1/1  235 NWLNVVG-AGETANNVLALYTQERNTG-------DMPRIKVSITNATEWANGDHPWADGTNADRLNFFGQLKLWAE  302 (302)
  Q Consensus       235 ~~~~~~g-~~EvtnNi~sl~~q~~~~~-------~~~~~~~~~~~~~~~~~~~~~~~~~~~~~~L~~f~QL~~~~G  302 (302)
                        ..|.+++ +||.|||+++.|+.+...+       ..+....|..+++.|+..+.....++.|++|..|.||+++||
  T Consensus       768 ~~WefpPHTTEAtCNLW~vYVHETVLGIPR~~A~~~L~pp~Re~r~~~yL~kG~~~~~Wn~WTALETYLQLqe~FG  843 (928)
  T PTHR15730       768 QEWEFPPHTTEATCNLWCVYVHETVLGIPRSRANIALWPPVREKRVRIYLSKGPSVKHWNAWTALETYLQLQEAFG  843 (928)
  T ss_pred             ccccCCCCcchhhhhheeeeehhhhccCChhhhhhhcCchhHHHHHHHHhccCCchhhHHHHHHHHHHHHHHHhhc


  No 2  
>PF03272 Enhancin:  Viral enhancin protein;  InterPro:   Metalloproteases are the most diverse of the
  four main types of protease, with more than 50 families identified to date. In these enzymes, a divalent cation,
  usually zinc, activates the water molecule. The metal ion is held in place by amino acid ligands, usually
  three in number. The known metal ligands are His, Glu, Asp or Lys and at least one other residue is required for
  catalysis, which may play an electrophillic role. Of the known metalloproteases, around half contain an HEXXH
  motif, which has been shown in crystallographic studies to form part of the metal-binding site . The HEXXH motif
  is relatively common, but can be more stringently defined for metalloproteases as 'abXHEbbHbc', where 'a' is
  most often valine or threonine and forms part of the S1' subsite in thermolysin and neprilysin, 'b' is an
  uncharged residue, and 'c' a hydrophobic residue. Proline is never found in this site, possibly because it would break
  the helical structure adopted by this motif in metalloproteases .   Peptidases are grouped into clans and
  families. Clans are groups of families for which there is evidence of common ancestry. Each clan is identified with
  two letters, the first representing the catalytic type of the families included in the clan (with the letter
  'P' being used for a clan containing families of more than one of the catalytic types serine, threonine and
  cysteine). Some families cannot yet be assigned to clans, and when a formal assignment is required, such a family is
  described as belonging to clan A-, C-, M-, S-, T- or U-, according to the catalytic type. Some clans are
  divided into subclans because there is evidence of a very ancient divergence within the clan, for example MA(E), the
  gluzincins, and MA(M), the metzincins.   Families are grouped by their catalytic type, the first character
  representing the catalytic type: A, aspartic; C, cysteine; G, glutamic acid; M, metallo; S, serine; T, threonine;
  and U, unknown. The serine, threonine and cysteine peptidases utilise the amino acid as a nucleophile and form
  an acyl intermediate - these peptidases can also readily act as transferases. In the case of aspartic, glutamic
  and metallopeptidases, the nucleophile is an activated water molecule.    This group of metallopeptidases
  belong to the MEROPS peptidase family M60 (enhancin family, clan MA(E)). The active site residues for members of
  this family and thermolysin, the type example for clan MA, occur in the motif HEXXH. The viral enhancin protein,
  or enhancing factor, is involved in disruption of the peritrophic membrane and fusion of nucleocapsids with
  mid-gut cells. ; GO: 0016032 viral reproduction
  Probab=100.00  E-value=2.2e-37  Score=307.77  Aligned_cols=281  Identities=16%  Similarity=0.127  Sum_probs=0.0

  Q ss_pred             CCcccceEEECCCCEEEEEe--cCCCCcEEEEEeecCCCcccccccccCCCCceEEEEecCCeEEEecCCCcEEEEEcCC
  Q Q5E705_VIBF1/1    1 GSRQSAGVWIPAREVAYVHG--LSSDDTVMIAMADNLTGRVNHEMALNRPPRVSMSFNGVEASNGFKVPYGGSVYITLGS   78 (302)
  Q Consensus         1 ~~~~~TG~y~~~Ge~i~V~~--~~~~~~~~v~i~~~~~~~~~~~~~~~r~~~~~~~~~L~~g~n~i~~p~GG~iyi~~~~   78 (302)
                        |+|||.|++++||..|+|+.  ++....+++++.+....++.             ++.+..+.++++++.-...+|..+-
  T Consensus        27 H~R~~lg~il~a~~~i~ir~~~~~~~~~~tlrlLNnd~~tE~-------------s~~~~~~~~~~~~~~~sVpFvd~~~   93 (775)
  T PF03272_consen   27 HDRQPLGYILPANTKIRIRQNNPNFVGPLTLRLLNNDRNTEK-------------SITVNNEWVTISVQHDSVPFVDTPY   93 (775)
  T ss_pred             cCCccCCEEECCCCEEEEEecCCCCCCCeEEEEecCCccceE-------------EEEecCccEEEEcccceEEEEeeee


  Q ss_pred             ---CCc---eEEEEecceEECceeecCCCCHHHHHHHHHhCCCCEEEEEcCcEEEEEEHHHHhhhc---ccCHHHHHHHH
  Q Q5E705_VIBF1/1   79 ---KES---AQVSFGGSAIAAPMFMMTSATEGSWITTPEESDAPITEIVGKRFSYTTTTAGIKGHS---EVDVLEMTKQF  149 (302)
  Q Consensus        79 ---~~~---v~v~i~~~~~~~P~f~~g~~t~~~w~~~l~~~~~p~~ei~~~~v~~t~p~~~~~~~~---~~d~~~l~~~~  149 (302)
                           ..+   |++.|.|...++|+|+.|.++ ++|++++++..+|+|.|+++++++++|..+.+.+.   ..|+++|+++|
  T Consensus        94 ~~~~~~~~~V~~~i~~~~~~LP~y~~g~~~-~~F~~~~~~~~~~fa~le~~~i~lLVP~~dk~~l~~~~~~~l~~L~~~Y  172 (775)
  T PF03272_consen   94 GDNSDGEYEVEYEITGEHKPLPVYRKGQNE-SDFFSEWDDSDSPFAFLEGDRIQLLVPPADKNYLRNKDDTDLDELNDFY  172 (775)
  T ss_pred             cCCCCCcEEEEEEeCCCccccCEEEeCCCH-HHHHHhhhhcCCceEEEEcCeEEEEeCHhHHHHHhhhccCCHHHHHHHH


  Q ss_pred             HHHHHHHHHHhCCCCCCccccccccccccccchhhceeeeeccccceecCCcceeeeccccccceeccCCCcchhHHhhh
  Q Q5E705_VIBF1/1  150 DLFTIGVNEFYGRDGVSGAHKMFTDSAPELEYQNMRLVDDIQISIGSAHSGYPVMSTSFPRQKSSLFKATDNWMLGHEIG  229 (302)
  Q Consensus       150 d~ii~~~~~l~Gl~~~~~~~~~~~~~~~~~~~~~~~~~~~~~~~~~~~~~g~~~~~~~~~~~~~~~~~~~~~WG~~HEiG  229 (302)
                        ++|++.|++++||+.++..+.+.+..+.++      +++|..+.|+++|++++++.+.++.. .++.....+||+|||||
  T Consensus       173 ~~Ii~~Yd~l~GL~~~~~~~~~~n~~~kyF------~KAD~~G~G~AYY~~~~~a~s~~s~~-~~L~~~~~nWg~LHEiG  245 (775)
  T PF03272_consen  173 NEIISFYDDLTGLSDDPSMPVDSNFNRKYF------AKADKSGPGAAYYGSNWTANSSSSMS-FYLNPSPTNWGALHEIG  245 (775)
  T ss_pred             HHHHHHHHhhcccccCCcccccccccceeE------EecCCCCCCccccccceeecChHHHH-HHhCcCCccchhHhhhh


  Q ss_pred             hhcccccc-ccCCCccchhhHHHHHHHHHHhcc-----------ccchhhhHHHHHHhhccCcccccCCHHHHHHHHH-H
  Q Q5E705_VIBF1/1  230 HNQAANWL-NVVGAGETANNVLALYTQERNTGD-----------MPRIKVSITNATEWANGDHPWADGTNADRLNFFG-Q  296 (302)
  Q Consensus       230 H~~Q~~~~-~~~g~~EvtnNi~sl~~q~~~~~~-----------~~~~~~~~~~~~~~~~~~~~~~~~~~~~~L~~f~-Q  296 (302)
                        |.||.+++ ++..++||||||||+++|+.+.+.           .-+.+.....+.+.+....++.+++.+.||.||+ +
  T Consensus       246 H~yd~~F~~n~~~~~EVw~NI~~d~yQ~~~~~~~e~~~~~wly~~G~r~~ve~~i~~~~~~~~~~~~w~~~~kL~~~~~~  325 (775)
  T PF03272_consen  246 HGYDFGFTRNDPYLGEVWNNILADRYQYTYMNPDERQQLGWLYDNGKRERVERNINNLIDNNKPFDSWDLREKLIFFTWI  325 (775)
  T ss_pred             hhhcceeeecCCeeeeeehhhhHHHHHHHhcCHHHhhccchhhcCCCHHHHHHHHHHHhhcCCCcccccHHHHHHHHHHH


  Q ss_pred             HHHhcC
  Q Q5E705_VIBF1/1  297 LKLWAE  302 (302)
  Q Consensus       297 L~~~~G  302 (302)
                        |...+|
  T Consensus       326 l~~~~G  331 (775)
  T PF03272_consen  326 LNTKAG  331 (775)
  T ss_pred             HHHHHh


  No 3  
>pfam03272 Enhancin Viral enhancin protein.
  Probab=100.00  E-value=1.3e-33  Score=277.70  Aligned_cols=287  Identities=14%  Similarity=0.055  Sum_probs=0.0

  Q ss_pred             CCcccceEEECCCCEEEEEe--cCCCCcEEEEEeecCCCcccccccccCCCCceEEEEecCCeEEEecCCCcEEEEEcCC
  Q Q5E705_VIBF1/1    1 GSRQSAGVWIPAREVAYVHG--LSSDDTVMIAMADNLTGRVNHEMALNRPPRVSMSFNGVEASNGFKVPYGGSVYITLGS   78 (302)
  Q Consensus         1 ~~~~~TG~y~~~Ge~i~V~~--~~~~~~~~v~i~~~~~~~~~~~~~~~r~~~~~~~~~L~~g~n~i~~p~GG~iyi~~~~   78 (302)
                        |+|||.|++++||..|+|+.  ++....+++++.+.....+.+......|..++..   ...+-.|.+|+||..    +.
  T Consensus        27 H~R~plg~il~a~~~i~ir~~~~~~~~~~tlrlLNnn~~tE~si~v~~~w~~~~~~---~~sVpFvd~~~~~~~----~~   99 (775)
  T pfam03272        27 HYRQPLGYVLKAGSTIRLRTNNAAPVGPVTLRLLNNNRNTERSITVTNDWTTLTVE---HDSVPFVDTVVVDNR----DG   99 (775)
  T ss_pred             cCCcccCEEECCCCEEEEEeccCCCCCCeEEEEeeCCccceEEEEecCceEEEEec---cceEEEEeccccCCC----Cc


  Q ss_pred             CCceEEEEecceEECceeecCCCCHHHHHHHHHhCCCCEEEEEcCcEEEEEEHHHHhhhc---ccCHHHHHHHHHHHHHH
  Q Q5E705_VIBF1/1   79 KESAQVSFGGSAIAAPMFMMTSATEGSWITTPEESDAPITEIVGKRFSYTTTTAGIKGHS---EVDVLEMTKQFDLFTIG  155 (302)
  Q Consensus        79 ~~~v~v~i~~~~~~~P~f~~g~~t~~~w~~~l~~~~~p~~ei~~~~v~~t~p~~~~~~~~---~~d~~~l~~~~d~ii~~  155 (302)
                        ...|++.|.|...++|+|+.|.+. ++|++++++..+|+|.|++++|++++|..+...+.   ..++++|.++|++|++.
  T Consensus       100 ~~~ve~~i~g~~~~LP~y~~g~~~-~~f~~~~~~~~~~fa~ve~~~i~lLVP~~Dk~~l~~~~~~~i~~L~~fY~~I~~~  178 (775)
  T pfam03272       100 QYEVEYEIDGEHTPLPVYTFGQNE-QEFKSEWRDSNSSYAFVELKYIQLLVPPADKNALLNMNDDDLDELDDFYNSIVSF  178 (775)
  T ss_pred             cEEEEEEECCccccCCEEecCCCh-HHHHhhhhhcCCceEEEECCeEEEEeCcchHHHHhccccCCHHHHHHHHHHHHHH


  Q ss_pred             HHHHhCCCCCCccccccccccccccchhhceeeeeccccceecCCcceeeeccccccceeccCCCcchhHHhhhhhcccc
  Q Q5E705_VIBF1/1  156 VNEFYGRDGVSGAHKMFTDSAPELEYQNMRLVDDIQISIGSAHSGYPVMSTSFPRQKSSLFKATDNWMLGHEIGHNQAAN  235 (302)
  Q Consensus       156 ~~~l~Gl~~~~~~~~~~~~~~~~~~~~~~~~~~~~~~~~~~~~~g~~~~~~~~~~~~~~~~~~~~~WG~~HEiGH~~Q~~  235 (302)
                        |++++||+.+..++.+.+..+.++      +++|..++||++|++++++.+.++.. .|+.+...+||++|||||.||..
  T Consensus       179 Yd~l~Gl~~~~~~~~~~n~~~kyF------~KAD~~G~G~AYY~~~w~a~s~~sl~-~yL~~s~~NW~~LHEIgH~Yd~~  251 (775)
  T pfam03272       179 YDDLTGLSFDPTAAVDINFNKKYF------AKADASGPGAAYYGPNWIAESSSSLG-FYLNPSPTNWGVLHEIGHAYDFY  251 (775)
  T ss_pred             HhhhhCCccCcccccccccccceE------EEecCCCCCcccccccceeccchhHH-HHhCCCCccchhhhhhhhhccee


  Q ss_pred             cc-ccCCCccchhhHHHHHHHHHHhcccc-----------chhhhHHHHHHhhccCcccccCCHHHHHHHHHHHHHhcC
  Q Q5E705_VIBF1/1  236 WL-NVVGAGETANNVLALYTQERNTGDMP-----------RIKVSITNATEWANGDHPWADGTNADRLNFFGQLKLWAE  302 (302)
  Q Consensus       236 ~~-~~~g~~EvtnNi~sl~~q~~~~~~~~-----------~~~~~~~~~~~~~~~~~~~~~~~~~~~L~~f~QL~~~~G  302 (302)
                        +. +-..+.|||||||+...|+.+.+.+.           +.+.....+.+.+....++.+|+.+.||.+|-.|..-.|
  T Consensus       252 F~~n~~~l~EVWnNil~d~yQy~~~~~~Erq~~~wiy~~G~r~~ve~~I~~~i~~~~~~~~w~~r~kL~~lt~l~~~~~  330 (775)
  T pfam03272       252 FTRNHTYLIEVWNNILADRYQYDWMTPAERQTDAWIYDNGRRDRVETNIIALIDTNKPFNSWSLRERLIFLTWLMNTKA  330 (775)
  T ss_pred             EecCCcchhHhhhhhhhHHHhHhhCChHHhhhhcceecCCchHHHHHHHHHHHhcCCCcccccHHHHHHHHHHHHHhhh


  No 4  
>pfam07828 PA-IL PA-IL-like protein. The members of this family are similar to the galactophilic lectin-1
  expressed by P. aeruginosa ((PA-IL). Lectins recognising specific carbohydrates found on the surface of host cells are
  known to be involved in the initiation of infections by this organism. The protein is thought to be organized
  into an extensive network of beta-sheets, as is the case with many other lectins.
  Probab=88.84  E-value=6.1  Score=31.96  Aligned_cols=85  Identities=7%  Similarity=0.039  Sum_probs=0.0

  Q ss_pred             ccceEEECCCCEEEE------EecCCCCcEEEEEeecCCCcccccccc---cCCCCceEEEEecCCeEEEecC---CCcE
  Q Q5E705_VIBF1/1    4 QSAGVWIPAREVAYV------HGLSSDDTVMIAMADNLTGRVNHEMAL---NRPPRVSMSFNGVEASNGFKVP---YGGS   71 (302)
  Q Consensus         4 ~~TG~y~~~Ge~i~V------~~~~~~~~~~v~i~~~~~~~~~~~~~~---~r~~~~~~~~~L~~g~n~i~~p---~GG~   71 (302)
                        |||||.+.+|+.|+|      +-.....-+..--|.+....+......   =....-.+.|.+-.|+-.=++|   .-|.
  T Consensus        14 q~Tglilk~GDiIsIvA~GW~kyG~~~~~~aapqg~~p~~g~~~~~~~~~~LvaKIgnk~y~igNg~Lhktvpp~~vdGe   93 (121)
  T pfam07828        14 KSTGIILKPGDTISIVAAGWAKYGPDNKEGAAGDGEHPDNGLICHDAFCAALVAKIANKGFAIGNGGLFKTVAPNNVDGA   93 (121)
  T ss_pred             CcceeEEcCCCEEEEeeechhhcCCCCCcccCCCCcCCCCCcccCcccchhhhhhhccccccccCceeeeecCCcccCce


  Q ss_pred             EEEEcCCCCc----------eEEEEec
  Q Q5E705_VIBF1/1   72 VYITLGSKES----------AQVSFGG   88 (302)
  Q Consensus        72 iyi~~~~~~~----------v~v~i~~   88 (302)
                        |-+.+.+.+.          |+|.|.+
  T Consensus        94 lillf~D~pg~ygdNSgeF~V~V~ies  120 (121)
  T pfam07828        94 IILIFNDVPGTFGDNSGEFQVEIGIDQ  120 (121)
  T ss_pred             EEEEEeCCCCcccCCCccEEEEEEEec


  No 5  
 >3N2V_Entity_1
  Probab=80.20  E-value=5.1  Score=34.36  Aligned_cols=90  Identities=11%  Similarity=-0.278  Sum_probs=0.0

  Q ss_pred             CHHHHHHHHHHHHHHHHHHhCCCCCCccccccccccccccchhhceeeeeccccceecCCcceeeeccccccceeccCCC
  Q Q5E705_VIBF1/1  141 DVLEMTKQFDLFTIGVNEFYGRDGVSGAHKMFTDSAPELEYQNMRLVDDIQISIGSAHSGYPVMSTSFPRQKSSLFKATD  220 (302)
  Q Consensus       141 d~~~l~~~~d~ii~~~~~l~Gl~~~~~~~~~~~~~~~~~~~~~~~~~~~~~~~~~~~~~g~~~~~~~~~~~~~~~~~~~~  220 (302)
                        ...+.-+...+.++...+.++++..............................+...++..+-.................
  T Consensus        21 ~~~~~~~~i~~A~~~W~~~~~i~F~~~~~~~~~~~~~~~~~~~~~~~~~~~~~g~~~~~~~~~~~~~~~~~~~~~~~~~~  100 (158)
  T 3N2V_Entity_1    21 NREDVDYAIRKAFQVWSNVTPLKFSKINTGMADILVVFARGAHGDDHAFDGKGGILAHAFGPGSGIGGDAHFDEDEFWTT  100 (158)
  T ss_pred             CHHHHHHHHHHHHHHHHhhcCeeEEEeccCCceEEEEEeecccCcccccccccceeeeccCCCCceeeeEEeecceeeee


  Q ss_pred             cch-------hHHhhhh
  Q Q5E705_VIBF1/1  221 NWM-------LGHEIGH  230 (302)
  Q Consensus       221 ~WG-------~~HEiGH  230 (302)
                        ..+       +.|||||
  T Consensus       101 ~~~~~~~~~v~~HEiGH  117 (158)
  T 3N2V_Entity_1   101 HSGGTNLFLTAVHEIGH  117 (158)
  T ss_pred             ccCCccceeeehhhhcc


  No 6  
 >3N2U_Entity_1
  Probab=80.20  E-value=5.1  Score=34.36  Aligned_cols=90  Identities=11%  Similarity=-0.278  Sum_probs=0.0

  Q ss_pred             CHHHHHHHHHHHHHHHHHHhCCCCCCccccccccccccccchhhceeeeeccccceecCCcceeeeccccccceeccCCC
  Q Q5E705_VIBF1/1  141 DVLEMTKQFDLFTIGVNEFYGRDGVSGAHKMFTDSAPELEYQNMRLVDDIQISIGSAHSGYPVMSTSFPRQKSSLFKATD  220 (302)
  Q Consensus       141 d~~~l~~~~d~ii~~~~~l~Gl~~~~~~~~~~~~~~~~~~~~~~~~~~~~~~~~~~~~~g~~~~~~~~~~~~~~~~~~~~  220 (302)
                        ...+.-+...+.++...+.++++..............................+...++..+-.................
  T Consensus        21 ~~~~~~~~i~~A~~~W~~~~~i~F~~~~~~~~~~~~~~~~~~~~~~~~~~~~~g~~~~~~~~~~~~~~~~~~~~~~~~~~  100 (158)
  T 3N2U_Entity_1    21 NREDVDYAIRKAFQVWSNVTPLKFSKINTGMADILVVFARGAHGDDHAFDGKGGILAHAFGPGSGIGGDAHFDEDEFWTT  100 (158)
  T ss_pred             CHHHHHHHHHHHHHHHHhhcCeeEEEeccCCceEEEEEeecccCcccccccccceeeeccCCCCceeeeEEeecceeeee


  Q ss_pred             cch-------hHHhhhh
  Q Q5E705_VIBF1/1  221 NWM-------LGHEIGH  230 (302)
  Q Consensus       221 ~WG-------~~HEiGH  230 (302)
                        ..+       +.|||||
  T Consensus       101 ~~~~~~~~~v~~HEiGH  117 (158)
  T 3N2U_Entity_1   101 HSGGTNLFLTAVHEIGH  117 (158)
  T ss_pred             ccCCccceeeehhhhcc


  No 7  
>1l7l_A PA-I galactophilic lectin; agglutinin, single wavelength anomalous scattering phasing, structural
  genomics, PSI; 1.50A {Pseudomonas aeruginosa} SCOP: b.18.1.16 PDB:  1oko _A*  1uoj _A  2vxj _A*  2wyf _A*
  Probab=78.63  E-value=3.8  Score=33.02  Aligned_cols=15  Identities=13%  Similarity=0.184  Sum_probs=0.0

  Q ss_pred             ccceEEECCCCEEEE
  Q Q5E705_VIBF1/1    4 QSAGVWIPAREVAYV   18 (302)
  Q Consensus         4 ~~TG~y~~~Ge~i~V   18 (302)
                        |||||.+.+|+.|+|
  T Consensus        14 q~Tglilk~GDiIsI   28 (121)
  T 1l7l_A           14 QVTSIIYNPGDVITI   28 (121)
  T ss_dssp             EEEEEEECTTCCEEE
  T ss_pred             CeeeEEecCCCEEEE


  No 8  
>1qib_A Protein (gelatinase A); inhibitor, matrixin, matrix metalloproteinase-2 (MMP-2), metzincin, hydrolase;
  2.80A {Homo sapiens} SCOP: d.92.1.11 PDB:  1hov _A*
  Probab=78.35  E-value=3.9  Score=35.24  Aligned_cols=90  Identities=10%  Similarity=-0.239  Sum_probs=0.0

  Q ss_pred             CHHHHHHHHHHHHHHHHHHhCCCCCCccccccccccccccchhhceeeeeccccceecCCcceeeeccccccceeccCCC
  Q Q5E705_VIBF1/1  141 DVLEMTKQFDLFTIGVNEFYGRDGVSGAHKMFTDSAPELEYQNMRLVDDIQISIGSAHSGYPVMSTSFPRQKSSLFKATD  220 (302)
  Q Consensus       141 d~~~l~~~~d~ii~~~~~l~Gl~~~~~~~~~~~~~~~~~~~~~~~~~~~~~~~~~~~~~g~~~~~~~~~~~~~~~~~~~~  220 (302)
                        +..++-+...+.++...+.+++...................................+......................
  T Consensus        22 ~~~~~~~ai~~A~~~W~~~~~i~F~~~~~~~ad~~~~~~~~~~~~~~~~~~~~~~~~~~~~~~~~~~~~~~~~~~~~~~~  101 (161)
  T 1qib_A           22 DPETVDDAFARAFQVWSDVTPLRFSRIHDGEADIMINFGRWEHGDGYPFDGKDGLLAHAFAPGTGVGGDSHFDDDELWSL  101 (161)
  T ss_dssp             CHHHHHHHHHHHHHHHHTSSSCEEEECSSSCCSEEEEEECSCCSSSCCCCSSSSCCEEECCSCSTTTTCEEEETTSCEES
  T ss_pred             CHHHHHHHHHHHHHHHhcccCceEEEeccCCCcEEEeeccccccccccccCccccccccccCCCccccceeecccccccc


  Q ss_pred             cch--------hHHhhhh
  Q Q5E705_VIBF1/1  221 NWM--------LGHEIGH  230 (302)
  Q Consensus       221 ~WG--------~~HEiGH  230 (302)
                        .+.        +.|||||
  T Consensus       102 ~~~~~~~~~~v~~HEiGH  119 (161)
  T 1qib_A          102 GKGVGYSLFLVAAHEFGH  119 (161)
  T ss_dssp             SSSSSEEHHHHHHHHHHH
  T ss_pred             ccccCcceeeeeeeehhh


  No 9  
>1i76_A MMP-8;, neutrophil collagenase; hydrolase, complex (metalloprotease/inhibitor); HET: BSI; 1.20A {Homo
  sapiens} SCOP: d.92.1.11 PDB:  1i73 _A*  1jao _A*  1jap _A  1jaq _A*  1jj9 _A*  1mmb _A*  1zp5 _A*  1zs0 _A*  1zvx _A*  3dng _A*
   3dpe _A*  3dpf _A*  1kbc _A*  1jan _A*  1bzs _A*  1mnc _A*  2oy2 _A  1a86 _A*  1jh1 _A*  1a85 _A ...
  Probab=76.88  E-value=9.9  Score=33.27  Aligned_cols=86  Identities=12%  Similarity=-0.072  Sum_probs=0.0

  Q ss_pred             CHHHHHHHHHHHHHHHHHHhCCCCCCccccccccccccccchhhceeeeeccccceecCCcceeeeccccccceeccCCC
  Q Q5E705_VIBF1/1  141 DVLEMTKQFDLFTIGVNEFYGRDGVSGAHKMFTDSAPELEYQNMRLVDDIQISIGSAHSGYPVMSTSFPRQKSSLFKATD  220 (302)
  Q Consensus       141 d~~~l~~~~d~ii~~~~~l~Gl~~~~~~~~~~~~~~~~~~~~~~~~~~~~~~~~~~~~~g~~~~~~~~~~~~~~~~~~~~  220 (302)
                        +..+.-+...+.++...+.+++...............+...............+...+.    ..+.........++...
  T Consensus        26 ~~~~~~~~i~~A~~~W~~v~~i~F~ev~~~~~di~i~~~~~~~~~~~~~~~~~~~~~~~----~~~~~~~~~~i~~~~~~  101 (163)
  T 1i76_A           26 SEAEVERAIKDAFELWSVASPLIFTRISQGEADINIAFYQRDHGDNSPFDGPNGILAHA----FQPGQGIGGDAHFDAEE  101 (163)
  T ss_dssp             CHHHHHHHHHHHHHHHHTTSSCEEEECSSSCCSEEEEEECSCCSSSCCCCSSSSCCEEE----CCSSSTTTTCEEEETTS
  T ss_pred             CHHHHHHHHHHHHHHHhCcCCcEEEEeccCCCcEEEEEeccccccccccCCcccceecc----cCCCCceeeEEecChHh


  Q ss_pred             cc-----------hhHHhhhh
  Q Q5E705_VIBF1/1  221 NW-----------MLGHEIGH  230 (302)
  Q Consensus       221 ~W-----------G~~HEiGH  230 (302)
                        .|           -+.|||||
  T Consensus       102 ~~~~~~~~~~~~~v~~HEIGH  122 (163)
  T 1i76_A          102 TWTNTSANYNLFLVAAHEFGH  122 (163)
  T ss_dssp             CCBSSSSSCBHHHHHHHHHHH
  T ss_pred             ccccccccceeEEEeeehhcc


  No 10 
>1hv5_A Stromelysin 3; inhibition, phosphinic inhibitor, hydrolase; HET: CPS RXP; 2.60A {Mus musculus} SCOP:
  d.92.1.11
  Probab=76.47  E-value=11  Score=32.51  Aligned_cols=90  Identities=11%  Similarity=-0.091  Sum_probs=0.0

  Q ss_pred             CHHHHHHHHHHHHHHHHHHhCCC--CCCccccccccccccccchhhceeeeeccccceecCCcceeeeccccccceeccC
  Q Q5E705_VIBF1/1  141 DVLEMTKQFDLFTIGVNEFYGRD--GVSGAHKMFTDSAPELEYQNMRLVDDIQISIGSAHSGYPVMSTSFPRQKSSLFKA  218 (302)
  Q Consensus       141 d~~~l~~~~d~ii~~~~~l~Gl~--~~~~~~~~~~~~~~~~~~~~~~~~~~~~~~~~~~~~g~~~~~~~~~~~~~~~~~~  218 (302)
                        ...+.-+.+.+.++..++.+++.  +..............................+..+....................
  T Consensus        26 ~~~~~~~~i~~A~~~W~~~~~i~F~~~~~~~~~~~~~~~~~~~~~~~~~~~~~~~~~~~~~~~~~~~~~~~~~~~~~~~~  105 (165)
  T 1hv5_A           26 VREQVRQTVAEALQVWSEVTPLTFTEVHEGRADIMIDFARYWHGDNLPFDGPGGILAHAFFPKTHREGDVHFDYDETWTI  105 (165)
  T ss_dssp             CHHHHHHHHHHHHHHHHTTSSCEEEECSSSBCSEEEEEECSCSSSSCCCCSSSSCCEEEECCTTSSSEEEEEETTSCEES
  T ss_pred             CHHHHHHHHHHHHHHHhCcCCceEEEccCCCCCeEEEeeccccCCcccccccceeecccccCCCceeeeeeecccccccc


  Q ss_pred             CCcch------hHHhhhh
  Q Q5E705_VIBF1/1  219 TDNWM------LGHEIGH  230 (302)
  Q Consensus       219 ~~~WG------~~HEiGH  230 (302)
                        ....+      +.|||||
  T Consensus       106 ~~~~~~~~~~v~~HEiGH  123 (165)
  T 1hv5_A          106 GDNQGTDLLQVAAHEFGH  123 (165)
  T ss_dssp             SCSSSEEHHHHHHHHHHH
  T ss_pred             ccccCcchhhhhhhhhhh


  No 11 
>PF12388 Peptidase_M57:  Dual-action HEIGH metallo-peptidase
  Probab=75.62  E-value=11  Score=34.62  Aligned_cols=85  Identities=14%  Similarity=-0.095  Sum_probs=0.0

  Q ss_pred             HHHHHHHHHHHHHHHHhCCCCCCccccccccccccccchhhceeeeeccccceecCCcceeeeccccccceeccCCC---
  Q Q5E705_VIBF1/1  144 EMTKQFDLFTIGVNEFYGRDGVSGAHKMFTDSAPELEYQNMRLVDDIQISIGSAHSGYPVMSTSFPRQKSSLFKATD---  220 (302)
  Q Consensus       144 ~l~~~~d~ii~~~~~l~Gl~~~~~~~~~~~~~~~~~~~~~~~~~~~~~~~~~~~~~g~~~~~~~~~~~~~~~~~~~~---  220 (302)
                        .+....++.|..++.+ |+.... +..-......................++.+.-..--+.+...... +.....+   
  T Consensus        56 ~~~~al~~Ai~~yN~l-~l~i~F-~~tfg~~~~~~di~v~~~~~~~~~g~ggsAgFP~s~G~P~~~I~I-~~~~~~~~~~  132 (211)
  T PF12388_consen   56 AWRTALDQAINNYNAL-NLSISF-RLTFGTNYQNADIVVYSDSSNNPSGVGGSAGFPTSNGNPYKSIQI-YGSNNYSVNV  132 (211)
  T ss_pred             HHHHHHHHHHHHHHhh-CcceEE-EEEeccCccccceEEEeccccCcCCcceeccCCccCCCCCceEEE-EecCCcchHH


  Q ss_pred             -cchhHHhhhhh
  Q Q5E705_VIBF1/1  221 -NWMLGHEIGHN  231 (302)
  Q Consensus       221 -~WG~~HEiGH~  231 (302)
                         -+-+.|||||.
  T Consensus       133 ~~~vi~HEIGH~  144 (211)
  T PF12388_consen  133 NAHVITHEIGHC  144 (211)
  T ss_pred             HHHHHHHHhhhh


  No 12 
>1y93_A Macrophage metalloelastase; matrix metalloproteinase, MMP12, complex (elastase-inhibitor),
  acetohydroxamic acid, hydrolase; 1.03A {Homo sapiens} SCOP: d.92.1.11 PDB:  1rmz _A  1ycm _A*  1z3j _A*  2hu6 _A*  2oxu _A  2oxw _A
   2oxz _A  1os9 _A  1os2 _A  3f17 _A*  3ehy _A*  3ehx _A*  3f15 _A*  3f16 _A*  3f18 _A*  3f19 _A*  3f1a _A*  3lka _A*  2wo9 _A*  2wo8 _A* ...
  Probab=74.81  E-value=5.9  Score=34.08  Aligned_cols=90  Identities=11%  Similarity=-0.278  Sum_probs=0.0

  Q ss_pred             CHHHHHHHHHHHHHHHHHHhCCCCCCccccccccccccccchhhceeeeeccccceecCCcceeeeccccccceeccCCC
  Q Q5E705_VIBF1/1  141 DVLEMTKQFDLFTIGVNEFYGRDGVSGAHKMFTDSAPELEYQNMRLVDDIQISIGSAHSGYPVMSTSFPRQKSSLFKATD  220 (302)
  Q Consensus       141 d~~~l~~~~d~ii~~~~~l~Gl~~~~~~~~~~~~~~~~~~~~~~~~~~~~~~~~~~~~~g~~~~~~~~~~~~~~~~~~~~  220 (302)
                        ...+.-+...+.++...+.+++...............................+...++..+..................
  T Consensus        22 ~~~~~~~~i~~Af~~W~~~~~i~f~~~~~~~~~~~i~~~~~~~~~~~~~~~~~g~~~~~~~~~~~~~~~~~~~~~~~~~~  101 (159)
  T 1y93_A           22 NREDVDYAIRKAFQVWSNVTPLKFSKINTGMADILVVFARGAHGDDHAFDGKGGILAHAFGPGSGIGGDAHFDEDEFWTT  101 (159)
  T ss_dssp             CHHHHHHHHHHHHHHHHTTSSCEEEECSSSCCSEEEEEECSCCSSSCCCCSSSSCCEEECCSCSTTTTCEEEETTSCEES
  T ss_pred             CHHHHHHHHHHHHHHHHhhccceEEEeeccCcceEEEeeecccccccccCCCceeeeeccCCCCceeeeEeeccccccee


  Q ss_pred             cch-------hHHhhhh
  Q Q5E705_VIBF1/1  221 NWM-------LGHEIGH  230 (302)
  Q Consensus       221 ~WG-------~~HEiGH  230 (302)
                        ..+       +.|||||
  T Consensus       102 ~~~~~~~~~v~~HEiGH  118 (159)
  T 1y93_A          102 HSGGTNLFLTAVHEIGH  118 (159)
  T ss_dssp             SSSSEEHHHHHHHHHHH
  T ss_pred             ccCCcchhhhhhhhhcc


  No 13 
>d1i76a_ d.92.1.11 (A:) Neutrophil collagenase (MMP-8) {Human (Homo sapiens) [TaxId: 9606]}
  Probab=74.68  E-value=7.2  Score=33.86  Aligned_cols=90  Identities=10%  Similarity=-0.222  Sum_probs=0.0

  Q ss_pred             CHHHHHHHHHHHHHHHHHHhCCCCCCccccccccccccccchhhceeeeeccccceecCCcceeeeccccccceeccCCC
  Q Q5E705_VIBF1/1  141 DVLEMTKQFDLFTIGVNEFYGRDGVSGAHKMFTDSAPELEYQNMRLVDDIQISIGSAHSGYPVMSTSFPRQKSSLFKATD  220 (302)
  Q Consensus       141 d~~~l~~~~d~ii~~~~~l~Gl~~~~~~~~~~~~~~~~~~~~~~~~~~~~~~~~~~~~~g~~~~~~~~~~~~~~~~~~~~  220 (302)
                        ...+.-+...+.++...+.+++...............................+...+......................
  T Consensus        26 ~~~~~~~~i~~Af~~ws~v~~i~F~ev~~~~~~~~~~~~~~~~~~~~~~~~~~~~~~~~~~~~~~~~~~i~~~~~~~~~~  105 (163)
  T d1i76a_          26 SEAEVERAIKDAFELWSVASPLIFTRISQGEADINIAFYQRDHGDNSPFDGPNGILAHAFQPGQGIGGDAHFDAEETWTN  105 (163)
  T ss_dssp             CHHHHHHHHHHHHHHHHTTSSCEEEECSSSCCSEEEEEECSCCSSSCCCCSSSSCCEEECCSSSTTTTCEEEETTSCCBS
  T ss_pred             CHHHHHHHHHHHHHHHhcccCceEEEecCCCcceEEEeeecccccccccCCcccceeeccCCCCceeeEEeecccccccc


  Q ss_pred             cch-------hHHhhhh
  Q Q5E705_VIBF1/1  221 NWM-------LGHEIGH  230 (302)
  Q Consensus       221 ~WG-------~~HEiGH  230 (302)
                        .++       ++|||||
  T Consensus       106 ~~~~~~~~~v~~HEiGH  122 (163)
  T d1i76a_         106 TSANYNLFLVAAHEFGH  122 (163)
  T ss_dssp             SSSSCBHHHHHHHHHHH
  T ss_pred             ccccchheeehhhhhhh


  No 14 
>1hy7_A Stromelysin-1, MMP-3; mixed alpha beta structure, zinc protease, inhibited, hydrolase; HET: MBS; 1.50A
  {Homo sapiens} SCOP: d.92.1.11 PDB:  1biw _A*  1bm6 _A*  1bqo _A*  1b3d _A*  1cqr _A  1d5j _A*  1d7x _A*  1d8f _A*  1d8m _A*
   1g05 _A*  1g49 _A*  1c3i _A*  1sln _A*  1uea _A  2srt _A*  1ums _A*  1umt _A*  2d1o _A*  1ciz _A*  1caq _A* ...
  Probab=73.26  E-value=9.4  Score=33.47  Aligned_cols=90  Identities=9%  Similarity=-0.210  Sum_probs=0.0

  Q ss_pred             CHHHHHHHHHHHHHHHHHHhCCCCCCccccccccccccccchhhceeeeeccccceecC-------Ccceeeeccccccc
  Q Q5E705_VIBF1/1  141 DVLEMTKQFDLFTIGVNEFYGRDGVSGAHKMFTDSAPELEYQNMRLVDDIQISIGSAHS-------GYPVMSTSFPRQKS  213 (302)
  Q Consensus       141 d~~~l~~~~d~ii~~~~~l~Gl~~~~~~~~~~~~~~~~~~~~~~~~~~~~~~~~~~~~~-------g~~~~~~~~~~~~~  213 (302)
                        ...+.-+...+.++..++++++...............................+...+.       ...+....+.....
  T Consensus        27 ~~~~~~~~i~~A~~~W~~~~~i~F~e~~~~~~~~~~~~~~~~~~~~~~~~~~~~~~~~~~~~~~~~~~~~~~~~~~~~~~  106 (173)
  T 1hy7_A           27 PKDAVDSAVEKALKVWEEVTPLTFSRLYEGEADIMISFAVREHGDFYPFDGPGNVLAHAYAPGPGINGDAHFDDDEQWTK  106 (173)
  T ss_dssp             CHHHHHHHHHHHHHHHHTTSSCEEEECSSSCCSEEEEEECSSCSSSCCCCSSSSCCEEECCSSSTTTTCEEEETTSCEES
  T ss_pred             CHHHHHHHHHHHHHHHhhhcCceEEEecCCCCcEEEEEeccccCccccccccccccccccccCCCccceeeecccccccc


  Q ss_pred             eeccCCCcchhHHhhhh
  Q Q5E705_VIBF1/1  214 SLFKATDNWMLGHEIGH  230 (302)
  Q Consensus       214 ~~~~~~~~WG~~HEiGH  230 (302)
                        ......-.+-+.|||||
  T Consensus       107 ~~~~~~~~~v~~HEiGH  123 (173)
  T 1hy7_A          107 DTTGTNLFLVAAHEIGH  123 (173)
  T ss_dssp             SSSSEEHHHHHHHHHHH
  T ss_pred             cccccchhhhhhhhhcc


  No 15 
>d1l7la_ b.18.1.16 (A:) PA-IL, galactose-binding lectin 1 {Pseudomonas aeruginosa [TaxId: 287]}
  Probab=73.12  E-value=18  Score=28.88  Aligned_cols=78  Identities=9%  Similarity=-0.024  Sum_probs=0.0

  Q ss_pred             ccceEEECCCCEEEE----EecCCCCcEEEEEe--ecCCCcccccc----cccCCCCceEEEEecCCeEEEecC--CCcE
  Q Q5E705_VIBF1/1    4 QSAGVWIPAREVAYV----HGLSSDDTVMIAMA--DNLTGRVNHEM----ALNRPPRVSMSFNGVEASNGFKVP--YGGS   71 (302)
  Q Consensus         4 ~~TG~y~~~Ge~i~V----~~~~~~~~~~v~i~--~~~~~~~~~~~----~~~r~~~~~~~~~L~~g~n~i~~p--~GG~   71 (302)
                        |+|||++.+|+.|+|    .+.-...+----.+  .|....+.+.+    .+.--...+-+|++..|+-.-+.|  .-|.
  T Consensus        14 k~T~lI~~~GD~IsvvA~GW~~yG~~~~WgPqg~~~~P~~~l~~~d~~~~aLv~KIgn~~t~~i~~Gv~~~~~P~~V~G~   93 (121)
  T d1l7la_          14 QVTSIIYNPGDVITIVAAGWASYGPTQKWGPQGDREHPDQGLICHDAFCGALVMKIGNSGTIPVNTGLFRWVAPNNVQGA   93 (121)
  T ss_dssp             EEEEEEECTTCCEEEEEEEEEESSSSSCBCTBCCTTSCCSSCSCTTSCTTBEEEEETTCCCEECTTEEEEECCCTTCCEE
  T ss_pred             ceeEEEecCCCEEEEeeechhhhCCcccccCCCCCCCCcccccccchhHHHHhhhhcccceEeccCcEEEEecCCccCce


  Q ss_pred             EEEEcCCCCc
  Q Q5E705_VIBF1/1   72 VYITLGSKES   81 (302)
  Q Consensus        72 iyi~~~~~~~   81 (302)
                        |-+.+.+.+.
  T Consensus        94 l~Ll~NDvPg  103 (121)
  T d1l7la_          94 ITLIYNDVPG  103 (121)
  T ss_dssp             EEEEECCCTT
  T ss_pred             EEEEEcCCCc


  No 16 
>1mmq_A Matrilysin; metalloprotease; HET: RRS; 1.90A {Homo sapiens} SCOP: d.92.1.11 PDB:  1mmp _A*  1mmr _A*  2ddy _A*
    Probab=72.30  E-value=9.9  Score=33.19  Aligned_cols=90  Identities=10%  Similarity=-0.186  Sum_probs=0.0

  Q ss_pred             CHHHHHHHHHHHHHHHHHHhCCCCCCccccccccccccccchhhceeeeeccccceecCCcceeeeccccccceeccCCC
  Q Q5E705_VIBF1/1  141 DVLEMTKQFDLFTIGVNEFYGRDGVSGAHKMFTDSAPELEYQNMRLVDDIQISIGSAHSGYPVMSTSFPRQKSSLFKATD  220 (302)
  Q Consensus       141 d~~~l~~~~d~ii~~~~~l~Gl~~~~~~~~~~~~~~~~~~~~~~~~~~~~~~~~~~~~~g~~~~~~~~~~~~~~~~~~~~  220 (302)
                        ...+.-+...+.++...+.+++................................+..+......................
  T Consensus        27 ~~~~~~~~i~~A~~~W~~v~~l~F~ev~~~~~di~i~~~~~~~~~~~~~~~~~~~~~~~~~~~~~~~~~~~~~~~~~~~~  106 (170)
  T 1mmq_A           27 PHITVDRLVSKALNMWGKEIPLHFRKVVWGTADIMIGFARGAHGDSYPFDGPGNTLAHAFAPGTGLGGDAHFDEDERWTD  106 (170)
  T ss_dssp             CHHHHHHHHHHHHHHHHHHSSCEEEECSSSCCSEEEEEECSCCSSSCCCCSSSSCCEEECCSSSTTTTCEEEETTSCEES
  T ss_pred             CHHHHHHHHHHHHHHHhCccCceEEEeccCCCceEEEeeccCcccccccccccceeccccccccceeeeEEccccccccc


  Q ss_pred             c--------chhHHhhhh
  Q Q5E705_VIBF1/1  221 N--------WMLGHEIGH  230 (302)
  Q Consensus       221 ~--------WG~~HEiGH  230 (302)
                        .        .-++|||||
  T Consensus       107 ~~~~~~~~~~v~~HEiGH  124 (170)
  T 1mmq_A          107 GSSLGINFLYAATHELGH  124 (170)
  T ss_dssp             SSSSSEEHHHHHHHHHHH
  T ss_pred             ccccccchhheeehhccc


  No 17 
>SUPFAM template d.92.1 Metalloproteases ("zincins"), catalytic domain (55486) SCOP seed sequence: d1fbl_2.
  Probab=71.43  E-value=4.4  Score=32.12  Aligned_cols=9  Identities=44%  Similarity=0.829  Sum_probs=0.0

  Q ss_pred             hHHhhhhhc
  Q Q5E705_VIBF1/1  224 LGHEIGHNQ  232 (302)
  Q Consensus       224 ~~HEiGH~~  232 (302)
                        ..|||||..
  T Consensus       117 ~~HE~GH~L  125 (172)
  T SUPFAM0037477   117 AAHELGHAL  125 (172)
  T ss_pred             hhhhhhhhh


  No 18 
>d1hfca_ d.92.1.11 (A:) Fibroblast collagenase (MMP-1) {Human (Homo sapiens) [TaxId: 9606]}
  Probab=70.95  E-value=16  Score=31.37  Aligned_cols=90  Identities=9%  Similarity=-0.121  Sum_probs=0.0

  Q ss_pred             CHHHHHHHHHHHHHHHHHHhCCCCCCccccccccccccccchhhceeeeeccccceecC-------Ccceeeeccccccc
  Q Q5E705_VIBF1/1  141 DVLEMTKQFDLFTIGVNEFYGRDGVSGAHKMFTDSAPELEYQNMRLVDDIQISIGSAHS-------GYPVMSTSFPRQKS  213 (302)
  Q Consensus       141 d~~~l~~~~d~ii~~~~~l~Gl~~~~~~~~~~~~~~~~~~~~~~~~~~~~~~~~~~~~~-------g~~~~~~~~~~~~~  213 (302)
                        ...+.-+.+.+.++...+.+++...............................+...+.       +.-+....+.....
  T Consensus        20 ~~~~~~~~i~~A~~~W~~~~~i~F~~v~~~~~~~~~~~~~~~~~~~~~~~~~~~~~~~~~~~~~~~~~~i~~~~~~~~~~   99 (157)
  T d1hfca_          20 PRADVDHAIEKAFQLWSNVTPLTFTKVSEGQADIMISFVRGDHRDNSPFDGPGGNLAHAFQPGPGIGGDAHFDEDERWTN   99 (157)
  T ss_dssp             CHHHHHHHHHHHHHHHHTTSSCEEEECSSSCCSEEEEEECSSCSSSCCCCSSSSCCEEECCSSSTTTTCEEEETTSCCBS
  T ss_pred             CHHHHHHHHHHHHHHHHhhcCccEEEeccCCcccceeeeccccccccccCCCcceeEEeecCCCCeeeeEEecccccccc


  Q ss_pred             eeccCCCcchhHHhhhh
  Q Q5E705_VIBF1/1  214 SLFKATDNWMLGHEIGH  230 (302)
  Q Consensus       214 ~~~~~~~~WG~~HEiGH  230 (302)
                        .......-.-+.|||||
  T Consensus       100 ~~~~~~~~~v~~HEiGH  116 (157)
  T d1hfca_         100 NFREYNLHRVAAHELGH  116 (157)
  T ss_dssp             SSSSCBHHHHHHHHHHH
  T ss_pred             ccccchhhhhHhhhhhh


  No 19 
>SUPFAM template d.92.1 Metalloproteases ("zincins"), catalytic domain (55486) SCOP seed sequence: d1q3aa_.
  Probab=70.95  E-value=4.2  Score=31.55  Aligned_cols=8  Identities=50%  Similarity=0.999  Sum_probs=0.0

  Q ss_pred             hHHhhhhh
  Q Q5E705_VIBF1/1  224 LGHEIGHN  231 (302)
  Q Consensus       224 ~~HEiGH~  231 (302)
                        ..|||||.
  T Consensus       111 ~~HE~GH~  118 (159)
  T SUPFAM0042596   111 AAHELGHA  118 (159)
  T ss_pred             hhhhhhhh


  No 20 
>SUPFAM template d.92.1 Metalloproteases ("zincins"), catalytic domain (55486) SCOP seed sequence: d1hfs__.
  Probab=70.22  E-value=4.6  Score=31.36  Aligned_cols=8  Identities=50%  Similarity=0.999  Sum_probs=0.0

  Q ss_pred             hHHhhhhh
  Q Q5E705_VIBF1/1  224 LGHEIGHN  231 (302)
  Q Consensus       224 ~~HEiGH~  231 (302)
                        ..||+||.
  T Consensus       112 ~~HE~GH~  119 (160)
  T SUPFAM0038581   112 AAHELGHA  119 (160)
  T ss_pred             hhhhhhhh


  No 21 
>SUPFAM template d.92.1 Metalloproteases ("zincins"), catalytic domain (55486) SCOP seed sequence: d1cxva_.
  Probab=69.47  E-value=4.9  Score=31.51  Aligned_cols=9  Identities=44%  Similarity=0.829  Sum_probs=0.0

  Q ss_pred             hHHhhhhhc
  Q Q5E705_VIBF1/1  224 LGHEIGHNQ  232 (302)
  Q Consensus       224 ~~HEiGH~~  232 (302)
                        ..||+||..
  T Consensus       117 ~~HE~GH~L  125 (164)
  T SUPFAM0036188   117 AAHELGHAL  125 (164)
  T ss_pred             hhhhhhhhc


  No 22 
>PIRSF005785 Zn-prot_arch 
  Probab=68.91  E-value=4.5  Score=36.84  Aligned_cols=10  Identities=40%  Similarity=0.521  Sum_probs=0.0

  Q ss_pred             hhHHhhhhhc
  Q Q5E705_VIBF1/1  223 MLGHEIGHNQ  232 (302)
  Q Consensus       223 G~~HEiGH~~  232 (302)
                        +..||+||.+
  T Consensus       149 Ev~HElGH~f  158 (204)
  T PIRSF005785     149 EVLHELGHVF  158 (204)
  T ss_pred             hhhcchhhhc


  No 23 
>d1mmqa_ d.92.1.11 (A:) Matrilysin (MMP-7) {Human (Homo sapiens) [TaxId: 9606]}
  Probab=68.51  E-value=13  Score=32.42  Aligned_cols=90  Identities=10%  Similarity=-0.216  Sum_probs=0.0

  Q ss_pred             CHHHHHHHHHHHHHHHHHHhCCCCCCccccccccccccccchhhceeeeeccccceecCCcceeeeccccccceeccCCC
  Q Q5E705_VIBF1/1  141 DVLEMTKQFDLFTIGVNEFYGRDGVSGAHKMFTDSAPELEYQNMRLVDDIQISIGSAHSGYPVMSTSFPRQKSSLFKATD  220 (302)
  Q Consensus       141 d~~~l~~~~d~ii~~~~~l~Gl~~~~~~~~~~~~~~~~~~~~~~~~~~~~~~~~~~~~~g~~~~~~~~~~~~~~~~~~~~  220 (302)
                        ...+.-+...+.++...+.+++...............................+..++..+.-.................
  T Consensus        27 ~~~~~~~~i~~A~~~W~~v~~i~F~~v~~~~~di~i~~~~~~~~~~~~~~~~~~~~~~~~~~~~~~~~~i~~~~~~~~~~  106 (166)
  T d1mmqa_          27 PHITVDRLVSKALNMWGKEIPLHFRKVVWGTADIMIGFARGAHGDSYPFDGPGNTLAHAFAPGTGLGGDAHFDEDERWTD  106 (166)
  T ss_dssp             CHHHHHHHHHHHHHHHHHHSSCEEEECSSSCCSEEEEEECSCCSSSCCCCSSSSCCEEECCSSSTTTTCEEEETTSCEES
  T ss_pred             CHHHHHHHHHHHHHHHhCcCCeeEEecccCCcceEEEEeccccCCcccccccceeeeeccCCCccccceEEecchhhhhc


  Q ss_pred             cch--------hHHhhhh
  Q Q5E705_VIBF1/1  221 NWM--------LGHEIGH  230 (302)
  Q Consensus       221 ~WG--------~~HEiGH  230 (302)
                        .+.        +.|||||
  T Consensus       107 ~~~~~~~~~~v~~HEiGH  124 (166)
  T d1mmqa_         107 GSSLGINFLYAATHELGH  124 (166)
  T ss_dssp             SSSSSEEHHHHHHHHHHH
  T ss_pred             cccccchhhhhhhhhhcc


  No 24 
>SUPFAM template d.92.1 Metalloproteases ("zincins"), catalytic domain (55486) SCOP seed sequence: d1cgla_.
  Probab=68.50  E-value=5.3  Score=31.53  Aligned_cols=9  Identities=44%  Similarity=0.829  Sum_probs=0.0

  Q ss_pred             hHHhhhhhc
  Q Q5E705_VIBF1/1  224 LGHEIGHNQ  232 (302)
  Q Consensus       224 ~~HEiGH~~  232 (302)
                        ..||+||..
  T Consensus       116 ~~HE~GH~L  124 (168)
  T SUPFAM0035975   116 AAHELGHAL  124 (168)
  T ss_pred             hhhhhhhhc


  No 25 
>d1kapp2 d.92.1.6 (P:1-246) Metalloprotease {Pseudomonas aeruginosa [TaxId: 287]}
  Probab=68.42  E-value=19  Score=33.97  Aligned_cols=88  Identities=14%  Similarity=-0.068  Sum_probs=0.0

  Q ss_pred             HHHHHHHHHHHHHHHHHhCCCCCCccccccccccccccchhhceeeeeccccceecCCcceeeeccccccceeccCCCcc
  Q Q5E705_VIBF1/1  143 LEMTKQFDLFTIGVNEFYGRDGVSGAHKMFTDSAPELEYQNMRLVDDIQISIGSAHSGYPVMSTSFPRQKSSLFKATDNW  222 (302)
  Q Consensus       143 ~~l~~~~d~ii~~~~~l~Gl~~~~~~~~~~~~~~~~~~~~~~~~~~~~~~~~~~~~~g~~~~~~~~~~~~~~~~~~~~~W  222 (302)
                        .+-.+...+.++...+++++.........................+...........+..+....+...........++.
  T Consensus        90 ~~q~~~vr~A~~~ws~va~i~F~ev~~~~~adI~~~~~~~~~~~~~~a~~~~~~~~~~~~~~~~~~~~~~~~~~~~~g~~  169 (246)
  T d1kapp2          90 AQQQAQAKLSLQSWSDVTNIHFVDAGQGDQGDLTFGNFSSSVGGAAFAFLPDVPDALKGQSWYLINSSYSANVNPANGNY  169 (246)
  T ss_dssp             HHHHHHHHHHHHHHHTTBSEEEEEEEESSCSSEEEEEECSCCSSSEEECCTTSCTTTTTEEEEECSSSCCTTTSCCTTSH
  T ss_pred             HHHHHHHHHHHHHHHHhhCcceeeccCCCceEEEEEEeCCCCCCeeEeccCCCCcccccccccccccccccccCcccchh


  Q ss_pred             h---hHHhhhh
  Q Q5E705_VIBF1/1  223 M---LGHEIGH  230 (302)
  Q Consensus       223 G---~~HEiGH  230 (302)
                        +   ++|||||
  T Consensus       170 ~~~t~lHEIGH  180 (246)
  T d1kapp2         170 GRQTLTHEIGH  180 (246)
  T ss_dssp             HHHHHHHHHHH
  T ss_pred             HHHHHHHHHHH


  No 26 
>SUPFAM template d.92.1 Metalloproteases ("zincins"), catalytic domain (55486) SCOP seed sequence: d1eaka2.
  Probab=68.11  E-value=5.3  Score=31.41  Aligned_cols=9  Identities=44%  Similarity=0.829  Sum_probs=0.0

  Q ss_pred             hHHhhhhhc
  Q Q5E705_VIBF1/1  224 LGHEIGHNQ  232 (302)
  Q Consensus       224 ~~HEiGH~~  232 (302)
                        ..|||||..
  T Consensus       118 ~~HE~GH~L  126 (169)
  T SUPFAM0036877   118 AAHELGHAL  126 (169)
  T ss_pred             hhhhhhhhh


  No 27 
>1cge_A Fibroblast collagenase; hydrolase (metalloprotease); 1.90A {Homo sapiens} SCOP: d.92.1.11 PDB:  2j0t _A
   1ayk _A  1hfc _A*  2ayk _A  2tcl _A*  3ayk _A*  4ayk _A*  1cgl _A*  1cgf _A  966c _A*
  Probab=67.83  E-value=19  Score=31.44  Aligned_cols=90  Identities=8%  Similarity=-0.156  Sum_probs=0.0

  Q ss_pred             CHHHHHHHHHHHHHHHHHHhCCCCCCccccccccccccccchhhceeeeeccccceecCCc-------ceeeeccccccc
  Q Q5E705_VIBF1/1  141 DVLEMTKQFDLFTIGVNEFYGRDGVSGAHKMFTDSAPELEYQNMRLVDDIQISIGSAHSGY-------PVMSTSFPRQKS  213 (302)
  Q Consensus       141 d~~~l~~~~d~ii~~~~~l~Gl~~~~~~~~~~~~~~~~~~~~~~~~~~~~~~~~~~~~~g~-------~~~~~~~~~~~~  213 (302)
                        +..+..+...+.++...+.+++...............................+..++..+       -+....+.....
  T Consensus        25 ~~~~~~~ai~~A~~~W~~~~~i~F~~v~~~~~di~~~~~~~~~~~~~~~~~~~~~~~~~~~~~~~~~~~~~~~~~~~~~~  104 (168)
  T 1cge_A           25 PRADVDHAIEKAFQLWSNVTPLTFTKVSEGQADIMISFVRGDHRDNSPFDGPGGNLAHAFQPGPGIGGDAHFDEDERWTN  104 (168)
  T ss_dssp             CHHHHHHHHHHHHHHHHTTSSCEEEECSSSCCSEEEEEECSCCSSSSCCCSSSSCCEEECCSSSTTTTCEEEETTSCCBS
  T ss_pred             CHHHHHHHHHHHHHHHHhhcCeeEEEccCCCCceEEEeecccccccccccccccceeeeeCCCCceeeeEEeeccccccc


  Q ss_pred             eeccCCCcchhHHhhhh
  Q Q5E705_VIBF1/1  214 SLFKATDNWMLGHEIGH  230 (302)
  Q Consensus       214 ~~~~~~~~WG~~HEiGH  230 (302)
                        .........-+.|||||
  T Consensus       105 ~~~~~~~~~v~~HEiGH  121 (168)
  T 1cge_A          105 NFREYNLHRVAAHELGH  121 (168)
  T ss_dssp             SSSSCBHHHHHHHHHHH
  T ss_pred             ccchhHHHHHHHHHhhh


  No 28 
>SUPFAM template d.92.1 Metalloproteases ("zincins"), catalytic domain (55486) SCOP seed sequence: d1mmq__.
  Probab=67.51  E-value=5.6  Score=31.27  Aligned_cols=9  Identities=44%  Similarity=0.829  Sum_probs=0.0

  Q ss_pred             hHHhhhhhc
  Q Q5E705_VIBF1/1  224 LGHEIGHNQ  232 (302)
  Q Consensus       224 ~~HEiGH~~  232 (302)
                        ..||+||..
  T Consensus       118 ~~HE~GH~L  126 (166)
  T SUPFAM0041006   118 AAHELGHAL  126 (166)
  T ss_pred             hhhhhhhhc


  No 29 
>pfam04228 Zn_peptidase Putative neutral zinc metallopeptidase. Members of this family have a predicted zinc
  binding motif characteristic of neutral zinc metallopeptidases (Prosite:PDOC00129).
  Probab=67.23  E-value=5.6  Score=37.94  Aligned_cols=12  Identities=33%  Similarity=0.938  Sum_probs=0.0

  Q ss_pred             chhHHhhhhhcc
  Q Q5E705_VIBF1/1  222 WMLGHEIGHNQA  233 (302)
  Q Consensus       222 WG~~HEiGH~~Q  233 (302)
                        +.+.||+|||-|
  T Consensus       172 YViAHEvGHHVQ  183 (292)
  T pfam04228       172 YVIAHEVGHHVQ  183 (292)
  T ss_pred             HHHHhhhhhHHH


  No 30 
>SUPFAM template d.92.1 Metalloproteases ("zincins"), catalytic domain (55486) SCOP seed sequence: d1bzsa_.
  Probab=66.81  E-value=4.9  Score=31.35  Aligned_cols=9  Identities=44%  Similarity=0.829  Sum_probs=0.0

  Q ss_pred             hHHhhhhhc
  Q Q5E705_VIBF1/1  224 LGHEIGHNQ  232 (302)
  Q Consensus       224 ~~HEiGH~~  232 (302)
                        ..||+||..
  T Consensus       117 ~~HE~GH~L  125 (165)
  T SUPFAM0035790   117 AAHELGHAL  125 (165)
  T ss_pred             eehhhhhhc


  No 31 
>1bkcA00 3.40.390.10 
  Probab=65.58  E-value=6.6  Score=33.49  Aligned_cols=9  Identities=56%  Similarity=1.091  Sum_probs=0.0

  Q ss_pred             hHHhhhhhc
  Q Q5E705_VIBF1/1  224 LGHEIGHNQ  232 (302)
  Q Consensus       224 ~~HEiGH~~  232 (302)
                        +.||+||++
  T Consensus       185 ~AHE~GH~l  193 (255)
  T 1bkcA00         185 VAHELGHNL  193 (255)
  T ss_pred             hhhhhhhhc


  No 32 
>SUPFAM template d.92.1 Metalloproteases ("zincins"), catalytic domain (55486) SCOP seed sequence: d1rm8a_.
  Probab=65.54  E-value=6.3  Score=31.13  Aligned_cols=9  Identities=44%  Similarity=0.829  Sum_probs=0.0

  Q ss_pred             hHHhhhhhc
  Q Q5E705_VIBF1/1  224 LGHEIGHNQ  232 (302)
  Q Consensus       224 ~~HEiGH~~  232 (302)
                        ..||+||..
  T Consensus       121 ~~HE~GH~L  129 (169)
  T SUPFAM0043304   121 AAHELGHAL  129 (169)
  T ss_pred             hhhhhhhhh


  No 33 
>SUPFAM template d.92.1 Metalloproteases ("zincins"), catalytic domain (55486) SCOP seed sequence: d830ca_.
  Probab=64.42  E-value=5.4  Score=31.45  Aligned_cols=9  Identities=44%  Similarity=0.829  Sum_probs=0.0

  Q ss_pred             hHHhhhhhc
  Q Q5E705_VIBF1/1  224 LGHEIGHNQ  232 (302)
  Q Consensus       224 ~~HEiGH~~  232 (302)
                        ..||+||..
  T Consensus       117 ~~HE~GH~L  125 (168)
  T SUPFAM0045657   117 AAHELGHAL  125 (168)
  T ss_pred             eeehhhhhc


  No 34 
>SUPFAM template d.92.1 Metalloproteases ("zincins"), catalytic domain (55486) SCOP seed sequence: d1bqqm_.
  Probab=64.18  E-value=6.9  Score=31.07  Aligned_cols=9  Identities=44%  Similarity=0.829  Sum_probs=0.0

  Q ss_pred             hHHhhhhhc
  Q Q5E705_VIBF1/1  224 LGHEIGHNQ  232 (302)
  Q Consensus       224 ~~HEiGH~~  232 (302)
                        ..||+||..
  T Consensus       124 ~~HElGH~L  132 (174)
  T SUPFAM0035666   124 AAHELGHAL  132 (174)
  T ss_pred             hhhhhhhhh


  No 35 
>SUPFAM template d.92.1 Metalloproteases ("zincins"), catalytic domain (55486) SCOP seed sequence: d1jk3a_.
  Probab=64.14  E-value=5.9  Score=30.64  Aligned_cols=9  Identities=44%  Similarity=0.829  Sum_probs=0.0

  Q ss_pred             hHHhhhhhc
  Q Q5E705_VIBF1/1  224 LGHEIGHNQ  232 (302)
  Q Consensus       224 ~~HEiGH~~  232 (302)
                        ..|||||..
  T Consensus       111 ~~HE~GH~L  119 (158)
  T SUPFAM0039592   111 AAHELGHAL  119 (158)
  T ss_pred             eehhhhhhc


  No 36 
 >3KEC_Entity_1
  Probab=63.67  E-value=18  Score=31.44  Aligned_cols=90  Identities=14%  Similarity=-0.151  Sum_probs=0.0

  Q ss_pred             CHHHHHHHHHHHHHHHHHHhCCCCCCccccccccccccccchhhceeeeeccccceecC-------Ccceeeeccccccc
  Q Q5E705_VIBF1/1  141 DVLEMTKQFDLFTIGVNEFYGRDGVSGAHKMFTDSAPELEYQNMRLVDDIQISIGSAHS-------GYPVMSTSFPRQKS  213 (302)
  Q Consensus       141 d~~~l~~~~d~ii~~~~~l~Gl~~~~~~~~~~~~~~~~~~~~~~~~~~~~~~~~~~~~~-------g~~~~~~~~~~~~~  213 (302)
                        +..+.-+...+.++...+.+++...............................+...+.       +..+....+.....
  T Consensus        27 ~~~~~~~~i~~Af~~W~~~~~~~f~~v~~~~~~~~~~~~~~~~~~~~~~~~~~~~~~~~~~~~~~~~~~i~~~~~~~~~~  106 (167)
  T 3KEC_Entity_1    27 THSEVEKAFKKAFKVWSDVTPLNFTRLHDGIADIMISFGIKEHGDFYPFDGPSGLLAHAFPPGPNYGGDAHFDDDETWTS  106 (167)
  T ss_pred             CHHHHHHHHHHHHHHHHhhcCeeEEEecCCCCceEEEEeeccccccccccCcccccccccCCCCcccceEeccccccccc


  Q ss_pred             eeccCCCcchhHHhhhh
  Q Q5E705_VIBF1/1  214 SLFKATDNWMLGHEIGH  230 (302)
  Q Consensus       214 ~~~~~~~~WG~~HEiGH  230 (302)
                        .......-+-+.|||||
  T Consensus       107 ~~~~~~~~~v~~HEiGH  123 (167)
  T 3KEC_Entity_1   107 SSKGYNLFLVAAHEFGH  123 (167)
  T ss_pred             cCCCceeEEEeeeeccc


  No 37 
>d1hv5a_ d.92.1.11 (A:) Stromelysin-3 (MMP-11) {Mouse (Mus musculus) [TaxId: 10090]}
  Probab=63.45  E-value=34  Score=29.96  Aligned_cols=88  Identities=13%  Similarity=-0.053  Sum_probs=0.0

  Q ss_pred             CHHHHHHHHHHHHHHHHHHhCCCCCCccccccccccccccchhhceeeeeccccceecCCcceeeeccccccceeccCCC
  Q Q5E705_VIBF1/1  141 DVLEMTKQFDLFTIGVNEFYGRDGVSGAHKMFTDSAPELEYQNMRLVDDIQISIGSAHSGYPVMSTSFPRQKSSLFKATD  220 (302)
  Q Consensus       141 d~~~l~~~~d~ii~~~~~l~Gl~~~~~~~~~~~~~~~~~~~~~~~~~~~~~~~~~~~~~g~~~~~~~~~~~~~~~~~~~~  220 (302)
                        ...+.-+...+.++...+.+++...............+..............++..+++    ..+..........+...
  T Consensus        26 ~~~~~~~~i~~A~~~W~~v~~i~F~ev~~~~~di~i~~~~~~~~~~~~~~~~~~~~~~~----~~~~~~~~~~i~~~~~~  101 (162)
  T d1hv5a_          26 VREQVRQTVAEALQVWSEVTPLTFTEVHEGRADIMIDFARYWHGDNLPFDGPGGILAHA----FFPKTHREGDVHFDYDE  101 (162)
  T ss_dssp             CHHHHHHHHHHHHHHHHTTSSCEEEECSSSBCSEEEEEECSCSSSSCCCCSSSSCCEEE----ECCTTSSSEEEEEETTS
  T ss_pred             CHHHHHHHHHHHHHHHhccCCceEEEeecCCCceEEEEeccccCCcccccCCCceeeee----ecCCCccccceeecchh


  Q ss_pred             cc------------hhHHhhhhhc
  Q Q5E705_VIBF1/1  221 NW------------MLGHEIGHNQ  232 (302)
  Q Consensus       221 ~W------------G~~HEiGH~~  232 (302)
                        .|            -+.|||||..
  T Consensus       102 ~~~~~~~~g~~~~~v~~HEiGHaL  125 (162)
  T d1hv5a_         102 TWTIGDNQGTDLLQVAAHEFGHVL  125 (162)
  T ss_dssp             CEESSCSSSEEHHHHHHHHHHHHT
  T ss_pred             ccccccccCcchhhhhhhhhhhhc


  No 38 
>SUPFAM template d.92.1 Metalloproteases ("zincins"), catalytic domain (55486) SCOP seed sequence: d1kapp2.
  Probab=63.10  E-value=5.6  Score=33.98  Aligned_cols=9  Identities=44%  Similarity=0.977  Sum_probs=0.0

  Q ss_pred             hHHhhhhhc
  Q Q5E705_VIBF1/1  224 LGHEIGHNQ  232 (302)
  Q Consensus       224 ~~HEiGH~~  232 (302)
                        +.|||||+.
  T Consensus       174 ~~HE~GH~L  182 (246)
  T SUPFAM0040001   174 IAHELGHAL  182 (246)
  T ss_pred             eeeehhhhh


  No 39 
>830c_A MMP-13, MMP-13; matrix metalloprotease; HET: RS1; 1.60A {Homo sapiens} SCOP: d.92.1.11 PDB:  456c _A*
   1you _A*  1eub _A*  1xuc _A*  1xud _A*  1xur _A*  3elm _A*  3i7g _A*  3i7i _A*  2ow9 _A*  2ozr _A*  2d1n _A*  1fls _A*  1fm1 _A*  1ztq _A*
   2e2d _A  2pjt _A*  1cxv _A*
  Probab=62.92  E-value=20  Score=31.32  Aligned_cols=92  Identities=14%  Similarity=-0.150  Sum_probs=0.0

  Q ss_pred             CHHHHHHHHHHHHHHHHHHhCCCCCCccccccccccccccchhhceeeeeccccceecC-------Ccceeeeccccccc
  Q Q5E705_VIBF1/1  141 DVLEMTKQFDLFTIGVNEFYGRDGVSGAHKMFTDSAPELEYQNMRLVDDIQISIGSAHS-------GYPVMSTSFPRQKS  213 (302)
  Q Consensus       141 d~~~l~~~~d~ii~~~~~l~Gl~~~~~~~~~~~~~~~~~~~~~~~~~~~~~~~~~~~~~-------g~~~~~~~~~~~~~  213 (302)
                        +..+.-+...+.++..++..++......................-........+...+.       +..+....+.....
  T Consensus        27 ~~~~~~~~i~~Af~~W~~~~~~~f~~v~~~~~~~~~~~~~~~~~~~~~~~~~~~~~~~~~~~~~~~~~~i~~~~~~~~~~  106 (168)
  T 830c_A           27 THSEVEKAFKKAFKVWSDVTPLNFTRLHDGIADIMISFGIKEHGDFYPFDGPSGLLAHAFPPGPNYGGDAHFDDDETWTS  106 (168)
  T ss_dssp             CHHHHHHHHHHHHHHHHTTSSCEEEEESSSCCSEEEEEECSSCSSSCCCCSSSSCCEEECCSSSTTTTCEEEETTSCEES
  T ss_pred             CHHHHHHHHHHHHHHHHcccCceEEEeccCCCceEEEeeeccccccccccCcccceeeccCCCCcccceEeccccccccc


  Q ss_pred             eeccCCCcchhHHhhhhhc
  Q Q5E705_VIBF1/1  214 SLFKATDNWMLGHEIGHNQ  232 (302)
  Q Consensus       214 ~~~~~~~~WG~~HEiGH~~  232 (302)
                        .......-.-+.|||||..
  T Consensus       107 ~~~~~~~~~v~~HEiGHaL  125 (168)
  T 830c_A          107 SSKGYNLFLVAAHEFGHSL  125 (168)
  T ss_dssp             SSSSEEHHHHHHHHHHHHT
  T ss_pred             cCCCceeEEEeeeeccccc


  No 40 
>SUPFAM template d.92.1 Metalloproteases ("zincins"), catalytic domain (55486) SCOP seed sequence: d1gkda_.
  Probab=62.90  E-value=5.9  Score=30.72  Aligned_cols=8  Identities=50%  Similarity=0.999  Sum_probs=0.0

  Q ss_pred             hHHhhhhh
  Q Q5E705_VIBF1/1  224 LGHEIGHN  231 (302)
  Q Consensus       224 ~~HEiGH~  231 (302)
                        ..|||||.
  T Consensus       115 ~~HE~GH~  122 (159)
  T SUPFAM0038130   115 AAHELGHA  122 (159)
  T ss_pred             eeehhhhh


  No 41 
>d1hy7a_ d.92.1.11 (A:) Stromelysin-1 (MMP-3) {Human (Homo sapiens), fibroblast [TaxId: 9606]}
  Probab=62.78  E-value=21  Score=31.09  Aligned_cols=90  Identities=9%  Similarity=-0.210  Sum_probs=0.0

  Q ss_pred             CHHHHHHHHHHHHHHHHHHhCCCCCCccccccccccccccchhhceeeeeccccceecC-------Ccceeeeccccccc
  Q Q5E705_VIBF1/1  141 DVLEMTKQFDLFTIGVNEFYGRDGVSGAHKMFTDSAPELEYQNMRLVDDIQISIGSAHS-------GYPVMSTSFPRQKS  213 (302)
  Q Consensus       141 d~~~l~~~~d~ii~~~~~l~Gl~~~~~~~~~~~~~~~~~~~~~~~~~~~~~~~~~~~~~-------g~~~~~~~~~~~~~  213 (302)
                        ...+.-+...+.++...+.+++...............................+...+.       ...+..........
  T Consensus        27 ~~~~~~~~i~~A~~~W~~~~~i~F~e~~~~~~d~~~~~~~~~~~~~~~~~~~~~~~~~~~~~~~~~~~~~~~~~~~~~~~  106 (168)
  T d1hy7a_          27 PKDAVDSAVEKALKVWEEVTPLTFSRLYEGEADIMISFAVREHGDFYPFDGPGNVLAHAYAPGPGINGDAHFDDDEQWTK  106 (168)
  T ss_dssp             CHHHHHHHHHHHHHHHHTTSSCEEEECSSSCCSEEEEEECSSCSSSCCCCSSSSCCEEECCSSSTTTTCEEEETTSCEES
  T ss_pred             CHHHHHHHHHHHHHHHHhhcCceeEeccCCcCceEEEeeccccccccccccccccccccccCCCCeeeeEeecccccccc


  Q ss_pred             eeccCCCcchhHHhhhh
  Q Q5E705_VIBF1/1  214 SLFKATDNWMLGHEIGH  230 (302)
  Q Consensus       214 ~~~~~~~~WG~~HEiGH  230 (302)
                        .......-+-+.|||||
  T Consensus       107 ~~~~~~~~~v~~HEiGH  123 (168)
  T d1hy7a_         107 DTTGTNLFLVAAHEIGH  123 (168)
  T ss_dssp             SSSSEEHHHHHHHHHHH
  T ss_pred             ccccccceeeeHhhhcc


  No 42 
>SUPFAM template d.92.1 Metalloproteases ("zincins"), catalytic domain (55486) SCOP seed sequence: d1bkca_.
  Probab=62.53  E-value=8.6  Score=33.13  Aligned_cols=9  Identities=56%  Similarity=0.981  Sum_probs=0.0

  Q ss_pred             hHHhhhhhc
  Q Q5E705_VIBF1/1  224 LGHEIGHNQ  232 (302)
  Q Consensus       224 ~~HEiGH~~  232 (302)
                        +.||+||+.
  T Consensus       185 ~aHE~GH~l  193 (255)
  T SUPFAM0035598   185 AAHELGHNL  193 (255)
  T ss_pred             HhhhhHHHh


  No 43 
>SUPFAM template d.92.1 Metalloproteases ("zincins"), catalytic domain (55486) SCOP seed sequence: d1hv5a_.
  Probab=62.17  E-value=6.4  Score=30.78  Aligned_cols=9  Identities=44%  Similarity=0.829  Sum_probs=0.0

  Q ss_pred             hHHhhhhhc
  Q Q5E705_VIBF1/1  224 LGHEIGHNQ  232 (302)
  Q Consensus       224 ~~HEiGH~~  232 (302)
                        ..||+||..
  T Consensus       117 ~~HE~GH~L  125 (162)
  T SUPFAM0038739   117 AAHELGHAL  125 (162)
  T ss_pred             eeehhhhhh


  No 44 
>SUPFAM template d.92.1 Metalloproteases ("zincins"), catalytic domain (55486) SCOP seed sequence: d1kuh__.
  Probab=61.82  E-value=4.9  Score=30.02  Aligned_cols=9  Identities=44%  Similarity=0.829  Sum_probs=0.0

  Q ss_pred             hHHhhhhhc
  Q Q5E705_VIBF1/1  224 LGHEIGHNQ  232 (302)
  Q Consensus       224 ~~HEiGH~~  232 (302)
                        ..||+||..
  T Consensus        81 ~~HE~GH~L   89 (132)
  T SUPFAM0040268    81 AAHELGHAL   89 (132)
  T ss_pred             eeeeccccc


  No 45 
>pfam12388 Peptidase_M57 Dual-action HEIGH metallo-peptidase. The catalytic triad for this family of proteases is
  HE-H-H, which in many members is in the sequence motif HEIGH.
  Probab=61.74  E-value=28  Score=31.97  Aligned_cols=102  Identities=15%  Similarity=0.133  Sum_probs=0.0

  Q ss_pred             HHHHHHHHHHHHHHHHhCCCCCCccc--cccccccccccchhhceeeeecccccee-cCCcceeeeccccccceeccCCC
  Q Q5E705_VIBF1/1  144 EMTKQFDLFTIGVNEFYGRDGVSGAH--KMFTDSAPELEYQNMRLVDDIQISIGSA-HSGYPVMSTSFPRQKSSLFKATD  220 (302)
  Q Consensus       144 ~l~~~~d~ii~~~~~l~Gl~~~~~~~--~~~~~~~~~~~~~~~~~~~~~~~~~~~~-~~g~~~~~~~~~~~~~~~~~~~~  220 (302)
                        .+....++.|..||.| |+.......  .+...-..........--...-+.+|+. ..|.|-....-.....+......
  T Consensus        56 ~~~~al~~AI~~yN~l-~l~l~F~~t~gtn~~~~di~v~~~~~~~~~g~ggsAGFP~s~G~P~~~V~i~~~~~~~~~~~~  134 (211)
  T pfam12388        56 KGQTALDDAVNNYNNL-GLDISFRLTFGTNYQNADMVVYDNSVNNPSGSGGSAGFPDSNGDPAKFVQIYDLENGSTNVNE  134 (211)
  T ss_pred             HHHHHHHHHHHHHhhc-CceEEEEEEeccCCCCCcEEEEeccccCCCCceeeccCCCCCCCCCceEEEeccCCCCcchhh


  Q ss_pred             cchhHHhhhhhcccccccc---CCCccchh
  Q Q5E705_VIBF1/1  221 NWMLGHEIGHNQAANWLNV---VGAGETAN  247 (302)
  Q Consensus       221 ~WG~~HEiGH~~Q~~~~~~---~g~~Evtn  247 (302)
                        . -+.|||||..=.+.-.|   .++|...|
  T Consensus       135 ~-vi~HEiGHciGfRHTD~fnRsSCG~~~n  163 (211)
  T pfam12388       135 H-VITHEIGHSIGFRHTDYFDRSSCGQGGN  163 (211)
  T ss_pred             h-hhhhhhhceeccccccccCccccccCCC


  No 46 
>PF04228 Zn_peptidase:  Putative neutral zinc metallopeptidase;  InterPro:   Members of this family of
  bacterial proteins are described as hypothetical proteins or zinc metallopeptidases. The majority have a HExxH
  zinc-binding motif characteristic of neutral zinc metallopeptidases, however there is no evidence to support
  their function as metallopeptidases.
  Probab=61.50  E-value=8.6  Score=37.28  Aligned_cols=10  Identities=50%  Similarity=0.850  Sum_probs=0.0

  Q ss_pred             hHHhhhhhcc
  Q Q5E705_VIBF1/1  224 LGHEIGHNQA  233 (302)
  Q Consensus       224 ~~HEiGH~~Q  233 (302)
                        +.||+||+-|
  T Consensus       174 lAHEyGHHVQ  183 (292)
  T PF04228_consen  174 LAHEYGHHVQ  183 (292)
  T ss_pred             HHHHHHHHHH


  No 47 
>2ovx_A Matrix metalloproteinase-9 (EC 3.4.24.35) (MMP- 9) (92 kDa type IV collagenase) (92...; hydrolase,
  S1-prime pocket; HET: 4MR; 2.00A {Homo sapiens} SCOP: d.92.1.11 PDB:  2ovz _A*  2ow0 _A*  2ow1 _A*  2ow2 _A*  1gkd _A*  1gkc _A*
    Probab=61.17  E-value=17  Score=31.24  Aligned_cols=90  Identities=8%  Similarity=-0.285  Sum_probs=0.0

  Q ss_pred             CHHHHHHHHHHHHHHHHHHhCCCCCCccccccccccccccchhhceeeeeccccceecCCcceeeeccccccceeccCCC
  Q Q5E705_VIBF1/1  141 DVLEMTKQFDLFTIGVNEFYGRDGVSGAHKMFTDSAPELEYQNMRLVDDIQISIGSAHSGYPVMSTSFPRQKSSLFKATD  220 (302)
  Q Consensus       141 d~~~l~~~~d~ii~~~~~l~Gl~~~~~~~~~~~~~~~~~~~~~~~~~~~~~~~~~~~~~g~~~~~~~~~~~~~~~~~~~~  220 (302)
                        ...+.-+...+.++...+..++..........................................................
  T Consensus        24 ~~~~~~~~i~~A~~~W~~~~~i~F~~v~~~~~~~~~~~~~~~~~~~~~~~~~~~~~~~~~~~~~~~~~~~~~~~~~~~~~  103 (159)
  T 2ovx_A           24 PRAVIDDAFARAFALWSAVTPLTFTRVYSRDADIVIQFGVAEHGDGYPFDGKDGLLAHAFPPGPGIQGDAHFDDDELWSL  103 (159)
  T ss_dssp             CHHHHHHHHHHHHHHHHTSSSCEEEECSSTTSSEEEEEECSSCSSSCCCCSSSSCCEEECCSSSTTTTCEEEETTSCEEC
  T ss_pred             CHHHHHHHHHHHHHHHHhhcCceEEEeccCCCCEEEEeeeeccCCCccccccccccccccccccccccceeeccchhccc


  Q ss_pred             cc--------hhHHhhhh
  Q Q5E705_VIBF1/1  221 NW--------MLGHEIGH  230 (302)
  Q Consensus       221 ~W--------G~~HEiGH  230 (302)
                        .+        .+.|||||
  T Consensus       104 ~~~~~~~~~~v~~HEiGH  121 (159)
  T 2ovx_A          104 GKGQGYSLFLVAAHQFGH  121 (159)
  T ss_dssp             SSSSSEEHHHHHHHHHHH
  T ss_pred             CCccccceeEEeeeeccc


  No 48 
>2jsd_A Matrix metalloproteinase-20; MMP-NNGH, structural genomics, structural proteomics in europe, spine,
  spine-2, spine2-complexes, hydrolase; HET: NGH; NMR {Homo sapiens}
  Probab=59.46  E-value=59  Score=28.21  Aligned_cols=90  Identities=11%  Similarity=0.048  Sum_probs=0.0

  Q ss_pred             CHHHHHHHHHHHHHHHHHHhCCC--CCCccccccccccccccchhhceeeeeccccceecCCcceeeeccccccceeccC
  Q Q5E705_VIBF1/1  141 DVLEMTKQFDLFTIGVNEFYGRD--GVSGAHKMFTDSAPELEYQNMRLVDDIQISIGSAHSGYPVMSTSFPRQKSSLFKA  218 (302)
  Q Consensus       141 d~~~l~~~~d~ii~~~~~l~Gl~--~~~~~~~~~~~~~~~~~~~~~~~~~~~~~~~~~~~~g~~~~~~~~~~~~~~~~~~  218 (302)
                        +..+.-+...+.++..++..++.  +..............................+..+....................
  T Consensus        22 ~~~~~~~~i~~A~~~W~~~~~i~F~ev~~~~~~i~~~~~~~~~~~~~~~~~~~~~~~~~~~~~~~~~~~~~~~~~~~~~~  101 (160)
  T 2jsd_A           22 SSVEVDKAVEMALQAWSSAVPLSFVRINSGEADIMISFENGDHGDSYPFDGPRGTLAHAFAPGEGLGGDTHFDNAEKWTM  101 (160)
  T ss_dssp             CHHHHHHHHHHHHHHHHHHSSCEEEECSSSCCSEEEEEECSCSSSSSCCCSSSSCSEEECCSSSSSTTCEEEETTSCEES
  T ss_pred             CHHHHHHHHHHHHHHHhcCCCceEEEEcCCCCCeEEEeeeccCCCcccccccccceeccccCCCcccceeeecccccccc


  Q ss_pred             CCcc-----hhHHhhhh
  Q Q5E705_VIBF1/1  219 TDNW-----MLGHEIGH  230 (302)
  Q Consensus       219 ~~~W-----G~~HEiGH  230 (302)
                        ...+     -+.|||||
  T Consensus       102 ~~~~~~~~~v~~HEiGH  118 (160)
  T 2jsd_A          102 GTNGFNLFTVAAHEFGH  118 (160)
  T ss_dssp             SSSSEEHHHHHHHHHHH
  T ss_pred             ccCCceeEEEeeeeccc


  No 49 
>SUPFAM template d.92.1 Metalloproteases ("zincins"), catalytic domain (55486) SCOP seed sequence: d1kufa_.
  Probab=59.27  E-value=11  Score=31.06  Aligned_cols=9  Identities=56%  Similarity=0.981  Sum_probs=0.0

  Q ss_pred             hHHhhhhhc
  Q Q5E705_VIBF1/1  224 LGHEIGHNQ  232 (302)
  Q Consensus       224 ~~HEiGH~~  232 (302)
                        ..|||||+.
  T Consensus       140 ~~HE~GH~L  148 (201)
  T SUPFAM0040267   140 AAHELGHNL  148 (201)
  T ss_pred             HHHHhHHHh


  No 50 
>d1rm8a_ d.92.1.11 (A:) Matrix metalloproteinase-16 (MMP-16) {Human (Homo sapiens) [TaxId: 9606]}
  Probab=58.93  E-value=11  Score=32.60  Aligned_cols=9  Identities=44%  Similarity=0.793  Sum_probs=0.0

  Q ss_pred             chhHHhhhh
  Q Q5E705_VIBF1/1  222 WMLGHEIGH  230 (302)
  Q Consensus       222 WG~~HEiGH  230 (302)
                        +.++|||||
  T Consensus       119 ~v~~HEiGH  127 (169)
  T d1rm8a_         119 LVAVHELGH  127 (169)
  T ss_dssp             HHHHHHHHH
  T ss_pred             hhhhhhhhh


  No 51 
>SUPFAM template d.92.1 Metalloproteases ("zincins"), catalytic domain (55486) SCOP seed sequence: d1wnia_.
  Probab=57.23  E-value=13  Score=30.55  Aligned_cols=9  Identities=67%  Similarity=1.173  Sum_probs=0.0

  Q ss_pred             hHHhhhhhc
  Q Q5E705_VIBF1/1  224 LGHEIGHNQ  232 (302)
  Q Consensus       224 ~~HEiGH~~  232 (302)
                        ..|||||+.
  T Consensus       138 ~~HelGH~l  146 (198)
  T SUPFAM0044993   138 LAHELGHNL  146 (198)
  T ss_pred             HHHHHHHHc


  No 52 
>cd04271 ZnMc_ADAM_fungal Zinc-dependent metalloprotease, ADAM_fungal subgroup. The adamalysin_like or ADAM (A
  Disintegrin And Metalloprotease) family of metalloproteases are integral membrane proteases acting on a variety
  of extracellular targets. They are involved in shedding soluble peptides or proteins from the cell surface. This
  subfamily contains fungal ADAMs, whose precise function has yet to be determined.
  Probab=56.95  E-value=8.1  Score=35.94  Aligned_cols=9  Identities=56%  Similarity=0.984  Sum_probs=0.0

  Q ss_pred             hHHhhhhhc
  Q Q5E705_VIBF1/1  224 LGHEIGHNQ  232 (302)
  Q Consensus       224 ~~HEiGH~~  232 (302)
                        ++||+||++
  T Consensus       149 ~AHEiGHnf  157 (228)
  T cd04271         149 FAHEIGHTF  157 (228)
  T ss_pred             eeehhhccc


  No 53 
>cd04279 ZnMc_MMP_like_1 Zinc-dependent metalloprotease; MMP_like sub-family 1. A group of bacterial, archaeal,
  and fungal metalloproteinase domains similar to matrix metalloproteinases and astacin.
  Probab=56.81  E-value=43  Score=29.48  Aligned_cols=89  Identities=6%  Similarity=-0.163  Sum_probs=0.0

  Q ss_pred             HHHHHHHHHHHHHHHHHHhCCCCCCccccccccccccccchhhceeeeeccccceecCCcceeeeccccccceeccCCCc
  Q Q5E705_VIBF1/1  142 VLEMTKQFDLFTIGVNEFYGRDGVSGAHKMFTDSAPELEYQNMRLVDDIQISIGSAHSGYPVMSTSFPRQKSSLFKATDN  221 (302)
  Q Consensus       142 ~~~l~~~~d~ii~~~~~l~Gl~~~~~~~~~~~~~~~~~~~~~~~~~~~~~~~~~~~~~g~~~~~~~~~~~~~~~~~~~~~  221 (302)
                        .....+...+.+..-.+...|.......................+...-...+...+.......................
  T Consensus        19 ~~~~~~Ai~~Af~~Ws~v~~l~fve~~~~~~~adI~I~f~~~~~~dg~gg~lA~a~~p~~~~~~~~~~~~~~~~~~~~~~   98 (156)
  T cd04279          19 AQSWLQAVKQAAAEWENVGPLKFVYNPEEDNDADIVIFFDRPPPVGGAGGGLARAGFPLISDGNRKLFNRTDINLGPGQP   98 (156)
  T ss_pred             HHHHHHHHHHHHHHHhccCCceEEEecCCCCCCCEEEEecCCCCCCCCCCcceEecCCCCCCCCccccceeeeeecccCC


  Q ss_pred             -------chhHHhhhh
  Q Q5E705_VIBF1/1  222 -------WMLGHEIGH  230 (302)
  Q Consensus       222 -------WG~~HEiGH  230 (302)
                               +...|||||
  T Consensus        99 ~~~~~l~~vA~HEiGH  114 (156)
  T cd04279          99 RGAENLQAIALHELGH  114 (156)
  T ss_pred             ccchhHHHHHHHHhcc


  No 54 
>SUPFAM template d.92.1 Metalloproteases ("zincins"), catalytic domain (55486) SCOP seed sequence: d1g9ka2.
  Probab=56.55  E-value=8.4  Score=32.78  Aligned_cols=9  Identities=44%  Similarity=0.829  Sum_probs=0.0

  Q ss_pred             hHHhhhhhc
  Q Q5E705_VIBF1/1  224 LGHEIGHNQ  232 (302)
  Q Consensus       224 ~~HEiGH~~  232 (302)
                        +.|||||..
  T Consensus       165 ~~HE~GH~L  173 (242)
  T SUPFAM0038010   165 AAHELGHAL  173 (242)
  T ss_pred             eeeehhhhh


  No 55 
>PIRSF020485 PA-IL 
  Probab=56.50  E-value=24  Score=29.71  Aligned_cols=15  Identities=13%  Similarity=0.209  Sum_probs=0.0

  Q ss_pred             ccceEEECCCCEEEE
  Q Q5E705_VIBF1/1    4 QSAGVWIPAREVAYV   18 (302)
  Q Consensus         4 ~~TG~y~~~Ge~i~V   18 (302)
                        |+|||.+.+|++|+|
  T Consensus        16 ~~T~lI~~~GD~I~~   30 (123)
  T PIRSF020485      16 KVTSLILKQGDVISV   30 (123)
  T ss_pred             ceeEEEEcCCCEEEE


  No 56 
>1bud_A Protein (acutolysin A); metalloproteinase, snake venom, MMP, toxin; 1.90A {Deinagkistrodon acutus} SCOP:
  d.92.1.9 PDB:  1bsw _A
  Probab=55.73  E-value=14  Score=33.28  Aligned_cols=9  Identities=56%  Similarity=0.936  Sum_probs=0.0

  Q ss_pred             hHHhhhhhc
  Q Q5E705_VIBF1/1  224 LGHEIGHNQ  232 (302)
  Q Consensus       224 ~~HEiGH~~  232 (302)
                        +.|||||++
  T Consensus       137 iAHElGH~l  145 (197)
  T 1bud_A          137 LAHEMAHNL  145 (197)
  T ss_dssp             HHHHHHHHT
  T ss_pred             hHHHhhcee


  No 57 
>SUPFAM template d.92.1 Metalloproteases ("zincins"), catalytic domain (55486) SCOP seed sequence: d1atla_.
  Probab=55.49  E-value=15  Score=30.34  Aligned_cols=9  Identities=67%  Similarity=1.173  Sum_probs=0.0

  Q ss_pred             hHHhhhhhc
  Q Q5E705_VIBF1/1  224 LGHEIGHNQ  232 (302)
  Q Consensus       224 ~~HEiGH~~  232 (302)
                        ..|||||+.
  T Consensus       138 ~~HelGH~L  146 (200)
  T SUPFAM0035195   138 LAHELGHNL  146 (200)
  T ss_pred             HHHHHHHHc


  No 58 
>d1xuca1 d.92.1.11 (A:104-272) Collagenase-3 (MMP-13) {Human (Homo sapiens) [TaxId: 9606]}
  Probab=55.31  E-value=14  Score=32.27  Aligned_cols=9  Identities=44%  Similarity=0.866  Sum_probs=0.0

  Q ss_pred             chhHHhhhh
  Q Q5E705_VIBF1/1  222 WMLGHEIGH  230 (302)
  Q Consensus       222 WG~~HEiGH  230 (302)
                        +-+.|||||
  T Consensus       115 ~v~~HEiGH  123 (169)
  T d1xuca1         115 LVAAHEFGH  123 (169)
  T ss_dssp             HHHHHHHHH
  T ss_pred             eehhhhhcc


  No 59 
>d1bswa_ d.92.1.9 (A:) Snake venom metalloprotease {Five-pace snake (Agkistrodon acutus), acutolysin A [TaxId:
  36307]}
  Probab=55.26  E-value=15  Score=33.17  Aligned_cols=9  Identities=56%  Similarity=0.936  Sum_probs=0.0

  Q ss_pred             hHHhhhhhc
  Q Q5E705_VIBF1/1  224 LGHEIGHNQ  232 (302)
  Q Consensus       224 ~~HEiGH~~  232 (302)
                        +.||+||++
  T Consensus       137 ~AHElGH~l  145 (197)
  T d1bswa_         137 LAHEMAHNL  145 (197)
  T ss_dssp             HHHHHHHHT
  T ss_pred             HHHHHHhhc


  No 60 
>SUPFAM template d.92.1 Metalloproteases ("zincins"), catalytic domain (55486) SCOP seed sequence: d1quaa_.
  Probab=55.24  E-value=15  Score=30.14  Aligned_cols=9  Identities=56%  Similarity=0.981  Sum_probs=0.0

  Q ss_pred             hHHhhhhhc
  Q Q5E705_VIBF1/1  224 LGHEIGHNQ  232 (302)
  Q Consensus       224 ~~HEiGH~~  232 (302)
                        ..||+||+.
  T Consensus       139 ~~He~GH~L  147 (197)
  T SUPFAM0042988   139 AAHELGHNL  147 (197)
  T ss_pred             HHHHHHHHh


  No 61 
>1slm_A Stromelysin-1; hydrolase, metalloprotease, fibroblast, collagen degradation; 1.90A {Homo sapiens} SCOP:
  a.20.1.2 d.92.1.11
  Probab=55.08  E-value=33  Score=32.88  Aligned_cols=90  Identities=10%  Similarity=-0.189  Sum_probs=0.0

  Q ss_pred             CHHHHHHHHHHHHHHHHHHhCCCCCCccccccccccccccchhhceeeeeccccceecCCcc-------eeeeccccccc
  Q Q5E705_VIBF1/1  141 DVLEMTKQFDLFTIGVNEFYGRDGVSGAHKMFTDSAPELEYQNMRLVDDIQISIGSAHSGYP-------VMSTSFPRQKS  213 (302)
  Q Consensus       141 d~~~l~~~~d~ii~~~~~l~Gl~~~~~~~~~~~~~~~~~~~~~~~~~~~~~~~~~~~~~g~~-------~~~~~~~~~~~  213 (302)
                        ...++-+...+.++...+++.+...........+...+..........-...++..+|+.++       +-+-.+..+..
  T Consensus       109 ~~~~vr~~i~~Af~~Ws~v~~l~F~Ev~~~~aDI~I~f~~~~h~~~~~fdg~~g~la~a~~p~~~~~g~i~fd~~e~w~~  188 (255)
  T 1slm_A          109 PKDAVDSAVEKALKVWEEVTPLTFSRLYEGEADIMISFAVREHGDFYPFDGPGNVLAHAYAPGPGINGDAHFDDDEQWTK  188 (255)
  T ss_dssp             CHHHHHHHHHHHHHHHHTTSSCEEEECSSSCCSEEEEEECSCCSSSCCCCSSSSBCEEECCSSSTTTTCEEEETTSCEES
  T ss_pred             CHHHHHHHHHHHhhhhhcccceEEEEecCCCCCEEEEEeccCCCccccccCCCCcceeeecccCCccceEEecccccccc


  Q ss_pred             eeccCCCcchhHHhhhh
  Q Q5E705_VIBF1/1  214 SLFKATDNWMLGHEIGH  230 (302)
  Q Consensus       214 ~~~~~~~~WG~~HEiGH  230 (302)
                        .....+--+-..|||||
  T Consensus       189 ~~~~~~l~~va~HEIGH  205 (255)
  T 1slm_A          189 DTTGTNLFLVAAHEIGH  205 (255)
  T ss_dssp             SSSSEEHHHHHHHHHHH
  T ss_pred             cccccchhhhhhhhhcc


  No 62 
>d1y93a1 d.92.1.11 (A:106-263) Macrophage elastase (MMP-12) {Human (Homo sapiens) [TaxId: 9606]}
  Probab=54.79  E-value=15  Score=31.52  Aligned_cols=9  Identities=56%  Similarity=0.778  Sum_probs=0.0

  Q ss_pred             hHHhhhhhc
  Q Q5E705_VIBF1/1  224 LGHEIGHNQ  232 (302)
  Q Consensus       224 ~~HEiGH~~  232 (302)
                        ++|||||..
  T Consensus       111 ~~HEiGHaL  119 (158)
  T d1y93a1         111 AVHEIGHSL  119 (158)
  T ss_dssp             HHHHHHHHT
  T ss_pred             hhhhhhhhc


  No 63 
>2w15_A Zinc metalloproteinase BAP1; hydrolase inhibitor complex, metal-binding, zinc-depending, metalloprotease,
  metalloproteinase/inhibitor complex; HET: WR2; 1.05A {Bothrops asper} PDB:  2w12 _A*  2w13 _A*  2w14 _A*  1nd1 _A
   3gbo _A
  Probab=54.65  E-value=15  Score=33.32  Aligned_cols=9  Identities=56%  Similarity=1.128  Sum_probs=0.0

  Q ss_pred             hHHhhhhhc
  Q Q5E705_VIBF1/1  224 LGHEIGHNQ  232 (302)
  Q Consensus       224 ~~HEiGH~~  232 (302)
                        ++|||||++
  T Consensus       140 ~AHElGH~l  148 (202)
  T 2w15_A          140 MAHELGHNL  148 (202)
  T ss_dssp             HHHHHHHHT
  T ss_pred             HHHHHHHhc


  No 64 
>d1bqqm_ d.92.1.11 (M:) Membrane-type matrix metalloproteinase (CDMT1-MMP) {Human (Homo sapiens) [TaxId: 9606]}
  Probab=54.33  E-value=1.6e+02  Score=25.60  Aligned_cols=92  Identities=4%  Similarity=-0.087  Sum_probs=0.0

  Q ss_pred             CHHHHHHHHHHHHHHHHHHhCCCCCCccccccccccccccchhhceeeeeccccceecCCcceeeecccc----------
  Q Q5E705_VIBF1/1  141 DVLEMTKQFDLFTIGVNEFYGRDGVSGAHKMFTDSAPELEYQNMRLVDDIQISIGSAHSGYPVMSTSFPR----------  210 (302)
  Q Consensus       141 d~~~l~~~~d~ii~~~~~l~Gl~~~~~~~~~~~~~~~~~~~~~~~~~~~~~~~~~~~~~g~~~~~~~~~~----------  210 (302)
                        ...+.-+...+.+....+..++.......................................+........          
  T Consensus        23 ~~~~~~~~i~~A~~~W~~~~~i~f~~~~~~~~~~~~~~~~~i~~~~~~~~~~~~~~~~~~~~~~~~~~~~~~~~~~~~~~  102 (174)
  T d1bqqm_          23 GEYATYEAIRKAFRVWESATPLRFREVPYAYIREGHEKQADIMIFFAEGFHGDSTPFDGEGGFLAHAYFPGPNIGGDTHF  102 (174)
  T ss_dssp             CHHHHHHHHHHHHHHHHHHSSCEEECCSCCSSCTTSSSCCSSEEEEECSCSSSSCCCCSSSSEEEEECCSCSTTTTCEEE
  T ss_pred             CHHHHHHHHHHHHHHHHHhcCcceeeecccccccccccccceEEEeccccCCCcccccCCCcceeeeccCCCCccceeee


  Q ss_pred             -ccceeccCCCc-------chhHHhhhhhc
  Q Q5E705_VIBF1/1  211 -QKSSLFKATDN-------WMLGHEIGHNQ  232 (302)
  Q Consensus       211 -~~~~~~~~~~~-------WG~~HEiGH~~  232 (302)
                         ...........       +-+.|||||..
  T Consensus       103 ~~~~~~~~~~~~~~~~~~~~v~~HEiGHaL  132 (174)
  T d1bqqm_         103 DSAEPWTVRNEDLNGNDIFLVAVHELGHAL  132 (174)
  T ss_dssp             ETTSCCBSTTSCCSSCBHHHHHHHHHHHTT
  T ss_pred             cccccccccccCCcchhhhhhhhccccccc


  No 65 
>SUPFAM template d.92.1 Metalloproteases ("zincins"), catalytic domain (55486) SCOP seed sequence: d4aig__.
  Probab=54.26  E-value=16  Score=30.23  Aligned_cols=9  Identities=67%  Similarity=1.173  Sum_probs=0.0

  Q ss_pred             hHHhhhhhc
  Q Q5E705_VIBF1/1  224 LGHEIGHNQ  232 (302)
  Q Consensus       224 ~~HEiGH~~  232 (302)
                        ..|||||+.
  T Consensus       139 ~~HelGH~L  147 (201)
  T SUPFAM0045583   139 LAHELGHNL  147 (201)
  T ss_pred             HHHHHHHHh


  No 66 
>pfam06262 DUF1025 Domain of unknown function (DUF1025). Family of bacterial protein with undetermined function.
    Probab=54.20  E-value=16  Score=29.23  Aligned_cols=9  Identities=56%  Similarity=0.818  Sum_probs=0.0

  Q ss_pred             hHHhhhhhc
  Q Q5E705_VIBF1/1  224 LGHEIGHNQ  232 (302)
  Q Consensus       224 ~~HEiGH~~  232 (302)
                        ..|||||++
  T Consensus        76 viHEiaHhf   84 (96)
  T pfam06262        76 VIHEIGHHF   84 (96)
  T ss_pred             HHHHHHHHc


  No 67 
>COG3824 Predicted Zn-dependent protease [General function prediction only]
  Probab=53.89  E-value=14  Score=30.72  Aligned_cols=9  Identities=67%  Similarity=0.900  Sum_probs=0.0

  Q ss_pred             hHHhhhhhc
  Q Q5E705_VIBF1/1  224 LGHEIGHNQ  232 (302)
  Q Consensus       224 ~~HEiGH~~  232 (302)
                        ..|||||++
  T Consensus       113 liHEIgHhF  121 (136)
  T COG3824         113 LIHEIGHHF  121 (136)
  T ss_pred             hhhhhhhhc


  No 68 
>1c7k_A NCNP, zinc endoprotease; alpha and beta protein, metalloproteinase, hydrolase; 1.00A {Streptomyces
  caespitosus} SCOP: d.92.1.1 PDB:  1kuh _A
  Probab=53.52  E-value=16  Score=30.95  Aligned_cols=7  Identities=57%  Similarity=0.990  Sum_probs=0.0

  Q ss_pred             hHHhhhh
  Q Q5E705_VIBF1/1  224 LGHEIGH  230 (302)
  Q Consensus       224 ~~HEiGH  230 (302)
                        ..|||||
  T Consensus        81 a~HEiGH   87 (132)
  T 1c7k_A           81 TAHETGH   87 (132)
  T ss_dssp             HHHHHHH
  T ss_pred             HHHHHhh


  No 69 
>d2ejqa1 d.92.1.17 (A:2-108) Uncharacterized protein TTHA0227 {Thermus thermophilus [TaxId: 274]}
  Probab=52.75  E-value=17  Score=29.87  Aligned_cols=9  Identities=33%  Similarity=0.571  Sum_probs=0.0

  Q ss_pred             hHHhhhhhc
  Q Q5E705_VIBF1/1  224 LGHEIGHNQ  232 (302)
  Q Consensus       224 ~~HEiGH~~  232 (302)
                        ++|||||++
  T Consensus        92 vvHEigHhf  100 (107)
  T d2ejqa1          92 MLHELRHHL  100 (107)
  T ss_dssp             HHHHHHHHH
  T ss_pred             HHHHHHHHH


  No 70 
>d2ovxa1 d.92.1.11 (A:110-443) Gelatinase B (MMP-9) {Human (Homo sapiens) [TaxId: 9606]}
  Probab=52.17  E-value=18  Score=31.23  Aligned_cols=9  Identities=33%  Similarity=0.693  Sum_probs=0.0

  Q ss_pred             hHHhhhhhc
  Q Q5E705_VIBF1/1  224 LGHEIGHNQ  232 (302)
  Q Consensus       224 ~~HEiGH~~  232 (302)
                        +.|||||..
  T Consensus       115 ~~HElGHaL  123 (159)
  T d2ovxa1         115 AAHQFGHAL  123 (159)
  T ss_dssp             HHHHHHHHT
  T ss_pred             ehhhhcccc


  No 71 
>1qua_A Acutolysin-C, hemorrhagin III; metalloprotease, hemorrhagic toxin, snake venom proteinase; 2.20A
  {Deinagkistrodon acutus} SCOP: d.92.1.9
  Probab=52.07  E-value=18  Score=32.70  Aligned_cols=9  Identities=56%  Similarity=1.128  Sum_probs=0.0

  Q ss_pred             hHHhhhhhc
  Q Q5E705_VIBF1/1  224 LGHEIGHNQ  232 (302)
  Q Consensus       224 ~~HEiGH~~  232 (302)
                        +.||+||++
  T Consensus       139 iAHElGH~l  147 (197)
  T 1qua_A          139 MAHELGHNL  147 (197)
  T ss_dssp             HHHHHHHHT
  T ss_pred             HHHHHHhhc


  No 72 
>cd04267 ZnMc_ADAM_like Zinc-dependent metalloprotease, ADAM_like or reprolysin_like subgroup. The
  adamalysin_like or ADAM family of metalloproteases contains proteolytic domains from snake venoms, proteases from the
  mammalian reproductive tract, and the tumor necrosis factor alpha convertase, TACE. ADAMs (A Disintegrin And
  Metalloprotease) are glycoproteins, which play roles in cell signaling, cell fusion, and cell-cell interactions.
  Probab=51.87  E-value=16  Score=33.08  Aligned_cols=9  Identities=56%  Similarity=1.128  Sum_probs=0.0

  Q ss_pred             hHHhhhhhc
  Q Q5E705_VIBF1/1  224 LGHEIGHNQ  232 (302)
  Q Consensus       224 ~~HEiGH~~  232 (302)
                        +.|||||+.
  T Consensus       137 ~AHElGH~l  145 (192)
  T cd04267         137 MAHELGHNL  145 (192)
  T ss_pred             hhhhhhhhc


  No 73 
>3b8z_A Protein adamts-5; alpha/beta, hydrolase; HET: 294; 1.40A {Homo sapiens} PDB:  3hyg _A*  3hy9 _A*  3hy7 _A*
   3ljt _A*
  Probab=51.66  E-value=18  Score=33.14  Aligned_cols=9  Identities=56%  Similarity=0.885  Sum_probs=0.0

  Q ss_pred             hHHhhhhhc
  Q Q5E705_VIBF1/1  224 LGHEIGHNQ  232 (302)
  Q Consensus       224 ~~HEiGH~~  232 (302)
                        +.||+||++
  T Consensus       145 ~AHElGH~l  153 (217)
  T 3b8z_A          145 VAHEIGHLL  153 (217)
  T ss_dssp             HHHHHHHHT
  T ss_pred             HHHHHhhcc


  No 74 
>d1r55a_ d.92.1.9 (A:) ADAM33 {Human (Homo sapiens) [TaxId: 9606]}
  Probab=51.63  E-value=18  Score=32.84  Aligned_cols=9  Identities=56%  Similarity=1.066  Sum_probs=0.0

  Q ss_pred             hHHhhhhhc
  Q Q5E705_VIBF1/1  224 LGHEIGHNQ  232 (302)
  Q Consensus       224 ~~HEiGH~~  232 (302)
                        +.|||||++
  T Consensus       136 ~AHElGH~l  144 (203)
  T d1r55a_         136 MAHEIGHSL  144 (203)
  T ss_dssp             HHHHHHHHT
  T ss_pred             HHHHHHHhc


  No 75 
>3ma2_D Matrix metalloproteinase-14; protein - protein complex, cleavage on PAIR of basic residue disulfide bond,
  membrane, metal-binding; 2.05A {Homo sapiens} PDB:  1bqq _M  1buv _M
  Probab=51.56  E-value=19  Score=31.59  Aligned_cols=9  Identities=44%  Similarity=0.689  Sum_probs=0.0

  Q ss_pred             hHHhhhhhc
  Q Q5E705_VIBF1/1  224 LGHEIGHNQ  232 (302)
  Q Consensus       224 ~~HEiGH~~  232 (302)
                        +.|||||..
  T Consensus       126 ~~HEiGHaL  134 (181)
  T 3ma2_D          126 AVHELGHAL  134 (181)
  T ss_dssp             HHHHHHHHT
  T ss_pred             HHhhhcccc


  No 76 
>d1qiba_ d.92.1.11 (A:) Gelatinase A {Human (Homo sapiens) [TaxId: 9606]}
  Probab=51.41  E-value=18  Score=31.21  Aligned_cols=7  Identities=57%  Similarity=1.070  Sum_probs=0.0

  Q ss_pred             hHHhhhh
  Q Q5E705_VIBF1/1  224 LGHEIGH  230 (302)
  Q Consensus       224 ~~HEiGH  230 (302)
                        +.|||||
  T Consensus       113 ~~HEiGH  119 (161)
  T d1qiba_         113 AAHEFGH  119 (161)
  T ss_dssp             HHHHHHH
  T ss_pred             eeecccc


  No 77 
>SUPFAM template d.92.1 Metalloproteases ("zincins"), catalytic domain (55486) SCOP seed sequence: d1sat_2.
  Probab=50.55  E-value=9.9  Score=32.24  Aligned_cols=7  Identities=57%  Similarity=0.918  Sum_probs=0.0

  Q ss_pred             hHHhhhh
  Q Q5E705_VIBF1/1  224 LGHEIGH  230 (302)
  Q Consensus       224 ~~HEiGH  230 (302)
                        +.|||||
  T Consensus       171 ~~HE~GH  177 (243)
  T SUPFAM0043540   171 AIHELGH  177 (243)
  T ss_pred             eeeecch


  No 78 
>PF07828 PA-IL:  PA-IL-like protein;  InterPro:   The members of this family are similar to the
  galactophilic lectin-1 expressed by P. aeruginosa ((PA-IL, Q05097 from SWISSPROT). Lectins recognising specific
  carbohydrates found on the surface of host cells are known to be involved in the initiation of infections by this
  organism. The protein is thought to be organised into an extensive network of beta-sheets, as is the case with
  many other lectins . ; PDB:  1uoj _D  2vxj _P  1oko _C  1l7l _A.
  Probab=50.53  E-value=12  Score=29.96  Aligned_cols=15  Identities=20%  Similarity=0.346  Sum_probs=0.0

  Q ss_pred             ccceEEECCCCEEEE
  Q Q5E705_VIBF1/1    4 QSAGVWIPAREVAYV   18 (302)
  Q Consensus         4 ~~TG~y~~~Ge~i~V   18 (302)
                        |+|||.+.+|+.|+|
  T Consensus        14 k~T~lI~~~GD~Isv   28 (121)
  T PF07828_consen   14 KVTGLILKQGDVISV   28 (121)
  T ss_dssp             -B-S-------BB--
  T ss_pred             ceeEEEecCCCEEEE


  No 79 
>SUPFAM template d.92.1 Metalloproteases ("zincins"), catalytic domain (55486) SCOP seed sequence: d1nd1a_.
  Probab=50.49  E-value=19  Score=29.65  Aligned_cols=9  Identities=56%  Similarity=0.981  Sum_probs=0.0

  Q ss_pred             hHHhhhhhc
  Q Q5E705_VIBF1/1  224 LGHEIGHNQ  232 (302)
  Q Consensus       224 ~~HEiGH~~  232 (302)
                        ..||+||+.
  T Consensus       140 ~aHelGH~L  148 (202)
  T SUPFAM0041314   140 AAHELGHNL  148 (202)
  T ss_pred             HHHHHHHHc


  No 80 
>2ejq_A Hypothetical protein TTHA0227; NPPSFA, national project on protein structural and functional analyses;
  2.08A {Thermus thermophilus} SCOP: d.92.1.17
  Probab=50.45  E-value=19  Score=30.68  Aligned_cols=9  Identities=33%  Similarity=0.571  Sum_probs=0.0

  Q ss_pred             hHHhhhhhc
  Q Q5E705_VIBF1/1  224 LGHEIGHNQ  232 (302)
  Q Consensus       224 ~~HEiGH~~  232 (302)
                        ++|||||++
  T Consensus        93 lvHEigHHf  101 (130)
  T 2ejq_A           93 MLHELRHHL  101 (130)
  T ss_dssp             HHHHHHHHH
  T ss_pred             HHHHHHHHH


  No 81 
>1yp1_A FII; FII hydrolase; 1.90A {Deinagkistrodon acutus}
  Probab=50.10  E-value=20  Score=32.68  Aligned_cols=9  Identities=56%  Similarity=1.128  Sum_probs=0.0

  Q ss_pred             hHHhhhhhc
  Q Q5E705_VIBF1/1  224 LGHEIGHNQ  232 (302)
  Q Consensus       224 ~~HEiGH~~  232 (302)
                        +.||+||++
  T Consensus       139 iAHElGH~l  147 (202)
  T 1yp1_A          139 MAHELGHNL  147 (202)
  T ss_dssp             HHHHHHHHT
  T ss_pred             HHHHHHhhc


  No 82 
>d1kufa_ d.92.1.9 (A:) Snake venom metalloprotease {Taiwan habu (Trimeresurus mucrosquamatus), atrolysin E
  [TaxId: 103944]}
  Probab=50.06  E-value=20  Score=32.56  Aligned_cols=9  Identities=56%  Similarity=1.069  Sum_probs=0.0

  Q ss_pred             hHHhhhhhc
  Q Q5E705_VIBF1/1  224 LGHEIGHNQ  232 (302)
  Q Consensus       224 ~~HEiGH~~  232 (302)
                        +.||+||++
  T Consensus       140 ~AHElGH~l  148 (201)
  T d1kufa_         140 MTHELGHNL  148 (201)
  T ss_dssp             HHHHHHHHT
  T ss_pred             HHHHHHHhc


  No 83 
>d1quaa_ d.92.1.9 (A:) Snake venom metalloprotease {Chinese five-pace snake (Agkistrodon acutus), acutolysin C
  [TaxId: 36307]}
  Probab=50.06  E-value=20  Score=32.41  Aligned_cols=9  Identities=56%  Similarity=1.128  Sum_probs=0.0

  Q ss_pred             hHHhhhhhc
  Q Q5E705_VIBF1/1  224 LGHEIGHNQ  232 (302)
  Q Consensus       224 ~~HEiGH~~  232 (302)
                        +.||+||++
  T Consensus       139 ~AHElGH~l  147 (197)
  T d1quaa_         139 MAHELGHNL  147 (197)
  T ss_dssp             HHHHHHHHT
  T ss_pred             HHHHHHhhc


  No 84 
>PF00413 Peptidase_M10:  Matrixin This Prosite motif covers only the active site.;  InterPro:  
  Metalloproteases are the most diverse of the four main types of protease, with more than 50 families identified to
  date. In these enzymes, a divalent cation, usually zinc, activates the water molecule. The metal ion is held in
  place by amino acid ligands, usually three in number. The known metal ligands are His, Glu, Asp or Lys and at
  least one other residue is required for catalysis, which may play an electrophillic role. Of the known
  metalloproteases, around half contain an HEXXH motif, which has been shown in crystallographic studies to form part of the
  metal-binding site . The HEXXH motif is relatively common, but can be more stringently defined for
  metalloproteases as 'abXHEbbHbc', where 'a' is most often valine or threonine and forms part of the S1' subsite in
  thermolysin and neprilysin, 'b' is an uncharged residue, and 'c' a hydrophobic residue. Proline is never found in this
  site, possibly because it would break the helical structure adopted by this motif in metalloproteases .  
  Peptidases are grouped into clans and families. Clans are groups of families for which there is evidence of common
  ancestry. Each clan is identified with two letters, the first representing the catalytic type of the families
  included in the clan (with the letter 'P' being used for a clan containing families of more than one of the
  catalytic types serine, threonine and cysteine). Some families cannot yet be assigned to clans, and when a formal
  assignment is required, such a family is described as belonging to clan A-, C-, M-, S-, T- or U-, according to
  the catalytic type. Some clans are divided into subclans because there is evidence of a very ancient divergence
  within the clan, for example MA(E), the gluzincins, and MA(M), the metzincins.   Families are grouped by their
  catalytic type, the first character representing the catalytic type: A, aspartic; C, cysteine; G, glutamic
  acid; M, metallo; S, serine; T, threonine; and U, unknown. The serine, threonine and cysteine peptidases utilise
  the amino acid as a nucleophile and form an acyl intermediate - these peptidases can also readily act as
  transferases. In the case of aspartic, glutamic and metallopeptidases, the nucleophile is an activated water molecule. 
    This group of metallopeptidases belong to the MEROPS peptidase families:     Family M10 (clan MA(M)),
  subfamily M10A - matrixin Family M12 (clan MA(M)), subfamily M12B - adamalysin   The protein fold of the peptidase
  domain for members of this family resembles that of thermolysin, the type example for clan MA.  Sequences having
  this domain are extracellular metalloproteases, such as collagenase and stromelysin, which degrade the
  extracellular matrix, are known as matrixins. They are zinc-dependent, calcium-activated proteases synthesised as
  inactive precursors (zymogens), which are proteolytically cleaved to yield the active enzyme , . All matrixins and
  related proteins possess 2 domains: an N-terminal domain, and a zinc-binding active site domain. The N-terminal
  domain peptide, cleaved during the activation step, includes a conserved PRCGVPDV octapeptide, known as the
  cysteine switch, whose Cys residue chelates the active site zinc atom, rendering the enzyme inactive. The active
  enzyme degrades components of the extracellular matrix, playing a role in the initial steps of tissue
  remodelling during morphogenesis, wound healing, angiogenesis and tumour invasion , . ; GO: 0004222 metalloendopeptidase
  activity, 0006508 proteolysis, 0005578 proteinaceous extracellular matrix; PDB:  1hv5 _B  1rm8 _A  1bqq _M  1buv _M
   1go7 _P  1go8 _P  1k7i _A  3hda _P  1k7q _A  3hbv _P  .... 
  Probab=50.05  E-value=17  Score=31.29  Aligned_cols=9  Identities=56%  Similarity=0.689  Sum_probs=0.0

  Q ss_pred             hHHhhhhhc
  Q Q5E705_VIBF1/1  224 LGHEIGHNQ  232 (302)
  Q Consensus       224 ~~HEiGH~~  232 (302)
                        +.|||||..
  T Consensus       106 ~~HEiGHaL  114 (152)
  T PF00413_consen  106 AIHEIGHAL  114 (152)
  T ss_dssp             HHHH-----
  T ss_pred             chhhhcccc


  No 85 
>1kuf_A Atrolysin E, metalloproteinase; alpha/beta protein, hydrolase; 1.35A {Protobothrops mucrosquamatus} SCOP:
  d.92.1.9 PDB:  1kui _A  1kuk _A  1kug _A  1wni _A
  Probab=49.83  E-value=20  Score=32.56  Aligned_cols=9  Identities=56%  Similarity=1.069  Sum_probs=0.0

  Q ss_pred             hHHhhhhhc
  Q Q5E705_VIBF1/1  224 LGHEIGHNQ  232 (302)
  Q Consensus       224 ~~HEiGH~~  232 (302)
                        +.||+||++
  T Consensus       142 ~AHElGH~l  150 (203)
  T 1kuf_A          142 MTHELGHNL  150 (203)
  T ss_dssp             HHHHHHHHT
  T ss_pred             HHHHHhhhc


  No 86 
>d1nd1a_ d.92.1.9 (A:) Snake venom metalloprotease {Terciopelo (Bothrops asper), bap1 [TaxId: 8722]}
  Probab=49.82  E-value=20  Score=32.58  Aligned_cols=9  Identities=56%  Similarity=1.128  Sum_probs=0.0

  Q ss_pred             hHHhhhhhc
  Q Q5E705_VIBF1/1  224 LGHEIGHNQ  232 (302)
  Q Consensus       224 ~~HEiGH~~  232 (302)
                        +.||+||++
  T Consensus       140 ~AHElGH~l  148 (202)
  T d1nd1a_         140 MAHELGHNL  148 (202)
  T ss_dssp             HHHHHHHHT
  T ss_pred             HHHHHHhhc


  No 87 
>PF01433 Peptidase_M1:  Peptidase family M1 This is family M1 in the peptidase classification. ;  InterPro:   Metalloproteases are the most diverse of the four main types of protease, with more than 50 families
  identified to date. In these enzymes, a divalent cation, usually zinc, activates the water molecule. The metal ion
  is held in place by amino acid ligands, usually three in number. The known metal ligands are His, Glu, Asp or
  Lys and at least one other residue is required for catalysis, which may play an electrophillic role. Of the
  known metalloproteases, around half contain an HEXXH motif, which has been shown in crystallographic studies to
  form part of the metal-binding site . The HEXXH motif is relatively common, but can be more stringently defined
  for metalloproteases as 'abXHEbbHbc', where 'a' is most often valine or threonine and forms part of the S1'
  subsite in thermolysin and neprilysin, 'b' is an uncharged residue, and 'c' a hydrophobic residue. Proline is never
  found in this site, possibly because it would break the helical structure adopted by this motif in
  metalloproteases .   Peptidases are grouped into clans and families. Clans are groups of families for which there is
  evidence of common ancestry. Each clan is identified with two letters, the first representing the catalytic type of
  the families included in the clan (with the letter 'P' being used for a clan containing families of more than
  one of the catalytic types serine, threonine and cysteine). Some families cannot yet be assigned to clans, and
  when a formal assignment is required, such a family is described as belonging to clan A-, C-, M-, S-, T- or U-,
  according to the catalytic type. Some clans are divided into subclans because there is evidence of a very
  ancient divergence within the clan, for example MA(E), the gluzincins, and MA(M), the metzincins.   Families are
  grouped by their catalytic type, the first character representing the catalytic type: A, aspartic; C, cysteine; G,
  glutamic acid; M, metallo; S, serine; T, threonine; and U, unknown. The serine, threonine and cysteine
  peptidases utilise the amino acid as a nucleophile and form an acyl intermediate - these peptidases can also readily
  act as transferases. In the case of aspartic, glutamic and metallopeptidases, the nucleophile is an activated
  water molecule.    This group of metallopeptidases belong to the MEROPS peptidase family M1 (clan MA(E)), the
  type example being aminopeptidase N from Homo sapiens. The protein fold of the peptidase domain for members of
  this family resembles that of thermolysin, the type example for clan MA.   Membrane alanine aminopeptidase
  (3.4.11.2 from EC) is part of the HEXXH^+E group; it consists entirely of aminopeptidases, spread across a wide
  variety of species . Functional studies show that CD13/APN catalyzes the removal of single amino acids from the amino
  terminus of small peptides and probably plays a role in their final digestion; one family member
  (leukotriene-A4 hydrolase) is known to hydrolyse the epoxide leukotriene-A4 to form an inflammatory mediator . This
  hydrolase has been shown to have aminopeptidase activity , and the zinc ligands of the M1 family were identified by
  site-directed mutagenesis on this enzyme  CD13 participates in trimming peptides bound to MHC class II molecules 
  and cleaves MIP-1 chemokine, which alters target cell specificity from basophils to eosinophils . CD13 acts as
  a receptor for specific strains of RNA viruses (coronaviruses) which cause a relatively large percentage of
  upper respiratory trace infections.   CD molecules are leucocyte antigens on cell surfaces. CD antigens
  nomenclature is updated at Protein Reviews On The Web (http://mpr.nci.nih.gov/prow/).  ; GO: 0008237 metallopeptidase
  activity, 0008270 zinc ion binding; PDB:  1z1w _A  1z5h _A  2gtq _A  3b3b _A  3b2x _A  2dq6 _A  2hpo _A  2dqm _A  2hpt _A  3b34 _A
   .... 
  Probab=49.71  E-value=1.9e+02  Score=29.64  Aligned_cols=100  Identities=11%  Similarity=-0.025  Sum_probs=0.0

  Q ss_pred             CHHHHHHHHHHHHHHHHHHhCCCCCCccccccccccccccchhhceeeeeccccceecCCcceeeeccccccceeccCCC
  Q Q5E705_VIBF1/1  141 DVLEMTKQFDLFTIGVNEFYGRDGVSGAHKMFTDSAPELEYQNMRLVDDIQISIGSAHSGYPVMSTSFPRQKSSLFKATD  220 (302)
  Q Consensus       141 d~~~l~~~~d~ii~~~~~l~Gl~~~~~~~~~~~~~~~~~~~~~~~~~~~~~~~~~~~~~g~~~~~~~~~~~~~~~~~~~~  220 (302)
                        .....++.-.++++.+.++.|++....               +..++.-+.+..+.|....-+.....   ....-....
  T Consensus       227 ~~~~~l~~~~~~l~~~~~~~g~pyP~~---------------~~~~v~~p~~~~~~me~~g~i~~~~~---~~l~~~~~~  288 (391)
  T PF01433_consen  227 QAERALDVAKDALDYFEEYFGIPYPFP---------------KLDIVAVPDFPFGGMENWGLITYSEN---FLLYDPDST  288 (391)
  T ss_dssp             GHHHHHHHHHHHHHHHHHH-----SSS---------------EEEEEEEST-----B--T-TEEEEGG---GTS--TTTT
  T ss_pred             HHHHHHHHHHHHHHHHHHhcCCCCCCc---------------cCCEEEecCcCcchhccceEEEEecc---ccccCCccc


  Q ss_pred             cc--------hhHHhhhhhccccccccCCCccch-hhHHHHHHHHHH
  Q Q5E705_VIBF1/1  221 NW--------MLGHEIGHNQAANWLNVVGAGETA-NNVLALYTQERN  258 (302)
  Q Consensus       221 ~W--------G~~HEiGH~~Q~~~~~~~g~~Evt-nNi~sl~~q~~~  258 (302)
                        .+        .+.||++|.-=.+..+-..+.+.| +.=|+.|....+
  T Consensus       289 ~~~~~~~~~~~iaHElaHqWfG~~Vt~~~w~~~WL~Eg~a~y~~~~~  335 (391)
  T PF01433_consen  289 DDSQKQRIARVIAHELAHQWFGNLVTPDWWKDLWLNEGFATYLEYLY  335 (391)
  T ss_dssp             THHHHHHHHHHHHHCCHGCT----SEESSGGGHHHHHHHHHHHHHHH
  T ss_pred             cccchhhhhhHHHHHHHHHHhhcEecccccCCEeHHHHHHHHHHHHH


  No 88 
>1rm8_A MMP-16, matrix metalloproteinase-16, MT3-MMP; membrane type - matrix metalloproteinase, batimastat,
  hydroxamate inhibitor, protease, hydrolase; HET: BAT; 1.80A {Homo sapiens} SCOP: d.92.1.11
  Probab=49.27  E-value=20  Score=31.06  Aligned_cols=9  Identities=44%  Similarity=0.689  Sum_probs=0.0

  Q ss_pred             hHHhhhhhc
  Q Q5E705_VIBF1/1  224 LGHEIGHNQ  232 (302)
  Q Consensus       224 ~~HEiGH~~  232 (302)
                        +.|||||..
  T Consensus       121 ~~HEiGHaL  129 (169)
  T 1rm8_A          121 AVHELGHAL  129 (169)
  T ss_dssp             HHHHHHHHH
  T ss_pred             hhhhhhhhc


  No 89 
>d1sata2 d.92.1.6 (A:4-246) Metalloprotease {Serratia marcescens [TaxId: 615]}
  Probab=49.22  E-value=75  Score=30.22  Aligned_cols=86  Identities=13%  Similarity=-0.051  Sum_probs=0.0

  Q ss_pred             HHHHHHHHHHHHHHHHHhCCCCCCccccccccccccccchhhceeeeeccccceecCCcceeeeccccccceeccCCCcc
  Q Q5E705_VIBF1/1  143 LEMTKQFDLFTIGVNEFYGRDGVSGAHKMFTDSAPELEYQNMRLVDDIQISIGSAHSGYPVMSTSFPRQKSSLFKATDNW  222 (302)
  Q Consensus       143 ~~l~~~~d~ii~~~~~l~Gl~~~~~~~~~~~~~~~~~~~~~~~~~~~~~~~~~~~~~g~~~~~~~~~~~~~~~~~~~~~W  222 (302)
                        .+-.+...++++...+++++................+..............+.......  ..................|
  T Consensus        80 ~~q~~~~r~a~~~Ws~vani~F~ev~~~~~adi~~~~~~~~~~~~~~~~~~a~a~~p~~--~~~~~~~~~~~~~~~~~~~  157 (243)
  T d1sata2          80 AEQQQQAKLSLQSWADVANITFTEVAAGQKANITFGNYSQDRPGHYDYGTQAYAFLPNT--IWQGQDLGGQTWYNVNQSN  157 (243)
  T ss_dssp             HHHHHHHHHHHHHHHHHBSEEEEECCTTSCCSEEEEEECEEETTEECCSCCEEECCTTC--EETTEECTTEEEEETTSHH
  T ss_pred             HHHHHHHHHHHHHHHhhcCcEEEEecCCCceEEEEEeccCCCCCCCCCCcceeeecCCC--cccCccccccccccccccc


  Q ss_pred             ------------hhHHhhhh
  Q Q5E705_VIBF1/1  223 ------------MLGHEIGH  230 (302)
  Q Consensus       223 ------------G~~HEiGH  230 (302)
                                    -++|||||
  T Consensus       158 ~~~~~~g~~~~~t~lHEIGH  177 (243)
  T d1sata2         158 VKHPATEDYGRQTFTHEIGH  177 (243)
  T ss_dssp             HHCTTTCHHHHHHHHHHHHH
  T ss_pred             ccCCcccchHHHHHHHHHHH


  No 90 
>d3b7sa3 d.92.1.13 (A:209-460) Leukotriene A4 hydrolase catalytic domain {Human (Homo sapiens) [TaxId: 9606]}
  Probab=48.82  E-value=2.1e+02  Score=27.15  Aligned_cols=114  Identities=11%  Similarity=0.048  Sum_probs=0.0

  Q ss_pred             EcCcEEEEEEHHHHhhhcccCHHHHHHHHHHHHHHHHHHhCCCCCCccccccccccccccchhhceeee-eccccceecC
  Q Q5E705_VIBF1/1  121 VGKRFSYTTTTAGIKGHSEVDVLEMTKQFDLFTIGVNEFYGRDGVSGAHKMFTDSAPELEYQNMRLVDD-IQISIGSAHS  199 (302)
  Q Consensus       121 ~~~~v~~t~p~~~~~~~~~~d~~~l~~~~d~ii~~~~~l~Gl~~~~~~~~~~~~~~~~~~~~~~~~~~~-~~~~~~~~~~  199 (302)
                        +|.++.+-.+.+.+     ......++.--++++.+.++.|                ..+..+..++.- +.+..+.|-.
  T Consensus         6 ~g~~vrv~~~p~~~-----~~~~~~l~~~~~~l~~~e~~~g----------------~YP~~k~d~v~~~~~~~~ggmE~   64 (252)
  T d3b7sa3           6 IGPRTLVWSEKEQV-----EKSAYEFSETESMLKIAEDLGG----------------PYVWGQYDLLVLPPSFPYGGMEN   64 (252)
  T ss_dssp             EETTEEEEECGGGH-----HHHHHHTTTHHHHHHHHHHHHC----------------CCCSSCCEEEECCTTCSSSEECC
  T ss_pred             cCCceEEEEccchH-----HHHHHHHHHHHHHHHHHHHhCC----------------CCCchhcCEEEeCCCcccccccc


  Q ss_pred             CcceeeeccccccceeccCCCcchhHHhhhhhccccccccCCCccch-hhHHHHHHHHHHh
  Q Q5E705_VIBF1/1  200 GYPVMSTSFPRQKSSLFKATDNWMLGHEIGHNQAANWLNVVGAGETA-NNVLALYTQERNT  259 (302)
  Q Consensus       200 g~~~~~~~~~~~~~~~~~~~~~WG~~HEiGH~~Q~~~~~~~g~~Evt-nNi~sl~~q~~~~  259 (302)
                        ..-+............+.    ..+.||++|.-=.+..+...+.+.| |.=|+.|+...+.
  T Consensus        65 ~~l~~~~~~~~~~~~~~~----~~iaHE~aHqWfG~~Vt~~~w~~~WL~EG~a~y~~~~~~  121 (252)
  T d3b7sa3          65 PCLTFVTPTLLAGDKSLS----NVIAHQISHSWTGNLVTNKTWDHFWLNEGHTVYLERHIC  121 (252)
  T ss_dssp             TTEEEECGGGCCSSSTTT----HHHHHHHHTTTBTTTEEESSGGGHHHHHHHHHHHHHHHH
  T ss_pred             ceeeeecchhccccchHH----HHHHHHHHHHHHhhhceeccccchHhhccHHHHHHHHhh


  No 91 
>COG1164 Oligoendopeptidase F [Amino acid transport and metabolism]
  Probab=48.72  E-value=48  Score=36.10  Aligned_cols=82  Identities=12%  Similarity=-0.072  Sum_probs=0.0

  Q ss_pred             EEHHHHhhhcccCHHHHHHHHHHHHHHHHHHhCCCCCCccccccccccccccchhhceeeeeccccceecCCcceeeecc
  Q Q5E705_VIBF1/1  129 TTTAGIKGHSEVDVLEMTKQFDLFTIGVNEFYGRDGVSGAHKMFTDSAPELEYQNMRLVDDIQISIGSAHSGYPVMSTSF  208 (302)
  Q Consensus       129 ~p~~~~~~~~~~d~~~l~~~~d~ii~~~~~l~Gl~~~~~~~~~~~~~~~~~~~~~~~~~~~~~~~~~~~~~g~~~~~~~~  208 (302)
                        ++.++.++.+-.....|-..|.+++....+- +...-.+++-++                    +|||+++.+..  ...
  T Consensus       312 ~s~~ea~~~v~~~l~~lg~ey~~~~~~a~~~-~WiD~~~~~gKr--------------------sGaYs~~~~~~--~~p  368 (598)
  T COG1164         312 YSYEEAKELVLKALAPLGPEYAKIARRAFDE-RWIDVYPRKGKR--------------------SGAYSIGFYKG--DHP  368 (598)
  T ss_pred             ccHHHHHHHHHHHHHhhCHHHHHHHHHHHHc-CCcccccCCCCC--------------------CCcccCCCCCC--CCc


  Q ss_pred             ccccceeccCCCcchhHHhhhhhcc
  Q Q5E705_VIBF1/1  209 PRQKSSLFKATDNWMLGHEIGHNQA  233 (302)
  Q Consensus       209 ~~~~~~~~~~~~~WG~~HEiGH~~Q  233 (302)
                        ..-+.|.-...+-=.+.||+||.++
  T Consensus       369 ~IlmN~~gt~~dV~TLaHElGHs~H  393 (598)
  T COG1164         369 FILMNYDGTLRDVFTLAHELGHSVH  393 (598)
  T ss_pred             eEEecCCCchhhhhHHhhhhhHHHH


  No 92 
>3ba0_A Macrophage metalloelastase; FULL-length MMP-12, hemopexin domain, catalytic domain, domain interaction.,
  calcium, extracellular matrix; 3.00A {Homo sapiens} PDB:  2jxy _A
  Probab=48.54  E-value=55  Score=33.14  Aligned_cols=90  Identities=10%  Similarity=-0.274  Sum_probs=0.0

  Q ss_pred             CHHHHHHHHHHHHHHHHHHhCCCCCCccccccccccccccchhhceeeeeccccceecCCcc-------eeeeccccccc
  Q Q5E705_VIBF1/1  141 DVLEMTKQFDLFTIGVNEFYGRDGVSGAHKMFTDSAPELEYQNMRLVDDIQISIGSAHSGYP-------VMSTSFPRQKS  213 (302)
  Q Consensus       141 d~~~l~~~~d~ii~~~~~l~Gl~~~~~~~~~~~~~~~~~~~~~~~~~~~~~~~~~~~~~g~~-------~~~~~~~~~~~  213 (302)
                        ...+.-+...+.++...+.++|...........++-.+..........-...++..+++-.+       +....+.....
  T Consensus        21 ~~~~~~~~i~~Af~~Ws~v~~l~F~ev~~~~adi~I~f~~~~~~~~~~~~~~~~~~~~~~~~~~~~~~~i~~~~~~~~~~  100 (365)
  T 3ba0_A           21 NREDVDYAIRKAFQVWSNVTPLKFSKINTGMADILVVFARGAHGDDHAFDGKGGILAHAFGPGSGIGGDAHFDEDEFWTT  100 (365)
  T ss_dssp             CSHHHHHHHHHHHHHHHTTSSCCEEECSSSCCSEEEEEECSSCSSSSCCCSSSSCCEEECCSCSSSSSCEEEETTSCEES
  T ss_pred             CHHHHHHHHHHHHHHHhcccCcEEEEeCCCCccEEEEEecCcCCCCcccCCCcceEeccccCCCceeeeEEecccccccc


  Q ss_pred             eeccCCCcchhHHhhhh
  Q Q5E705_VIBF1/1  214 SLFKATDNWMLGHEIGH  230 (302)
  Q Consensus       214 ~~~~~~~~WG~~HEiGH  230 (302)
                        ..-....-.-+.|||||
  T Consensus       101 ~~~~~~~~~v~~HEiGH  117 (365)
  T 3ba0_A          101 HSGGTNLFLTAVHEIGH  117 (365)
  T ss_dssp             SSSSEESSHHHHHHHHH
  T ss_pred             CCCCceeEEEeeehhhh


  No 93 
>1k7iA02 3.40.390.10 
  Probab=48.08  E-value=52  Score=27.45  Aligned_cols=88  Identities=9%  Similarity=-0.078  Sum_probs=0.0

  Q ss_pred             HHHHHHHHHHHHHHHHHhCCCCCCccccccccccccccchhhce------eeeeccccceecCCcceeeeccccccceec
  Q Q5E705_VIBF1/1  143 LEMTKQFDLFTIGVNEFYGRDGVSGAHKMFTDSAPELEYQNMRL------VDDIQISIGSAHSGYPVMSTSFPRQKSSLF  216 (302)
  Q Consensus       143 ~~l~~~~d~ii~~~~~l~Gl~~~~~~~~~~~~~~~~~~~~~~~~------~~~~~~~~~~~~~g~~~~~~~~~~~~~~~~  216 (302)
                        .+.-+.....++...+++.|........................      .+...........+................
  T Consensus        64 ~~~~~~~~~al~~w~~v~~l~F~~~~~~~~~~~~~~~~~~~~~~~~~~~~~a~A~~P~~~~~~~~~~~~~~~~~~~~~~~  143 (227)
  T 1k7iA02          64 AEVKEAIKLALQVWSDVANLTFTEVSDGSDANITFGFYSLGDPGDLPGGTLAHAYLPGSGGIGGDAHFDIDEDWTLNPDL  143 (227)
  T ss_pred             hHHHHHHHHHHHhhhhhccceEEEecCCCcceEEEEeeccccccCCCcceeEEEEcCCCcccccceeecccccccccccc


  Q ss_pred             cCCCcchhHHhhhh
  Q Q5E705_VIBF1/1  217 KATDNWMLGHEIGH  230 (302)
  Q Consensus       217 ~~~~~WG~~HEiGH  230 (302)
                        ......-..||+||
  T Consensus       144 ~~~~~~~~~HE~GH  157 (227)
  T 1k7iA02         144 GNYLFLVAAHEIGH  157 (227)
  T ss_pred             cceeeeeeeeecch


  No 94 
>cd04272 ZnMc_salivary_gland_MPs Zinc-dependent metalloprotease, salivary_gland_MPs. Metalloproteases secreted by
  the salivary glands of arthropods.
  Probab=47.87  E-value=20  Score=33.34  Aligned_cols=9  Identities=33%  Similarity=0.593  Sum_probs=0.0

  Q ss_pred             hHHhhhhhc
  Q Q5E705_VIBF1/1  224 LGHEIGHNQ  232 (302)
  Q Consensus       224 ~~HEiGH~~  232 (302)
                        +.||+||+.
  T Consensus       149 ~AHElGHnL  157 (220)
  T cd04272         149 MTHELAHLL  157 (220)
  T ss_pred             hhhhhhhhc


  No 95 
>COG3590 PepO Predicted metalloendopeptidase [Posttranslational modification, protein turnover, chaperones]
  Probab=47.69  E-value=22  Score=37.62  Aligned_cols=20  Identities=40%  Similarity=0.654  Sum_probs=0.0

  Q ss_pred             hHHhhhhhccccccccCCCc
  Q Q5E705_VIBF1/1  224 LGHEIGHNQAANWLNVVGAG  243 (302)
  Q Consensus       224 ~~HEiGH~~Q~~~~~~~g~~  243 (302)
                        +.|||||+++..--.+.+.|
  T Consensus       491 IgHEI~HgFDdqGakfD~~G  510 (654)
  T COG3590         491 IGHEIGHGFDDQGAKFDGDG  510 (654)
  T ss_pred             ehhhhcccccCCccccCCCC


  No 96 
>d1atla_ d.92.1.9 (A:) Snake venom metalloprotease {Western diamonback rattlesnake (Crotalus atrox), atrolysin C
  [TaxId: 8730]}
  Probab=47.36  E-value=23  Score=32.12  Aligned_cols=9  Identities=56%  Similarity=1.128  Sum_probs=0.0

  Q ss_pred             hHHhhhhhc
  Q Q5E705_VIBF1/1  224 LGHEIGHNQ  232 (302)
  Q Consensus       224 ~~HEiGH~~  232 (302)
                        +.||+||++
  T Consensus       138 ~AHElGH~l  146 (200)
  T d1atla_         138 MAHELGHNL  146 (200)
  T ss_dssp             HHHHHHHHT
  T ss_pred             HHHHHHhhc


  No 97 
>2ddf_A ADAM 17; hydrolase; HET: INN CIT; 1.70A {Homo sapiens} PDB:  2fv5 _A*  3l0v _A*  3kme _A*  3l0t _A*  3kmc _A*
   3ewj _A*  3edz _A*  3e8r _A*  2fv9 _A*  1zxc _A*  2oi0 _A*  3b92 _A*  2a8h _A*  1bkc _A*  3cki _A  1bkc _I*  1bkc _E*
  Probab=47.05  E-value=24  Score=33.49  Aligned_cols=9  Identities=56%  Similarity=0.881  Sum_probs=0.0

  Q ss_pred             hHHhhhhhc
  Q Q5E705_VIBF1/1  224 LGHEIGHNQ  232 (302)
  Q Consensus       224 ~~HEiGH~~  232 (302)
                        +.|||||++
  T Consensus       186 ~AHElGH~l  194 (257)
  T 2ddf_A          186 TTHELGHNF  194 (257)
  T ss_dssp             HHHHHHHHT
  T ss_pred             HHHHhHHhc


  No 98 
>cd04273 ZnMc_ADAMTS_like Zinc-dependent metalloprotease, ADAMTS_like subgroup. ADAMs (A Disintegrin And
  Metalloprotease) are glycoproteins, which play roles in cell signaling, cell fusion, and cell-cell interactions. This
  particular subfamily represents domain architectures that combine ADAM-like metalloproteinases with
  thrombospondin type-1 repeats. ADAMTS (a disintegrin and metalloproteinase with thrombospondin motifs) proteinases are
  inhibited by TIMPs (tissue inhibitors of metalloproteinases), and they play roles in coagulation, angiogenesis,
  development and progression of arthritis. They hydrolyze the von Willebrand factor precursor and various
  components of the extracellular matrix.
  Probab=47.03  E-value=23  Score=32.69  Aligned_cols=9  Identities=44%  Similarity=0.907  Sum_probs=0.0

  Q ss_pred             hHHhhhhhc
  Q Q5E705_VIBF1/1  224 LGHEIGHNQ  232 (302)
  Q Consensus       224 ~~HEiGH~~  232 (302)
                        +.||+||+.
  T Consensus       144 ~AHElGHnL  152 (207)
  T cd04273         144 IAHELGHVL  152 (207)
  T ss_pred             HHHHHHhhc


  No 99 
>1atl_A Atrolysin C; metalloendopeptidase; HET: MTJ; 1.80A {Crotalus atrox} SCOP: d.92.1.9 PDB:  1htd _A  1dth _A*
   2aig _P*  3aig _A*  4aig _A*  1iag _A
  Probab=46.09  E-value=25  Score=31.98  Aligned_cols=9  Identities=56%  Similarity=1.128  Sum_probs=0.0

  Q ss_pred             hHHhhhhhc
  Q Q5E705_VIBF1/1  224 LGHEIGHNQ  232 (302)
  Q Consensus       224 ~~HEiGH~~  232 (302)
                        +.||+||++
  T Consensus       140 ~AHElGH~l  148 (202)
  T 1atl_A          140 MAHELGHNL  148 (202)
  T ss_dssp             HHHHHHHHT
  T ss_pred             HHHHHHHhc


  No 100
>COG1913 Predicted Zn-dependent proteases [General function prediction only]
  Probab=45.40  E-value=26  Score=31.33  Aligned_cols=10  Identities=40%  Similarity=0.527  Sum_probs=0.0

  Q ss_pred             hhHHhhhhhc
  Q Q5E705_VIBF1/1  223 MLGHEIGHNQ  232 (302)
  Q Consensus       223 G~~HEiGH~~  232 (302)
                        +..||+||.+
  T Consensus       127 Ev~HElGH~~  136 (181)
  T COG1913         127 EVLHELGHLL  136 (181)
  T ss_pred             HHHHHhhhhc


Done!
```

Please cite as appropriate:
  
  
***HHpred*: Söding, J. (2005) Protein homology detection by HMM-HMM comparison. Bioinformatics 21: 951-960.**  
  
*PSIPRED*: Jones, D.T. (1999) Protein secondary structure prediction based on position-specific scoring matrices. JMB 292: 195-202.  
  
*PDB*: Bourne, PE. *et al.* (2004) The distribution and query systems of the RCSB Protein Data Bank. NAR 32: D223.  
  
*SCOP*: Andreeva A, Howorth D, Brenner SE, Hubbard TJ, Chothia C, Murzin AG. (2004) SCOP database in 2004: refinements integrate structure and sequence family data. NAR 32: D226-229.  
  
*Interpro*: Mulder NJ, Apweiler R, Attwood TK, Bairoch A, Bateman A, Binns D, Bradley P, Bork P, Bucher P, Cerutti L, Copley R, Courcelle E, Das U, Durbin R, Fleischmann W, Gough J, Haft D, Harte N, Hulo N, Kahn D, Kanapin A, Krestyaninova M, Lonsdale D, Lopez R, Letunic I, Madera M, Maslen J, McDowall J, Mitchell A, Nikolskaya AN, Orchard S, Pagni M, Ponting CP, Quevillon E, Selengut J, Sigrist CJ, Silventoinen V, Studholme DJ, Vaughan R, Wu CH. (2005) InterPro, progress and status in 2005. NAR 33: D201-205.  
  
*Pfam*: Bateman A, Coin L, Durbin R, Finn RD, Hollich V, Griffiths-Jones S, Khanna A, Marshall M, Moxon S, Sonnhammer EL, Studholme DJ, Yeats C, Eddy SR. (2004) The Pfam protein families database. NAR 32: D138-141.  
  
*CDD*: Marchler-Bauer A, Anderson JB, Cherukuri PF, DeWeese-Scott C, Geer LY, Gwadz M, He S, Hurwitz DI, Jackson JD, Ke Z, Lanczycki CJ, Liebert CA, Liu C, Lu F, Marchler GH, Mullokandov M, Shoemaker BA, Simonyan V, Song JS, Thiessen PA, Yamashita RA, Yin JJ, Zhang D, Bryant SH. (2005) CDD: a Conserved Domain Database for protein classification.. NAR 33: D192-196.  
  
*COG*: Tatusov RL, Fedorova ND, Jackson JD, Jacobs AR, Kiryutin B, Koonin EV, Krylov DM, Mazumder R, Mekhedov SL, Nikolskaya AN, Rao BS, Smirnov S, Sverdlov AV, Vasudevan S, Wolf YI, Yin JJ, Natale DA. (2003) The COG database: an updated version includes eukaryotes. BMC Bioinformatics 4: 41.  
  
*PANTHER*: Mi H, Lazareva-Ulitsky B, Loo R, Kejariwal A, Vandergriff J, Rabkin S, Guo N, Muruganujan A, Doremieux O, Campbell MJ, Kitano H, Thomas PD. (2005) The PANTHER database of protein families, subfamilies, functions and pathways. NAR 33: D284-288.  
  
*PIRSF*: Wu CH, Nikolskaya A, Huang H, Yeh LS, Natale DA, Vinayaka CR, Hu ZZ, Mazumder R, Kumar S, Kourtesis P, Ledley RS, Suzek BE, Arminski L, Chen Y, Zhang J, Cardenas JL, Chung S, Castro-Alvear J, Dinkov G, Barker WC. (2004) PIRSF: family classification system at the Protein Information Resource. NAR 32: D112-114.  
  
*Superfamily*: Madera M, Vogel C, Kummerfeld SK, Chothia C and Gough J. (2004) The SUPERFAMILY database in 2004: additions and improvements. NAR 32: D235-D239.
